# Fatty Acid Biomarkers and Incidence of Type 2 diabetes: A Systematic Review and Dose–Response Meta-analysis of Prospective Observational Studies

**DOI:** 10.1016/j.advnut.2025.100565

**Published:** 2025-12-03

**Authors:** Edyta Schaefer, Manuela Neuenschwander, Tim Schiemann, Nadine Iser, Christina Baechle, Nafiseh Shokri-Mashhadi, Lukas Schwingshackl, Matthias B Schulze, Sabrina Schlesinger

**Affiliations:** 1 Institute for Biometrics and Epidemiology, German Diabetes Center, Leibniz Center for Diabetes Research at Heinrich Heine University Düsseldorf, Düsseldorf, Germany; 2 German Center for Diabetes Research, Neuherberg, Germany; c Institute of Nutritional and Food Science, Nutritional Physiology, University of Bonn, Bonn, Germany; 4 Institute for Evidence in Medicine, Faculty of Medicine and Medical Center–University of Freiburg, Freiburg, Germany; 5 Department of Molecular Epidemiology, German Institute of Human Nutrition Potsdam-Rehbruecke, Nuthetal, Germany; 6 Institute of Nutritional Science, University of Potsdam, Nuthetal, Germany

**Keywords:** biomarkers, fatty acids, type 2 diabetes, meta-analysis, prevention

## Abstract

The role of dietary fat in type 2 diabetes (T2D) development remains debated. Fatty acid (FA) biomarkers may better reflect bioavailable FAs than self-reported dietary intake. We conducted a systematic review and dose–response meta-analysis investigating associations between FA biomarkers and risk of T2D, considering different biospecimens of FA measurement. PubMed and Web of Science were searched until 9 November, 2022, and a search alert was followed until 17 February, 2025. Prospective cohort studies investigating FA biomarkers and T2D risk were included. Summary relative risks (SRR) and 95% confidence intervals (CIs) for all biospecimens combined and separately were estimated using a random-effects model. Risk of bias was assessed with the Risk Of Bias In Non-randomized Studies of Interventions tool, and the certainty of evidence (CoE) was rated with the Grades of Recommendations, Assessment, Development, and Evaluation approach. We included 27 articles. Analyses of plasma phospholipids (PPL) and red blood cells (RBC) provided inverse associations between higher concentrations of specific saturated FAs (SFAs) [15:0: SRR 0.68 (95% CI: 0.56, 0.81); 17:0: 0.64 (0.41, 0.78)], n−3 polyunsaturated FAs (PUFAs) [20:5: 0.97 (0.95, 0.99), 22:6: 0.92 (0.88, 0.96)], and n−6 PUFA [18:2: 0.93 (0.91, 0.95)] and risk of T2D, with moderate CoE. In the same biospecimens, higher levels of specific monounsaturated FAs (MUFAs) [16:1n−7: 1.06 (1.03, 1.10), 18:1n−9: 1.04 (1.01, 1.06)], and n−6 PUFAs [γ−20:3: 1.07 (1.03, 1.11), γ−18:3: 2.23 (1.42, 3.50), and 20:4: 1.02 (1.01, 1.04)] were associated with higher risk of T2D. Although combined analyses across biospecimens were consistent, stronger associations were observed in PPL and RBC. Associations of specific FAs within the same class (SFAs, MUFAs, PUFAs) varied in direction regarding risk of T2D. Certain SFAs, n−3 PUFAs, and 18:2 were inversely associated with T2D risk, whereas certain MUFAs and n−6 PUFAs were positively associated. Stronger associations in PPL and RBC highlight the importance of biospecimen selection. The protocol of this study was registered a priori in PROSPERO (CRD42020184575).

**Statements of Significance**

This systematic review and meta-analysis is the first to investigate dose–response associations between FA biomarkers and risk of T2D across different biospecimens, and the first to assess the CoE for these associations.

## Introduction

Guidelines for type 2 diabetes (T2D) prevention recommend increasing vegetable fats, lowering animal fats, replacing SFAs with MUFAs and PUFAs, and lowering *trans*-fatty acid (FAs) intake [1]. However, a recent systematic review with meta-analysis of randomized controlled trials suggested that higher intake of omega-3 (n−3), omega-6 (n−6) FAs, or total PUFA has little effect on T2D prevention [2]. Our recent systematic review and dose–response meta-analysis also did not find an increased risk of T2D with higher SFA intake [3], possibly because of differing associations for specific FAs within the SFA group. A large body of literature indicates that circulating SFAs, such as palmitic acid (16:0) and stearic acid (18:0) [4], exert lipotoxic effects by promoting ceramide and diacylglycerol accumulation in pancreatic ß-cells, impairing insulin signaling, and triggering inflammation [5]. In contrast, circulating MUFAs appear less harmful [6], and PUFAs may enhance insulin secretion by increasing membrane fluidity, facilitating glucose transporter (GLUT4) translocation, and reducing inflammation [[7], [8], [9]]. Together, these findings highlight the pivotal role of FAs in T2D development.

Circulating FAs measured in red blood cells (RBCs) or plasma show moderate-to-strong correlations with corresponding intake assessed via food frequency questionnaire [10]. These biomarkers reflect both diet and endogenous metabolism, offering a more objective measure of bioavailable FAs than self-reported dietary information alone [11]. Previous systematic reviews with meta-analyses combining studies that measured FAs in different biospecimens found that circulating odd-chain SFAs were linked to lower risk of T2D, whereas even-chain SFAs and palmitoleic acid (16:1n−7) concentrations were associated with higher risk of T2D [[12], [13], [14]]. No associations were observed for total MUFAs and oleic acid (18:1n−9) [14]. Higher concentrations of α-linolenic (α−18:3), linoleic acid (18:2), and total n−6 PUFA were associated with lower risk of T2D [[14], [15], [16]], whereas the association with total n−3 PUFA was imprecisely estimated, as indicated by wide 95% confidence intervals (CIs) [14]. However, not all of the existing systematic reviews and meta-analyses quantified dose–response associations [12,14] or covered the whole spectrum of FA [13,15,17]. Importantly, prior studies suggested that the biospecimen used for FA measurement may influence its association with the risk of T2D. Findings of the pooling project “Fatty Acids and Outcomes Research Consortium” suggest the strongest associations for odd-chain FAs, *trans*-FAs, and n−3 PUFAs measured in plasma phospholipids (PPL) compared with total plasma or cholesteryl ester [[18], [19], [20]]. Therefore, the influence of the biospecimen type on the associations between FA biomarkers and risk of T2D should be comprehensively evaluated at the highest available evidence level. However, none of the systematic reviews with meta-analyses published to date have captured the role of biospecimen type [[12], [13], [14], [15], [16], [17],^21^], nor have they assessed the confidence in the findings, expressed as the certainty of evidence (CoE) [[12], [13], [14], [15], [16], [17],^21^]. Moreover, several new studies on this topic have been published since the publication of the most recent systematic reviews and meta-analyses [19,20,22,23].

Therefore, our study aimed to examine the association between all FA biomarkers, stratified by the type of biospecimen, with the risk of T2D in a systematic review and dose–response meta-analysis of prospective observational studies.

## Methods

The present systematic review and meta-analysis was planned, conducted, and reported according to the PRISMA 2020 statement (Supplemental Table 1). The protocol of this study was registered a priori with PROSPERO (CRD42020184575).

### Search strategy and study selection process

We included studies if the following criteria were met: *1*) adult participants (≥18 y) were free of T2D at the study begin, *2*) FA concentrations were measured in blood (total plasma, PPL, RBC, cholesteryl ester, and triacylglycerol measured in plasma or serum, total serum, whole blood, without further differentiation between more detailed fractions, e.g., PPL subfractions) or in adipose tissue as exposure, *3*) the outcome of interest was T2D incidence, and *4*) the study design was a prospective observational study (including cohort, nested case-control, case-cohort studies, and follow-up of intervention studies) published in a peer-reviewed journal.

The systematic literature search was performed in PubMed and Web of Science using predetermined search terms, from inception to 9 November, 2022. We continued screening articles from the PubMed search alert until 17 February, 2025 to identify newly published studies. No restrictions regarding publication language or filters were applied. Two researchers independently conducted the entire study selection process. Initially, titles and abstracts of all identified articles were screened, followed by retrieval and examination of the full-texts of potentially relevant studies based on the inclusion criteria. Disagreements between the 2 researchers were resolved through consensus or consultation with a third researcher. Additionally, reference lists of all eligible articles were reviewed to identify any additional relevant studies. The full search strategy is provided in Supplemental Table 2.

In the meta-analysis combining specific FAs measured in various biospecimens, we selected results based on the ability of the biospecimen to reflect long-term dietary intake in the case of duplicate cohorts. Biospecimens of FAs measurement were ranked according to their ability to reflect long-term intake as follows: *1*) adipose tissue, *2*) RBC membrane phospholipids, *3*) serum phospholipids or PPL, *4*) whole blood, *5*) total plasma/serum, and *6*) cholesteryl esters [24]. For the meta-analyses stratified by biospecimen type, when duplicate cohorts were available for a particular FA, we included the most recent result based on the largest sample size. If 2 reports from the same cohort were available for the same FA but used different units of measurement, we prioritized the measurement expressed as a % of total FA concentration. If only absolute concentration values were available, we converted them to % of total FA concentration. Studies with insufficient data for the calculation of dose–response meta-analyses were excluded.

### Data extraction

One reviewer extracted the data from all identified studies using a predefined data extraction form, and a second reviewer checked the data for accuracy. The following characteristics were extracted from each study: last name of the first author, year of publication, country where the study was conducted, cohort name, duration of follow-up, baseline participant characteristics (sex, age), total number of participants, number of T2D cases, outcome assessment (self-report of diabetes with or without objective medical details, use of diabetes medication, blood test, medical records), exposure (group of FAs, single FA), biospecimen type (RBC, PPL, total serum, total plasma, cholesteryl ester, whole blood, adipose tissue), categories of exposure with measured concentration of a given FA, and maximally adjusted risk estimates expressed as hazard ratio, relative risk (RR) or odds ratio with their corresponding 95% CI and the adjustment factors. For 7 studies, relevant data were missing, and the authors were contacted; 5 of them provided the necessary information [22, [25], [26], [27], [28]].

### Risk of bias and CoE

We evaluated the risk of bias (RoB) using the RoB in nonrandomized studies of interventions tool [29]. Supplemental Table 3 contains detailed information on the rating. RoB was assessed by 2 reviewers independently, and any discrepancies were discussed with a third researcher to reach a consensus.

The CoE for each meta-analysis outcome was assessed using the Grades of Recommendations, Assessment, Development, and Evaluation approach [30]. Supplemental Table 4 contains the description of the instrument and rating judgments. Two reviewers independently rated the CoE, and any disagreements were resolved by consensus or consultation with a third researcher.

### Statistical analysis

Summary relative risks (SRR) and corresponding 95% CI were calculated by conducting dose–response meta-analyses with a random-effects model, applying the method of DerSimonian and Laird [31]. First, we performed dose–response meta-analyses for FA concentrations and risk of T2D, combining all biospecimens. Next, we conducted dose–response meta-analyses for specific FA concentrations measured in the same biospecimen (e.g., RBC or PPL) and risk of T2D, when 2 or more studies were available. To conduct dose–response meta-analyses, for studies that did not report linear estimates, we applied the method described by Greenland and Longnecker [32] and computed study-specific slopes (linear trends) and 95% CIs based on the natural logarithm of the RRs and 95% CIs across categories of each FA. The following data were required for ≥3 exposure categories: the quantified exposure value (e.g., % of total FAs), the risk ratio with the corresponding 95% CI, and the number of cases and person-years. If the number of cases in single categories was not provided in a study, information on the total number of cases and total person-years or the number of total persons plus follow-up period was used to equally distribute the number of cases across the quantiles or categories as described previously [33]. If a range of FA concentration were presented, the midpoint value was assigned as the exposure level for the respective category. For open categories, we assumed the same width as the adjacent category. Because circulating FAs concentrations vary widely [34], we harmonized the dose–response estimates to specific FAs or their groups to make RRs directly comparable across specific FAs with differing abundances. Specifically, groups of FAs, such as total SFAs, total MUFAs, or total n−3 and n−6 PUFAs, were analyzed per 3.0% higher concentration in total FA. Specific FAs, with large relative concentrations, including 16:0, 18:0, 18:1n−9, 18:2, arachidonic acid (20:4), and DHA (22:6), were analyzed per 1.0% higher concentration in total FAs. For the remaining FAs, the calculations were conducted per 0.1% of total FAs concentration [34,35]. A nonlinear dose–response meta-analysis could not be conducted because the data from the original studies were insufficient and only available from very few cohorts. We calculated the I^2^ statistic to assess the heterogeneity among the observed RRs in the meta-analyses. Potential publication bias was evaluated using funnel plots and Egger’s test (*P* < 0.1) when ≥10 studies were available.

For meta-analyses including ≥10 cohorts, we conducted subgroup analysis in the biospecimen-specific meta-analyses by continent of the study origin. A *P* value <0.05 was considered statistically significant. To assess the robustness of our findings, we excluded studies with high RoB and omitted 1 study at a time to investigate the influence of individual studies on the results.

All analyses were performed using Stata (Version 14.2, StataCorp).

### Patient and public involvement

No patients or members of the public were involved in setting the research question, designing the study, interpreting the results, or writing the manuscript. The results of this study will be spread to the public via social media or a press release.

## Results

**FIGURE 1:**
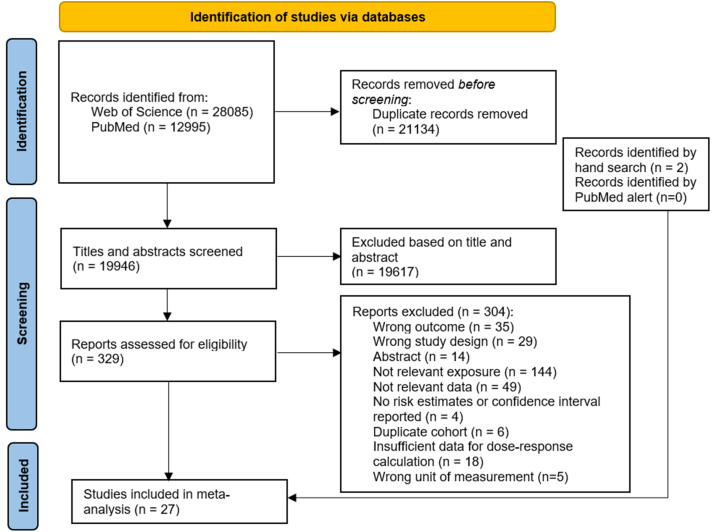
Flow-chart of the systematic literature search.

In total, we identified 19,946 articles after removing duplicates (Figure 1), of which 329 articles were assessed at the full-text level. Out of these, 25 articles met our inclusion criteria. In addition, we identified 2 articles through reference screening [36,37], whereas no eligible articles were identified via the PubMed alert. Thus, 27 articles were included in the present systematic review and meta-analyses. Articles excluded after full-text reviewing, with corresponding reasons for exclusion, are listed in Supplemental Table 5.

Supplemental Table 6 presents the characteristics of the included studies**.** The mean follow-up time ranged from 2.5 to 42.3 y, and the number of participants ranged from 187 to 95,854. The number of incidents T2D cases ranged from 28 to 12,132, and the mean age of participants ranged from 49 to 82 y. A total of 16 cohorts were conducted in Europe, 9 in the United States, 4 in Asia, and 2 in Australia. Six cohorts included only men, and 2 only women. FA biomarkers were measured in total plasma, total serum, PPL, RBC, plasma cholesteryl ester, whole blood, and adipose tissue. Quantification of FAs was conducted mainly by gas chromatography, whereas in 2 cohorts a targeted nuclear magnetic resonance metabolomics platform was used [22,23].

RoB was moderate in *n* = 19 studies and serious in *n* = 8 studies (Supplemental Figure 1). In the confounder domain, 6 studies were evaluated with a serious RoB, indicating that most of the studies adjusted for important confounding factors such as age, sex, smoking, alcohol intake, and education/socioeconomic status. Additionally, 4 studies were evaluated with a serious RoB because of exposure assessment and potential exposure misclassification during follow-up.

### FA biomarkers and incidence of T2D

**FIGURE 2:**
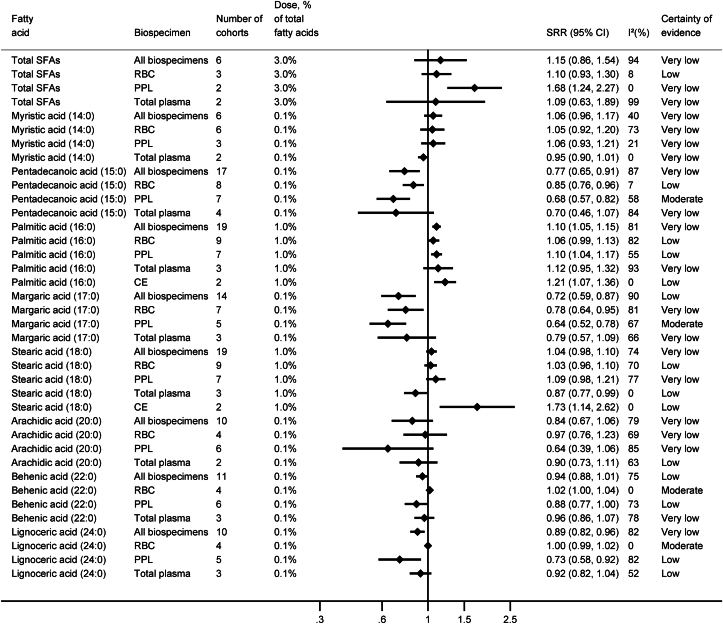
Summary results of linear dose–response meta-analyses for SFAs and risk of type 2 diabetes. CE, cholesteryl ester; CI, confidence interval; PPL, plasma phospholipids; RBC, red blood cells; SRR, summary relative risk.

**FIGURE 3:**
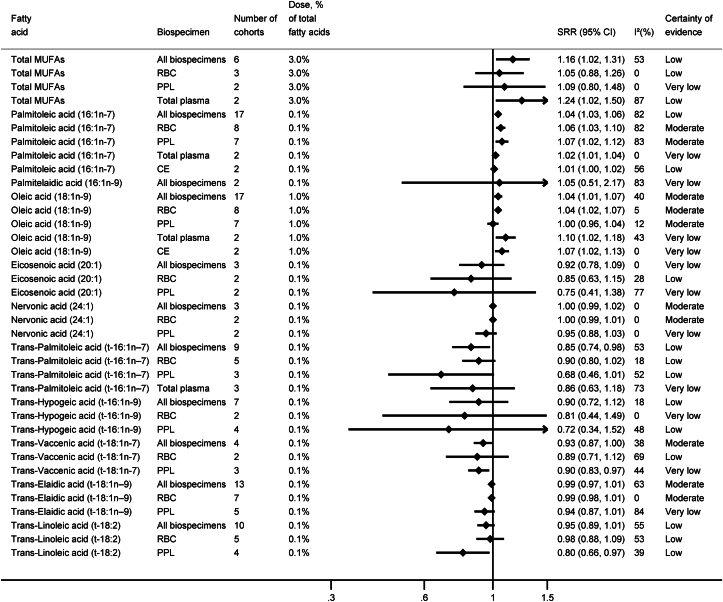
Summary results of linear dose–response meta-analyses for MUFAs and *trans*-fatty acids and risk of type 2 diabetes. CE, cholesteryl ester; CI, confidence interval; PPL, plasma phospholipids; RBC, red blood cells; SRR, summary relative risk.

**FIGURE 4:**
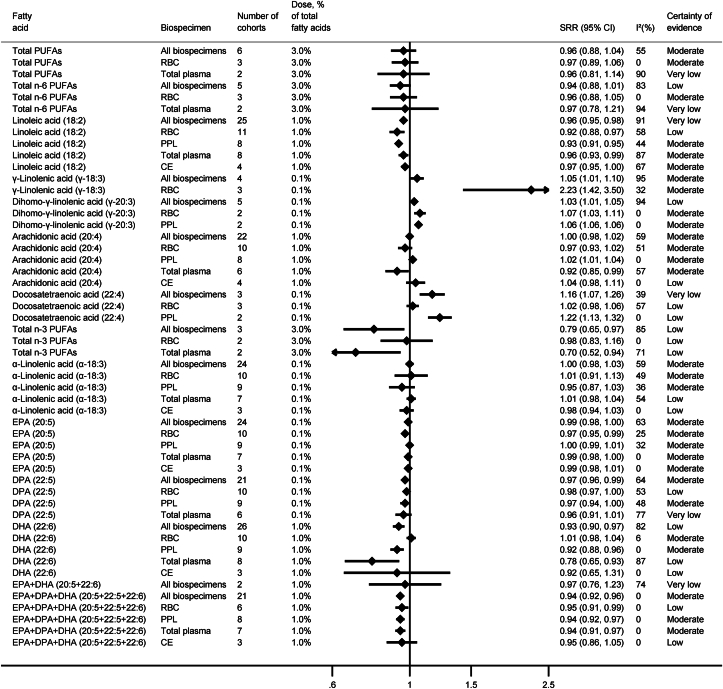
Summary results of linear dose–response meta-analyses for PUFAs and risk of type 2 diabetes. CE, cholesteryl ester; CI, confidence interval; DPA, docosapentaenoic acid; PPL, plasma phospholipids; RBC, red blood cells; SRR, summary relative risk.

We conducted 34 dose–response meta-analyses in all biospecimens combined and 93 biospecimen-specific dose–response meta-analyses on the association between the concentrations of 27 specific FAs and their groups (SFAs, MUFAs, *trans*-FAs, n−6, and n−3 PUFAs) with the risk of T2D (Supplemental Table 7). FIGURE 2, FIGURE 3, FIGURE 4 summarize the results of the meta-analyses for all biospecimens combined and for specific biospecimens. Supplemental Figures 2.1–2.34 present forest plots of each separate meta-analysis. The assessment of the CoE for each meta-analysis is summarized in the supplement (Supplemental Tables 8–11).

### SFAs

The associations for total SFAs pointed to an increased risk of T2D when measured in all biospecimens combined, as well as in specific biospecimens; however, the 95% CI included the null value (Figure 2). The association was strongest when SFAs were measured in PPL (per 3.0% higher concentration: [SRR (95% CI)] 1.68 (1.24, 2.27), *n*_cohorts_ = 2, *I*^2^ = 0%); however, the CoE was rated very low.

The meta-analyses on specific SFAs revealed strongest inverse associations with risk of T2D for pentadecanoic (15:0) and margaric (17:0) acids when measured in PPL [per 0.1% higher concentration: 15:0: 0.68 (0.57, 0.82), *n*_cohorts_ = 7, I^2^ = 58%; and 17:0: 0.64 (0.52, 0.78), *n*_cohorts_ = 5, I^2^ = 67%, both moderate CoE]. On the other hand, the risk of T2D was increased with higher concentrations of 16:0 in the meta-analysis of all biospecimens combined, as well as in PPL, RBC, cholesteryl ester, or total plasma, but the CoE was low to very low.

For the remaining SFAs, the associations were weak or inconsistent, with a CoE rated mainly as low to very low.

### MUFAs and *trans*-FAs

For total MUFAs measured in all biospecimens combined, a positive association with risk of T2D was observed [per 3.0% higher concentration: 1.16 (1.02, 1.31), *n*_cohorts_ = 6, I^2^ = 53%], but the CoE was low (Figure 3). The association persisted in measurements from total plasma but not from RBC and PPL, as the null value was included in the 95% CI (low to very low CoE).

For 16:1n−7, a weak but positive association with risk of T2D was observed in the meta-analysis on all biospecimens combined, with strongest associations observed in meta-analyses on RBC and PPL measurements [per 0.1% higher concentration, RBC: 16:1n−7: 1.06 (1.03, 1.10), *n*_cohorts_ = 8, I^2^ = 82%; PPL 1.07 (1.02, 1.12), *n*_cohorts_ = 7, I^2^ = 83%, both moderate CoE]. For 18:1n−9, the meta-analysis combining all biospecimens and the meta-analysis including only RBC measurements indicated a weak positive association with risk of T2D [per 1% higher concentration, all biospecimens combined: 1.04 (1.01, 1.07), *n*_cohorts_ = 17, I^2^ = 40%; RBC: 1.04 (1.01, 1.06), *n*_cohorts_ = 8, I^2^ = 5%, both moderate CoE]. In contrast, no association was observed for PPL (moderate CoE). There was an indication for an inverse association between higher concentration of *trans-*vaccenic acid (*t*−18:1n−7) in all biospecimens combined and risk of T2D [per 0.1% higher concentration: 0.93 (0.87, 1.00); *n*_cohorts_ = 4, I^2^ = 83%; moderate CoE]. Even stronger association was observed for PPL measurement; however, CoE was rated as very low. Similarly, for eicosenoic acid (20:1), and the *trans*-FAs—*trans*-palmitoleic acid (*t*−16:1n−7), *trans*-hypogeic acid (*t*−16:1n−9), and *trans*-linoleic acid (*t*−18:2)—findings pointed to inverse associations with the risk of T2D when measured in all biospecimens, PPL or RBC (low to very low CoE). For the remaining FAs [palmitelaidic acid (16:1n−9), nervonic acid (24:1), and *trans-*elaidic acid (*t*−18:1n−9)], no associations were observed (moderate to very low CoE).

### PUFAs

Total PUFAs and total n−6 PUFAs pointed to an inverse association with risk of T2D when measured in all biospecimens combined, RBC, or total plasma (moderate to very low CoE). However, the 95% CI included the null value (Figure 4).

For specific n−6 PUFAs, there was a lower risk of T2D for higher 18:2 concentration in all biospecimens combined, but the CoE was low. The inverse association was also observed across specific biospecimens, and the CoE was moderate for the associations in PPL [0.93 (0.91, 0.95), *n*_cohorts_ = 8, I^2^ = 44%) and total plasma [0.96 (0.93, 0.99), *n*_cohorts_ = 8, I^2^ = 87%]. For higher concentrations of γ-linolenic acid (γ−18:3) and di-homo-γ-linolenic acid (γ−20:3), the risk of T2D was increased [γ−18:3 in RBC: 2.23 (1.42, 3.50), *n*_cohorts_ = 3, I^2^ = 32%; γ−20:3 in RBC: 1.07 (1.03, 1.11), *n*_cohorts_ = 2, I^2^ = 0%; γ−20:3 in PPL: 1.06 (1.05, 1.07), *n*_cohorts_ = 2, I^2^ = 0%, all with moderate CoE]. For 20:4, no association with risk of T2D was observed in all biospecimens combined, or when measured in RBC, whereas contrary directions of associations were observed for measurements in PPL and total plasma (moderate CoE). Docosatetraenoic acid (22:4) was positively associated with risk of T2D for all biospecimens combined and PPL, but no associations were observed for RBC, with low to very low CoE.

An inverse association for total n−3 PUFAs was observed for all biospecimens combined and for total plasma, but no association was evident for RBC (all rated as low CoE). For specific n−3 PUFAs, a clear inverse association was observed only for 22:6 in all biospecimens combined [per 0.1% higher concentration: 0.93 (0.90, 0.97), *n*_cohorts_ = 26, I^2^ = 82%), low CoE], with the association confirmed in PPL [per 0.1% higher concentration: 0.92 (0.88, 0.96), *n*_cohorts_ = 9, I^2^ = 0%], but not in RBC (both moderate CoE). An inverse association between the combination of EPA (20:5), docosapentaenoic acid (22:5), and 22:6 and risk of T2D was observed when measured in all biospecimens combined and in biospecimen-specific analyses (RBC, PPL, total plasma, cholesteryl ester), with CoE ranging from low to moderate. However, when 20:5 and 22:5 were considered separately, as well as α−18:3, weak inverse or no associations were observed, regardless of the biospecimen (low to moderate CoE) (Figure 4).

### Subgroups and sensitivity analyses

Geographic location explained some of the heterogeneity: I^2^ decreased to 0% for α−18:3, 22:5, and 22:6 for studies conducted only in the United States, and for 20:5 and 22:6 for studies conducted only in Europe (Supplemental Table 12). Subgroup analyses stratified by geographic location further pointed to a positive association between α−18:3 and an inverse association between 20:4 and risk of T2D in the United States, whereas no precise association was found in Europe. Conversely, an inverse association between 20:5 and a positive association between 22:6 and risk of T2D was found in studies conducted in Europe, whereas no association was found in studies conducted in the United States (Supplemental Table 12). Furthermore, most of the results were robust (Supplemental Table 13), as excluding studies with high RoB from the meta-analyses only weakened the association for 17:0 measured in RBC but did not considerably affect the results. In contrast, a precise positive association was observed between α−18:3 measured in RBC and risk of T2D (Supplemental Table 13). In sensitivity analyses using the leave-one-out approach, the exclusion of any single study did not influence the overall results (Supplemental Figures 3.1–3.68).

### Publication bias and small study effects

We detected asymmetry in the funnel plots for 15:0, 16:0, 22:0, 24:0, 18:2, and 22:6 measured in all biospecimens and 18:2 and 22:5 measured in RBC caused by the lack of small studies with positive associations between these FAs and risk of T2D (Supplemental Figures 4.1, 4.2, 4.6, 4.7, 4.13, and **4.22**). For 16:1n−7, the asymmetry was caused by the lack of small studies pointing to an inverse association between 16:1n−7 and risk of T2D (Supplemental Figure 4.8).

## Discussion

Our systematic review with dose–response meta-analysis provides robust evidence for an association between higher concentrations of certain SFAs (15:0, 17:0), n−3 PUFAs (22:5, 22:5, 22:6), and n−6 PUFA (18:2) with a lower risk of T2D. In contrast, higher levels of MUFAs (16:1n−7, 18:1n−9) as well as n−6 PUFAs (γ−20:3, γ−18:3, and 20:4) were linked to a higher risk of T2D. We showed that concentrations of FAs measured in different biospecimens generally point in the same direction. However, the associations from PPL and RBC measurements were usually stronger, with higher confidence in these findings.

### Comparison with other studies

In contrast to previous studies, we found moderate CoE for an inverse association for 22:5 and the combination of 22:5, 22:5, and 22:6 measured in PPL, and for a positive association for γ−18:3 measured in RBC [[12], [13], [14], [15]]. The differences may stem from the fact that most previous meta-analyses only compared high compared with low FA concentrations and, crucially, combined different biospecimens. Also, in contrast to previous studies, we identified positive associations for 18:1n−9 measured in RBC, and an inverse association for n−3 PUFAs measured in total plasma. We did not confirm associations with *t*−16:1n−7 or α−18:3 regarding the risk of T2D [14]. Our results often pointed to stronger associations in PPL, suggesting that this biospecimen may better reflect long-term dietary intake of specific FAs and provide more accurate insights into metabolic processes linked to risk of T2D. In contrast, prior systematic reviews and meta-analyses tended to prioritize RBC because of its long-term stability, potentially diluting associations when different biospecimens were combined [10].

On the other hand, our results on 15:0, 17:0, 16:0, 16:1n−7, 18:2, and γ−20:3 measured in PPL or RBC, which were rated with low to moderate CoE, are in line with previous studies [13,14,16]. Similarly, we found positive associations for total SFAs measured in PPL, 18:0 measured in cholesteryl esters, and total MUFAs measured in total plasma, as well as an inverse association for *t*−18:1n−7 measured in PPL. However, these findings were graded with low to very low CoE, meaning that future studies may change the results [13,14].

Compared with previous systematic reviews and meta-analyses, our study assessed the CoE, showing that several findings were rated as low or very low CoE, primarily because of imprecision and wide 95% CIs that included the minimal important difference, which raises concerns about clinical relevance. However, although some findings rated with moderate CoE may show weak associations with T2D, it is crucial to emphasize their importance. For example, a 1% higher 18:2 concentration was associated with a 7% lower risk of T2D. Considering that 18:2 levels in PPL average around 20% and dietary interventions can shift this by ≤5% [35], the observed association is likely clinically significant.

### Potential explanations

The stronger associations observed for FAs measured in PPL may reflect the physiological relevance of phospholipids in cell membrane structure and function. Membrane phospholipid saturation influences fluidity, which affects insulin receptor activity and signaling, and thus contributes to the pathology of T2D [38]. Additionally, n−3 PUFAs in phospholipids help stabilize membranes, modulate inflammation, and influence gene expression via receptor interactions [39].

The different associations between specific SFAs and risk of T2D in our study support the view that SFAs are not a uniform group [3]. Higher intakes of dairy products rich in 15:0 and 17:0, such as milk and yogurt, have been linked to a lower risk of T2D [40], and replacing low-fat milk with whole-fat yogurt appears to be beneficial [41]. However, a recent RCT-based study found that substituting dairy SFAs with unsaturated plant-derived FAs was associated with reduced risk of T2D, suggesting a more favorable role for plant-based fats [42]. Nonetheless, dairy SFAs could still offer advantages compared with other animal SFAs. Consistently, our results pointed to a positive association for 16:0, a predominant FA in meat [24]. Experimental studies further suggest that 16:0 and 18:1n−9 may impair insulin secretion by promoting inflammation in pancreatic cells [5]. However, interpretation is complicated by the fact that circulating FAs reflect both dietary intake and endogenous synthesis [24]. Odd-chain FAs can be synthetized endogenously from short-chain FAs produced by the gut microbiome because of higher fiber intake [43]. Similarly, even-chain SFAs like 16:0 and 18:0 are produced through de novo lipogenesis, typically driven by excess intake of sugar or alcohol, and can be desaturated into 16:1n−7 or 18:1n−9, respectively [44].

Although higher intake of plant-based MUFAs, like olive oil, nuts, and seeds, was inversely associated with risk of T2D [3,45,46], animal-based MUFAs were associated with an increased risk [47]. However, circulating MUFAs correlate poorly with dietary intake [24], suggesting that the positive associations observed for 18:1n−9 and 16:1n−7 in our study may be partly driven by endogenous synthesis via de novo lipogenesis.

Although previous studies linked higher intake of *trans-*FAs with a higher risk of T2D [46], we found no association for *t*−18:1n−9. This may be due to *trans-*FA biomarkers capturing both industrial (e.g., from margarine spreads, bakery foods, fried foods) and natural ruminant sources (e.g., dairy foods) [48]. In addition, strong intercorrelations between *trans*-FAs with other FAs exist, and a previous study demonstrated that adjusting for other *trans*-FAs considerably attenuated the association between *t−*16:1n*−*7 and risk of T2D [49]. Similarly, another study showed that adjusting for produced FAs via de novo lipogenesis attenuated the association for 18:2 and the risk of T2D [50]. However, most of the studies in our meta-analysis accounted for such confounding. Additionally, as FAs were measured as percentages of total FAs rather than absolute amounts, an increase in the relative percentage of 1 FA necessarily corresponds to a decrease in the percentage of another FA. This means that the observed associations might reflect a substitution effect, where the changes in 1 FA are indirectly driven by shifts in the levels of other FAs. Future research should consider FA interrelationships and apply substitution models to better isolate independent effects.

Long-chain PUFAs, particularly essential FAs like 18:2 and α−18:3 and their derivatives 20:5 and 22:6, are considered better dietary biomarkers [11,24,51]. However, 18:2 levels are also influenced by genetic variability in FA desaturase 1 and 2, which affects conversion to 20:4 [52]. Increased Δ-6 desaturase activity has been linked to risk of T2D, which is consistent with our observed positive associations for γ−18:3 and γ−20:3 [11]. For α−18:3, no clear inverse association was observed in our study. Previously, we showed a weak J-shaped association between dietary intakes of α−18:3 and risk of T2D [3]. This could explain the null association in the current study, as we only examined linear associations. In addition, α−18:3 rapid oxidation after consumption may explain low correlations with circulating levels [24]. In contrast, 20:5, 22:5, and 22:6 showed inverse associations, likely because of their roles in improving insulin sensitivity and glucose metabolism [17].

### Strengths and limitations

This is the first systematic review with dose–response meta-analysis to comprehensively evaluate the associations between a broad range of FA biomarkers and the risk of T2D, examining both all biospecimens combined and specific biospecimens separately. For the first time, CoE has been assessed for the reported associations, offering an evaluation of the confidence in the findings. The objective exposure measurement in the included studies lowers the risk of misreporting and misclassification bias.

The limitation of this work is that 30% of the studies were rated as serious RoB because of insufficient confounder control (socioeconomic status, education, and alcohol intake), classification of intervention, or deviations from intended intervention. However, our findings did not change after excluding studies with high RoB. As with all observational studies, residual confounding cannot be completely ruled out. In this context, RoB was the most common reason for downgrading the CoE. Additionally, publication bias was detected for 2 meta-analyses and also contributed to lower CoE. Subgroup analyses could not be performed for all FAs because of the low number of included cohorts in biospecimen-specific meta-analyses. Furthermore, the FA concentrations were measured only once at baseline in most studies, and changes during follow-up could not be taken into account. In addition, the investigation of the nonlinearity of dose–response analyses was not feasible because of insufficient information provided from primary studies.

In conclusion, our results summarize the potential of circulating FAs, reflecting both dietary intake and endogenous metabolic processes, in the prevention of T2D. The findings show robust inverse associations between certain SFAs (15:0, 17:0), n−3 PUFAs (20:5, 22:5, 22:6), and n−6 PUFA (18:2) and risk of T2D. Conversely, a higher concentration of specific MUFAs (16:1n−7, 18:1n−9) and certain n−6 PUFAs (γ−20:3, γ−18:3, and 20:4) was associated with a higher risk of T2D. In general, findings from different biospecimens pointed in the same direction but were particularly pronounced in PPL and RBC. From a methodological perspective, our findings emphasize the importance of considering specific biospecimens for FA measurement when conducting meta-analyses.

## Author contributions

The authors' responsibilities were as follows — MN, SS: designed the study question and developed the search term of the systematic review and meta-analysis; ES, MN, NS-M, TS, NI, SS: conducted the systematic literature search; ES, MN, TS, NI, SS: were involved in data acquisition; ES, MN, TS, NI, CB, SS: conducted the assessment of risk of bias; ES: conducted the statistical analyses; ES, MN, LS: rated the certainty of evidence; ES, SS: interpreted the results and drafted the first version of the manuscript; and all authors: critically reviewed and approved the submission of the final manuscript.

## Data availability

Data are available upon reasonable request. All data relevant to the study are included in the article or uploaded as supplementary information. All data for this study were extracted from published meta-analyses, all of which are available and accessible.

## Declaration of generative AI in scientific writing

During the preparation of this work, the author(s) used ChatGPT to improve the language. After using this tool/service, the author(s) reviewed and edited the content as needed and take(s) full responsibility for the content of the publication.

## Funding

The German Diabetes Center is funded by the German Federal Ministry of Health (Berlin, Germany) and the Ministry of Culture and Science of the state North Rhine–Westphalia (Düsseldorf, Germany) and receives additional funding from the German Federal Ministry of Education and Research through the German Center for Diabetes Research (DZD e.V.). The funders had no role in the design, analysis, interpretation, or writing of this article.

## Conflict of interest

LS is an Editor for *Advances in Nutrition* and played no role in the journal's evaluation of the manuscript. The other authors report no conflicts of interest.
