# Supplementary material for: Fatty Acid Biomarkers and Incidence of Type 2 diabetes: A Systematic Review and Dose–Response Meta-analysis of Prospective Observational Studies
Source: Adv Nutr. 2025 Dec 3;17(1):100565. doi: 10.1016/j.advnut.2025.100565 (PMC12796107; doi:10.1016/j.advnut.2025.100565)
Supplement: Multimedia component 1 [file mmc1.docx]

**Online Supplemental Material**

**Fatty acid biomarkers and incidence of type 2 diabetes:**

**a systematic review and dose-response meta-analysis of observational prospective studies**

Edyta Schaefer^1,2^, Manuela Neuenschwander^1,2^, Tim Schiemann^1^, Nadine Iser^1^, Christina Baechle^1,2^, Nafiseh Shokri-Mashhadi^1,2^, Lukas Schwingshackl^3^, Matthias B. Schulze^2,4,5^, Sabrina Schlesinger^1,2^

1 Institute for Biometrics and Epidemiology, German Diabetes Center, Leibniz Center for Diabetes Research at Heinrich Heine University Düsseldorf, Düsseldorf, Germany

2 German Center for Diabetes Research (DZD), Neuherberg, Germany

3 Institute for Evidence in Medicine, Faculty of Medicine and Medical Center - University of Freiburg, Freiburg, Germany

4 Department of Molecular Epidemiology, German Institute of Human Nutrition Potsdam-Rehbruecke, Nuthetal, Germany

5 Institute of Nutritional Science, University of Potsdam, Nuthetal, Germany

**Supplemental Table 1.** Preferred Reporting Items for Systematic reviews and Meta-Analyses (PRISMA) checklist

| **Section and Topic** | **Item #** | **Checklist item** | **Location where item is reported** |
| --- | --- | --- | --- |
| **TITLE** | | |  |
| Title | 1 | Identify the report as a systematic review. | Page 1 |
| **ABSTRACT** | | |  |
| Abstract | 2 | See the PRISMA 2020 for Abstracts checklist. | Page 3, line 90 |
| **INTRODUCTION** | | |  |
| Rationale | 3 | Describe the rationale for the review in the context of existing knowledge. | Lines 133-138 |
| Objectives | 4 | Provide an explicit statement of the objective(s) or question(s) the review addresses. | Lines 139-141 |
| **METHODS** | | |  |
| Eligibility criteria | 5 | Specify the inclusion and exclusion criteria for the review and how studies were grouped for the syntheses. | Lines 150-157 |
| Information sources | 6 | Specify all databases, registers, websites, organisations, reference lists and other sources searched or consulted to identify studies. Specify the date when each source was last searched or consulted. | Lines 158-168 |
| Search strategy | 7 | Present the full search strategies for all databases, registers and websites, including any filters and limits used. | Table S2 |
| Selection process | 8 | Specify the methods used to decide whether a study met the inclusion criteria of the review, including how many reviewers screened each record and each report retrieved, whether they worked independently, and if applicable, details of automation tools used in the process. | Line 162, 165-166 |
| Data collection process | 9 | Specify the methods used to collect data from reports, including how many reviewers collected data from each report, whether they worked independently, any processes for obtaining or confirming data from study investigators, and if applicable, details of automation tools used in the process. | Lines 169-181, 184-196 |
| Data items | 10a | List and define all outcomes for which data were sought. Specify whether all results that were compatible with each outcome domain in each study were sought (e.g. for all measures, time points, analyses), and if not, the methods used to decide which results to collect. | Lines 184-196 |
|  | 10b | List and define all other variables for which data were sought (e.g. participant and intervention characteristics, funding sources). Describe any assumptions made about any missing or unclear information. | Lines 184-196 |
| Study risk of bias assessment | 11 | Specify the methods used to assess risk of bias in the included studies, including details of the tool(s) used, how many reviewers assessed each study and whether they worked independently, and if applicable, details of automation tools used in the process. | Lines 199-207, Table S3 |
| Effect measures | 12 | Specify for each outcome the effect measure(s) (e.g. risk ratio, mean difference) used in the synthesis or presentation of results. | Line 210 |
| Synthesis methods | 13a | Describe the processes used to decide which studies were eligible for each synthesis (e.g. tabulating the study intervention characteristics and comparing against the planned groups for each synthesis (item #5)). | Lines 216-230 |
|  | 13b | Describe any methods required to prepare the data for presentation or synthesis, such as handling of missing summary statistics, or data conversions. | Line 195, 222-226 |
|  | 13c | Describe any methods used to tabulate or visually display results of individual studies and syntheses. | Lines 210-241 |
|  | 13d | Describe any methods used to synthesize results and provide a rationale for the choice(s). If meta-analysis was performed, describe the model(s), method(s) to identify the presence and extent of statistical heterogeneity, and software package(s) used. | Lines 242-246 |
|  | 13e | Describe any methods used to explore possible causes of heterogeneity among study results (e.g. subgroup analysis, meta-regression). | Line 242 |
|  | 13f | Describe any sensitivity analyses conducted to assess robustness of the synthesized results. | Lines 244-246 |
| Reporting bias assessment | 14 | Describe any methods used to assess risk of bias due to missing results in a synthesis (arising from reporting biases). | Line 239 |
| Certainty assessment | 15 | Describe any methods used to assess certainty (or confidence) in the body of evidence for an outcome. | Lines 203-207 |
| **RESULTS** | | |  |
| Study selection | 16a | Describe the results of the search and selection process, from the number of records identified in the search to the number of studies included in the review, ideally using a flow diagram. | Lines 256-260, Figure 1 |
|  | 16b | Cite studies that might appear to meet the inclusion criteria, but which were excluded, and explain why they were excluded. | Table S5 |
| Study characteristics | 17 | Cite each included study and present its characteristics. | Table S6 |
| Risk of bias in studies | 18 | Present assessments of risk of bias for each included study. | Figure S1 |
| Results of individual studies | 19 | For all outcomes, present, for each study: (a) summary statistics for each group (where appropriate) and (b) an effect estimate and its precision (e.g. confidence/credible interval), ideally using structured tables or plots. | Table S6 |
| Results of syntheses | 20a | For each synthesis, briefly summarise the characteristics and risk of bias among contributing studies. | Lines 262-276 |
|  | 20b | Present results of all statistical syntheses conducted. If meta-analysis was done, present for each the summary estimate and its precision (e.g. confidence/credible interval) and measures of statistical heterogeneity. If comparing groups, describe the direction of the effect. | Lines 278-358, Figures 2-4, S2.1-S2.34 |
|  | 20c | Present results of all investigations of possible causes of heterogeneity among study results. | Lines 361-368 |
|  | 20d | Present results of all sensitivity analyses conducted to assess the robustness of the synthesized results. | Lines 369-374 |
| Reporting biases | 21 | Present assessments of risk of bias due to missing results (arising from reporting biases) for each synthesis assessed. | Lines 377-381 |
| Certainty of evidence | 22 | Present assessments of certainty (or confidence) in the body of evidence for each outcome assessed. | Tables S11-S8 |
| **DISCUSSION** | | |  |
| Discussion | 23a | Provide a general interpretation of the results in the context of other evidence. | Lines 394-413 |
|  | 23b | Discuss any limitations of the evidence included in the review. | Lines 489-500 |
|  | 23c | Discuss any limitations of the review processes used. | Lines 184-196 |
|  | 23d | Discuss implications of the results for practice, policy, and future research. | Lines 465-467 |
| **OTHER INFORMATION** | | |  |
| Registration and protocol | 24a | Provide registration information for the review, including register name and registration number, or state that the review was not registered. | Line 91 |
|  | 24b | Indicate where the review protocol can be accessed, or state that a protocol was not prepared. | Line 91 |
|  | 24c | Describe and explain any amendments to information provided at registration or in the protocol. | - |
| Support | 25 | Describe sources of financial or non-financial support for the review, and the role of the funders or sponsors in the review. | Lines 529-535 |
| Competing interests | 26 | Declare any competing interests of review authors. | Line 522 |
| Availability of data, code and other materials | 27 | Report which of the following are publicly available and where they can be found: template data collection forms; data extracted from included studies; data used for all analyses; analytic code; any other materials used in the review. | Line 538-540 |

*From:*  Page MJ, McKenzie JE, Bossuyt PM, Boutron I, Hoffmann TC, Mulrow CD, et al. The PRISMA 2020 statement: an updated guideline for reporting systematic reviews. BMJ 2021;372:n71. doi: 10.1136/bmj.n71. This work is licensed under CC BY 4.0. To view a copy of this license, visit <https://creativecommons.org/licenses/by/4.0/>

**Supplemental Table 2**. Detailed search strategy.

| Data base | Search strategy |
| --- | --- |
| PubMed | (fat OR fats OR fatty OR „fish oil“ OR „fish oils“)  AND  diabetes  AND  (“observational study” OR prospective OR cohort OR cohorts OR longitudinal OR “case-control” OR retrospective OR „follow-up“) |
| Web of Science | Query #1  (((ALL=(fats)) OR ALL=(fatty)) OR ALL=(fat)) OR ALL=("fish oil")  Query #2  ALL=(diabetes)  Query #3  (((((((ALL=("observational study")) OR ALL=(prospective)) OR ALL=(cohort)) OR ALL=(cohorts)) OR ALL=(longitudinal)) OR ALL=("case-control")) OR ALL=(retrospective)) OR ALL=("follow-up")  Query #1 AND Query #2 AND Query #3 |

**Supplemental Table 3**. ROBINS-I (Risk Of Bias In Non-randomised Studies - of Interventions) Tool description and justification of the rating rules.

| **Domain** | **Explanation** | **Judgement** |
| --- | --- | --- |
| **Bias due to confounding** | - Is there potential for confounding of the effect of exposure in this study? - Did the authors use a multivariable-adjusted analysis controlling at least for age, sex, smoking, alcohol intake, education/socioeconomic status? - Did the authors avoid adjusting for post-exposure variables?   Notes: Confounding is expected in all observational studies, low risk of bias was not assigned to any study. Time-varying confounding was expected to be unlikely and is not expected to cause risk of bias in the present study. | Low risk of bias: No bias expected due to confounding, including time-varying confounding.  Moderate risk of bias: Confounding is expected: age, sex, smoking, alcohol intake, education/socioeconomic status, have been appropriately controlled for (in a multivariable-adjusted analysis or through matching);  *or* confounding is expected: age, sex, smoking, alcohol intake have been controlled for (in a multivariable-adjusted analysis or through matching) *and* education/socioeconomic status is not expected to vary substantially within the cohort (e.g., Nurses' Health Study (NHS), Health Professionals Follow-Up Study (HPFS));  *or* authors assessed if confounders affect risk estimates and excluded confounders as covariates if no effect present.  Serious risk of bias: At least one known important confounder was not measured or appropriately controlled for.  No information: No accounting for potential confounders or missing information on which confounders have been controlled for. |
| **Bias due to selection of participants** | - Was participant selection based on characteristics observed after start of the study/exposure assessment? - Do start of follow-up and exposure coincide for most participants? Were methods used that are likely to correct for the presence of selection biases? - Were all samples treated identically (no differential measurement error)   Notes: In observational studies, it is unlikely that post-exposure variables influenced selection of participants into the study. Exclusion of participants may be mostly based on missing data, which will be considered in the domain referring to missing values (see below). Start of follow-up is considered to coincide with the baseline exposure assessment. However, participants are already exposed at start of the study which might have influenced outcome measured that occurred shortly after start of the study. | Low risk of bias: All participants who would have been eligible for the target study were included in the study; and authors performed sensitivity analysis excluding T2D cases that occurred within first 2 follow-up years;  Moderate risk of bias: Selection into the study may have been related to exposure and outcome (e.g., inclusion of postmenopausal women only);  *and* authors used appropriate methods to correct for selection bias; or authors did not perform sensitivity analysis excluding T2D cases that occurred within first 2 follow-up years;  Serious risk of bias: Selection into the study was related to exposure and outcome (e.g., only participants with prediabetes were included in the analysis), *and* this could not be corrected for in the analyses;  *or* start of follow-up and start of exposure do not coincide *and* rate ratio is not constant over time.  No information: No information is reported about selection of participants into the study. |
| **Bias due to exposure assessment** | - Were exposure groups clearly defined and adequately assessed? - Was the information used to define the exposure groups based on reasonable a priori data?   Note: The start of follow-up is considered to coincide with the baseline exposure assessment. The error in exposure measurement within the cohort is expected to be non-differential.  Biomarkers of fatty acids that are not produced endogenously through de novo lipogenesis or enzymatic conversion pathways but rather obtained primarily through dietary intake can reliably represent relative intakes of individuals.  For the measurement of fatty acids in biospecimens, the following aspects should be considered.  1. Source of the biological samples: biological samples vary in the degree to which they reflect long-term dietary intake, which can lead to the misclassification of exposure (6, 7).  2. Fasting state of the individual: fasting state is crucial to reflect habitual dietary intake. If total plasma was used for the measurement but no fasting condition applied, the time since the last meal should be adjusted for (8). Fasting state is not unequivocal if fatty acids measured in red blood cells or adipose tissue (9). n–3 PUFA status can be accurately assessed in postprandial samples in whole blood, plasma, or red blood cells (10).  3. Processing: samples with a longer lag time from blood draw to freezing can be less reliable, samples not kept chilled throughout can be less reliable.  4. Storage of the sample: suitably low temperatures, storage period adapted to the bio-sample, use of antioxidants. Red blood cells require more conservative storage (9).  5. Measurement methods: different analytical techniques are available and a validated method should be applied. Evidence of external validation should be available (11). | Low risk of bias: Exposure status was well defined (comprehensibly derived categories);  and no differences between cases and controls in the exposure assessment (no differential measurement error expected), all samples were treated identically;  *and* no significant measurement error is expected; adipose tissue, red blood cells, whole blood, total plasma, total serum or plasma phospholipids were used as the biological source;  *and* fasting samples were used (exception time since the last meal has been adjusted for, red blood cells or adipose tissue used for measurement, or n-3 PUFA measured in whole blood or plasma.)  *and* measurement was performed following validated method GC or HPLC combined with FID or MS, TLC, electrospray via liquid chromatography MS/MS, NMR, NIR, shotgun methods;  *and* tubes stored by at least -70°C to -80°C or, except for red blood cells, at -20°C for <3 years, or in the case of red blood cells at -20°C for a period of <6 months with sufficient use of antioxidants;  *and* CVs reported (precision of concentration measurements is acceptable at the <30% CV) or any control for measurement errors (e.g., control for batch effects, duplicate measurements, or calibration).  Moderate risk of bias: Exposure status was well defined (comprehensibly derived categories);  no differences between cases and controls in the exposure assessment, all samples were treated identically; *and* plasma cholesteryl ester, free fatty acids or triglycerides used for the measurement of fatty acids concentration;  *and* at least 2/3 of the samples were fasting;  *and* measurement was performed following validated method GC or HPLC combined with FID or MS, TLC, electrospray via liquid chromatography MS/MS, NMR, NIR, shotgun methods;  *and* tubes stored by at least -70°C to -80°C;  *and* CVs reported (precision of concentration measurements is acceptable at the <30% CV) or any control for measurement errors (e.g., control for batch effects, duplicate measurements, or calibration).  Serious risk of bias: Exposure status was not well defined;  and/or differences between cases and controls in the exposure assessment, samples were not treated equally;  *and/or* non-fasting samples were used *and* it has not been adjusted for the time since the last meal;  *and/or* fatty acids were measured using not validated tools;  *and*/*or* tubes stored by the temperature higher than −80°C for a period >3 years without the use of antioxidants preserving from oxidation, or red blood cells stored at -20°C for a period >6 months without sufficient use of antioxidants;  *and/or* incorrect sample processing between sampling and freezing, e.g., lack of cooling or a too long time between sampling and freezing (>2days);  *and/or* CVs not reported *or* to large or no control for measurement errors or significant measurement errors are present.  No information: No definition of exposure or no explanation of the source of information about exposure status is reported. |
| **Bias due to misclassification during follow-up** | - Were there deviations from the exposure beyond what would be expected in usual practice? - Were these deviations unbalanced between groups and likely to have affected the outcome?   Notes:  Repeated measurements of the exposure are mostly not available in observational studies.  Changes in fatty acid concentrations over time are likely to be influenced by a combination of biological factors such as aging and dietary intake. However, significant changes in diet in healthy participants which may lead to fluctuations in fatty acid concentrations are not expected. Changes in diet may be similar between studies and may also be similar between groups (differential misclassification is not expected). Recent studies have shown that diet is constant or changes only slightly over time (12, 13).  Evidence is available that small changes in marine and plant n-3 PUFAs (increase) and *trans*-fatty acids (decrease) were observed over 13 years but changes in odd-chain, even-chain, very-long-chain SFAs, MUFA, and n-6 PUFA were negligible (14).  It is unlikely that changes in circulating fatty acids concentrations resulting from potential slight changes in dietary patterns will distort T2D risk assessment. Thus, if repeated measures are not available, moderate risk of bias could be assigned to a study. | Low risk of bias: Repeated measurements of the exposure status during follow-up are available;  *and* no or only slight changes in fatty acids concentrations were observed and the changes were considered in the analysis;  *and* assessment of the exposure can be rated as low risk of bias (compare domain: Bias due to exposure assessment).  Moderate risk of bias: Repeated measurements of the exposure are not available, but high variation is not expected during follow-up (compare notes);  *or* repeated measurements of the exposure status during follow-up are available and some changes in the fatty acids’ concentrations were observed. Analysis was appropriate to estimate the effect of changes, allowing for deviations that were likely to impact the outcome;  *and* assessment of the exposure can be rated at least as moderate risk of bias (compare domain: Bias due to exposure assessment).  Serious risk of bias: Exposure status is measured during follow-up and high changes have been observed, and the analysis was not appropriate to estimate the effect of changes, allowing for deviations that were likely to impact the outcome;  *or* repeated measurements of the exposure status during follow-up are available and changes in the fatty acids’ concentrations were observed. Analysis was appropriate to estimate the effect of changes, allowing for deviations that were likely to impact the outcome;  *and* assessment of the exposure can be rated as high risk of bias (compare domain: Bias due to exposure assessment).  No information: No information on deviations from the exposure is reported. |
| **Bias due to missing data** | - Were there missing outcome data? - Were participants excluded due to missing data on exposure status? - Were participants excluded due to missing data on other variables needed for analysis?   Notes: Missing data on exposure variables and other variables are expected to be missing at random and not related to exposure or outcome that have been assessed during follow-up. | Low risk of bias: Little loss-to-follow-up (<20%) and data on exposure and other variables were reasonably complete (<10% missing data) and was unlikely to introduce bias;  *or* analysis addressed missing data and is likely to have reduced risk of bias.  Moderate risk of bias: There is a proportion of missing data in the original cohort or a high proportion of loss-to-follow-up;  *and* analysis is unlikely to have removed the risk of bias arising from the missing data (e.g., using logistic regression).  Serious risk of bias: High proportions (>50%) of missing data;  *and* analysis is unlikely to have removed the risk of bias arising from the missing data;  *or* missing data were addressed inappropriately in the analysis;  *or* nature of the missing data means that the risk of bias cannot be removed through appropriate analysis.  No information: No information is reported about missing data or the potential for data to be missing. |
| **Bias due to measurement of the outcome** | - Could the outcome measure have been influenced by knowledge of the exposure status? - Were the methods of outcome assessment comparable across exposure groups? - Was any systematic error in measurement of the outcome related to exposure status?   Notes: In observational studies, it is not expected that outcome assessors were aware of exposure status of the participants. | Low risk of bias: The methods of outcome assessment were comparable across exposure groups;  *and* the outcome measure was unlikely to be influenced by knowledge of the exposure status of study participants;  *and* any error in measuring the outcome is unrelated to exposure status (i.e., objective measures such as confirmed medical records, record linkage).  Moderate risk of bias: The methods of outcome assessment were comparable across exposure groups;  *and* any error in measuring the outcome may be minimally related to exposure status;  *or* if the outcome measure was not reliable measured (i.e., self-reports were not confirmed for the whole study population).  Serious risk of bias: The methods of outcome assessment were not comparable across exposure groups;  *or* the outcome measure was subjective (i.e., self-report of T2D by study participants);  *and* error in measuring the outcome was related to exposure status.  No information: No information is reported about the methods of outcome assessment. |
| **Bias due to selective reporting of the results** | - Is the reported effect estimate likely to be selected from multiple analyses of the exposure-outcome relationship? - Is the reported effect estimate likely to be selected from different subgroups?   Notes: In observational studies, it is unusual to publish an a priori analysis plan or protocol. Multiple outcome measurements for the definition of T2D are not expected. | Low risk of bias: There is a clear description of all analyses and the analyses are consistent and all reported results correspond to all intended outcomes, analyses, and sub-cohorts.  Moderate risk of bias: Analyses are clearly defined;  *and* there is indication of selection of the reported analysis from among multiple analyses;  *and* there is indication of selection of cohort or subgroups for analysis and reporting on basis of the results (e.g., estimates not shown for all analyses).  Serious risk of bias: There is a high risk of selective reporting from among multiple analyses;  *or* the cohort or subgroup is selected from a larger study for analysis and appears to be reported based on the results.  No information: There is too little information to make a judgement. |
| **Overall judgement** | Low risk of bias | The study is judged to be at low risk of bias for all domains. |
|  | Moderate risk of bias | The study is judged to be at low or moderate risk of bias for all domains. |
|  | Serious risk of bias | The study is judged to be at serious risk of bias in at least one domain, but not at critical risk in any domain. |
|  | No information | There is no clear indication that the study is at serious or critical risk of bias and there is a lack of information in one or more key domains of bias. |
| CVs, coefficient of variation; FID, Flame Ionization Detector; GC, gas chromatography; HPLC, High-Performance Liquid Chromatography; MS, Mass Spectrometry; MUFA, monounsaturated fatty acids; NIR, Near-Infrared Spectroscopy; NMR, Nuclear Magnetic Resonance; PUFA, polyunsaturated fatty acids; SFA, saturated fatty acids; T2D, type 2 diabetes; TLC, Thin-Layer Chromatography | | |

| **Supplemental Table 4.** Grades of Recommendations, Assessment, Development, and Evaluation (GRADE) assessment algorithm (15). |
| --- |
| Five domains can downgrade the CoE: RoB of primary studies, imprecision, inconsistency, indirectness, and publication bias. In case of meta-analyses of PCS, the initial CoE was downgraded by two levels unless the study design reduced confounding, selection and information bias, as evaluated by a tool for assessing RoB in non-randomized studies of interventions (ROBINS-I). Additionally, the CoE can be upgraded for meta-analyses of PCS due to a large magnitude of association (e.g., risk ratio (RR) <0.5 or >2) or due to a dose–response gradient.  Detailed description of the CoE assessment in each of the domains:   1. **Study design:** Since we applied ROBINS-I tool for RoB assessment; all meta-analyses will start at high CoE. For this the box “ROBINS-I” in the GRADEpro online tool needs to be checked. 2. **Risk of Bias:**  - not serious: no studies will be rated as not serious, since maximum RoB score is moderate - serious: if not more than 50% rated as serious RoB - very serious: if maximally 50% (based on weights in meta-analysis) of the studies high RoB  1. **Inconsistency:** In order to assess the inconsistency, we evaluate the forest plots. I² and p-value for statistical heterogeneity should be used as supportive information.  - If estimates pointed to same directions and 95% CIs overlap -> not serious - If estimates pointed to same directions but were appreciably different and 95% CIs did not overlap -> serious - If estimates pointed to different directions and 95% CIs only slightly overlapped -> serious - If estimates pointed to different directions, 95% CIs mostly overlap but I^2^ >90% -> serious - If estimates pointed to different directions and 95% CI did not overlap -> very serious  1. **Indirectness:** In general, we rarely downgraded because of indirectness, since all included studies fulfilled strict inclusion criteria. 2. **Imprecision:** if n cases and n participants are given, absolute effects will be calculated and applied for imprecision assessment. MID was set at 5 cases per 1000; If CI cross the MID -> serious  - Null value excluded in 95% CI AND CI did not include MID, cases >400/n >800 -> not serious - Null value excluded in 95% CI AND CI did not include MID, cases <400/n <800 -> serious - Null value excluded in 95% CI AND CI included MID, cases >400/ n>800 -> serious - Null value excluded in 95% CI AND CI included MID, cases <400/ n<800 -> very serious - Null value included in 95% CI BUT CI did not include MID, cases >400/ n>800 -> not serious - Null value included in 95% CI BUT CI did not include MID, cases <400/ n<800 -> serious - Null value included in 95% CI AND CI included MID, cases >400/ n>800 -> serious - Null value included in 95% CI AND CI included MID, cases <400/ n<800 -> very serious - Extreme values included in 95% CI -> extremely serious  1. **Other considerations:**   *****If only one domain was downgraded by one level but an upgrade happened in “other considerations”, e.g., due to dose-response association, the CoE is still moderate -> the overall assessment must be changed per Hand and marked with a *****  **Strong association:**   - RR <0.5 and/or >2.0 -> upgrade for one level - RR <0.2 and/or >5 -> upgrade for two levels   **Publication bias:** if at least 10 studies are available; check funnel plots + Eggers test   - Not applicable (<10 studies) for publication bias -> not serious - No, ≥10 studies and no publication bias was detected -> not serious - Yes, ≥10 studies and publication bias was detected -> serious |

CI, confidence interval; CoE, certainty of evidence; MID, minimal important difference; PCS, prospective cohort studies; RoB, risk of bias; RR, relative ri

**Supplemental Table 5**. List of studies excluded after full text analysis with reasons.

| Exclusion reasons (number of studies) | References |
| --- | --- |
| Abstract (n=14) | (16-29) |
| Duplicate cohort (n=6) | (30-35) |
| Insufficient data for dose-response analysis calculation (n=18) | (36-53) |
| No risk estimates or confidence interval reported (n=4) | (54-57) |
| Not relevant data (n=49) | (58-106) |
| Not relevant exposure (n=144) | (107-250) |
| Wrong outcome (n=35) | (251-285) |
| Wrong study design (n=29) | (286-314) |
| Wrong unit of measurement (n=5) | (4, 5, 315-317) |
| Included studies (n=27) | (1-3, 196, 318-340) |

| **Supplemental Table 6.** Characteristics table of included studies. | | | | | | | | | | |
| --- | --- | --- | --- | --- | --- | --- | --- | --- | --- | --- |
| **Study (author, year, location, cohort)** | | **Follow-up** | **Exposure assessment** | **Number of participants (cases), sex, age** | **T2D assessment** | **Exposure** | **% of total fatty acids** † | **RR (95% CI), HR [95% CI], OR (95% CI)** †† | **Adjustment factors** | **Risk of Bias** |
| Alhazmi, 2014, Australia, HCS (318) | | 5 years | Whole blood, GC | 187 (37), m/f, 70 years  **3322 (NA)* | A self-administered questionnaire was used to ascertain all HCS 5-year incident cases of T2D. | Linoleic acid (18:2) | Per 2,85 % | 1,00 (1,00; 1,01) | Age, sex, BMI, physical activity, alcohol intake, smoking, supplement use, carbohydrate, fiber, protein | High |
|  |  |  |  |  |  | Dihomo-ɣ-Linolenic acid (ɣ-20:3) | Per 0,6 % | 1,04 (1,01; 1,07) |  |  |
|  |  |  |  |  |  | Arachidonic acid (20:4) | Per 2,1 % | 1,01 (1,00; 1,01) |  |  |
|  |  |  |  |  |  | α-Linolenic acid (α-18:3) | Per 0,3 % | 1,10 (1,03; 1,18) |  |  |
|  |  |  |  |  |  | Eicosapentaenoic acid (20:5) | Per 1,2 % | 1,05 (1,02; 1,08) |  |  |
|  |  |  |  |  |  | Docosahexaenoic acid (22:6) | Per 1,58 % | 1,03 (1,02; 1,05) |  |  |
|  |  |  |  |  |  | Docosapentaenoic acid (22:5) | Per 0,29 % | 0,99 (0,97; 1,02) |  |  |
| Bragg, 2022, China, CKB (1) | | 8,6 years | Total plasma, high-throughput targeted NMR-metabolomics platform | 1645 (882), m/f, 54 years  **432763 (9033)* | Incident T2D cases were recorded during follow-up until 1 January 2017 based on ICD10 E11. | Total SFA | Per 7,7 % | 0,62 [0,53; 0,73] | Age, sex, study area, education, fasting time, smoking, alcohol, physical activity, dietary factors, family history  of diabetes, BMI and waist circumference | Moderate |
|  |  |  |  |  |  | Total MUFA | Per 6,6 % | 1,30 [1,09; 1,54] |  |  |
|  |  |  |  |  |  | Total PUFA | Per 6,5 % | 1,10 [0,95; 1,28] |  |  |
|  |  |  |  |  |  | Total n-3 PUFA | Per 1,8 % | 0,72 [0,60; 0,87] |  |  |
|  |  |  |  |  |  | Docosahexaenoic acid (22:6) | Per 0,7 % | 0,46 [0,38; 0,55] |  |  |
|  |  |  |  |  |  | Total n-6 PUFA | Per 5,5 % | 1,17 [1,01; 1,36] |  |  |
|  |  |  |  |  |  | Linoleic acid (18:2) | Per 5,4 % | 1,23 [1,06; 1,44] |  |  |
| Chiava-Blanch, 2022, Spain, Di@betes Study (319) | | 7,5 years | RBC, GC | 1035 (131), m/f, 49,4 years | T2D was defined as hypoglycaemic treatment at the final visit, FBG ≥126 mg/dL, HbA1c ≥ 6.5%, and/or blood glucose ≥200 mg/dL after 2-h of oral overload. | EPA + DHA | Per 1,7 % | 1,33 \|0,99; 1,78\| | Age, sex, first-degree family history of diabetes, BMI, and glucose metabolism category, hypertension, physical activity, central obesity,  and educational level | High |
|  |  |  |  |  |  | Linoleic acid (18:2) | Per 2,19 % | 0,85 \|0,61; 1,19\| |  |  |
|  |  |  |  |  |  | α-Linolenic acid (α-18:3) | Per 0,09 % | 0,48 \|0,27; 0,84\| |  |  |
|  |  |  |  |  |  | Eicosapentaenoic acid (20:5) | Per 0,34 % | 0,78 \|0,55; 1,09\| |  |  |
|  |  |  |  |  |  | Docosapentaenoic acid (22:5) | Per 1,39 % | 1,26 [1,02; 1,56] |  |  |
|  |  |  |  |  |  | Docosahexaenoic acid (22:6) | Per 1,39 % | 1,48 \|1,12; 1,96\| |  |  |
| Djousse, 2011, USA, CHS (320) | | 10,6 years | PPL, GC | 3088 (204), m/f, 81,5 years | Incident T2D was ascertained if any of the following conditions were present: 1) new use of insulin or oral hypoglycemic agents, 2) a FBG concentration ≥126 mg/dL, or 3) a non-FBG concentration ≥200 mg/dL. | EPA + DHA | 2,52 %  3,12 %  3,82 %  4,62 % | 1,00  0,96 (0,65; 1,43)  1,03 (0,69; 1,54)  0,64 (0,41; 1,01) | Age, race, sex, clinic site, BMI, alcohol consumption, physical activity, current smoking, PPL Linoleic acid (18:2), and LDL cholesterol. | High |
|  |  |  |  |  |  | α-Linolenic acid (α-18:3) | 0,10 %  0,13 %  0,16 %  0,20 % | 1,00  0,93 (0,65; 1,34)  0,99 (0,68; 1,44)  0,57 (0,36; 0,90) |  |  |
| Forouhi, 2016, Europe, EPIC-InterAct (2) | | 9,8 years | PPL, GC | 27296 (12132), m/f, 52,3 years  **340234 (12403)* | Incident T2D was ascertained by reviewing multiple sources of evidence: self-report, linkage to primary-care registers and secondary care registers, medication use, hospital admissions, and mortality data. No diabetes cases were ascertained solely by self-report. | Total n-3 PUFA | 4,46 %  5,56 %  6,43 %  7,45 %  9,75% | 1,00  1,00 [0,87; 1,15]  0,96 [0,84; 1,11]  0,96 [0,81; 1,13]  0,90 [0,79; 1,03] | Age, centre, sex, physical activity, smoking, education level, BMI, total energy intake, alcohol, intake of meat, fruit and vegetables, soft drinks, dairy, fish and shellfish, nuts and seeds, vegetable oil, olive oil, margarine | Moderate |
|  |  |  |  |  |  | Total n-6 PUFA | 31,70 %  34,48 %  36,01 %  37,49 %  39,89 % | 1,00  0;94 [0,85; 1,03]  0,85 [0,76; 0,94]  0,84 [0,75; 0,94]  0,74 [0,64; 0,85] |  |  |
|  |  |  |  |  |  | Linoleic acid (18:2) | 18,23 %  20,94 %  22,56 %  24,23 %  27,09 % | 1,00  0,83 [0,75; 0,91]  0,72 [0,64; 0,80]  0,64 [0,57; 0,73]  0,54 [0,47; 0,61] |  |  |
|  |  |  |  |  |  | γ-Linolenic acid (γ-18:3) | 0,03 %  0,06 %  0,07 %  0,10 %  0,15 % | 1,00  1,19 [1,06; 1,33]  1,49 [1,31; 1,70]  1,69 [1,39; 2,04]  2,04 [1,73; 2,39] |  |  |
|  |  |  |  |  |  | Dihomo-ɣ-Linolenic acid (ɣ-20:3) | 2,13 %  2,69 %  3,08 %  3,50 %  4,28 % | 1,00  1,40 [1,15; 1,72]  1,79 [1,48; 2,15]  2,34 [1,91; 2,88]  3,26 [2,44; 4,36] |  |  |
|  |  |  |  |  |  | Arachidonic acid (20:4) | 6,85 %  8,20 %  9,12 %  10,10 %  11,91 % | 1,00  0,93 [0,83; 1,05]  1,10 [0,98; 1,22]  1,04 [0,93; 1,16]  1,03 [0,91; 1,17] |  |  |
|  |  |  |  |  |  | Docosatetraenoic acid (22:4) | 0,19 %  0,24 %  0,28 %  0,31 %  0,39 % | 1,00  1,06 [0,92; 1,22]  1,16 [1,04; 1,29]  1,27 [1,04; 1,55]  1,49 [1,24; 1,79] |  |  |
| Fretts, 2019 (321) | Iceland, AGESR | 9 years | PPL, GC | 753 (28), m/f, 75,5 years | Most participating cohorts, incident T2D was defined based on ≥1 criterion: FBG concentrations ≥126 mg/dL, Non-fasting or 2-h OGTT ≥200 mg/dL, HbA1c ≥6.5%, use of insulin or oral  Hypoglycemic medications, or self-report. For 3 European studies (EPIC-InterAct, KIHD, and METSIM), diabetes was ascertained by linkage to registries of primary care, secondary care, medication use, hospital admissions, or mortality. | Arachidic acid (20:0) | Per 0,10 % | 0,36 (0,12; 1,11) | Age, sex, clinic, race, education, physical activity, smoking, alcohol use, prevalent hypertension, prevalent dyslipidaemia, prevalent coronary heart disease, self-reported health status, BMI, waist circumference. | Moderate |
|  |  |  |  |  |  | Behenic acid (22:0) | Per 0,27 % | 0,42 (0,15; 1,17) |  |  |
|  |  |  |  |  |  | Lignoceric acid (24:0) | Per 0,22 % | 0,34 (0,13; 0,93) |  |  |
|  | Taiwan, CCCC | 8 years | Total plasma, GC | 616 (128), m/f, 58,7 years |  | Arachidic acid (20:0) | Per 0,29 % | 0,97 (0,62; 1,51) |  |  |
|  |  |  |  |  |  | Behenic acid (22:0) | Per 0,31 % | 1,01 (0,84; 1,21) |  |  |
|  |  |  |  |  |  | Lignoceric acid (24:0) | Per 0,33 % | 0,72 (0,43; 1,20) |  |  |
|  | Europe, EPIC-InterAct, | 14 years | PPL, GC | 27296 (12132), m/f, 52,3 years  **340234 (12403)* |  | Arachidic acid (20:0) | Per 0,04 % | 0,55 (0,44; 0,69) |  |  |
|  |  |  |  |  |  | Behenic acid (22:0) | Per 0,08 % | 0,68 (0,58; 0,80) |  |  |
|  |  |  |  |  |  | Lignoceric acid (24:0) | Per 0,07 % | 0,61 (0,52; 0,72) |  |  |
|  | USA, FHS | 10 years | RBC, GC | 1870 (95), m/f, 64,4 years |  | Lignoceric acid (24:0) | Per 0,16 % | 0,73 (0,43; 1,24) |  |  |
|  | Finland, KIHD | 12 years | Total plasma, GC | 1543 (205), m/f, 62,7 years |  | Behenic acid (22:0) | Per 0,09 % | 0,53 (0,35; 0,79) |  |  |
|  |  |  |  |  |  | Lignoceric acid (24:0) | Per 0,12 % | 0,68 (0,46; 0,99) |  |  |
|  | Australia, MCCS | 10 years | PPL, GC | 5617 (485), m/f, 56,3 years  **21523 (929)* |  | Arachidic acid (20:0) | Per 0,07 % | 0,93 (0,75; 1,15) |  |  |
|  |  |  |  |  |  | Behenic acid (22:0) | Per 0,17 % | 0,95 (0,76; 1,19) |  |  |
|  |  |  |  |  |  | Lignoceric acid (24:0) | Per 0,15 % | 0,82 (0,65; 1,04) |  |  |
|  | USA, MESA | 12 years | PPL, GC | 2252 (309), m/f, 60,9 years |  | Arachidic acid (20:0) | Per 0,09 % | 1,01 (0,58; 1,75) |  |  |
|  |  |  |  |  |  | Behenic acid (22:0) | Per 0,29 % | 0,92 (0,51; 1,65) |  |  |
|  | Finland, METSIM | 8 years | PPL, GC | 1302 (71), m, 55 years |  | Arachidic acid (20:0) | Per 0,07 % | 1,23 (0,93; 1,62) |  |  |
|  |  |  |  |  |  | Behenic acid (22:0) | Per 0,16 % | 1,02 (0,75; 1,39) |  |  |
|  |  |  |  |  |  | Lignoceric acid (24:0) | Per 0,14 % | 0,77 (0,39; 1,51) |  |  |
|  | USA, WHIMS | 14 years | RBC, GC | 6510 (502), f, 70,1 years |  | Arachidic acid (20:0) | Per 0,06 % | 0,93 (0,69; 1,26) |  |  |
|  |  |  |  |  |  | Behenic acid (22:0) | Per 0,10 % | 1,05 (0,82; 1,35) |  |  |
|  |  |  |  |  |  | Lignoceric acid (24:0) | Per 0,24 % | 1,01 (0,84; 1,22) |  |  |
|  | USA, CHS | 18 years | PPL, GC | 3107 (282), m/f, 75,1 years |  | Arachidic acid (20:0) | Per 0,08 % | 0,59 (0,43; 0,80) |  |  |
|  |  |  |  |  |  | Behenic acid (22:0) | Per 0,32 % | 0,77 (0,56; 1,04) |  |  |
|  |  |  |  |  |  | Lignoceric acid (24:0) | Per 0,28 % | 0,72 (0,52; 0,99) |  |  |
|  | France, 3C Study | 13 years | RBC, Plasma, GC | 565 (39), m/f, 76 years |  | Arachidic acid (20:0) | Per 0,08 % | 0,75 (0,35; 1,63) |  |  |
|  |  |  |  |  |  | Behenic acid (22:0) | Per 0,30 % | 0,78 (0,35; 1,76) |  |  |
| Imamura, 2018, USA, Iceland, FORCE (322) | USA, CHS | 10,6 years | PPL, GC | 3179 (282), m/f, 75,1 years | Most participating cohorts, incident T2D was defined based on ≥1 criterion: FBG ≥126 mg/dL; 2-hour OGTT ≥200 mg/dL; new use of insulin or oral hypoglycaemic medication assessed by participant reports, medication inventories or registries; and fasting or non-fasting HbA1C concentration ≥6.5%. In the MCCS and the AOC, incident T2D was defined by self-reported physician diagnosis, use of antidiabetic medication, or both. | Pentadecanoic acid (15:0) | 0,09 % | 0,75 (0,55; 1,01) | Age, sex, field site, race, socioeconomic status, smoking status, physical activity, alcohol, family history of diabetes, dyslipidaemia, hypertension, menopausal status, prevalent coronary heart disease, BMI, waist circumference | Moderate |
|  |  |  |  |  |  | Margaric acid (17:0) | 0,16 % | 0,49 (0,35; 0,69) |  |  |
|  |  |  |  |  |  | *Trans-*Palmitoleic acid (*t-*16:1n–7) | 0,12 % | 0,57 (0,40; 0,79) |  |  |
|  | USA, MESA | 9,3 years | PPL, GC | 2252 (309), m/f, 61 years |  | Pentadecanoic acid (15:0) | 0,13 % | 0,48 (0,25; 0,95) |  |  |
|  | USA, FHS | 5,8 years | RBC, GC | 2209 (98), m/f, 64,4 years |  | Pentadecanoic acid (15:0) | 0,10 % | 0,90 (0,50; 1,63) |  |  |
|  |  |  |  |  |  | Margaric acid (17:0) | 0,14 % | 0,69 (0,34; 1,42) |  |  |
|  |  |  |  |  |  | *Trans-*Palmitoleic acid (*t-*16:1n–7) | 0,12 % | 0,83 (0,47; 1,45) |  |  |
|  | USA, WHIMS | 11 years | RBC, GC | 6510 (502), f, 70,1 years |  | Pentadecanoic acid (15:0)) | 0,15 % | 0,72 (0,56; 0,94) |  |  |
|  |  |  |  |  |  | Margaric acid (17:0) | 0,38 % | 0,84 (0,64; 1,11) |  |  |
|  |  |  |  |  |  | *Trans-*Palmitoleic acid (*t*-16:1n–7) | 0,32 % | 0,83 (0,59; 1,16) |  |  |
|  | USA, IRAS | 5,3 years | Total plasma, GC | 719 (146), m/f, 55,1 years |  | Pentadecanoic acid (15:0) | 0,13 % | 0,55 (0,33; 0,90) |  |  |
|  |  |  |  |  |  | *Trans-*Palmitoleic acid (*t*-16:1n–7) | 0,26 % | 0,94 (0,56; 1,58) |  |  |
|  | USA, HPFS | 11,1 years | RBC, GC | 1519 (112), m, 64,1 years |  | Pentadecanoic acid (15:0) | 0,11 % | 1,14 (0,82; 1,58) |  |  |
|  |  |  |  |  |  | Margaric acid (17:0) | 0,22 % | 1,26 (0,88; 1,81) |  |  |
|  |  |  |  |  |  | *Trans-*Palmitoleic acid (*t*-16:1n–7) | 0,10 % | 1,07 (0,74; 1,55) |  |  |
|  | Europe, InterAct | 12,3 years | PPL, GC | 27296 (12132), m/f, 52,3 years  **340234 (12403)* |  | Pentadecanoic acid (15:0) | 0,15 % | 0,52 (0,44; 0,60) |  |  |
|  |  |  |  |  |  | Margaric acid (17:0) | 0,20 % | 0,26 (0,21; 0,32) |  |  |
|  | France, 3C Study | 8 years | RBC, GC | 565 (39), m/f, 76,0 years |  | Pentadecanoic acid (15:0) | 0,11 % | 0,56 (0,20; 1,51) |  |  |
|  |  |  |  |  |  | Margaric acid (17:0) | 0,15 % | 0,21 (0,08; 0,56) |  |  |
|  | Netherlands, AOC | 2,5 years | PPL, GC | 760 (37), m/f, 68,9 years |  | Pentadecanoic acid (15:0) | 0,08 % | 0,85 (0,53; 1,36) |  |  |
|  |  |  |  |  |  | Margaric acid (17:0) | 0,19 % | 0,45 (0,16; 1,23) |  |  |
|  | Sweden, ULSAM | 21,4 years | AT, GC | 2009 (396), m, 54,4 years |  | Pentadecanoic acid (15:0) | 0,18 % | 1,02 (0,54; 1,95) |  |  |
|  |  |  |  |  |  | Margaric acid (17:0) | 0,12 % | 1,05 (0,53; 2,09) |  |  |
|  | Sweden, PIVUS | 10 years | PPL, CE, GC | 879 (67), m/f, 72,5 years |  | Pentadecanoic acid (15:0) | 0,21 % | 0,83 (0,48; 1,43) |  |  |
|  |  |  |  |  |  | Margaric acid (17:0) | 0,15 % | 0,69 (0,44; 1,09) |  |  |
|  | Finland, METSIM | 5,5 years | PPL, GC | 1302 (71), m, 57,3 years |  | Pentadecanoic acid (15:0) | 0,12 % | 0,62 (0,26; 1,48) |  |  |
|  |  |  |  |  |  | Margaric acid (17:0) | 0,19 % | 0,55 (0,29; 1,03) |  |  |
|  | Australia, MCCS | 4 years | PPL, GC | 6151 (490), m/f, 56,3 years  **21523 (929)* |  | Pentadecanoic acid (15:0) | 0,12 % | 0,44 (0,33; 0,58) |  |  |
|  | Taiwan, CCCC | 6 years | Total plasma, GC | 1838 (128), m/f, 58,7 years |  | Pentadecanoic acid (15:0) | 0,37 % | 1,19 (1,00; 1,42) |  |  |
|  |  |  |  |  |  | Margaric acid (17:0) | 0,17 % | 1,02 (0,67; 1,55) |  |  |
|  | Iceland, AGESR | 5,2 years | PPL, GC | 753 (28), m/f, 75,5 years |  | Pentadecanoic acid (15:0) | 0,11 % | 1,51 (0,49; 4,69) |  |  |
|  |  |  |  |  |  | Margaric acid (17:0) | 0,18 % | 0,50 (0,17; 1,50) |  |  |
|  |  |  |  |  |  | *Trans-*Palmitoleic acid (*t*-16:1n–7) | 0,18 % | 1,14 (0,42; 3,12) |  |  |
| Imamura, 2020, FORCE (323) | Sweden, PIVUS | 10 years | Total plasma, GC | 879 (67), m/f, 72,5 years | Most participating cohorts, incident T2D was defined based on ≥1 criterion: FBG ≥126 mg/dL; 2-hour OGTT ≥200 mg/dL; new use of insulin or oral hypoglycaemic medication assessed by participant reports, medication inventories or registries; and fasting or non-fasting HbA1C concentration ≥6.5%. In the MCCS and the AOC, incident T2D was defined by self-reported physician diagnosis, use of antidiabetic medication, or both. | Palmitoleic acid (16:1n-7) | Per 0,51 % | 0,66 (0,32; 1,34) | Field site, sex, age, race/ethnicity, socioeconomic characteristics (education, occupation), smoking status, alcohol consumption, physical activity, family history of diabetes, dyslipidemia, hypertension, menopausal status, prevalent coronary heart disease, BMI, waist circumference | Moderate |
|  |  |  |  |  |  | Oleic acid (18:1n-9) | Per 2,80 % | 0,82 (0,42; 1,58) |  |  |
|  |  |  |  |  |  | Palmitic acid (16:0) | Per 2,90 % | 1,26 (0,75; 2,12) |  |  |
|  |  |  |  |  |  | Stearic acid (18:0) | Per 2,50 % | 1,07 (0,58; 1,98) |  |  |
|  | Sweden, ULSAM | 21,4 years | AT, GC | 2009 (396), m, 54,4 years |  | Palmitoleic acid (16:1n-7) | Per 4,11 % | 0,90 (0,55; 1,48) |  |  |
|  |  |  |  |  |  | Oleic acid (18:1n-9) | Per 5,70 % | 1,62 (0,91; 2,86) |  |  |
|  |  |  |  |  |  | Palmitic acid (16:0) | Per 4,90 % | 2,22 (1,35; 3,64) |  |  |
|  |  |  |  |  |  | Stearic acid (18:0) | Per 2,50 % | 1,46 (0,81; 2,62) |  |  |
|  | USA, CHS | 10,6 years | PPL, GC | 3179 (284), m/f, 75,1 years |  | Palmitoleic acid (16:1n-7) | Per 0,45 % | 1,10 (0,86; 1,42) |  |  |
|  |  |  |  |  |  | Oleic acid (18:1n-9) | Per 2,70 % | 0,97 (0,71; 1,33) |  |  |
|  |  |  |  |  |  | Palmitic acid (16:0) | Per 4,00 % | 1,67 (1,26; 2,23) |  |  |
|  |  |  |  |  |  | Stearic acid (18:0) | Per 2,80 % | 1,06 (0,76; 1,50) |  |  |
|  | Finland, METSIM | 5,5 years | Total plasma, GC | 1302 (71), m, 57,3 years |  | Palmitoleic acid (16:1n-7) | Per 0,58 % | 1,21 (0,82; 1,79) |  |  |
|  |  |  |  |  |  | Oleic acid (18:1n-9) | Per 3,10 % | 0,87 (0,51; 1,50) |  |  |
|  |  |  |  |  |  | Palmitic acid (16:0) | Per 3,30 % | 0,77 (0,40; 1,47) |  |  |
|  |  |  |  |  |  | Stearic acid (18:0) | Per 2,50 % | 2,38 (1,11; 5,10) |  |  |
|  | USA, MESA | 9,3 | PPL, GC | 2252 (309), m/f, 61 years |  | Palmitoleic acid (16:1n-7) | Per 0,54 % | 1,25 (0,71; 2,22) |  |  |
|  |  |  |  |  |  | Oleic acid (18:1n-9) | Per 3,20 % | 0,75 (0,37; 1,52) |  |  |
|  |  |  |  |  |  | Palmitic acid (16:0) | Per 4,00 % | 1,37 (0,86; 2,19) |  |  |
|  |  |  |  |  |  | Stearic acid (18:0) | Per 4,10 % | 0,88 (0,48;1,61) |  |  |
|  | USA, NHS | 16,9 | RBC, GC | 1760 (177), f, 60,4 years |  | Palmitoleic acid (16:1n-7) | Per 2,11 % | 1,26 (1,14; 1,40) |  |  |
|  |  |  |  |  |  | Oleic acid (18:1n-9) | Per 6,30 % | 1,76 (1,28; 2,41) |  |  |
|  |  |  |  |  |  | Palmitic acid (16:0) | Per 6,80 % | 1,33 (0,89; 1,99) |  |  |
|  |  |  |  |  |  | Stearic acid (18:0) | Per 8,80 % | 0,77 (0,55; 1,08) |  |  |
|  | USA, WHIMS | 11 years | RBC, GC | 6510 (502), f, 70,1 years |  | Palmitoleic acid (16:1n-7) | Per 0,49 % | 1,31 (1,08; 1,59) |  |  |
|  |  |  |  |  |  | Oleic acid (18:1n-9) | Per 2,70 % | 1,03 (0,79; 1,34) |  |  |
|  |  |  |  |  |  | Palmitic acid (16:0) | Per 3,80 % | 1,67 (1,34; 2,09) |  |  |
|  |  |  |  |  |  | Stearic acid (18:0) | Per 3,00 % | 0,95 (0,74; 1,23) |  |  |
|  | USA, FHS | 5,8 years | RBC, GC | 2209 (98), m/f, 64,4 years |  | Palmitoleic acid (16:1n-7) | Per 0,42 % | 1,36 (0,78; 2,39) |  |  |
|  |  |  |  |  |  | Oleic acid (18:1n-9) | Per 2,40 % | 0,99 (0,58; 1,70) |  |  |
|  |  |  |  |  |  | Palmitic acid (16:0) | Per 3,10 % | 1,89 (1,04; 3,44) |  |  |
|  |  |  |  |  |  | Stearic acid (18:0) | Per 2,40 % | 1,13 (0,63; 2,02) |  |  |
|  | Europe, EPIC-InterAct | 12,3 years | PPL, GC | 27296 (12132), m/f, 52,3 years  **340234 (12403)* |  | Palmitoleic acid (16:1n-7) | Per 0,49 % | 1,42 (1,30; 1,56) |  |  |
|  |  |  |  |  |  | Oleic acid (18:1n-9) | Per 4,20 % | 1,09 (0,93; 1,28) |  |  |
|  |  |  |  |  |  | Palmitic acid (16:0) | Per 4,00 % | 1,83 (1,49; 2,26) |  |  |
|  |  |  |  |  |  | Stearic acid (18:0) | Per 3,20 % | 1,08 (0,92; 1,25) |  |  |
|  | Australia, MCCS | 4 years | PPL, GC | 6151 (490), m/f, 56,3 years  **21523 (929)* |  | Palmitoleic acid (16:1n-7) | Per 0,41 % | 1,57 (1,26; 1,94) |  |  |
|  |  |  |  |  |  | Oleic acid (18:1n-9) | Per 3,60 % | 0,87 (0,68; 1,12) |  |  |
|  |  |  |  |  |  | Palmitic acid (16:0) | Per 3,80 % | 1,11 (0,85; 1,45) |  |  |
|  |  |  |  |  |  | Stearic acid (18:0) | Per 3,10 % | 2,16 (1,65; 2,82) |  |  |
|  | Netherlands, AOC | 2,5 years | CE, GC | 1741 (201), m/f, 68,9 years |  | Palmitoleic acid (16:1n-7) | Per 2,64 % | 1,58 (1,19; 2,12) |  |  |
|  |  |  |  |  |  | Oleic acid (18:1n-9) | Per 5,60 % | 1,55 (1,11; 2,15) |  |  |
|  |  |  |  |  |  | Palmitic acid (16:0) | Per 2,00 % | 1,50 (1,15; 1,97) |  |  |
|  |  |  |  |  |  | Stearic acid (18:0) | Per 0,40 % | 1,25 (1,04; 1,49) |  |  |
|  | Finland, KIHD | 10,3 years | Total plasma, GC | 1543 (205), m/f, 62,7 years |  | Palmitoleic acid (16:1n-7) | Per 2,36 % | 1,59 (1,13; 2,22) |  |  |
|  |  |  |  |  |  | Oleic acid (18:1n-9) | Per 5,90 % | 1,45 (1,00; 2,09) |  |  |
|  |  |  |  |  |  | Palmitic acid (16:0) | Per 4,10 % | 2,37 (1,69; 3,32) |  |  |
|  |  |  |  |  |  | Stearic acid (18:0) | Per 1,60 % | 0,96 (0,67; 1,39) |  |  |
|  | USA, HPFS | 11,1 years | RBC, GC | 1519 (112), m, 64,1 years |  | Palmitoleic acid (16:1n-7) | Per 1,60 % | 1,86 (1,26; 2,76) |  |  |
|  |  |  |  |  |  | Oleic acid (18:1n-9) | Per 7,50 % | 1,21 (1,00; 1,48) |  |  |
|  |  |  |  |  |  | Palmitic acid (16:0) | Per 4,80 % | 1,60 (0,99; 2,59) |  |  |
|  |  |  |  |  |  | Stearic acid (18:0) | Per 5,80 % | 1,75 (1,27; 2,42) |  |  |
|  | USA, IRAS | 5,3 years | Total plasma, GC | 719 (146), m/f, 55,1 years |  | Palmitoleic acid (16:1n-7) | Per 2,24 % | 2,07 (1,25; 3,43) |  |  |
|  |  |  |  |  |  | Oleic acid (18:1n-9) | Per 6,90 % | 2,53 (1,40; 4,58) |  |  |
|  |  |  |  |  |  | Palmitic acid (16:0) | Per 5,10 % | 2,35 (1,43; 3,87) |  |  |
|  |  |  |  |  |  | Stearic acid (18:0) | Per 1,80 % | 0,76 (0,45; 1,28) |  |  |
|  | France, 3C Study | 8,0 years | Total plasma, GC | 565 (39), m/f, 76 years |  | Palmitoleic acid (16:1n-7) | Per 2,30 % | 2,29 (1,08; 4,82) |  |  |
|  |  |  |  |  |  | Oleic acid (18:1n-9) | Per 8,50 % | 1,09 (0,43; 2,74) |  |  |
|  |  |  |  |  |  | Palmitic acid (16:0) | Per 4,80 % | 1,75 (0,91; 3,38) |  |  |
|  |  |  |  |  |  | Stearic acid (18:0) | Per 2,50 % | 0,37 (0,16; 0,87) |  |  |
|  | Iceland, AGESR | 5,2 years | Total plasma, GC | 753 (28), m/f, 75,5 years |  | Palmitoleic acid (16:1n-7) | Per 0,30 % | 2,54 (1,65; 3,92) |  |  |
|  |  |  |  |  |  | Oleic acid (18:1n-9) | Per 3,40 % | 2,41 (0,93; 6,21) |  |  |
|  |  |  |  |  |  | Palmitic acid (16:0) | Per 3,10 % | 2,68 (0,93; 7,73) |  |  |
|  |  |  |  |  |  | Stearic acid (18:0) | Per 2,40 % | 0,93 (0,36; 2,43) |  |  |
|  | Taiwan, CCCC | 6,0 years | Total plasma, GC | 1,838 (128), m/f, 58,7 years |  | Stearic acid (18:0) | Per 2,80 % | 0,58 (0,36; 0,95) |  |  |
|  |  |  |  |  |  | Palmitic acid (16:0) | Per 10,7 % | 0,8 (0,49; 1,32) |  |  |
| Krachler, 2007, Sweden, VIP-survey (324) | | 8,8 years | RBC, GLC | 450 (159), m/f, 50 years  **36196 (1184)* | Information on T2D incidence was obtained from register of diagnoses from the Departments of Internal Medicine and Cardiology at the Umea University Hospital, and from all primary care centres. Case records were verified for correct clinical T2D diagnosis according to 1998 WHO definitions. | Total SFA | Per 2,45 % | 0,96 \|0,68; 1,36\| | Age, sex, survey year, alcohol, BMI, HbA1c | High |
|  |  |  |  |  |  | Myristic acid (14:0) | Per 0,16 % | 0,99 \|0,72; 1,37\| |  |  |
|  |  |  |  |  |  | Pentadecanoic acid (15:0) | Per 0,06 % | 0,71 \|0,52; 0,97\| |  |  |
|  |  |  |  |  |  | Palmitic acid (16:0) | Per 1,90 % | 0,96 \|0,69; 1,34\| |  |  |
|  |  |  |  |  |  | Margaric acid (17:0) | Per 0,06 % | 0,54 \|0,35; 0,83\| |  |  |
|  |  |  |  |  |  | Stearic acid (18:0) | Per 0,95 % | 1,05 \|0,71; 1,56\| |  |  |
|  |  |  |  |  |  | Total MUFA | Per 1,49 % | 0,91 \|0,66; 1,26\| |  |  |
|  |  |  |  |  |  | Palmitoleic acid (16:1n-7) | Per 0,30 % | 1,32 \|0,91; 1,89\| |  |  |
|  |  |  |  |  |  | Oleic acid (18:1n-9) | Per 1,34 % | 0,86 \|0,62; 1,19\| |  |  |
|  |  |  |  |  |  | Total PUFA | Per 3,03 % | 1,08 \|0,77; 1,52\| |  |  |
|  |  |  |  |  |  | Linoleic acid (18:2) | Per 1,74 % | 0,93 \|0,62; 1,39\| |  |  |
|  |  |  |  |  |  | α-Linolenic acid (α-18:3) | Per 0,12 % | 0,85 \|0,61; 1,20\| |  |  |
|  |  |  |  |  |  | Dihomo-ɣ-Linolenic acid (ɣ-20:3) | Per 0,31 % | 1,21 \|0,88; 1,65\| |  |  |
|  |  |  |  |  |  | Arachidonic acid (20:4) | Per 1,39 % | 1,19 \|0,86; 1,64\| |  |  |
|  |  |  |  |  |  | Eicosapentaenoic acid (20:5) | Per 0,46 % | 0,75 \|0,53; 1,05\| |  |  |
|  |  |  |  |  |  | Docosatetraenoic acid (22:4) | Per 0,43 % | 1,49 \|1,05; 2,13\| |  |  |
|  |  |  |  |  |  | Docosapentaenoic acid (22:5) | Per 0,35 % | 0,90 \|0,66; 1,24\| |  |  |
|  |  |  |  |  |  | Docosahexaenoic acid (22:6) | Per 1,02 % | 1,02 \|0,74; 1,40\| |  |  |
| Kröger, 2011, Germany, EPIC-Potsdam Study (325) | | 7 years | RBC, GC | 2724 (673), m/f, 50 years  **27548 (849)* | Incident cases of T2D were identified via self-reports of diagnosis, diabetes-relevant medication, or dietary treatment because of diabetes. All incident cases were verified by questionnaires mailed to the diagnosing physician. Only cases with a physician diagnosis of T2D [(ICD 10): E11] and a diagnosis date after the baseline examination were considered as confirmed incident cases of T2D. | Total SFA | 41,20 %  42,40 %  43,30 %  44,40 %  48,60 % | 1,00  1,04 (0,71; 1,52)  0,90 (0,61; 1,34)  1,40 (0,97; 2,01)  1,24 (0,87; 1,76) | Age, sex, BMI, waist circumference, physical activity, education, smoking, alcohol, occupational activity, coffee intake (energy adjusted), fibre intake (energy adjusted) | Moderate |
|  |  |  |  |  |  | Myristic acid (14:0) | 0,23 %  0,31 %  0,38 %  0,45 %  0,57 % | 1,00  0,76 (0,55; 1,05)  0,79 (0,57; 1,11)  0,73 (0,52; 1,03)  0,32 (0,28; 0,97) |  |  |
|  |  |  |  |  |  | Pentadecanoic acid (15:0) | 0,13 %  0,18 %  0,21 %  0,25 %  0,32 % | 1,00  0,81 (0,58; 1,14)  1,04 (0,74; 1,47)  0,82 (0,58; 1,15)  0,79 (0,54; 1,16) |  |  |
|  |  |  |  |  |  | Palmitic acid (16:0) | 19,80 %  21,30 %  22,30 %  23,30 %  25,60 % | 1,00  1,11 (0,81; 1,53)  0,73 (0,52; 1,01)  0,91 (0,65; 1,28)  0,78 (0,55; 1,1) |  |  |
|  |  |  |  |  |  | Margaric acid (17:0) | 0,26 %  0,30 %  0,33 %  0,36 %  0,41 % | 1,00  0,82 (0,58; 1,14)  0,96 (0,69; 1,34)  0,83 (0,58; 1,19)  0,74 (0,52; 1,06) |  |  |
|  |  |  |  |  |  | Stearic acid (18:0) | 12,20 %  13,20 %  13,90 %  14,40 %  15,10 % | 1,00  1,34 (0,92; 1,96)  1,16 (0,79; 1,71)  1,65 (1,14; 2,37)  1,38 (0,96; 2,00) |  |  |
|  |  |  |  |  |  | Arachidic acid (20:0) | 0,30 %  0,35 %  0,39 %  0,44 %  0,52 % | 1,00  0,83 (0,60; 1,15)  0,89 (0,65; 1,23)  0,88 (0,63; 1,22)  0,66 (0,47; 0,94) |  |  |
|  |  |  |  |  |  | Behenic acid (22:0) | 1,16 %  1,39 %  1,59 %  1,8 %  2,15 % | 1,00  1,24 (0,90; 1,72)  1,08 (0,76; 1,52)  1,06 (0,76; 1,47)  1,14 (0,80; 1,61) |  |  |
|  |  |  |  |  |  | Lignoceric acid (24:0) | 3,1 %  3,7 %  4,2 %  4,8 %  5,6 % | 1,00  1,33 (0,91; 1,93)  1,38 (0,96; 1,98)  1,31 (0,91; 1,87)  1,56 (1,11; 2,21) |  |  |
|  |  |  |  |  |  | Total MUFA | 17,50 %  18,40 %  19,10 %  19,90 %  22,00 % | 1,00  1,00 (0,69; 1,46)  1,10 (0,77; 1,59)  1,07 (0,75; 1,52)  1,10 (0,78; 1,57) |  |  |
|  |  |  |  |  |  | Palmitoleic acid (16:1n-7) | 0,28 %  0,38 %  0,45 %  0,55 %  0,73 % | 1,00  1,14 (0,75; 1,73)  1,55 (1,06; 2,26)  1,57 (1,08; 2,28)  2,11 (1,46; 3,05) |  |  |
|  |  |  |  |  |  | Palmitoelaidic acid (16:1n-9) | 0,06 %  0,08 %  0,10 %  0,13 %  0,18 % | 1,00  0,88 (0,60; 1,30)  1,01 (0,70; 1,46)  1,14 (0,81; 1,61)  1,45 (1,04; 2,02) |  |  |
|  |  |  |  |  |  | Trans-Vaccenic acid (*t-*18:1n-7) | 0,88 %  0,96 %  1,03 %  1,10 %  1,23 % | 1,00  0,97 (0,69; 1,35)  0,83 (0,59; 1,17)  0,80 (0,56; 1,15)  0,95 (0,69; 1,32) |  |  |
|  |  |  |  |  |  | Oleic acid (18:1n-9) | 11,4 %  12,2 %  12,8 %  13,4 %  14,7 % | 1,00  1,21 (0,85; 1,72)  1,01 (0,70; 1,46)  0,88 (0,61; 1,27)  1,15 (0,81; 1,62) |  |  |
|  |  |  |  |  |  | Eicosenoic acid (20:1) | 0,23 %  0,26 %  0,29 %  0,31 %  0,36 % | 1,00  1,31 (0,93; 1,83)  1,22 (0,87; 1,71)  1,16 (0,82; 1,63)  0,99 (0,69; 1,40) |  |  |
|  |  |  |  |  |  | Nervonic acid (24:1) | 3,08 %  3,67 %  4,14 %  4,62 %  5,34 % | 1,00  0,97 (0,67; 1,41)  1,26 (0,90; 1,78)  1,09 (0,77; 1,53)  1,17 (0,83; 1,64) |  |  |
|  |  |  |  |  |  | *Trans-*Palmitoleic acid (*t*-16:1n–7) | 0,12 %  0,15 %  0,17 %  0,20 %  0,27 % | 1,00  1,04 (0,76; 1,42)  0,82 (0,58; 1,15)  0,75 (0,53; 1,07)  0,89 (0,64; 1,23) |  |  |
|  |  |  |  |  |  | Total PUFA | 29,8 %  35,6 %  36,9 %  38,1 %  39,7 % | 1,00  1,34 (0,99; 1,83) 0,57 (0,40; 0,82) 0,97 (0,69; 1,36) 1,01 (0,72; 1,41) |  |  |
|  |  |  |  |  |  | Total n-6 PUFAs | 23,7 %  27,1 %  28,4 %  29,5 %  31,0 % | 1,00  1,16 (0,86; 1,56) 0,86 (0,62; 1,20) 0,83 (0,58; 1,17) 0,89 (0,62; 1,28) |  |  |
|  |  |  |  |  |  | Linoleic acid (18:2) | 9,1 %  10,0 %  10,7 %  11,3 %  12,4 % | 1,00  0,84 (0,62; 1,13) 0,61 (0,44; 0,85) 0,50 (0,35; 0,72) 0,76 (0,54; 1,08) |  |  |
|  |  |  |  |  |  | ɣ-Linolenic acid (ɣ-18:3) | 0,027 %  0,042 %  0,054 %  0,067 %  0,091 % | 1,00  1,25 (0,83; 1,87) 1,63 (1,11; 2,40) 1,51 (1,03; 2,21) 2,00 (1,38; 2,88) |  |  |
|  |  |  |  |  |  | Eicosadienoic acid (20:2) | 0,21 %  0,23 %  0,25 %  0,27 %  0,31 % | 1,00  0,89 (0,65; 1,21) 0,86 (0,62; 1,20) 0,78 (0,56; 1,08) 0,98 (0,70; 1,38) |  |  |
|  |  |  |  |  |  | Dihomo-ɣ-Linolenic acid (ɣ-20:3) | 1,12 %  1,33 %  1,47 %  1,64 %  1,89 % | 1,00  1,50 (1,02; 2,21) 1,05 (0,71; 1,57) 1,70 (1,17; 2,46) 1,72 (1,18; 2,53) |  |  |
|  |  |  |  |  |  | Arachidonic acid (20:4) | 9,5 %  12,2 %  13,1 %  13,8 %  14,9 % | 1,00  0,97 (0,71; 1,34) 1,00 (0,72; 1,38) 0,96 (0,69; 1,34) 0,76 (0,54; 1,08) |  |  |
|  |  |  |  |  |  | Docosatetraenoic acid (22:4) | 1,7 %  2,4 %  2,7 %  3,0 %  3,4 % | 1,00  1,00 (0,73; 1,36) 0,81 (0,59; 1,12) 0,68 (0,48; 0,97) 1,24 (0,89; 1,72) |  |  |
|  |  |  |  |  |  | Total n-3 PUFAs | 5,0 %  7,2 %  8,0 %  8,9 %  10,1 % | 1,00  0,83 (0,58; 1,19) 1,24 (0,89; 1,73) 0,79 (0,56; 1,12) 0,98 (0,71; 1,36) |  |  |
|  |  |  |  |  |  | α-Linolenic acid (α-18:3) | 0,10 %  0,13 %  0,15 %  0,17 %  0,22 % | 1,00  0,98 (0,72; 1,34) 0,89 (0,64; 1,25) 0,97 (0,69; 1,35) 0,92 (0,66; 1,28) |  |  |
|  |  |  |  |  |  | Eicosapentaenoic acid (20:5) | 0,42 %  0,61 %  0,75 %  0,90 %  1,20 % | 1,00  0,85 (0,60; 1,22) 0,69 (0,48; 0,98) 0,84 (0,60; 1,18) 0,78 (0,55; 1,09) |  |  |
|  |  |  |  |  |  | Docosapentaenoic acid (22:5) | 1,5 %  2,1 %  2,3 %  2,5 %  2,8 % | 1,00  1,15 (0,82; 1,62) 0,98 (0,69; 1,38) 0,97 (0,70; 1,36) 0,88 (0,62; 1,23) |  |  |
|  |  |  |  |  |  | Docosahexaenoic acid (22:6) | 2,7 %  4,1 %  4,7 %  5,3 %  6,2 % | 1,00  1,17 (0,83; 1,65) 1,02 (0,71; 1,45) 1,11 (0,79; 1,55) 1,09 (0,78; 1,54) |  |  |
| Lai, 2022 (326) | Iceland, AGESR | 7,8 years | PPL, GC | 753 (28), m/f, 76 years | Incident T2D was ascertained by each cohort based on established combinations of self-report together with physician’s diagnosis using medical records, FBG ≥126 mg/dL, OGTT >200 mg/dL, HbA1c ≥6.5%, or use of insulin or oral hypoglycaemic medication. | *Trans*-Hypogeic acid (*t-*16:1n-9) | Per 0,04 % | 0,57 (0,16; 2,00) | Age, sex, race, field site (if applicable), education, occupation, physical activity, smoking, alcohol use, prevalent hypertension, prevalent dyslipidaemia, prevalent coronary heart disease, BMI, waist circumference, circulating Palmitic acid (16:0), circulating Stearic acid (18:0), circulating Linoleic acid (18:2), and TG. | Moderate |
|  |  |  |  |  |  | *Trans*-Elaidic acid (*t-*18:1n–9) | Per 0,65 % | 0,71 (0,19; 2,65) |  |  |
|  |  |  |  |  |  | *Trans*-Linoleic acid (*t-*18:2) | Per 0,07 % | 0,97 (0,29; 3,21) |  |  |
|  | Taiwan, CCC | 10,3 years | Total plasma, GC | 1443 (302), m/f, 60 years |  | *Trans*-Hypogeic acid (*t-*16:1n-9) | Per 0,95 % | 0,80 (0,50; 1,27) |  |  |
|  |  |  |  |  |  | *Trans*-Elaidic acid (*t-*18:1n–9) | Per 1,70 % | 0,85 (0,59; 1,22) |  |  |
|  |  |  |  |  |  | *Trans*-Linoleic acid (*t-*18:2) | Per 3,18 % | 0,65 (0,39; 1,08) |  |  |
|  | USA, CHS | 22,1 years | PPL, GC | 3007 (291), m/f, 75 years |  | *Trans*-Hypogeic acid (*t-*16:1n-9) | Per 0,07 % | 1,12 (0,75; 1,68) |  |  |
|  |  |  |  |  |  | *Trans*-Elaidic acid (*t-*18:1n–9) | Per 1,76 % | 1,39 (0,85; 2,28) |  |  |
|  |  |  |  |  |  | *Trans*-Linoleic acid (*t-*18:2) | Per 0,18 % | 0,87 (0,64; 1,19) |  |  |
|  | UK, EPIC-Norfolk | 12,1 years | RBC, GC | 383 (199), m/f, 64 years  **25639 (892)* |  | *Trans*-Hypogeic acid (*t-*16:1n-9) | Per 0,07 % | 0,80 (0,45; 1,43) |  |  |
|  |  |  |  |  |  | *Trans*-Elaidic acid (*t-*18:1n–9) | Per 0,58 % | 1,33 (0,54; 3,27) |  |  |
|  | Germany, EPIC-Potsdam | 10,1 years | RBC, GC | 2165 (488), m/f, 49 years  **27548 (849)* |  | *Trans*-Elaidic acid (*t-*18:1n–9) | Per 0,29 % | 1,00 (0,72; 1,39) |  |  |
|  | USA, FHS | 5,2 years | RBC, GC | 1870 (95), m/f, 64 years |  | *Trans*-Elaidic acid (*t-*18:1n–9) | Per 1,26 % | 0,85 (0,44; 1,66) |  |  |
|  |  |  |  |  |  | *Trans*-Linoleic acid (*t-*18:2) | Per 0,22 % | 0,95 (0,57; 1,59) |  |  |
|  | USA, HPFS | 20,2 years | RBC, GC | 1519 (112), m, 65 years |  | *Trans*-Elaidic acid (*t-*18:1n–9) | Per 1,43 % | 1,43 (0,89; 2,29) |  |  |
|  |  |  |  |  |  | *Trans*-Linoleic acid (*t-*18:2) | Per 0,15 % | 1,51 (1,05; 2,17) |  |  |
|  | Australia, MCCS | 9,9 years | PPL, GC | 2545 (205), m/f, 55 years  **21523 (929)* |  | T*rans*-Hypogeic acid (*t-*16:1n-9) | Per 0,01 % | 0,65 (0,38; 1,11) |  |  |
|  |  |  |  |  |  | *Trans*-Elaidic acid (*t-*18:1n–9) | Per 0,80 % | 0,18 (0,08; 0,40) |  |  |
|  |  |  |  |  |  | *Trans*-Linoleic acid (*t-*18:2) | Per 0,09 % | 0,58 (0,38; 0,89) |  |  |
|  | USA, MESA | 11,2 years | PPL, GC | 2234 (297), m/f, 61 years |  | T*rans*-Hypogeic acid (*t-*16:1n-9) | Per 0,07 % | 0,68 (0,45; 1,01) |  |  |
|  |  |  |  |  |  | *Trans*-Elaidic acid (*t-*18:1n–9) | Per 1,68 % | 0,47 (0,29; 0,77) |  |  |
|  |  |  |  |  |  | *Trans*-Linoleic acid (*t-*18:2) | Per 0,18 % | 0,64 (0,44; 0,92) |  |  |
|  | USA, NHS | 24,8 years | RBC,  GC | 1482 (152), f, 60 years |  | *Trans*-Elaidic acid (*t-*18:1n–9) | Per 1,83 % | 0,68 (0,40; 1,17) |  |  |
|  |  |  |  |  |  | *Trans*-Linoleic acid (*t-*18:2) | Per 0,30 % | 0,69 (0,43; 1,09) |  |  |
|  | USA, PHS | 13,9 years | RBC, GC | 941 (53), m, 69 years |  | T*rans*-Hypogeic acid (*t-*16:1n-9) | Per 0,07 % | 0,94 (0,50; 1,79) |  |  |
|  |  |  |  |  |  | *Trans*-Elaidic acid (*t-*18:1n–9) | Per 0,99 % | 0,83 (0,39; 1,21) |  |  |
|  |  |  |  |  |  | *Trans*-Linoleic acid (*t-*18:2) | Per 0,31 % | 0,71 (0,42; 1,21) |  |  |
|  | USA, WHIMS | 14,1 years | RBC, GC | 5668 (490), f, 70 years |  | *Trans*-Elaidic acid (*t-*18:1n–9) | Per 1,57 % | 0,86 (0,65; 1,15) |  |  |
|  |  |  |  |  |  | *Trans*-Linoleic acid (*t-*18:2) | Per 0,40 % | 0,92 (0,69; 1,23) |  |  |
| Lankinen, 2015, Finland, METSIM (327) | | 5,9 years | PPL, GC | 1302 (71), m, 56,5 years | Incident T2D were diagnosed based on an OGTT or on HbA1c measurements at the follow-up visit or on National Drug Reimbursement registry data (drug treatment started for diabetes during the follow-up; participants who did not have an OGTT). | Total SFA | Per 0,90 % | 1,17 [0,90; 1,53] | Age, BMI, smoking, physical activity, FBG at baseline | High |
|  |  |  |  |  |  | Myristic acid (14:0) | Per 0,10 % | 0,97 [0,76; 1,25] |  |  |
|  |  |  |  |  |  | Total MUFA | Per 1,50 % | 0,99 [0,77; 1,26] |  |  |
|  |  |  |  |  |  | Trans-Vaccenic acid (*t-*18:1n-7) | Per 0,20 % | 0,94 [0,74; 1,20] |  |  |
|  |  |  |  |  |  | Eicosenoic acid (20:1) | Per 0,10 % | 0,98 [0,77; 1,26] |  |  |
|  |  |  |  |  |  | Nervonic acid (24:1) | Per 0,30 % | 0,85 [0,67; 1,08] |  |  |
|  |  |  |  |  |  | Total PUFA | Per 1,80 % | 0,94 [0,73; 1,21] |  |  |
|  |  |  |  |  |  | Linoleic acid (18:2) | Per 2,7 % | 0,86 [0,67; 1,10] |  |  |
|  |  |  |  |  |  | α-Linolenic acid (α-18:3) | Per 0,1 % | 1,08 [0,85; 1,37] |  |  |
|  |  |  |  |  |  | Arachidonic acid (20:4) | Per 1,6 % | 1,11 [0,87; 1,42] |  |  |
|  |  |  |  |  |  | Dihomo-ɣ-Linolenic acid (ɣ-20:3) | Per 0,60 % | 1,46 [1,16; 1,84] |  |  |
|  |  |  |  |  |  | Eicosapentaenoic acid (20:5) | Per 1,1 % | 0,99 [0,78; 1,25] |  |  |
|  |  |  |  |  |  | Docosatetraenoic acid (22:4) | Per 1,60 % | 1,11 [0,87; 1,42] |  |  |
|  |  |  |  |  |  | Docosapentaenoic acid (22:5) | Per 0,1 % | 1,22 [0,97; 1,54] |  |  |
|  |  |  |  |  |  | Docosahexaenoic acid (22:6) | Per 1,4 % | 0,93 [0,73; 1,19] |  |  |
|  |  |  | CE, GC |  |  | Total SFA | Per 1,1 % | 1,14 [0,91; 1,43] |  |  |
|  |  |  |  |  |  | Myristic acid (14:0) | Per 0,3 % | 1,06 [0,83; 1,35] |  |  |
|  |  |  |  |  |  | Palmitic acid (16:0) | Per 0,9 % | 1,14 [0,90; 1,44] |  |  |
|  |  |  |  |  |  | Stearic acid (18:0) | Per 0,2 % | 1,10 [0,88; 1,36] |  |  |
|  |  |  |  |  |  | Total MUFA | Per 3,70 % | 1,07 [0,85; 1,35] |  |  |
|  |  |  |  |  |  | Palmitoleic acid (16:1n-7) | Per 1,60 % | 1,07 [0,87; 1,32] |  |  |
|  |  |  |  |  |  | Trans-Vaccenic acid (*t-*18:1n-7) | Per 0,20 % | 0,91 [0,72; 1,14] |  |  |
|  |  |  |  |  |  | Oleic acid (18:1n-9) | Per 2,30 % | 1,06 [0,83; 1,36] |  |  |
|  |  |  |  |  |  | Total PUFA | Per 4,2 % | 0,91 [0,72; 1,15] |  |  |
|  |  |  |  |  |  | ɣ-Linolenic acid (ɣ-18:3) | Per 0,40 % | 1,17 [0,94; 1,46] |  |  |
|  |  |  |  |  |  | Dihomo-ɣ-Linolenic acid (ɣ-20:3) | Per 0,2 % | 1,45 [1,15; 1,83] |  |  |
|  |  |  |  |  |  | Arachidonic acid (20:4) | Per 1,4 % | 1,15 [0,92; 1,44] |  |  |
|  |  |  | Triacylglycerol |  |  | Total SFA | Per 4,5 % | 1,19 [0,93; 1,53] |  |  |
|  |  |  |  |  |  | Myristic acid (14:0) | Per 0,9 % | 1,06 [0,82; 1,36] |  |  |
|  |  |  |  |  |  | Palmitic acid (16:0) | Per 3,5 % | 1,17 [0,90; 1,51] |  |  |
|  |  |  |  |  |  | Stearic acid (18:0) | Per 0,8 % | 1,24 [1,00; 1,55] |  |  |
|  |  |  |  |  |  | Total MUFA | Per 3,40 % | 0,97 [0,76; 1,22] |  |  |
|  |  |  |  |  |  | Palmitoleic acid (16:1n-7) | Per 1,20 % | 1,02 [0,82; 1,28] |  |  |
|  |  |  |  |  |  | Trans-Vaccenic acid (*t-*18:1n-7) | Per 0,40 % | 0,93 [0,74; 1,17] |  |  |
|  |  |  |  |  |  | Oleic acid (18:1n-9) | Per 3,10 % | 0,97 [0,76; 1,23] |  |  |
|  |  |  |  |  |  | Total PUFA | Per 5,2 % | 0,87 [0,67; 1,14] |  |  |
|  |  |  |  |  |  | Linoleic acid (18:2) | 3,3 % | 0,87 [0,67; 1,13] |  |  |
|  |  |  |  |  |  | ɣ-Linolenic acid (ɣ-18:3) | Per 0,2 % | 1,07 [0,84; 1,36] |  |  |
|  |  |  |  |  |  | Dihomo-ɣ-Linolenic acid (ɣ-20:3) | Per 0,1 % | 1,18 [0,95; 1,46] |  |  |
|  |  |  |  |  |  | Arachidonic acid (20:4) | Per 0,4 % | 0,89 [0,69; 1,15] |  |  |
| Lemaitre, 2015, USA, CHS (328) | | 10 years | PPL, GC | 3179 (284), m/f, 75 years | Incident T2D cases were defined by glucose ≥126 mg/dL when participants reported fasting ≥8 h before venepuncture, glucose ≥200 mg/dL when fasting was <8h, or use of insulin or oral hypoglycaemic medication. In secondary analyses, additional T2D cases identified from the Centres for Medicare & Medicaid Services records were included. | Arachidic acid (20:0) | 0,41 %  0,47 %  0,52 %  0,59 % | 1,00  0,99 [0,71; 1,38]  0,88 [0,62; 1,27]  0,68 [0,46; 1,00] | Age, race, sex, clinic, education, smoking, alcohol use, physical activity, treated hypertension, ischemic heart disease, self-reported health status, BMI and waist circumference, PPL levels of Palmitic acid (16:0) and TG levels. | Moderate |
|  |  |  |  |  |  | Behenic acid (22:0) | 1,31 %  1,55 %  1,74 %  2,02 % | 1,00  0,99 [0,69; 1,43]  0,95 [0,65; 1,37]  0,94 [0,63; 1,40] |  |  |
|  |  |  |  |  |  | Lignoceric acid (24:0) | 1,08 %  1,28 %  1,45 %  1,85 % | 1,00  0,90 [0,63; 1,27]  0,91 [0,63; 1,31]  0,84 [0,57; 1,24] |  |  |
| Lin, 2018, China, GNHS (329) | | 5,6 years | RBC, GC | 2683 (216), m/f, 57,5 years | Incident T2D cases were identified as FBG ≥126 mg/dL or HbA1c ≥6.5%, or as self-reported diabetic medications during the follow-up visits according to the American Diabetes Association criteria. | Myristic acid (14:0) | 0,19 %  0,24 %  0,29 %  0,40 % | 1,00  0,90 [0,59; 1,35]  0,89 [0,59; 1,33]  1,20 [0,82; 1,76] | Age, sex, BMI, waist to hip--ratio, smoking status, alcohol drinking, tea drinking, education level, household income, physical activity, family history of diabetes, total energy intake, TG, HDL, LDL and FBG | Moderate |
|  |  |  |  |  |  | Palmitic acid (16:0) | 23,7 %  26,5 %  28,4 %  31,5 % | 1,00  0,55 [0,37; 0,81]  0,53 [0,35; 0,78]  0,69 [0,48; 0,99] |  |  |
|  |  |  |  |  |  | Stearic acid (18:0) | 15,5 %  16,4 %  17,4 %  19,5 % | 1,00  1,19 [0,80; 1,79]  1,35 [0,91; 2,02]  1,49 [1,02; 2,19] |  |  |
|  |  |  |  |  |  | Arachidic acid (20:0) | 0,36 %  0,41 %  0,46 %  0,59 % | 1,00  1,04 [0,70; 1,54]  1,39 [0,96; 2,02]  1,46 [1,00; 2,12] |  |  |
|  |  |  |  |  |  | Behenic acid (22:0) | 0,46 %  1,48 %  1,74 %  2,07 % | 1,00  1,53 [1,05; 2,22]  1,61 [1,10; 2,36]  1,48 [0,99; 2,22] |  |  |
|  |  |  |  |  |  | Lignoceric acid (24:0) | 3,86 %  4,53 %  5,09 %  6,18 % | 1,00  0,99 [0,68; 1,44]  0,99 [0,68; 1,44]  1,08 [0,74; 1,56] |  |  |
| Ma, 2015, USA, CHS (196) | | 9 years | PPL, GC-mass spectrometry | 3004 (297), m/f, 74 years | Incident T2D cases defined by single measure of FBG concentration ≥126 mg/dL, non-fasting or 2-h OGTT ≥200 mg/dL, or new use of an insulin or oral hypoglycaemic medication. | Myristic acid (14:0) | 0,19 %  0,24 %  0,27 %  0,31 %  0,37 % | 1,00  1,31 [0,90; 1,90]  1,18 [0,81; 1,71]  1,22 [0,83; 1,79]  0,98 [0,65; 1,47] | Age, sex, race, education, clinic, smoking status, alcohol consumption, leisure time physical activity, prevalence of ischemic heart disease, hypertension at baseline, BMI, waist circumference, and consumption of carbohydrate (percentage of energy), protein (percentage of energy), and total energy. | Moderate |
|  |  |  |  |  |  | Trans-Vaccenic acid (*t-*18:1n-7) | 1,10 %  1,20 %  1,30 %  1,40 %  1,60 % | 1,00  0,72 [0,52; 1,00]  0,63 [0,44; 0,90]  0,72 [0,51; 1,01]  0,56 [0,38; 0,83] |  |  |
|  |  |  |  |  |  | Palmitioelaidic acid (16:1n-9) | 0,07 %  0,08 %  0,09 %  0,10 %  0,12 % | 1,00  0,97 [0,68; 1,37]  1,00 [0,71; 1,40]  0,92 [0,63; 1,33]  0,83 [0,67; 1,22] |  |  |
| Miao, 2020, China, GNHS (330) | | 6,2 years | RBC, GC | 2731 (276), m/f, 57,5 years | Incident T2D cases defined by a participant met one of the following criteria: a FBG concentration ≥126 mg/dL, HbA1c ≥6.5 %, or self-reported medical treatment for diabetes. | Total n-6 PUFA | 16,76 %  20,77 %  22,32 %  23,97 % | 1,00  1,02 (0,75; 1,39)  0,98 (0,70; 1,36)  1,05 (0,76; 1,47) | Age, sex, BMI, WHR, education, household income, smoking, alcohol, physical activity, total energy intake, family history of diabetes, baseline RBC total n-3 PUFAs, FBG. | Moderate |
|  |  |  |  |  |  | Linoleic acid (18:2) | 8,11 %  9,43 %  10,29 %  11,41 % | 1,00  0,93 (0,69; 1,24)  0,93 (0,69; 1,26)  0,91 (0,67; 1,24) |  |  |
|  |  |  |  |  |  | ɣ-Linolenic acid (ɣ-18:3) | 0,02 %  0,03 %  0,04 %  0,07 % | 1,00  1,22 (0,85; 1,74)  1,43 (1,01; 2,03)  1,72 (1,21; 2,44) |  |  |
|  |  |  |  |  |  | Arachidonic acid (20:4) | 7,73 %  10,90 %  12,02 %  13,41 % | 1,00  0,89 (0,65; 1,22)  0,96 (0,69; 1,35)  1,00 (0,71; 1,40) |  |  |
| Mozaffarian, 2010, USA, CHS (331) | | 9,3 years | PPL, GC | 3736 (304), m/f, 75,4 years | Incident T2D was diagnosed based on new use of insulin or hypoglycaemic medication, fasting glucose ≥126mg/dl, or 2-hour post-challenge glucose ≥200mg/dl. | *Trans-*Palmitoleic acid (*t*-16:1n–7) | 0,13 %  0,16 %  0,18 %  0,21 %  0,25 %  Per 0,5 % | 1,00  0,79 [0,54; 1,15]  0,89 [0,58; 1,33]  0,41 [0,27; 0,64]  0,38 [0,24; 0,62]  0,72 [0,61; 0,86] | Age, sex, race, education, enrolment site, smoking, alcohol, BMI, waist circumference, coronary heart disease, physical activity, carbohydrate intake, protein intake, red meat intake, whole fat dairy foods, low fat dairy foods, total energy | Moderate |
| Mozaffarian, 2013, USA, MESA (332) | | 7 years | PPL, GC | 2617 (205), m/f, 61,7 | Incident T2D was diagnosed on the basis of FBG ≥126 mg/dL or the new use of insulin or oral hypoglycaemic medications assessed at study clinic examinations. | *Trans-*Palmitoleic acid (*t*-16:1n–7) | 0,03 %  0,04 %  0,05 %  0,07 %  Per 0,03 % | 1,00  0,77 [0,47; 1,25]  0,66 [0,40; 1,10]  0,89 [0,58; 1,35]  0,80 [0,66; 0,96] | Age, sex, race, education, energy intake, BMI, waist circumference, field centre, smoking status, alcohol use, physical activity | Moderate |
| Patel, 2010, England, EPIC-Norfolk study (3) | | 5-11 years | RBC, GC | 383 (199), m/f, 59,5 years  **25639 (892)* | Incident T2D cases defined based on self-report of a physician’s diagnosis of diabetes or diabetes medication on any of the follow-up health and lifestyle questionnaires or diabetes medication brought to the follow-up health check visit. Linkage with general practice diabetes registers, hospital outpatient diabetes registers, and hospital admissions information for diabetes was applied for the verification of diagnoses. | Total SFA | 36,79 %  37,47 %  38,46 % | 1,00  1,08 (0,61; 1,91)  1,51 (0,85; 2,68) | Age, sex, family history of diabetes, BMI, smoking status, physical activity, alcohol intake | High |
|  |  |  |  |  |  | Myristic acid (14:0) | 0,34 %  0,42 %  0,54 % | 1,00  1,56 (0,88; 2,79)  1,52 (0,86; 2,69) |  |  |
|  |  |  |  |  |  | Palmitic acid (16:0) | 21,59 %  22,37 %  23,52 % | 1,00  1,71 (0,96; 3,05)  1,96 (1,10; 3,49) |  |  |
|  |  |  |  |  |  | Pentadecanoic acid (15:0) | 0,22 %  0,26 %  0,32 % | 1,00  1,31 (0,75; 2,30)  0,90 (0,50; 1,62) |  |  |
|  |  |  |  |  |  | Stearic acid (18:0) | 13,31 %  14,07 %  14,77 % | 1,00  1,03 (0,59; 1,81)  0,69 (0,39; 1,24) |  |  |
|  |  |  |  |  |  | Total MUFA | 16,32 %  17,61 %  18,97 % | 1,00  1,10 (0,62; 1,94)  1,21 (0,67; 2,19) |  |  |
|  |  |  |  |  |  | Palmitoleic acid (16:1n-7) | 0,42 %  0,55 %  0,73 % | 1,00  1,37 (0,76; 2,45)  2,57 (1,36; 4,84) |  |  |
|  |  |  |  |  |  | Trans-Vaccenic acid (*t-*18:1n-7) | 0,81 %  0,91 %  1,04 % | 1,00  1,31 (0,74; 2,33)  0,55 (0,31; 0,99) |  |  |
|  |  |  |  |  |  | Oleic acid (18:1n-9) | 11,44 %  12,48 %  13,70 % | 1,00  0,96 (0,54; 1,71)  1,13 (0,62; 2,04) |  |  |
|  |  |  |  |  |  | Eicosenoic acid (20:1) | 0,21 %  0,25 %  0,32 % | 1,00  1,02 (0,58; 1,77)  0,64 (0,36; 1,16) |  |  |
|  |  |  |  |  |  | Nervonic acid (24:1) | 2,64 %  3,32 %  4,12 % | 1,00  1,28 (0,73; 2,25)  1,02 (0,57; 1,80) |  |  |
|  |  |  |  |  |  | Total PUFA | 35,51 %  37,62 %  39,39 % | 1,00  0,61 (0,34; 1,09)  0,90 (0,51; 1,61) |  |  |
|  |  |  |  |  |  | Total n-6 PUFA | 25,90 %  28,40 %  30,82 % | 1,00  0,58 (0,33; 1,04)  0,74 (0,41; 1,32) |  |  |
|  |  |  |  |  |  | ɣ-Linolenic acid (ɣ-18:3) | 0,04 %  0,07 %  0,10 % | 1,00  1,22 (0,70; 2,11)  1,00 (0,55; 1,79) |  |  |
|  |  |  |  |  |  | Docosatetraenoic acid (22:4) | 1,92 %  2,45 %  3,02 % | 1,00  0,70 (0,39; 1,24)  1,17 (0,65; 2,11) |  |  |
|  |  |  |  |  |  | Total n-3 PUFA | 7,53 %  9,01 %  10,90 % | 1,00  1,20 (0,68; 2,11)  1,14 (0,64; 2,04) |  |  |
|  |  |  |  |  |  | α-Linolenic acid (α-18:3) | 0,10 %  0,14 %  0,19 % | 1,00  0,93 (0,53; 1,63)  0,95 (0,54; 1,69) |  |  |
|  |  |  |  |  |  | Eicosapentaenoic acid (20:5) | 0,61 %  0,90 %  1,49 % | 1,00  0,95 (0,54; 1,67)  0,85 (0,47; 1,52) |  |  |
|  |  |  |  |  |  | Docosapentaenoic acid (22:5) | 2,14 %  2,56 %  2,94 % | 1,00  0,81 (0,46; 1,42)  0,92 (0,51; 1,67) |  |  |
|  |  |  |  |  |  | Docosahexaenoic acid (22:6) | 2,14 %  2,56 %  2,94 % | 1,00  0,81 (0,46; 1,42)  0,92 (0,51; 1,67) |  |  |
|  |  |  | PPL, GC |  |  | Total SFA | 38,07 %  41,34 %  43,59 % | 1,00  1,39 (0,79; 2,46)  2,57 (1,42; 4,66) |  |  |
|  |  |  |  |  |  | Myristic acid (14:0) | 0,25 %  0,38 %  0,59 % | 1,00  1,18 (0,67; 2,08)  1,70 (0,95; 3,04) |  |  |
|  |  |  |  |  |  | Total MUFA | 10,10 %  12,15 %  14,65 % | 1,00  0,73 (0,42; 1,29)  1,24 (0,69; 2,23) |  |  |
|  |  |  |  |  |  | Trans-Vaccenic acid (*t-*18:1n-7) | 0,66 %  0,86 %  1,07 % | 1,00  0,73 (0,41; 1,28)  0,40 (0,22; 0,72) |  |  |
|  |  |  |  |  |  | Eicosenoic acid (20:1) | 0,08 %  0,12 %  0,19 % | 1,00  1,06 (0,60; 1,87)  0,48 (0,27; 0,87) |  |  |
|  |  |  |  |  |  | Nervonic acid (24:1) | 0,01 %  0,03 %  0,13 % | 1,00  1,36 (0,77; 2,39)  0,99 (0,56; 1,74) |  |  |
|  |  |  |  |  |  | *Trans*-Elaidic acid *(t-*18:1n–9) | 0,04 %  0,08 %  0,17 % | 1,00  0,86 (0,49; 1,53)  0,73 (0,41; 1,29) |  |  |
| Petriwi, 2020, Netherlands, AOC (340) | | 3,4 years | Total plasma CE, GC | 3257 (171), m/f, 68,9 years | Self-reported physician’s diagnosis and/ or the initiation of antidiabetic medication | Linoleic acid (18:2) | 43,9 %  47,6 %  50,1 %  52,8 %  56,3 % | 1,00  0,74 [0,48; 1,14]  0,64 [0,40; 1,00]  0,60 [0,38; 0,94]  0,44 [0,26; 0,75] | Age, sex, plus physical  Activity, smoking status, educational level, BMI, family history of T2D, total energy intake, alcohol intake, dietary fiber, and dietary cholesterol | High |
| Qian, 2021 (333) | France, 3C Study | 13,0 years | Total plasma, GC | 1218 (83), m/f, 74,4 years | Incident T2D was defined by one or more of the following definitions: 1) FBG ≥126 mg/dL, 2) HbA1c ≥6.5%, 3) 2-h OGTT ≥200 mg/dL, 4) self-reported use of oral hypoglycaemic medications or insulin, and 5) self-reported physician diagnosis or linkage to disease registries. | α-Linolenic acid (α-18:3) | Per 1,30 % | 0,56 (0,34; 0,94) | Sex, age, field site, race, socioeconomic status, smoking status, physical activity, alcohol consumption, treatment for hypertension, treatment for hypercholesterolemia, prevalent coronary heart disease, BMI, waist circumference, and biomarkers of Linoleic acid (18:2) and trans-FAs (*t-*18:1 and *t-*18:2) | Moderate |
|  |  |  |  |  |  | Eicosapentaenoic acid (20:5) | Per 0,40 % | 0,92 (0,66; 1,28) |  |  |
|  |  |  |  |  |  | Docosapentaenoic acid (22:5) | Per 2,80 % | 0,92 (0,66; 1,28) |  |  |
|  |  |  |  |  |  | Docosahexaenoic acid (22:6) | Per 0,40 % | 1,02 (0,65; 1,60) |  |  |
|  |  |  |  |  |  | EPA+DPA+DHA | Per 4,27 % | 0,94 (0,52; 1,71) |  |  |
|  | Iceland, AGESR | 7,8 years | PPL, GC | 753 (28), m/f, 75,5 years |  | α-Linolenic acid (α-18:3) | Per 0,16 % | 0,47 (0,17; 1,32) |  |  |
|  |  |  |  |  |  | Eicosapentaenoic acid (20:5) | Per 3,59 % | 0,74 (0,28; 1,94) |  |  |
|  |  |  |  |  |  | Docosapentaenoic acid (22:5) | Per 0,50 % | 1,36 (0,56; 3,29) |  |  |
|  |  |  |  |  |  | Docosahexaenoic acid (22:6) | Per 3,75 % | 0,76 (0,27; 2,09) |  |  |
|  |  |  |  |  |  | EPA+DPA+DHA | Per 7,38 % | 0,76 (0,27; 2,09) |  |  |
|  | Netherlands, AOC | 4,8 years | PPL, GC | 779 (38), m/f, 68,9 years |  | α-Linolenic acid (α-18:3) | Per 0,21 % | 1,64 (0,53; 5,07) |  |  |
|  |  |  |  |  |  | Eicosapentaenoic acid (20:5) | Per 1,54 % | 0,99 (0,48; 2,02) |  |  |
|  |  |  |  |  |  | Docosapentaenoic acid (22:5) | Per 0,60 % | 0,79 (0,26; 2,34) |  |  |
|  |  |  |  |  |  | Docosahexaenoic acid (22:6) | Per 3,36 % | 0,55 (0,21; 1,45) |  |  |
|  |  |  |  |  |  | EPA+DPA+DHA | Per 4,86 % | 0,69 (0,25; 1,89) |  |  |
|  | USA, ARIC | 9,0 years | PPL, GC | 3273 (512), m/f, 54,4 years |  | α-Linolenic acid (α-18:3) | Per 0,12 % | 0,93 (0,65; 1,32) |  |  |
|  |  |  |  |  |  | Eicosapentaenoic acid (20:5) | Per 0,52 % | 1,28 (1,01; 1,62) |  |  |
|  |  |  |  |  |  | Docosapentaenoic acid (22:5) | Per 0,43 % | 0,95 (0,66; 1,38) |  |  |
|  |  |  |  |  |  | Docosahexaenoic acid (22:6) | Per 2,04 % | 1,04 (0,76; 1,42) |  |  |
|  |  |  |  |  |  | EPA+DPA+DHA | Per 2,43 % | 1,12 (0,82; 1,53) |  |  |
|  | Taiwan, CCCC | 10,4 years | Total plasma, GC | 1443 (651), m/f, 60,1 years |  | α-Linolenic acid (α-18:3) | Per 0,48 % | 0,96 (0,82; 1,12) |  |  |
|  |  |  |  |  |  | Eicosapentaenoic acid (20:5) | Per 0,41 % | 0,97 (0,88; 1,07) |  |  |
|  |  |  |  |  |  | Docosahexaenoic acid (22:6) | Per 1,56 % | 0,92 (0,74; 1,15) |  |  |
|  |  |  |  |  |  | EPA+DPA+DHA | Per 1,87 % | 0,93 (0,77; 1,12) |  |  |
|  | USA, CHS | 18,0 years | PPL, GC | 3007 (291), m/f, 75,1 years |  | Eicosapentaenoic acid (20:5) | Per 0,65 % | 0,92 (0,70; 1,21) |  |  |
|  |  |  |  |  |  | Docosapentaenoic acid (22:5) | Per 0,41 % | 1,02 (0,76; 1,37) |  |  |
|  |  |  |  |  |  | Docosahexaenoic acid (22:6) | Per 2,42 % | 0,84 (0,60; 1,17) |  |  |
|  |  |  |  |  |  | EPA+DPA+DHA | Per 2,96 % | 0,85 (0,60; 1,21) |  |  |
|  | Europe, EPIC-InterAct | 17,5 years | PPL, GC | 27296 (1213), m/f, 52,3 years  **340234 (12403)* |  | α-Linolenic acid (α-18:3) | Per 0,35 % | 0,92 (0,85; 0,99) |  |  |
|  |  |  |  |  |  | Eicosapentaenoic acid (20:5) | Per 1,59 % | 0,88 (0,82; 0,95) |  |  |
|  |  |  |  |  |  | Docosapentaenoic acid (22:5) | Per 0,53 % | 0,67 (0,58; 0,77) |  |  |
|  |  |  |  |  |  | Docosahexaenoic acid (22:6) | Per 3,12 % | 0,66 (0,54; 0,80) |  |  |
|  |  |  |  |  |  | EPA+DPA+DHA | Per 4,47 % | 0,67 (0,56; 0,81) |  |  |
|  | Finland, FDPS | 16,0 years | Total plasma, GC | 396 (161), m/f, 55,5 years |  | α-Linolenic acid (α-18:3) | Per 0,58 % | 1,40 (0,92; 2,14) |  |  |
|  |  |  |  |  |  | Eicosapentaenoic acid (20:5) | Per 2,04 % | 0,69 (0,44; 1,08) |  |  |
|  |  |  |  |  |  | Docosapentaenoic acid (22:5) | Per 0,40 % | 0,55 (0,33; 0,91) |  |  |
|  |  |  |  |  |  | Docosahexaenoic acid (22:6) | Per 2,75 % | 0,57 (0,34; 0,96) |  |  |
|  |  |  |  |  |  | EPA+DPA+DHA | Per 4,93 % | 0,59 (0,35; 0,99) |  |  |
|  | USA, FHS | 9,0 years | RBC, GC | 1872 (95), m/f, 64,5 years |  | α-Linolenic acid (α-18:3) | Per 0,16 % | 1,08 (0,78; 1,49) |  |  |
|  |  |  |  |  |  | Eicosapentaenoic acid (20:5) | Per 0,77 % | 0,91 (0,58; 1,43) |  |  |
|  |  |  |  |  |  | Docosapentaenoic acid (22:5) | Per 1,03 % | 0,91 (0,53; 1,58) |  |  |
|  |  |  |  |  |  | Docosahexaenoic acid (22:6) | Per 3,44 % | 0,72 (0,38; 1,37) |  |  |
|  |  |  |  |  |  | EPA+DPA+DHA | Per 4,52 % | 0,76 (0,42; 1,40) |  |  |
|  | Japan, Hisayama | 7,0 years | Total plasma, GC | 2172 (222), m/f, 58,5 years |  | α-Linolenic acid (α-18:3) | Per 0,48 % | 1,09 (0,81; 1,47) |  |  |
|  |  |  |  |  |  | Eicosapentaenoic acid (20:5) | Per 2,88 % | 0,66 (0,47; 0,92) |  |  |
|  |  |  |  |  |  | Docosapentaenoic acid (22:5) | Per 0,43 % | 0,65 (0,44; 0,96) |  |  |
|  |  |  |  |  |  | Docosahexaenoic acid (22:6) | Per 3,02 % | 0,62 (0,43; 0,89) |  |  |
|  |  |  |  |  |  | EPA+DPA+DHA | Per 5,96 % | 0,61 (0,43; 0,88) |  |  |
|  | USA, HPFS | 20,2 years | RBC, GC | 1491 (108), m, 64,5 years |  | α-Linolenic acid (α-18:3) | Per 0,17 % | 1,27 (1,06; 1,52) |  |  |
|  |  |  |  |  |  | Eicosapentaenoic acid (20:5) | Per 0,57 % | 0,94 (0,59; 1,51) |  |  |
|  |  |  |  |  |  | Docosapentaenoic acid (22:5) | Per 1,00 % | 0,78 (0,44; 1,39) |  |  |
|  |  |  |  |  |  | Docosahexaenoic acid (22:6) | Per 2,96 % | 1,11 (0,66; 1,87) |  |  |
|  |  |  |  |  |  | EPA+DPA+DHA | Per 3,98 % | 1,01 (0,59; 1,76) |  |  |
|  |  |  | Total plasma, GC | 1443 (105), m, 64,5 years |  | α-Linolenic acid (α-18:3) | Per 0,53% | 1,08 (0,72; 1,61) |  |  |
|  |  |  |  |  |  | Eicosapentaenoic acid (20:5) | Per 0,79% | 0,83 (0,52; 1,33) |  |  |
|  |  |  |  |  |  | Docosapentaenoic acid (22:5) | Per 0,35% | 0,65 (0,38; 1,11) |  |  |
|  |  |  |  |  |  | EPA+DPA+DHA | Per 2,58% | 1,01 (0,59; 1,76) |  |  |
|  | Finland, KIHD | 29,5 years | Total plasma, GC | 3389 (595), m/f, 55,5 years |  | α-Linolenic acid (α-18:3) | Per 0,65 % | 1,03 (0,84; 1,26) |  |  |
|  |  |  |  |  |  | Eicosapentaenoic acid (20:5) | Per 1,82 % | 0,87 (0,73; 1,04) |  |  |
|  |  |  |  |  |  | Docosapentaenoic acid (22:5) | Per 0,37 % | 0,76 (0,61; 0,96) |  |  |
|  |  |  |  |  |  | Docosahexaenoic acid (22:6) | Per 1,94 % | 0,75 (0,61; 0,92) |  |  |
|  |  |  |  |  |  | EPA+DPA+DHA | Per 3,72 % | 0,80 (0,65; 0,97) |  |  |
|  | Australia, MCCS | 9,9 years | PPL, GC | 4034 (335), m/f, 50 years  **21523 (929)* |  | α-Linolenic acid (α-18:3) | Per 0,18 % | 1,03 (0,80; 1,33) |  |  |
|  |  |  |  |  |  | Eicosapentaenoic acid (20:5) | Per 1,06 % | 0,73 (0,53; 1,00) |  |  |
|  |  |  |  |  |  | Docosapentaenoic acid (22:5) | Per 0,61 % | 1,16 (0,80; 1,67) |  |  |
|  |  |  |  |  |  | Docosahexaenoic acid (22:6) | Per 2,66 % | 0,90 (0,66; 1,24) |  |  |
|  |  |  |  |  |  | EPA+DPA+DHA | Per 3,30 % | 0,83 (0,61; 1,14) |  |  |
|  | USA, MESA | 11,2 years | PPL, GC | 2099 (285), m/f, 61 years |  | α-Linolenic acid (α-18:3) | Per 0,16 % | 0,66 (0,48; 0,90) |  |  |
|  |  |  |  |  |  | Eicosapentaenoic acid (20:5) | Per 1,42 % | 1,29 (0,88; 1,88) |  |  |
|  |  |  |  |  |  | Docosapentaenoic acid (22:5) | Per 0,53 % | 0,87 (0,64; 1,20) |  |  |
|  |  |  |  |  |  | Docosahexaenoic acid (22:6) | Per 3,90 % | 0,72 (0,45; 1,14) |  |  |
|  |  |  |  |  |  | EPA+DPA+DHA | Per 5,32 % | 0,75 (0,49; 1,14) |  |  |
|  | Finland, METSIM | 7,9 years | RBC, GC | 1302 (101), m, 55 years |  | α-Linolenic acid (α-18:3) | Per 0,13 % | 1,04 (0,79; 1,37) |  |  |
|  |  |  |  |  |  | Eicosapentaenoic acid (20:5) | Per 1,37 % | 0,52 (0,29; 0,93) |  |  |
|  |  |  |  |  |  | Docosapentaenoic acid (22:5) | Per 0,98 % | 0,70 (0,41; 1,22) |  |  |
|  |  |  |  |  |  | Docosahexaenoic acid (22:6) | Per 2,87 % | 0,82 (0,47; 1,45) |  |  |
|  |  |  |  |  |  | EPA+DPA+DHA | Per 4,49 % | 0,64 (0,36; 1,15) |  |  |
|  |  |  | CE, GC |  |  | α-Linolenic acid (α-18:3) | Per 0,67 % | 1,04 (0361; 1,78) |  |  |
|  |  |  |  |  |  | Eicosapentaenoic acid (20:5) | Per 1,37 % | 0,52 (0,29; 0,93) |  |  |
|  |  |  |  |  |  | Docosahexaenoic acid (22:6) | Per 2,87 % | 0,82 (0,47; 1,45) |  |  |
|  |  |  |  |  |  | EPA+DPA+DHA | Per 4,49 % | 0,64 (0,36; 1,15) |  |  |
|  |  |  | TG, GC |  |  | α-Linolenic acid (α-18:3) | Per 1,71 % | 0,92 (0,46; 1,83) |  |  |
|  |  |  |  |  |  | Eicosapentaenoic acid (20:5) | Per 1,16 % | 1,05 (0,79; 1,39) |  |  |
|  |  |  |  |  |  | Docosapentaenoic acid (22:5) | Per 0,74 % | 0,62 (0,31; 1,24) |  |  |
|  |  |  |  |  |  | Docosahexaenoic acid (22:6) | Per 2,97 % | 1,00 (0,69; 1,46) |  |  |
|  |  |  |  |  |  | EPA+DPA+DHA | Per 4,63 % | 0,98 (0,66; 1,48) |  |  |
|  | USA, NHS | 24,8 years | RBC, GC | 1446 (149), f, 60,4 years |  | α-Linolenic acid (α-18:3) | Per 0,12 % | 1,37 (0,90; 2,07) |  |  |
|  |  |  |  |  |  | Eicosapentaenoic acid (20:5) | Per 0,39 % | 1,27 (0,96; 1,68) |  |  |
|  |  |  |  |  |  | Docosapentaenoic acid (22:5) | Per 0,87 % | 0,92 (0,54; 1,56) |  |  |
|  |  |  |  |  |  | Docosahexaenoic acid (22:6) | Per 2,34 % | 0,65 (0,38; 1,13) |  |  |
|  |  |  |  |  |  | EPA+DPA+DHA | Per 3,12 % | 0,76 (0,44; 1,32) |  |  |
|  |  |  | Total plasma, GC | 1524 (150), f, 60,4 years |  | α-Linolenic acid (α-18:3) | Per 0,38 % | 1,51 (1,05; 2,18) |  |  |
|  |  |  |  |  |  | Eicosapentaenoic acid (20:5) | Per 0,51 % | 1,21 (0,92; 1,60) |  |  |
|  |  |  |  |  |  | Docosapentaenoic acid (22:5) | Per 0,27 % | 1,17 (1,01; 1,37) |  |  |
|  |  |  |  |  |  | Docosahexaenoic acid (22:6) | Per 1,30 % | 0,93 (0,59; 1,47) |  |  |
|  |  |  |  |  |  | EPA+DPA+DHA | Per 1,83 % | 1,15 (0,78; 1,70) |  |  |
|  | Sweden, PIVUS | 10,9 years | PPL, GC | 872 (69), m/f, 70,2 years |  | α-Linolenic acid (α-18:3) | Per 0,24 % | 0,91 (0,50; 1,66) |  |  |
|  |  |  |  |  |  | Eicosapentaenoic acid (20:5) | Per 2,35 % | 0,72 (0,39; 1,32) |  |  |
|  |  |  |  |  |  | Docosapentaenoic acid (22:5) | Per 0,55 % | 0,63 (0,35; 1,12) |  |  |
|  |  |  |  |  |  | Docosahexaenoic acid (22:6) | Per 3,07 % | 1,15 (0,60; 2,20) |  |  |
|  |  |  |  |  |  | EPA+DPA+DHA | Per 5,24 % | 0,85 (0,44; 1,63) |  |  |
|  |  |  | CE, GC | 834 (67), m/f, 70,2 years |  | α-Linolenic acid (α-18:3) | Per 0,59 % | 0,81 (0,40; 1,64) |  |  |
|  |  |  |  |  |  | Eicosapentaenoic acid (20:5) | Per 2,52 % | 0,83 (0,45; 1,52) |  |  |
|  |  |  |  |  |  | Docosahexaenoic acid (22:6) | Per 0,67 % | 1,13 (0,66; 1,93) |  |  |
|  |  |  |  |  |  | EPA+DPA+DHA | Per 3,05 % | 0,87 (0,48; 1,58) |  |  |
|  | Sweden, ULSAM-50 | 42,3 years | CE, GC | 1899 (335), m, 49,7 years |  | α-Linolenic acid (α-18:3) | Per 0,40 % | 0,89 (0,67; 1,19) |  |  |
|  |  |  |  |  |  | Eicosapentaenoic acid (20:5) | Per 1,46 % | 1,00 (0,73; 1,35) |  |  |
|  |  |  |  |  |  | Docosahexaenoic acid (22:6) | Per 0,50 % | 0,92 (0,68; 1,26) |  |  |
|  |  |  |  |  |  | EPA+DPA+DHA | Per 1,85 % | 0,97 (0,71; 1,33) |  |  |
|  | Sweden, ULSAM-70 | 21,5 years | AT, GC | 738 (99), m, 71 years |  | α-Linolenic acid (α-18:3) | Per 0,55 % | 0,65 (0,36; 1,16) |  |  |
|  |  |  |  |  |  | Eicosapentaenoic acid (20:5) | Per 0,12 % | 1,12 (0,59; 2,13) |  |  |
|  |  |  |  |  |  | Docosapentaenoic acid (22:5) | Per 0,21 % | 1,69 (1,03; 2,77) |  |  |
|  |  |  |  |  |  | Docosahexaenoic acid (22:6) | Per 0,33 % | 1,53 (0,99; 2,37) |  |  |
|  |  |  |  |  |  | EPA+DPA+DHA | Per 0,66 % | 1,55 (0,96; 2,48) |  |  |
|  | USA, WHIMS | 14,1 years | RBC, GC | 5668 (490), f, 70,1 years |  | α-Linolenic acid (α-18:3) | Per 0,16 % | 1,10 (0,85; 1,42) |  |  |
|  |  |  |  |  |  | Eicosapentaenoic acid (20:5) | Per 0,85 % | 0,84 (0,64; 1,11) |  |  |
|  |  |  |  |  |  | Docosapentaenoic acid (22:5) | Per 1,08 % | 0,66 (0,49; 0,89) |  |  |
|  |  |  |  |  |  | Docosahexaenoic acid (22:6) | Per 3,51 % | 0,85 (0,64; 1,14) |  |  |
|  |  |  |  |  |  | EPA+DPA+DHA | Per 4,42 % | 0,75 (0,57; 0,98) |  |  |
| Virtanen, 2014, Finland, KIHD (334) | | 19,3 years | Total serum, GC | 2212 (422), m, 51 years | Self-reported physician-set diagnosis of T2D and/or FBG ≥126 mg/dl, OGTT ≥ 200 mg/dL, record linkage to the national hospital discharge registry and to the Social Insurance Institution of Finland register for reimbursement of medicine | EPA+DPA+DHA | 3,26 %  3,98 %  4,84 %  5,82 % | 1,00  0,71 [0,54; 0,93]  0,78 [0,60; 1,02]  0,67 [0,51; 0,87] | Age, BMI, smoking, physical activity, alcohol, family history of diabetes, education, year of examination, Linoleic acid (18:2) | Moderate |
|  |  |  |  |  |  | α-Linolenic acid (α-18:3) | 0,51 %  0,64 %  0,79 %  0,95 % | 1,00  0,87 [0,67; 1,14]  0,88 [0,67; 1,17]  0,82 [0,62; 1,10] |  |  |
|  |  |  |  |  |  | Docosapentaenoic acid (22:5) | 0,45 %  0,51 %  0,58 %  0,64 % | 1,00  0,77 [0,59; 1,01]  0,73 [0,56; 0,96]  0,72 [0,55; 0,94] |  |  |
|  |  |  |  |  |  | Docosahexaenoic acid (22:6) | 1,76 %  2,15 %  2,60 %  3,09 % | 1,00  0,81 [0,62; 1,06]  0,71 [0,54; 0,94]  0,66 [0,51; 0,87] |  |  |
| Wu, 2017 (335) | Island, AGESR | 7,8 years | PPL, GC | 753 (28), m/f, 76 years | Incident T2D was defined by whichever of the following criteria were met first: a FBG concentration of 1≥26 mg/dL or higher, a glucose concentration of ≥200 mg/dL or higher as measured by a 2-h post-OGTT, new use of insulin or oral hypoglycaemic medication, fasting or non-fasting HBA1c concentrations of 6∙5% or more, or by self-reported physician diagnosis in some cohorts, | Linoleic acid (18:2) | 6,8 % | 0,27 (0,08; 0,91) | Age, sex, race, site of patient recruitment if applicable, BMI, education, smoking, physical activity, alcohol intake, prevalent coronary heart disease, treatment for hypertension, treatment for hypercholesterolaemia, and biomarker omega-3 PUFA concentrations | Moderate |
|  |  |  |  |  |  | Arachidonic acid (20:4) | 3,8 % | 1,51 (0,64; 3,56) |  |  |
|  | Netherlands, AOC | 4,8 years | CE, GC | 2888 (154), m/f, 69 years |  | Linoleic acid (18:2) | 12,5 % s | 0,58 (0,37; 0,93) |  |  |
|  |  |  |  |  |  | Arachidonic acid (20:4) | 5,1 % s | 1,12 (0,70; 1,80) |  |  |
|  | UK, EPIC-Norfolk | 12,1 years | RBC, GC | 383 (199), m/f, 64 years  **25639 (892)* |  | Linoleic acid (18:2) | 3,4% s | 0,53 (0,31; 1,00) |  |  |
|  |  |  |  |  |  | Arachidonic acid (20:4) | 3,0 % | 1,00 (0,62; 1,63) |  |  |
|  | USA, ARIC | 9,0 years | PPL, GC | 3493 (304), m/f, 54 years |  | Linoleic acid (18:2) | 6,7 % | 0,65 (0,49; 0,88) |  |  |
|  |  |  |  |  |  | Arachidonic acid (20:4) | 5,0 % | 1,30 (0,96; 1,76) |  |  |
|  | Taiwan, CCCC | 8,1 years | Total plasma, GC | 616 (128), m/f, 59 years |  | Linoleic acid (18:2) | 11,4 % | 1,03 80,62; 1,69) |  |  |
|  |  |  |  |  |  | Arachidonic acid (20:4) | 2,5 % | 1,66 (0,96; 2,87) |  |  |
|  | USA, CHS | 18 years | PPL, GC | 3179 (284), m/f, 72 years |  | Linoleic acid (18:2) | 6,2 % | 0,66 (0,46; 0,95) |  |  |
|  |  |  |  |  |  | Arachidonic acid (20:4) | 4,9 % | 1,42 (1,04; 1,95) |  |  |
|  | USA, FHS | 9,0 years | RBC, GC | 1913 (98), m/f, 64 years |  | Linoleic acid (18:2) | 4,1 % | 0,48 (0,26; 0,90) |  |  |
|  |  |  |  |  |  | Arachidonic acid (20:4) | 3,8 % | 0,93 (0,52; 1,68) |  |  |
|  | USA, HPFS | 17,6 years | RBC, GC | 1545 (113), m, 65 years |  | Linoleic acid (18:2) | 5,1 % | 0,81 (0,58; 1,15) |  |  |
|  |  |  |  |  |  | Arachidonic acid (20:4) | 4,4 % | 0,45 (0,30; 0,69) |  |  |
|  |  |  | Total plasma, GC |  |  | Linoleic acid (18:2) | 11,3 % | 0,42 (0,26; 0,69) |  |  |
|  |  |  |  |  |  | Arachidonic acid (20:4) | 4,6 % | 0,38 (0,22; 0,68) |  |  |
|  | USA, IRAS | 5,0 | Total plasma, GC | 719 (146), m/f, 55 |  | Linoleic acid (18:2) | 11,0 % | 0,56 (0,33; 0,95) |  |  |
|  |  |  |  |  |  | Arachidonic acid (20:4) | 4,7 % | 0,69 (0,38; 1,28) |  |  |
|  | Finland, KIHD | 26,8 years | Total serum, GC | 3145 (595), m, 56 years |  | Linoleic acid (18:2) | 10,9 % | 0,54 (0,43; 0,68) |  |  |
|  |  |  |  |  |  | Arachidonic acid (20:4) | 2,9 % | 0,70 (0,56; 0,89) |  |  |
|  | Australia, MCCS | 9,9 years | PPL, GC | 4046 (336), m/f, 55 years  **21523 (929)* |  | Linoleic acid (18:2) | 7,5 % | 0,36 (0,24; 0,55) |  |  |
|  |  |  |  |  |  | Arachidonic acid (20:4) | 4,5 % | 1,01 (0,73; 1,38) |  |  |
|  | USA. MESA | 11,2 years | PPL, GC | 2230 (297), m/f, 61 years |  | Linoleic acid (18:2) | 8,4 % | 0,81 (0,58; 1,14) |  |  |
|  |  |  |  |  |  | Arachidonic acid (20:4) | 6,6 % | 1,25 (0,89; 1,75) |  |  |
|  | Finland, METSIM | 7,9 years | RBC, GC | 1301 (71), m, 55 years |  | Linoleic acid (18:2) | 2,7 % | 0,29 (0,14; 0,59) |  |  |
|  |  |  |  |  |  | Arachidonic acid (20:4) | 2,8 % | 2,17 (1,03; 4,57) |  |  |
|  |  |  | CE, GC |  |  | Linoleic acid (18:2) | 12,3 % | 0,50 (0,28; 0,91) |  |  |
|  | USA, NHS | 22,8 years | RBC, GC | 1500 (154), f, 60 years |  | Linoleic acid (18:2) | 5,6 % | 1,15 (0,77; 1,70) |  |  |
|  |  |  |  |  |  | Arachidonic acid (20:4) | 4,9 % | 0,78 (0,50; 1,24) |  |  |
|  |  |  | Total plasma, GC | 1595 (159), f, 60 years |  | Linoleic acid (18:2) | 12,1 % | 0,44 (0,30; 0,64) |  |  |
|  |  |  |  |  |  | Arachidonic acid (20:4) | 4,7 % | 0,67 (0,41; 1,10) |  |  |
|  | Sweden, PIVUS | 10,9 years | PPL, GC | 861 (69), m/f, 70 years |  | Linoleic acid (18:2) | 6,6 % | 0,70 (0,26; 1,86) |  |  |
|  |  |  |  |  |  | Arachidonic acid (20:4) | 3,3 % | 1,25 (0,71; 2,19) |  |  |
|  |  |  | CE, GC | 822 (67), m/f, 70 years |  | Linoleic acid (18:2) | 10,6 % | 0,80 (0,34; 1,87) |  |  |
|  |  |  |  |  |  | Arachidonic acid (20:4) | 3,1 % | 1,55 (0,89; 2,68) |  |  |
|  | France, 3C | 13,0 years | RBC, GC | 574 (36), m/f, 74 years |  | Linoleic acid (18:2) | 3,8 % | 0,51 (0,19; 1,37) |  |  |
|  |  |  |  |  |  | Arachidonic acid (20:4) | 3,1 % | 1,21 (0,51; 2,86) |  |  |
|  |  |  | Total plasma, GC | 1220 (83), m/f, 74 years |  | Linoleic acid (18:2) | 12,9 % | 0,57 (0,34; 0,94) |  |  |
|  |  |  |  |  |  | Arachidonic acid (20:4) | 4,5 % | 0,81 (0,45; 1,45) |  |  |
|  | Sweden, ULSAM-50 | 42,3 years | CE, GC | 1891 (246), m, 50 years |  | Linoleic acid (18:2) | 13,0 % | 0,57 (0,41; 0,79) |  |  |
|  |  |  |  |  |  | Arachidonic acid (20:4) | 2,4 % | 0,95 (0,70; 1,29) |  |  |
|  | Sweden, ULSAM-70 | 21,5 years | AT, GC | 738 (99), m, 71 years |  | Linoleic acid (18:2) | 6,3 % | 0,82 (0,49; 1,35) |  |  |
|  |  |  |  |  |  | Arachidonic acid (20:4) | 0,3 % | 1,56 (0,84; 2,89) |  |  |
|  | USA, WHIMS | 14,1 years | RBC, GC | 5799 (502), f, 70 years |  | Linoleic acid (18:2) | 4,5 % | 0,89 (0,67; 1,17) |  |  |
|  |  |  |  |  |  | Arachidonic acid (20:4) | 5,5 % | 0,79 (0,59; 1,05) |  |  |
| Yakoob, 2016 (336) | USA, NHS | 15,2 years | RBC, GLC | 1864 (184), f, 60,4 years | T2D diagnosis based on self-report of physician-diagnosed diabetes and the calendar year of diagnosis. Self-reports were validated by a supplementary questionnaire if they met ≥1 of the National Diabetes Data Group criteria: (1) classic symptoms plus FBG ≥140 mg/dL or random glucose ≥200 mg/dL; (2) at least two separate elevated plasma glucose levels (fasting ≥140, random ≥200 mg/dL, or 2-hour challenge ≥200 mg/dL); or (3) medical prescription of oral hypoglycaemic agents or insulin. The Diagnostic criteria were modified after Jun 1996; FBG of ≥ 126 mg/dL. | Myristic acid (14:0) | 0,11 %  0,20 %  0,31 %  0,64 % | 1,00  1,18 [0,74; 1,87]  0,76 [0,45; 1,29]  1,21 [0,61; 2,39] | Age, race, smoking status, physical activity, alcohol, family history of diabetes, parental history of myocardial infarction, hypercholesterolemia, hypertension, menopausal status in NHS, postmenopausal hormone use in NHS, and consumption of fish, processed meats, unprocessed meats, fruits, vegetables, whole grains, coffee, sugar-sweetened beverages, glycaemic load, dietary calcium, polyunsaturated fat, total energy, and plasma trams-Oleic acid (*t*-18-1n-9), *trans*-Linoleic acid (*t-*18:2), Palmitic acid (16:0), and Stearic acid (18:0). | High |
|  |  |  |  |  |  | Pentadecanoic acid (15:0) | 0,08 %  0,11 %  0,14 %  0,18 % | 1,00  0,73 [0,46; 1,16]  0,93 [0,60; 1,43]  0,65 [0,39; 1,09] |  |  |
|  |  |  |  |  |  | Margaric acid (17:0) | 0,31 %  0,37 %  0,42 %  0,59 % | 1,00  0,66 [0,44; 0,98]  0,30 [0,18; 0,49]  0,37 [0,20; 0,66] |  |  |
|  |  |  |  |  |  | *Trans-*Palmitoleic acid (*t-*16:1n–7) | 0,11 %  0,14 %  0,17 %  0,22 % | 1,00  1,03 [0,69; 1,54]  0,65 [0,41; 1,02]  0,60 [0,36; 1,02] |  |  |
|  |  |  | Total plasma, GLC |  |  | Myristic acid (14:0) | 0,24 %  0,44 %  0,64 %  0,98 % | 1,00  1,55 [0,97; 2,49]  0,95 [0,57; 1,57]  0,93 [0,55; 1,57] |  |  |
|  |  |  |  |  |  | Pentadecanoic acid (15:0) | 0,11 %  0,14 %  0,17 %  0,22 % | 1,00  1,26 [0,80; 2,00]  0,89 [0,56; 1,43]  0,60 [0,36; 1,01] |  |  |
|  |  |  |  |  |  | Margaric acid (17:0) | 0,26 %  0,30 %  0,33 %  0,39 % | 1,00  0,79 [0,53; 1,19]  0,75 [0,49; 1,14]  0,50 [0,31; 0,81] |  |  |
|  |  |  |  |  |  | *Trans-*Palmitoleic acid (*t-*16:1n–7) | 0,13 %  0,17 %  0,21 %  0,28 % | 1,00  0,79 [0,51; 1,22]  0,69 [0,46; 1,05]  0,48 [0,30; 0,76] |  |  |
|  | USA, HPFS |  | RBC, GLC | 1469 (93), m, 64,6 years |  | Myristic acid (14:0) | 0,12 %  0,21 %  0,31 %  0,53 % | 1,00  1,49 [0,70; 3,14]  2,29 [1,10; 4,78]  3,43 [1,51; 7,77] |  |  |
|  |  |  |  |  |  | *Trans-*Palmitoleic acid (*t*-16:1n–7) | 0,09 %  0,12 %  0,14 %  0,18 % | 1,00  1,31 [0,72; 2,39]  0,82 [0,42; 1,59]  1,21 [0,61; 2,39] |  |  |
|  |  |  | Total plasma, GLC |  |  | Myristic acid (14:0) | 0,25 %  0,40 %  0,57 %  0,90 % | 1,00  1,98 [0,90; 4,33]  1,85 [0,83; 4,15]  1,50 [0,56; 3,98] |  |  |
|  |  |  |  |  |  | Pentadecanoic acid (15:0) | 0,1 %  0,13 %  0,15 %  0,2 % | 1,00  0,63 [0,31; 1,30]  1,12 [0,59; 2,12]  0,49 [0,23; 1,04] |  |  |
|  |  |  |  |  |  | Margaric acid (17:0) | 0,24 %  0,29 %  0,32 %  0,38 % | 1,00  0,80 [0,43; 1,47]  0,48 [0,25; 0,94]  0,69 [0,38; 1,26] |  |  |
| Yary, 2016, Finland, KIHD (337) | | 19,3 years | Total serum, GC | 2189 (417), m, 51 years | T2D was defined as self-reported diagnosis, physician-diagnosed T2D and/or FBG >7.0 126mg/dL or 2-h OGTT or plasma glucose >200 mg/dL at re-examination, and linkage to the national health registry and to the Social Insurance Institution. | Total n-6 PUFA | 20,97 %  30,25 %  33,33 %  40,68 % | 1,00  0,68 [0,53; 0,87]  0,58 [0,44; 0,77]  0,54 [0,41; 0,73] | Age, BMI, smoking, physical activity, family history of diabetes, education, alcohol intake, energy intake, serum long  chain n–3 PUFA concentrations | Moderate |
|  |  |  |  |  |  | Linoleic acid (18:2) | 16,87 %  25,17 %  28,15 %  35,52 % | 1,00  0,71 [0,55; 0,92]  0,68 [0,52; 0,89]  0,52 [0,39; 0,70] |  |  |
|  |  |  |  |  |  | ɣ-Linolenic acid (ɣ-18:3) | 0,13 %  0,25 %  0,32 %  0,69 % | 1,00  0,85 [0,63; 1,13]  1,03 [0,79;1,36]  1,28 [0,98; 1,68] |  |  |
|  |  |  |  |  |  | Dihomo-ɣ-Linolenic acid (ɣ-20:3) | 0,86 %  1,25 %  1,42 %  4,65 % | 1,00  1,12 [0,84; 1,50]  1,06 [0,79; 1,42]  1,38 [1,04; 1,84] |  |  |
|  |  |  |  |  |  | Arachidonic acid (20:4) | 2,73 %  4,42 %  5,07 %  7,33 % | 1,00  0,76 [0,58; 0,99]  0,78 [0,60; 1,03]  0,62 [0,46; 0,85] |  |  |
| Zheng, 2019, China, GNHS (338) | | 5,6 years | RBC, GC | 2671 (213), m/f, 57 years | Incident T2D cases defined by FBG ≥126 mg/dL or HbA1c ≥ 6.5% or currently under medical treatment for diabetes at either of the two follow-up visits, according to the American Diabetes Association criteria for the diagnosis of diabetes. | DHA + DPA + EPA | 4,78 %  6,45 %  7,46 %  8,72 % | 1,00  0,74 [0,49; 1,11]  0,89 [0,60; 1,32]  0,96 [0,63; 1,45] | Age, sex, BMI, and ratio of waist to hip circumference, physical activity, education, alcohol drinking, smoking, household income, family history of diabetes, total energy intake, and intake of dairy products, red and processed meat, fruits and vegetables, fasting serum glucose and RBC total n-6 PUFA | Moderate |
|  |  |  |  |  |  | α-Linoleic acid (α-18:3) | 0,06 %  0,07 %  0,09 %  0,12 % | 1,00  1,00 [0,67; 1,48]  1,17 [0,80; 1,72]  1,07 [0,72; 1,60] |  |  |
|  |  |  |  |  |  | Docosahexaenoic acid (22:6) | 3,03 %  4,18 %  4,96 %  5,92 % | 1,00  1,15 [0,77; 1,73]  1,05 [0,68; 1,61]  1,52 [0,99 2,34] |  |  |
|  |  |  |  |  |  | Docosapentaenoic acid (22:5) | 1,11 %  1,45 %  1,65 %  1,90 % | 1,00  0,68 [0,47; 1;00]  0,77 [0,52; 1,15]  0,63 [0,41; 0,95] |  |  |
|  |  |  |  |  |  | Eicosapentaenoic acid (20:5) | 0,26 %  0,44 %  0,70 %  1,61 % | 1,00  1,08 [0,74; 1,60]  1,03 [0,70; 1,51]  0,57 [0,38; 0,86] |  |  |
| Zhuang, 2022, UK, UK Biobank (339) | | 11,6 years | Total plasma, high-throughput NMR based metabolic biomarker profiling platform | 95854 (3052), m/f, 55,7 years | T2D status was identified using the UK Biobank algorithms for the diagnosis of diabetes, which  has been validated in a subset of the participants with 96% accuracy. Individuals with T2D were ascertained using cumulative hospital inpatient records containing data on admissions and diagnoses  from the Hospital Episode Statistics | Total SFA | 31,9 %  33,3 %  34,4 %  36,1 % | 1,00  1,20 [1,05; 1,38]  1,21 [1,04; 1,41]  1,72 [1,46; 2,02] | Age, sex, race, BMI, education, Townsend deprivation index (quartiles), household income, smoking, alcohol intake, physical activity, history of hypertension, history of high cholesterol, family history of diabetes, vitamin supplement use, mineral supplement use, aspirin use, remaining plasma fatty acids (SFA, MUFA, PUFA, n-6 PUFA, and n-3 PUFA) | Moderate |
|  |  |  |  |  |  | Total MUFA | 20,5 %  22,3 %  23,9 %  26,5 % | 1,00  1,16 [0,97; 1,39]  1,33 [1,08; 1,64]  1,87 [1,48; 2,37] |  |  |
|  |  |  |  |  |  | Total PUFA | 38,3 %  42,0 %  44,2 %  46,5 % | 1,00  0,86 [0,75; 0,98]  0,79 [0,65; 0,96]  0,67 [0,51; 0,89] |  |  |
|  |  |  |  |  |  | Total n-6 PUFA | 34,1 %  37,6 %  39,7 %  42,0 % | 1,00  0,81 [0,72; 0,92]  0,76 [0,64; 0,90]  0,72 [0,57; 0,90] |  |  |
|  |  |  |  |  |  | Total n-3 PUFA | 2,8 %  3,8 %  4,6 %  6,0 % | 1,00  0,94 [0,86; 1,04]  0,77 [0,69; 0,86]  0,82 [0,72; 0,93] |  |  |
|  |  |  |  |  |  | Linoleic acid (18:2) | 25,6 %  28,5 %  30,5 %  33,0 % | 1,00  0,80 [0,72; 0,89]  0,66 [0,57; 0,77]  0,56 [0,46; 0,69] |  |  |
|  |  |  |  |  |  | Docosahexaenoic acid (22:6) | 1,3 %  1,7 %  2,1 %  2,7 % | 1,00  0,92 [0,83; 1,02]  0,86 [0,75; 0,98]  0,95 [0,79; 1,14] |  |  |
| † % of total fatty acid corresponds either to the increase in the relative concentration of a specific fatty acid in dose-response analyses, or to the concentration of specific fatty acids measured across exposure quantiles in high vs. low analyses.  †† In dose-response analyses, estimates represent the relative risk of T2D per increase in FA concentration as specified in the column “% of total fatty acids.” For high vs. low comparisons, estimates represent the relative risk of T2D for participants in higher exposure quantiles compared with participants in the lowest quantile.  3C STUDY, The 3-City Study; AGESR, Age, Gene/Environment Susceptibility - Reykjavik Study; AOC, Advances ovarian cancer; ARIC, Atherosclerosis Risk in Communities Studies; AT, Adipose tissue; BMI, Body Mass Index; CCCC, Chin-Shan Community Cardiovascular Cohort; CE, cholesteryl ester; CHS, Cardiovascular Health Study; CI, confidence interval; CKB, China Kadoorie Biobank; DHA, Docosahexaenoic acid; DPA, Docosapentaenoic acid; EPA, Eicosapentaenoic acid; EPIC, European Prospective Investigation into Cancer and Nutrition; f, female; FA, fatty acid; FBG, Fasting blood glucose; FDPS, The Finnish Diabetes Prevention Study; FHS, Framingham Heart Study-Cohort; FORCE, Fatty Acids and Outcomes Research Consortium; GC; Gas chromatography; GLC, Gas-liquid chromatography; GNHS, Guangzhou Nutrition and Health Study; HbA1c, Glycated haemoglobin; HCS, Hunter Community Study; HDL, high-density lipoprotein cholesterol; HPFS, Health Professionals Follow-up Study; HR, hazard ratio; ICD, international classification of diseases; IRAS, The Insulin Resistance Atherosclerosis Study; KIHD, Kuopio Ischaemic Heart Disease Risk Factor; LDL, Low-density lipoprotein cholesterol; m, male; MCCS, Melbourne Collaborative Cohort Study; MESA, Multi-Ethnic Study of Atherosclerosis; METSIM, Metabolic Syndrome in Men Study; MUFA, Monounsaturated fatty acids; NA, not available; NHS, Nurses’ Health Study; NMR, Nuclear magnetic resonance spectroscopy; OGTT, Oral glucose tolerance test; OR, odds ratio; PHS, Physicians’ Health Study; PIVUS, Prospective Investigation of the Vaculature in Uppsala; PPL, Plasma phospholipids; PUFA, Polyunsaturated fatty acids; RBC, Red blood cells; SFA, Saturated fatty acids; RR, relative risk; T2D, Type 2 diabetes; TFA, Total fatty acids; TG, triglycerides; UK, United Kingdom; ULSAM, Uppsala Longitudinal Study of Adult Men; USA, United States of America; VIP, The Västerbotten Intervention Programme; WHIMS, The Women’s Health Initiative Memory Study; WHO, World Health Organisation; WHR, waist-to-hip ratio.  *Number of participants and (number of cases) obtained from the whole cohort. If the included study was a case-cohort or nested case-control study, information on the ratio of number of cases and number of participants was necessary in order to obtain comparable ratios with other studies (prospective cohorts design) for the calculation of absolute effects in the Certainty of Evidence Assessment step. Following publications were used to obtain information on number of cases and number of participants in EPIC-InterAct: (31), EPIC-Norfolk: (3), EPIC-Potsdam: (325), CBK: (341), MCCS: (342), VIP: (343), HCS: since no information was available for this cohort, we implemented ratio calculated within another Australian cohort (MCCS). | | | | | | | | | | |

| Myristic acid | 14:0 |
| --- | --- |
| Pentadecanoic acid | 15:0 |
| Palmitic acid | 16:0 |
| Margaric acid | 17:0 |
| Stearic acid | 18:0 |
| Arachidic acid | 20:0 |
| Behenic acid | 22:0 |
| Lignoceric acid | 24:0 |
| Palmitoleic acid | 16:1n-7 |
| Palmitelaidic acid | 16:1n-9 |
| Oleic acid | 18:1n-9 |
| Eicosanoic acid | 20:1 |
| Nervonic acid | 24:1 |
| *Trans-*Vaccenic acid | *t-*18:1n-7 |
| *Trans-*Elaidic acid | *t-*18:1n-9 |
| *Trans-*Hypogeic acid | *t-*16:1n-9 |
| *Trans-*Palmitoleic acid | *t-*16:1n-7 |
| *Trans-*Linoleic aicd | *t-*18:2 |
| Linoleic acid | 18:2 |
| γ-Linolenic acid | γ-18:3 |
| Dihomo-γ-linolenic acid | γ-20:3 |
| Arachidonic acid | 20:4 |
| Docosatetraenoic acid | 22:4 |
| α-Linolenic acid | α-18:3 |
| Eicosapentaenoic acid | 20:5 |
| Docosapentaenoic acid | 22:5 |
| Docosahexaenoic acid | 22:6 |

**Supplemental Table 7.** List of the fatty acids included into dose-response meta-analyses on the association with the risk of type 2 diabetes.

**Supplemental Table 8.** Certainty of evidence of the meta-analyses of prospective cohort studies on the association between biomarkers of saturated fatty acids (SFAs) and risk of type 2 diabetes by using the GRADE tool.

| **Certainty assessment** | | | | | | | **N cases/ N participants for Absolute effects calculation*** | **Effect** | | **Certainty** |
| --- | --- | --- | --- | --- | --- | --- | --- | --- | --- | --- |
| **№ of studies** | **Study design** | **Risk of bias** | **Inconsistency** | **Indirectness** | **Imprecision** | **Other considerations** |  | **Relative (95% CI)** | **Absolute (95% CI)** |  |
| **Total SFAs (per 3% in total FAs) in all biospecimens** | | | | | | | | | | |
| 6 | non-randomised studies | serious^a^ | serious^b^ | not serious | serious^c^ | none | 15081/619302 (2.4%) | **RR 1.15** (0.86 to 1.54) | **4 more per 1.000** (from 3 fewer to 13 more) | ⨁◯◯◯ Very low^a,b,c^ |
| **Total SFAs (per 3% in total FAs) in Red blood cells** | | | | | | | | | | |
| 3 | non-randomised studies | serious^a^ | not serious | not serious | serious^c^ | none | 2925/89383 (3.3%) | **RR 1.10** (0.93 to 1.29) | **3 more per 1.000** (from 2 fewer to 9 more) | ⨁⨁◯◯ Low^a,c^ |
| **Total SFA (per 3% in total FAs) in Plasma phospholipids** | | | | | | | | | | |
| 2 | non-randomised studies | very serious^d^ | not serious | not serious | serious^e^ | none | 863/26941 (3.2%) | **RR 1.68** (1.24 to 2.27) | **22 more per 1.000** (from 8 more to 41 more) | ⨁◯◯◯ Very low^d,e^ |
| **Total SFAs (per 3% in total FAs) in Total plasma** | | | | | | | | | | |
| 2 | non-randomised studies | serious^a^ | very serious^f^ | not serious | serious^c^ | none | 12085/528617 (2.3%) | **RR 1.09** (0.63 to 1.89) | **2 more per 1.000** (from 8 fewer to 20 more) | ⨁◯◯◯ Very low^a,c,f^ |
| **Myristic acid, 14:0 (per 0.1% of total FAs) in all biospecimens** | | | | | | | | | | |
| 6 | non-randomised studies | very serious^d^ | not serious | not serious | serious^c^ | none | 2045/46518 (4.4%) | **RR 1.06** (0.96 to 1.17) | **3 more per 1.000** (from 2 fewer to 7 more) | ⨁◯◯◯ Very low^c,d^ |
| **Myristic acid (14:0) (per 0,1% in total FAs) in Red blood cells** | | | | | | | | | | |
| 6 | non-randomised studies | very serious^d^ | serious^g^ | not serious | serious^c^ | none | 3418/95399 (3.6%) | **RR 1.05** (0.92 to 1.20) | **2 more per 1.000** (from 3 fewer to 7 more) | ⨁◯◯◯ Very low^c,d,g^ |
| **Myristic acid (14:0) (per 0,1% in total FAs) in Plasma phospholipids** | | | | | | | | | | |
| 3 | non-randomised studies | very serious^d^ | not serious | not serious | serious^c^ | none | 1260/29945 (4.2%) | **RR 1.06** (0.92 to 1.20) | **3 more per 1.000** (from 3 fewer to 8 more) | ⨁◯◯◯ Very low^c,d^ |
| **Myristic acid (14:0) (per 0,1% in total FAs) in Total plasma** | | | | | | | | | | |
| 2 | non-randomised studies | very serious^d^ | not serious | not serious | very serious^h^ | none | 277/3333 (8.3%) | **RR 0.95** (0.90 to 1.01) | **4 fewer per 1.000** (from 8 fewer to 1 more) | ⨁◯◯◯ Very low^d,h^ |
| **Pentadecanoic acid, 15:0 (per 0.1% of total FAs) in all biospecimens** | | | | | | | | | | |
| 17 | non-randomised studies | serious^a^ | not serious | not serious | serious^i^ | publication bias strongly suspected^j^ | 16898/424402 (4.0%) | **RR 0.77** (0.65 to 0.91) | **9 fewer per 1.000** (from 14 fewer to 4 fewer) | ⨁◯◯◯ Very low^a,i,j^ |
| **Pentadecanoic acid (15:0) (per 0,1% of total FAs) in Red blood cells** | | | | | | | | | | |
| 8 | non-randomised studies | serious^a^ | not serious | not serious | serious^i^ | none | 3845/102141 (3.8%) | **RR 0.85** (0.76 to 0.96) | **6 fewer per 1.000** (from 9 fewer to 2 fewer) | ⨁⨁◯◯ Low^a,i^ |
| **Pentadecanoic acid (15:0) (per 0,1% of total FAs) in Plasma phospholipids** | | | | | | | | | | |
| 7 | non-randomised studies | serious^a^ | not serious | not serious | not serious | none | 13648/354750 (3.8%) | **RR 0.68** (0.56 to 0.81) | **12 fewer per 1.000** (from 17 fewer to 7 fewer) | ⨁⨁⨁◯ Moderate^a^ |
| **Pentadecanoic acid (15:0) (per 0,1% of total FAs) in Total plasma** | | | | | | | | | | |
| 4 | non-randomised studies | serious^a^ | serious^g^ | not serious | serious^c^ | none | 551/5890 (9.4%) | **RR 0.70** (0.46 to 1.07) | **28 fewer per 1.000** (from 51 fewer to 7 more) | ⨁◯◯◯ Very low^a,c,g^ |
| **Palmitic acid (16:0) (per 1.0% of total FAs) in all biospecimens** | | | | | | | | | | |
| 19 | non-randomised studies | serious^a^ | serious^g^ | not serious | serious^i^ | none | 17497/429414 (4.1%) | **RR 1.10** (1.05 to 1.15) | **4 more per 1.000** (from 2 more to 6 more) | ⨁◯◯◯ Very low^a,g,i^ |
| **Palmitic acid (16:0) (per 1.0% of total FAs) in Red blood cells** | | | | | | | | | | |
| 9 | non-randomised studies | serious^a^ | serious^g^ | not serious | not serious | none | 4069/104629 (3.9%) | **RR 1.06** (0.99 to 1.13) | **2 more per 1.000** (from 0 fewer to 5 more) | ⨁⨁◯◯ Low^a,g^ |
| **Palmitic acid (16:0) (per 1.0% of total FAs) in Plasma phospholipids** | | | | | | | | | | |
| 7 | non-randomised studies | serious^a^ | not serious | not serious | serious^i^ | none | 14093/370122 (3.8%) | **RR 1.10** (1.03 to 1.16) | **4 more per 1.000** (from 1 more to 6 more) | ⨁⨁◯◯ Low^a,i^ |
| **Palmitic acid (16:0) (per 1.0% of total FAs) in Total plasma** | | | | | | | | | | |
| 3 | non-randomised studies | serious^a^ | serious^g^ | not serious | serious^c^ | none | 479/4100 (11.7%) | **RR 1.12** (0.95 to 1.32) | **14 more per 1.000** (from 6 fewer to 37 more) | ⨁◯◯◯ Very low^a,c,g^ |
| **Palmitic acid (16:0) (per 1.0% of total FAs) in Cholesteryl ester** | | | | | | | | | | |
| 2 | non-randomised studies | serious^a^ | not serious | not serious | serious^e^ | none | 271/3043 (8.9%) | **RR 1.21** (1.07 to 1.36) | **19 more per 1.000** (from 6 more to 32 more) | ⨁⨁◯◯ Low^a,e^ |
| **Margaric acid (17:0) (per 0.1% of total FAs) in all biospecimens** | | | | | | | | | | |
| 14 | non-randomised studies | serious^a^ | serious^b^ | not serious | not serious | none | 15533/399908 (3.9%) | **RR 0.72** (0.60 to 0.88) | **11 fewer per 1.000** (from 16 fewer to 5 fewer) | ⨁⨁◯◯ Low^a,b^ |
| **Margaric acid (17:0) (per 0.1% of total FAs) in Red blood cells** | | | | | | | | | | |
| 7 | non-randomised studies | serious^a^ | serious^g^ | not serious | serious^i^ | none | 2968/76502 (3.9%) | **RR 0.78** (0.64 to 0.95) | **9 fewer per 1.000** (from 14 fewer to 2 fewer) | ⨁◯◯◯ Very low^a,g,i^ |
| **Margaric acid (17:0) (per 0.1% of total FAs) in Plasma phospholipids** | | | | | | | | | | |
| 5 | non-randomised studies | serious^a^ | not serious | not serious | not serious | none | 12853/346347 (3.7%) | **RR 0.64** (0.52 to 0.78) | **13 fewer per 1.000** (from 18 fewer to 8 fewer) | ⨁⨁⨁◯ Moderate^a^ |
| **Margaric acid (17:0) (per 0.1% of total FAs) in Total plasma** | | | | | | | | | | |
| 3 | non-randomised studies | very serious^d^ | not serious | not serious | serious^c^ | none | 405/5171 (7.8%) | **RR 0.79** (0.57 to 1.09) | **16 fewer per 1.000** (from 34 fewer to 7 more) | ⨁◯◯◯ Very low^c,d^ |
| **Stearic acid, 18:0 (per 1.0% of total FAs) in all biospecimens** | | | | | | | | | | |
| 19 | non-randomised studies | serious^a^ | very serious^f^ | not serious | not serious | none | 19232/429414 (4.5%) | **RR 1.04** (0.98 to 1.10) | **2 more per 1.000** (from 1 fewer to 4 more) | ⨁◯◯◯ Very low^a,f^ |
| **Stearic acid (18:0) (per 1.0% of total FAs) in Red blood cells** | | | | | | | | | | |
| 9 | non-randomised studies | serious^a^ | serious^g^ | not serious | not serious | none | 4069/104629 (3.9%) | **RR 1.03** (0.96 to 1.10) | **1 more per 1.000** (from 2 fewer to 4 more) | ⨁⨁◯◯ Low^a,g^ |
| **Stearic acid (18:0) (per 1.0% of total FAs) in Plasma phospholipids** | | | | | | | | | | |
| 7 | non-randomised studies | serious^a^ | serious^g^ | not serious | serious^c^ | none | 14093/370122 (3.8%) | **RR 1.09** (0.98 to 1.21) | **3 more per 1.000** (from 1 fewer to 8 more) | ⨁◯◯◯ Very low^a,c,g^ |
| **Stearic acid (18:0) (per 1.0% of total FAs) in Total plasma** | | | | | | | | | | |
| 3 | non-randomised studies | serious^a^ | not serious | not serious | serious^i^ | none | 479/4100 (11.7%) | **RR 0.87** (0.77 to 0.99) | **15 fewer per 1.000** (from 27 fewer to 1 fewer) | ⨁⨁◯◯ Low^a,i^ |
| **Stearic acid (18:0) (per 1.0% of total FAs) in Cholesteryl ester** | | | | | | | | | | |
| 2 | non-randomised studies | serious^a^ | not serious | not serious | serious^e^ | none | 272/3043 (8.9%) | **RR 1.73** (1.14 to 2.62) | **65 more per 1.000** (from 13 more to 145 more) | ⨁⨁◯◯ Low^a,e^ |
| **Arachidic acid, 20:0 (per 0.1% of total FAs) in all biospecimens** | | | | | | | | | | |
| 10 | non-randomised studies | serious^a^ | serious^g^ | not serious | serious^c^ | none | 14870/379617 (3.9%) | **RR 0.84** (0.67 to 1.06) | **6 fewer per 1.000** (from 13 fewer to 2 more) | ⨁◯◯◯ Very low^a,c,g^ |
| **Arachidic acid (20:0) (per 0.1% of total FAs) in Red blood cells** | | | | | | | | | | |
| 4 | non-randomised studies | serious^a^ | serious^g^ | not serious | serious^c^ | none | 1606/37306 (4.3%) | **RR 0.97** (0.76 to 1.23) | **1 fewer per 1.000** (from 10 fewer to 10 more) | ⨁◯◯◯ Very low^a,c,g^ |
| **Arachidic acid (20:0) (per 0.1% of total FAs) in Plasma phospholipids** | | | | | | | | | | |
| 6 | non-randomised studies | serious^a^ | serious^g^ | not serious | serious^c^ | none | 14022/369171 (3.8%) | **RR 0.64** (0.39 to 1.07) | **14 fewer per 1.000** (from 23 fewer to 3 more) | ⨁◯◯◯ Very low^a,c,g^ |
| **Arachidic acid (20:0) (per 0.1% of total FAs) in Total plasma** | | | | | | | | | | |
| 2 | non-randomised studies | serious^a^ | not serious | not serious | serious^c^ | none | 412/3795 (10.9%) | **RR 0.90** (0.73 to 1.11) | **11 fewer per 1.000** (from 29 fewer to 12 more) | ⨁⨁◯◯ Low^a,c^ |
| **Behenic acid (22:0) (per 0.1% of total FAs) in all biospecimens** | | | | | | | | | | |
| 11 | non-randomised studies | serious^a^ | serious^k^ | not serious | not serious | none | 14670/381160 (3.8%) | **RR 0.94** (0.88 to 1.01) | **2 fewer per 1.000** (from 5 fewer to 0 fewer) | ⨁⨁◯◯ Low^a,k^ |
| **Behenic acid (22:0) (per 0.1% of total FAs) in Red blood cells** | | | | | | | | | | |
| 4 | non-randomised studies | serious^a^ | not serious | not serious | not serious | none | 1606/37306 (4.3%) | **RR 1.02** (1.00 to 1.04) | **1 more per 1.000** (from 0 fewer to 2 more) | ⨁⨁⨁◯ Moderate^a^ |
| **Behenic acid (22:0) (per 0.1% of total FAs) in Plasma phospholipids** | | | | | | | | | | |
| 7 | non-randomised studies | serious^a^ | not serious | not serious | serious^c^ | none | 14045/369390 (3.8%) | **RR 0.88** (0.77 to 1.00) | **5 fewer per 1.000** (from 9 fewer to 0 fewer) | ⨁⨁◯◯ Low^a,c^ |
| **Behenic acid (22:0) (per 0.1% of total FAs) in Total plasma** | | | | | | | | | | |
| 3 | non-randomised studies | serious^a^ | serious^k^ | not serious | serious^c^ | none | 617/5338 (11.6%) | **RR 0.96** (0.86 to 1.07) | **5 fewer per 1.000** (from 16 fewer to 8 more) | ⨁◯◯◯ Very low^a,c,k^ |
| **Lignoceric acid (24:0) (per 0.1% of total FAs) in all biospecimens** | | | | | | | | | | |
| 10 | non-randomised studies | serious^a^ | serious^k^ | not serious | serious^i^ | publication bias strongly suspected^j^ | 14861/380213 (3.9%) | **RR 0.89** (0.82 to 0.98) | **4 fewer per 1.000** (from 7 fewer to 1 fewer) | ⨁◯◯◯ Very low^a,i,j,k^ |
| **Lignoceric acid (24:0) (per 0.1% of total FAs) in Red blood cells** | | | | | | | | | | |
| 4 | non-randomised studies | serious^a^ | not serious | not serious | not serious | none | 1606/37306 (4.3%) | **RR 1.00** (0.99 to 1.02) | **0 fewer per 1.000** (from 0 fewer to 1 more) | ⨁⨁⨁◯ Moderate^a^ |
| **Lignoceric acid (24:0) (per 0.1% of total FAs) in Plasma phospholipids** | | | | | | | | | | |
| 5 | non-randomised studies | serious^a^ | not serious | not serious | serious^i^ | none | 13708/366385 (3.7%) | **RR 0.73** (0.58 to 0.93) | **10 fewer per 1.000** (from 16 fewer to 3 fewer) | ⨁⨁◯◯ Low^a,i^ |
| **Lignoceric acid (24:0) (per 0.1% of total FAs) in Total phospholipids** | | | | | | | | | | |
| 3 | non-randomised studies | serious^a^ | not serious | not serious | serious^c^ | none | 617/5338 (11.6%) | **RR 0.92** (0.82 to 1.04) | **9 fewer per 1.000** (from 21 fewer to 5 more) | ⨁⨁◯◯ Low^a,c^ |

CI: confidence interval; FA: fatty acid; RR: relative risk

Absolute risks were calculated in GRADEpro (<https://www.gradepro.org/>) as the difference between the baseline risk of an outcome (e.g., in persons receiving control intervention or estimated in the observational studies) and the risk of outcome after the intervention is applied, i.e., the risk of an outcome in persons who were exposed or received an intervention. The calculation of the absolute effect was based on the relative magnitude of an effect and baseline risk. Absolute effect calculation option is selected by default and GRADEpro calculates the absolute effect risk once at least one of the baseline risk values is provided and the magnitude of the relative effect (RR, OR, or HR) has been entered.

*Numbers of cases and numbers of participants used for the calculation of absolute effects were obtained from the original cohorts in case if included publications applies case-cohort or nested case-control design.

**Explanations**

a. Downgraded by one level for risk of bias: not more than 50% (based on weights in meta-analysis) rated as serious RoB

b. Downgraded by one level for inconsistency: estimates pointed to different directions; 95% CIs mostly overlap but I2 >90%

c. Downgraded by one level for imprecision: Null value included in 95% CI AND CI included MID (5 cases per 1000), cases >400/ n>800

d. Downgraded by two levels for risk of bias: >50% (based on weights in meta-analysis) of the studies high RoB

e. Downgraded by one level for imprecision: Null value excluded in 95% CI AND CI excluded MID (5 cases per 1000), BUT n cases <400

f. Downgraded by two levels for inconsistency: Estimates pointed to different directions and 95% CI did not overlap

g. Downgraded by one level for inconsistency: Estimates pointed to different directions and 95% CIs only slightly overlapped

h. Downgraded by two levels for imprecision: Null value included in 95% CI AND CI included MID (5 cases per 1000) AND n cases <400

i. Downgraded by one level for imprecision: Null value excluded in 95% CI BUT CI included MID (5 cases per 1000), cases >400/ n>800

j. Downgraded by one level for publication bias: smaller studies indicating increased risk of T2D are missing.

k. Downgraded by one level for inconsistency: Estimates pointed to the same direction but 95% CIs did not overlap

**Supplemental Table 9.** Certainty of evidence of the meta-analyses of prospective cohort studies on the association between biomarkers of monounsaturated fatty acids (MUFAs) and risk of type 2 diabetes by using the GRADE tool.

| **Certainty assessment** | | | | | | | **N cases/ N participants for Absolute effects calculation*** | **Effect** | | **Certainty** |
| --- | --- | --- | --- | --- | --- | --- | --- | --- | --- | --- |
| **№ of studies** | **Study design** | **Risk of bias** | **Inconsistency** | **Indirectness** | **Imprecision** | **Other considerations** |  | **Relative (95% CI)** | **Absolute (95% CI)** |  |
| **Total MUFAs (per 3.0% of total FAs) in all biospecimens** | | | | | | | | | | |
| 6 | non-randomised studies | serious^a^ | not serious | not serious | serious^b^ | none | 15081/619302 (2.4%) | **RR 1.16** (1.03 to 1.32) | **4 more per 1.000** (from 1 more to 8 more) | ⨁⨁◯◯ Low^a,b^ |
| **Total MUFAs (per 3% in total FAs) in Red blood cells** | | | | | | | | | | |
| 3 | non-randomised studies | serious^a^ | not serious | not serious | serious^c^ | none | 2925/89383 (3.3%) | **RR 1.05** (0.88 to 1.26) | **2 more per 1.000** (from 4 fewer to 9 more) | ⨁⨁◯◯ Low^a,c^ |
| **Total MUFAs (per 3% in total FAs) in Plasma phospholipids** | | | | | | | | | | |
| 2 | non-randomised studies | very serious^d^ | not serious | not serious | very serious^e^ | none | 963/26941 (3.6%) | **RR 1.09** (0.80 to 1.47) | **3 more per 1.000** (from 7 fewer to 17 more) | ⨁◯◯◯ Very low^d,e^ |
| **Total MUFAs (per 3% in total FAs) in Total plasma** | | | | | | | | | | |
| 2 | non-randomised studies | serious^a^ | not serious | not serious | serious^c^ | none | 12085/528617 (2.3%) | **RR 1.24** (1.02 to 1.50) | **5 more per 1.000** (from 0 fewer to 11 more) | ⨁⨁◯◯ Low^a,c^ |
| **Palmitoleic acid (16:1n-7) (per 0.1% of total FAs) in all biospecimens** | | | | | | | | | | |
| 17 | non-randomised studies | serious^a^ | not serious | not serious | not serious | publication bias strongly suspected^f^ | 17153/424893 (4.0%) | **RR 1.04** (1.02 to 1.05) | **2 more per 1.000** (from 1 more to 2 more) | ⨁⨁◯◯ Low^a,f^ |
| **Palmitoleic acid (16:1n-7) (per 0.1% of total FAs) in Red blood cells** | | | | | | | | | | |
| 8 | non-randomised studies | serious^a^ | not serious | not serious | not serious | none | 3755/101946 (3.7%) | **RR 1.06** (1.03 to 1.10) | **2 more per 1.000** (from 1 more to 4 more) | ⨁⨁⨁◯ Moderate^a^ |
| **Palmitoleic acid (16:1n-7) (per 0.1% of total FAs) in Plasma phospholipids** | | | | | | | | | | |
| 7 | non-randomised studies | serious^a^ | not serious | not serious | not serious | none | 14093/370122 (3.8%) | **RR 1.07** (1.02 to 1.12) | **3 more per 1.000** (from 1 more to 5 more) | ⨁⨁⨁◯ Moderate^a^ |
| **Palmitoleic acid (16:1n-7) (per 0.1% of total FAs) in Total plasma** | | | | | | | | | | |
| 2 | non-randomised studies | serious^a^ | not serious | not serious | very serious^g^ | none | 351/2262 (15.5%) | **RR 1.02** (1.01 to 1.04) | **3 more per 1.000** (from 2 more to 6 more) | ⨁◯◯◯ Very low^a,g^ |
| **Palmitoleic acid (16:1n-7) (per 0.1% of total FAs) in Cholesteryl ester** | | | | | | | | | | |
| 2 | non-randomised studies | serious^a^ | not serious | not serious | serious^h^ | none | 351/2262 (15.5%) | **RR 1.01** (1.00 to 1.02) | **2 more per 1.000** (from 0 fewer to 3 more) | ⨁⨁◯◯ Low^a,h^ |
| **Palmitoelaidic acid (16:1n-9) (per 0.1% of total FAs) in all biospecimens** | | | | | | | | | | |
| 2 | non-randomised studies | serious^a^ | very serious^i^ | not serious | serious^c^ | none | 1146/30552 (3.8%) | **RR 1.05** (0.51 to 2.18) | **2 more per 1.000** (from 18 fewer to 44 more) | ⨁◯◯◯ Very low^a,c,i^ |
| **Oleic acid (18:1n-9) (per 1.0% of total FAs) in all biospecimens** | | | | | | | | | | |
| 17 | non-randomised studies | serious^a^ | not serious | not serious | not serious | none | 16714/437267 (3.8%) | **RR 1.04** (1.01 to 1.07) | **2 more per 1.000** (from 0 fewer to 3 more) | ⨁⨁⨁◯ Moderate^a^ |
| **Oleic acid (18:1n-9) (per 1.0% of total FAs) in Red blood cells** | | | | | | | | | | |
| 8 | non-randomised studies | serious^a^ | not serious | not serious | not serious | none | 3853/101946 (3.8%) | **RR 1.04** (1.01 to 1.06) | **2 more per 1.000** (from 0 fewer to 2 more) | ⨁⨁⨁◯ Moderate^a^ |
| **Oleic acid (18:1n-9) (per 1.0% of total FAs) in Plasma phospholipids** | | | | | | | | | | |
| 7 | non-randomised studies | serious^a^ | not serious | not serious | not serious | none | 14164/371424 (3.8%) | **RR 1.00** (0.96 to 1.04) | **0 fewer per 1.000** (from 2 fewer to 2 more) | ⨁⨁⨁◯ Moderate^a^ |
| **Oleic acid (18:1n-9) (per 1.0% of total FAs) in Total plasma** | | | | | | | | | | |
| 2 | non-randomised studies | serious^a^ | not serious | not serious | very serious^g^ | none | 350/2262 (15.5%) | **RR 1.10** (1.02 to 1.18) | **15 more per 1.000** (from 3 more to 28 more) | ⨁◯◯◯ Very low^a,g^ |
| **Oleic acid (18:1n-9) (per 1.0% of total FAs) in Cholesteryl ester** | | | | | | | | | | |
| 2 | non-randomised studies | serious^a^ | not serious | not serious | very serious^g^ | none | 272/3043 (8.9%) | **RR 1.07** (1.01 to 1.12) | **6 more per 1.000** (from 1 more to 11 more) | ⨁◯◯◯ Very low^a,g^ |
| **Eicosenoic acid (20:1) (per 0.1% of total FAs) in all biospecimens** | | | | | | | | | | |
| 3 | non-randomised studies | very serious^d^ | not serious | not serious | serious^c^ | none | 1812/54489 (3.3%) | **RR 0.92** (0.79 to 1.09) | **3 fewer per 1.000** (from 7 fewer to 3 more) | ⨁◯◯◯ Very low^c,d^ |
| **Eicosenoic acid (20:1) (per 0.1% of total FAs) in Red blood cells** | | | | | | | | | | |
| 2 | non-randomised studies | serious^a^ | not serious | not serious | serious^c^ | none | 1741/53187 (3.3%) | **RR 0.85** (0.63 to 1.15) | **5 fewer per 1.000** (from 12 fewer to 5 more) | ⨁⨁◯◯ Low^a,c^ |
| **Eicosenoic acid (20:1) (per 0.1% of total FAs) in Plasma phospholipids** | | | | | | | | | | |
| 2 | non-randomised studies | very serious^d^ | not serious | not serious | very serious^e^ | none | 963/26941 (3.6%) | **RR 0.75** (0.41 to 1.38) | **9 fewer per 1.000** (from 21 fewer to 14 more) | ⨁◯◯◯ Very low^d,e^ |
| **Nervonic acid (24:1) (per 0.1% of total FAs) in all biospecimens** | | | | | | | | | | |
| 3 | non-randomised studies | serious^a^ | not serious | not serious | not serious | none | 1812/54489 (3.3%) | **RR 1.00** (0.98 to 1.01) | **0 fewer per 1.000** (from 1 fewer to 0 fewer) | ⨁⨁⨁◯ Moderate^a^ |
| **Nervonic acid (24:1) (per 0.1% of total FAs) in Red blood cells** | | | | | | | | | | |
| 2 | non-randomised studies | serious^a^ | not serious | not serious | not serious | none | 1741/53187 (3.3%) | **RR 1.00** (0.99 to 1.01) | **0 fewer per 1.000** (from 0 fewer to 0 fewer) | ⨁⨁⨁◯ Moderate^a^ |
| **Nervonic acid (24:1) (per 0.1% of total FAs) in Plasma phospholipids** | | | | | | | | | | |
| 2 | non-randomised studies | very serious^d^ | not serious | not serious | serious^h^ | none | 963/26941 (3.6%) | **RR 0.95** (0.88 to 1.03) | **2 fewer per 1.000** (from 4 fewer to 1 more) | ⨁◯◯◯ Very low^d,h^ |

CI: confidence interval; FA: fatty acid; RR: relative risk

Absolute risks were calculated in GRADEpro (<https://www.gradepro.org/>) as the difference between the baseline risk of an outcome (e.g., in persons receiving control intervention or estimated in the observational studies) and the risk of outcome after the intervention is applied, i.e., the risk of an outcome in persons who were exposed or received an intervention. The calculation of the absolute effect was based on the relative magnitude of an effect and baseline risk. Absolute effect calculation option is selected by default and GRADEpro calculates the absolute effect risk once at least one of the baseline risk values is provided and the magnitude of the relative effect (RR, OR, or HR) has been entered.

* The numbers of cases and participants used to calculate absolute effects were obtained from the original cohorts if the included publications applied a case-cohort or nested case-control design.

#### Explanations

a. Downgraded by one level for risk of bias: not more than 50% rated as serious RoB

b. Downgraded by one level for imprecision: Null value excluded in 95% CI BUT CI included MID (5 cases per 1000), cases >400/ n>800

c. Downgraded by one level for imprecision: Null value included in 95% CI AND CI included MID (5 cases per 1000), cases >400/ n>800

d. Downgraded by two levels for risk of bias: >50% (based on weights in meta-analysis) of the studies high RoB

e. Downgraded by two levels for imprecision: Null value included in 95% CI AND CI excluded MID (5 cases per 1000) AND cases <400

f. Downgraded by one level for publication bias: Smaller studies indicating decreased risk of T2D are missing.

g. Downgraded by two levels for imprecision: Null value excluded in 95% CI BUT CI included MID (5 cases per 1000) AND cases >400/ n>800

h. Downgraded by one level for imprecision: Null value included in 95% CI, 95% CI excluded MID (5 cases per 1000) BUT cases <400

i. Downgraded by two levels for inconsistency: Estimates point to different directions and 95% CI only slightly overlap.

**Supplemental Table 10.** Certainty of evidence of the meta-analyses of prospective cohort studies on the association between biomarkers of trans-fatty acids and risk of type 2 diabetes by using the GRADE tool.

| **Certainty assessment** | | | | | | | **N cases/ N participants for Absolute effects calculation*** | **Effect** | | **Certainty** |
| --- | --- | --- | --- | --- | --- | --- | --- | --- | --- | --- |
| **№ of studies** | **Study design** | **Risk of bias** | **Inconsistency** | **Indirectness** | **Imprecision** | **Other considerations** |  | **Relative (95% CI)** | **Absolute (95% CI)** |  |
| ***Trans*-Vaccenic acid (*t-*18:1n-7) (per 0.1% of total FAs) in all biospecimens** | | | | | | | | | | |
| 4 | non-randomised studies | serious^a^ | not serious | not serious | not serious | none | 2109/57493 (3.7%) | **RR 0.93** (0.87 to 1.00) | **3 fewer per 1.000** (from 5 fewer to 0 fewer) | ⨁⨁⨁◯ Moderate^a^ |
| ***Trans*-Vaccenic acid (*t-*18:1n7) (per 0.1% of total FAs) in Red blood cells** | | | | | | | | | | |
| 2 | non-randomised studies | serious^a^ | not serious | not serious | serious^b^ | none | 1741/53187 (3.3%) | **RR 0.89** (0.71 to 1.13) | **4 fewer per 1.000** (from 9 fewer to 4 more) | ⨁⨁◯◯ Low^a,c^ |
| ***Trans*-Vaccenic acid (*t-*18:1n7) (per 0.1% of total FAs) in Plasma phospholipids** | | | | | | | | | | |
| 3 | non-randomised studies | very serious^c^ | not serious | not serious | serious^d^ | none | 1260/29945 (4.2%) | **RR 0.90** (0.83 to 0.97) | **4 fewer per 1.000** (from 7 fewer to 1 fewer) | ⨁◯◯◯ Very low^b,d^ |
| ***Trans*-Palmitoleic acid (*t-*16:1n–7) (per 0.1% of total FAs) in Total plasma** | | | | | | | | | | |
| 3 | non-randomised studies | serious^a^ | serious^e^ | not serious | serious^b^ | none | 442/4102 (10.8%) | **RR 0.86** (0.63 to 1.18) | **15 fewer per 1.000** (from 40 fewer to 19 more) | ⨁◯◯◯ Very low^a,c,d^ |
| ***Trans*-Hypogeic acid (*t-*16:1n-9) (per 0.1% of total FAs) in all biospecimens** | | | | | | | | | | |
| 7 | non-randomised studies | serious^a^ | not serious | not serious | serious^b^ | none | 2068/36562 (5.7%) | **RR 0.90** (0.73 to 1.13) | **6 fewer per 1.000** (from 15 fewer to 7 more) | ⨁⨁◯◯ Low^a,c^ |
| ***Trans*-Hypogeic acid (*t-*16:1n–9) (per 0.1% of total FAs) in Red blood cells** | | | | | | | | | | |
| 6 | non-randomised studies | serious^a^ | not serious | not serious | very serious^f^ | none | 945/26580 (3.6%) | **RR 0.81** (0.44 to 1.49) | **7 fewer per 1.000** (from 20 fewer to 17 more) | ⨁◯◯◯ Very low^a,e^ |
| ***Trans*-Hypogeic acid (*t-*16:1n–9) (per 0.1% of total FAs) in Plasma phospholipids** | | | | | | | | | | |
| 4 | non-randomised studies | serious^a^ | not serious | not serious | serious^b^ | none | 1260/23911 (5.3%) | **RR 0.72** (0.34 to 1.51) | **15 fewer per 1.000** (from 35 fewer to 27 more) | ⨁⨁◯◯ Low^a,c^ |
| ***Trans*-Elaidic acid (*t-*18:1n–9) (per 0.1% of total FAs) in all biospecimens** | | | | | | | | | | |
| 13 | non-randomised studies | serious^a^ | not serious | not serious | not serious | none | 4085/76156 (5.4%) | **RR 0.99** (0.97 to 1.01) | **1 fewer per 1.000** (from 2 fewer to 1 more) | ⨁⨁⨁◯ Moderate^a^ |
| ***Trans*-Elaidic acid (*t-*18:1n–9) (per 0.1% of total FAs) in Red blood cells** | | | | | | | | | | |
| 7 | non-randomised studies | serious^a^ | not serious | not serious | not serious | None | 2962/66174 (4.5%) | **RR 0.99** (0.98 to 1.01) | **0 fewer per 1.000** (from 1 fewer to 0 fewer) | ⨁⨁⨁◯ Moderate^a^ |
| ***Trans*-Elaidic acid (*t-*18:1n–9) (per 0.1% of total FAs) in Plasma phospholipids** | | | | | | | | | | |
| 5 | non-randomised studies | serious^a^ | serious^e^ | not serious | serious^b^ | None | 2223/50852 (4.4%) | **RR 0.94** (0.87 to 1.01) | **3 fewer per 1.000** (from 6 fewer to 0 fewer) | ⨁◯◯◯ Very low^a,c,d^ |
| ***Trans*-Linoleic acid (*t-*18:2) (per 0.1% of total FAs) in all biospecimens** | | | | | | | | | | |
| 10 | non-randomised studies | serious^a^ | not serious | not serious | serious^b^ | None | 2464/36834 (6.7%) | **RR 0.95** (0.89 to 1.01) | **3 fewer per 1.000** (from 7 fewer to 1 more) | ⨁⨁◯◯ Low^a,c^ |
| ***Trans*-Linoleic acid (*t-*18:2) (per 0.1% in total FAs) in Red blood cells** | | | | | | | | | | |
| 5 | non-randomised studies | serious^a^ | not serious | not serious | serious^b^ | None | 902/11480 (7.9%) | **RR 0.98** (0.88 to 1.08) | **2 fewer per 1.000** (from 9 fewer to 6 more) | ⨁⨁◯◯ Low^a,c^ |
| ***Trans*-Linoleic acid (*t-*18:2) (per 0.1% in total FAs) in Plasma phospholipids** | | | | | | | | | | |
| 4 | non-randomised studies | serious^a^ | not serious | not serious | serious^d^ | None | 1260/23911 (5.3%) | **RR 0.80** (0.66 to 0.98) | **11 fewer per 1.000** (from 18 fewer to 1 fewer) | ⨁⨁◯◯ Low^a,b^ |

CI: confidence interval; FA, fatty acid; RR: relative risk

Absolute risks were calculated in GRADEpro (<https://www.gradepro.org/>) as the difference between the baseline risk of an outcome (e.g., in persons receiving control intervention or estimated in the observational studies) and the risk of outcome after the intervention is applied, i.e., the risk of an outcome in persons who were exposed or received an intervention. The calculation of the absolute effect was based on the relative magnitude of an effect and baseline risk. Absolute effect calculation option is selected by default and GRADEpro calculates the absolute effect risk once at least one of the baseline risk values is provided and the magnitude of the relative effect (RR, OR, or HR) has been entered.

* The numbers of cases and participants used to calculate absolute effects were obtained from the original cohorts if the included publications applied a case-cohort or nested case-control design.

**Explanations**

1. Downgraded by one level for risk of bias: not more than 50% (based on weights in meta-analysis) of studies were assessed as high risk of bias.
2. Downgraded by one level for imprecision: Null value included in 95% CI AND CI included MID (5 cases per 1000), cases >400/ n>800.
3. Downgraded by two levels for risk of bias: >50% (based on weights in meta-analysis) of the studies high RoB
4. Downgraded by one level for imprecision: Null value excluded in 95% CI BUT CI included MID (5 cases per 1000), cases >400/ n>800
5. Downgraded by one level for inconsistency: Not all estimates pointed to the same direction, and 95% CIs partially overlap
6. Downgraded by two levels for imprecision: Null value included in 95% CI AND CI excluded MID (5 cases per 1000) AND cases <400

**Supplemental Table 11.** Certainty of evidence of the meta-analyses of prospective cohort studies on the association between biomarkers of polyunsaturated fatty acids (PUFAs) and risk of type 2 diabetes by using the GRADE tool.

| **Certainty assessment** | | | | | | | **N cases/ N participants for Absolute effects calculation*** | **Effect** | | **Certainty** |
| --- | --- | --- | --- | --- | --- | --- | --- | --- | --- | --- |
| **№ of studies** | **Study design** | **Risk of bias** | **Inconsistency** | **Indirectness** | **Imprecision** | **Other considerations** |  | **Relative (95% CI)** | **Absolute (95% CI)** |  |
| **Total PUFAs (per 3.0% of total FAs) in all biospecimens** | | | | | | | | | | |
| 6 | non-randomised studies | serious^a^ | not serious | not serious | not serious | none | 15081/619302 (2.4%) | **RR 0.96** (0.89 to 1.05) | **1 fewer per 1.000** (from 3 fewer to 1 more) | ⨁⨁⨁◯ Moderate^a^ |
| **Total PUFAs (per 3.0% of total FAs) in Red blood cells** | | | | | | | | | | |
| 3 | non-randomised studies | serious^a^ | not serious | not serious | not serious | none | 2925/89383 (3.3%) | **RR 0.97** (0.89 to 1.06) | **1 fewer per 1.000** (from 4 fewer to 2 more) | ⨁⨁⨁◯ Moderate^a^ |
| **Total PUFAs (per 3.0% of total FAs) in Total plasma** | | | | | | | | | | |
| 2 | non-randomised studies | serious^a^ | very serious^c^ | not serious | not serious | none | 12085/528617 (2.3%) | **RR 0.96** (0.81 to 1.14) | **1 fewer per 1.000** (from 4 fewer to 3 more) | ⨁◯◯◯ Very low^a,c^ |
| **Total n-6 PUFAs (per 3.0% of total FAs) in all biospecimens** | | | | | | | | | | |
| 5 | non-randomised studies | serious^a^ | serious^d^ | not serious | not serious | none | 25181/873771 (2.9%) | **RR 0.94** (0.88 to 1.01) | **2 fewer per 1.000** (from 3 fewer to 0 fewer) | ⨁⨁◯◯ Low^a,d^ |
| **Total n6-PUFAs (per 3.0% of total FAs) in Red blood cells** | | | | | | | | | | |
| 3 | non-randomised studies | serious^a^ | not serious | not serious | not serious | none | 2017/55918 (3.6%) | **RR 0.96** (0.88 to 1.05) | **1 fewer per 1.000** (from 4 fewer to 2 more) | ⨁⨁⨁◯ Moderate^a^ |
| **Total n6-PUFAs (per 3.0% of total FAs) in Total plasma** | | | | | | | | | | |
| 2 | non-randomised studies | serious^a^ | very serious^c^ | not serious | not serious | none | 12085/528617 (2.3%) | **RR 0.97** (0.78 to 1.21) | **1 fewer per 1.000** (from 5 fewer to 5 more) | ⨁◯◯◯ Very low^a,c^ |
| **Linoleic acid (18:2) (per 0.1% of total FAs) in all biospecimens** | | | | | | | | | | |
| 25 | non-randomised studies | serious^a^ | serious^d^ | not serious | not serious | publication bias strongly suspected^e^ | 29853/949430 (3.1%) | **RR 0.96** (0.94 to 0.97) | **1 fewer per 1.000** (from 2 fewer to 1 fewer) | ⨁◯◯◯ Very low^a,d,e^ |
| **Linoleic acid (18:2) (per 1.0% of total FAs) in Red blood cells** | | | | | | | | | | |
| 11 | non-randomised studies | serious^a^ | not serious | not serious | not serious | publication bias strongly suspected^e^ | 4306/105778 (4.1%) | **RR 0.92** (0.88 to 0.97) | **3 fewer per 1.000** (from 5 fewer to 1 fewer) | ⨁⨁◯◯ Low^a,e^ |
| **Linoleic acid (18:2) (per 1.0% of total FAs) in Plasma phospholipids** | | | | | | | | | | |
| 8 | non-randomised studies | serious^a^ | not serious | not serious | not serious | none | 14231/370471 (3.8%) | **RR 0.93** (0.91 to 0.95) | **3 fewer per 1.000** (from 3 fewer to 2 fewer) | ⨁⨁⨁◯ Moderate^a^ |
| **Linoleic acid (18:2) (per 1.0% of total FAs) in Total plasma** | | | | | | | | | | |
| 8 | non-randomised studies | serious^a^ | not serious | not serious | not serious | none | 13305/537409 (2.5%) | **RR 0.97** (0.95 to 0.99) | **1 fewer per 1.000** (from 1 fewer to 0 fewer) | ⨁⨁⨁◯ Moderate^a^ |
| **Linoleic acid (18:2) (per 1.0% of total FAs) in Cholesteryl ester** | | | | | | | | | | |
| 4 | non-randomised studies | serious^a^ | not serious | not serious | not serious | none | 641/7271 (8.8%) | **RR 0.97** (0.95 to 1.00) | **3 fewer per 1.000** (from 4 fewer to 0 fewer) | ⨁⨁⨁◯ Moderate^a^ |
| **γ-Linolenic acid (γ-18:3) (per 0.1% of total FAs) in all biospecimens** | | | | | | | | | | |
| 4 | non-randomised studies | serious^a^ | not serious | not serious | not serious | none | 13167/346456 (3.8%) | **RR 1.05** (1.00 to 1.09) | **2 more per 1.000** (from 0 fewer to 3 more) | ⨁⨁⨁◯ Moderate^a^ |
| **γ-Linolenic acid (γ-18:3) (per 0.1% of total FAs) in Red blood cells** | | | | | | | | | | |
| 4 | non-randomised studies | serious^a^ | not serious | not serious | not serious | none | 2017/55918 (3.6%) | **RR 2.23** (1.42 to 3.49) | **44 more per 1.000** (from 15 more to 90 more) | ⨁⨁⨁◯ Moderate^a^ |
| **Dihomo-γ-linolenic acid (γ-20:3) (per 0.1% of total FAs) in all biospecimens** | | | | | | | | | | |
| 5 | non-randomised studies | serious^a^ | not serious | not serious | not serious | none | 14086/382980 (3.7%) | **RR 1.03** (1.01 to 1.05) | **1 more per 1.000** (from 0 fewer to 2 more) | ⨁⨁⨁◯ Moderate^a^ |
| **Dihomo-γ-linolenic acid (γ-20:3) (per 0.1% of total FAs) in Red blood cells** | | | | | | | | | | |
| 2 | non-randomised studies | serious^a^ | not serious | not serious | not serious | none | 2033/63744 (3.2%) | **RR 1.07** (1.03 to 1.11) | **2 more per 1.000** (from 1 more to 4 more) | ⨁⨁⨁◯ Moderate^a^ |
| **Dihomo-γ-linolenic acid (γ-20:3) (per 0.1% of total FAs) in Plasma phospholipids** | | | | | | | | | | |
| 2 | non-randomised studies | serious^a^ | not serious | not serious | not serious | none | 12474/341536 (3.7%) | **RR 1.06** (1.05 to 1.07) | **2 more per 1.000** (from 2 more to 3 more) | ⨁⨁⨁◯ Moderate^a^ |
| **Arachidonic acid (20:4) (per 1.0% of total FAs) in all biospecimens** | | | | | | | | | | |
| 22 | non-randomised studies | serious^a^ | not serious | not serious | not serious | none | 18059/434784 (4.2%) | **RR 1.00** (0.98 to 1.02) | **0 fewer per 1.000** (from 1 fewer to 1 more) | ⨁⨁⨁◯ Moderate^a^ |
| **Arachidonic acid (20:4) (per 1.0% of total FAs) in Red blood cells** | | | | | | | | | | |
| 10 | non-randomised studies | serious^a^ | not serious | not serious | not serious | none | 3424/79407 (4.3%) | **RR 0.97** (0.93 to 1.02) | **1 fewer per 1.000** (from 3 fewer to 1 more) | ⨁⨁⨁◯ Moderate^a^ |
| **Arachidonic acid (20:4) (per 1.0% of total FAs) in Plasma phospholipids** | | | | | | | | | | |
| 8 | non-randomised studies | serious^a^ | not serious | not serious | not serious | none | 14231/371471 (3.8%) | **RR 1.02** (1.01 to 1.04) | **1 more per 1.000** (from 0 fewer to 2 more) | ⨁⨁⨁◯ Moderate^a^ |
| **Arachidonic acid (20:4) (per 1.0% of total FAs) in Total plasma** | | | | | | | | | | |
| 6 | non-randomised studies | serious^a^ | not serious | not serious | serious^f^ | none | 1220/8792 (13.9%) | **RR 0.92** (0.85 to 0.99) | **11 fewer per 1.000** (from 21 fewer to 1 fewer) | ⨁⨁◯◯ Low^a,f^ |
| **Arachidonic acid (20:4) (per 1.0% of total FAs) in Cholesteryl ester** | | | | | | | | | | |
| 4 | non-randomised studies | serious^a^ | not serious | not serious | serious^g^ | none | 624/6903 (9.0%) | **RR 1.04** (0.98 to 1.11) | **4 more per 1.000** (from 2 fewer to 10 more) | ⨁⨁◯◯ Low^a,g^ |
| **Docosatetraenoic acid (22:4) (per 0.1% of total FAs) in all biospecimens** | | | | | | | | | | |
| 3 | non-randomised studies | very serious^b^ | not serious | not serious | serious^f^ | none | 13658/377732 (3.6%) | **RR 1.16** (1.07 to 1.26) | **6 more per 1.000** (from 3 more to 9 more) | ⨁◯◯◯ Very low^b,f^ |
| **Docosatetraenoic acid (20:4) (per 0.1% of total FAs) in Red blood cells** | | | | | | | | | | |
| 3 | non-randomised studies | very serious^b^ | not serious | not serious | not serious | none | 2925/89383 (3.3%) | **RR 1.02** (0.98 to 1.06) | **1 more per 1.000** (from 1 fewer to 2 more) | ⨁⨁◯◯ Low^b^ |
| **Docosatetraenoic acid (20:4) (per 0.1% of total FAs) in Plasma phospholipids** | | | | | | | | | | |
| 2 | non-randomised studies | very serious^b^ | not serious | not serious | not serious | none | 12474/341536 (3.7%) | **RR 1.22** (1.13 to 1.32) | **8 more per 1.000** (from 5 more to 12 more) | ⨁⨁◯◯ Low^b^ |
| **Total n-3 PUFAs (per 3.0% of total FAs) in all biospecimens** | | | | | | | | | | |
| 3 | non-randomised studies | serious^a^ | not serious | not serious | serious^f^ | none | 24488/868851 (2.8%) | **RR 0.79** (0.65 to 0.97) | **6 fewer per 1.000** (from 10 fewer to 1 fewer) | ⨁⨁◯◯ Low^a,f^ |
| **Total n-3 PUFAs (per 3.0% of total FAs) in Red blood cells** | | | | | | | | | | |
| 2 | non-randomised studies | serious^a^ | not serious | not serious | serious^g^ | none | 1741/53187 (3.3%) | **RR 0.98** (0.83 to 1.17) | **1 fewer per 1.000** (from 6 fewer to 6 more) | ⨁⨁◯◯ Low^a,g^ |
| **Total n-3 PUFAs (per 3.0% of total FAs) in Total plasma** | | | | | | | | | | |
| 2 | non-randomised studies | serious^a^ | not serious | not serious | serious^f^ | none | 12085/528617 (2.3%) | **RR 0.70** (0.52 to 0.94) | **7 fewer per 1.000** (from 11 fewer to 1 fewer) | ⨁⨁◯◯ Low^a,f^ |
| **α-Linolenic acid, (α-18:3) (per 0.1% of total FAs) in all biospecimens** | | | | | | | | | | |
| 24 | non-randomised studies | serious^a^ | not serious | not serious | not serious | none | 8671/436496 (2.0%) | **RR 1.00** (0.97 to 1.02) | **0 fewer per 1.000** (from 1 fewer to 0 fewer) | ⨁⨁⨁◯ Moderate^a^ |
| **α-Linolenic acid (α-18:3) (per 0.1% of total FAs) in Red blood cells** | | | | | | | | | | |
| 10 | non-randomised studies | serious^a^ | not serious | not serious | not serious | none | 4212/104865 (4.0%) | **RR 1.01** (0.91 to 1.13) | **0 fewer per 1.000** (from 4 fewer to 5 more) | ⨁⨁⨁◯ Moderate^a^ |
| **α-Linolenic acid (α-18:3) (per 0.1% of total FAs) in Plasma phospholipids** | | | | | | | | | | |
| 9 | non-randomised studies | serious^a^ | not serious | not serious | not serious | none | 3465/371806 (0.9%) | **RR 0.95** (0.87 to 1.03) | **0 fewer per 1.000** (from 1 fewer to 0 fewer) | ⨁⨁⨁◯ Moderate^a^ |
| **α-Linolenic acid (α-18:3) (per 0.1% of total FAs) in Total plasma** | | | | | | | | | | |
| 7 | non-randomised studies | serious^a^ | not serious | not serious | serious^g^ | none | 1967/10142 (19.4%) | **RR 1.01** (0.98 to 1.04) | **2 more per 1.000** (from 4 fewer to 8 more) | ⨁⨁◯◯ Low^a,g^ |
| **α-Linolenic acid (α-18:3) (per 0.1% of total FAs) in Cholesteryl ester** | | | | | | | | | | |
| 3 | non-randomised studies | serious^a^ | not serious | not serious | serious^g^ | none | 503/4035 (12.5%) | **RR 0.98** (0.94 to 1.03) | **2 fewer per 1.000** (from 7 fewer to 4 more) | ⨁⨁◯◯ Low^a,g^ |
| **Eicosapentaenoic acid (20:5) (per 0.1% of total FAs) in all biospecimens** | | | | | | | | | | |
| 24 | non-randomised studies | serious^a^ | not serious | not serious | not serious | none | 19028/436415 (4.4%) | **RR 0.99** (0.98 to 1.00) | **0 fewer per 1.000** (from 1 fewer to 0 fewer) | ⨁⨁⨁◯ Moderate^a^ |
| **Eicosapentaenoic acid (20:5) (per 0.1% of total FAs) in Red blood cells** | | | | | | | | | | |
| 10 | non-randomised studies | serious^a^ | not serious | not serious | not serious | none | 4212/104865 (4.0%) | **RR 0.97** (0.95 to 0.99) | **1 fewer per 1.000** (from 2 fewer to 0 fewer) | ⨁⨁⨁◯ Moderate^a^ |
| **Eicosapentaenoic acid (20:5) (per 0.1% of total FAs) in Plasma phospholipids** | | | | | | | | | | |
| 9 | non-randomised studies | serious^a^ | not serious | not serious | not serious | none | 14471/371725 (3.9%) | **RR 1.00** (0.99 to 1.01) | **0 fewer per 1.000** (from 0 fewer to 0 fewer) | ⨁⨁⨁◯ Moderate^a^ |
| **Eicosapentaenoic acid (20:5) (per 0.1% of total FAs) in Total plasma** | | | | | | | | | | |
| 7 | non-randomised studies | serious^a^ | not serious | not serious | not serious | none | 1937/11585 (16.7%) | **RR 0.99** (0.98 to 1.00) | **2 fewer per 1.000** (from 3 fewer to 0 fewer) | ⨁⨁⨁◯ Moderate^a^ |
| **Eicosapentaenoic acid (20:5) (per 0.1% of total FAs) in Cholesteryl ester** | | | | | | | | | | |
| 3 | non-randomised studies | serious^a^ | not serious | not serious | not serious | none | 503/4035 (12.5%) | **RR 0.99** (0.98 to 1.01) | **1 fewer per 1.000** (from 2 fewer to 1 more) | ⨁⨁⨁◯ Moderate^a^ |
| **Docosapentaenoic acid (22:5) (per 0.1% of total FAs) in all biospecimens** | | | | | | | | | | |
| 23 | non-randomised studies | serious^a^ | not serious | not serious | not serious | none | 17911/432041 (4.1%) | **RR 0.98** (0.97 to 0.99) | **1 fewer per 1.000** (from 1 fewer to 0 fewer) | ⨁⨁⨁◯ Moderate^a^ |
| **Docosapentaenoic acid (22:5) (per 0.1% of total FAs) in Red blood cells** | | | | | | | | | | |
| 10 | non-randomised studies | serious^a^ | not serious | not serious | not serious | publication bias strongly suspected^e^ | 4212/104865 (4.0%) | **RR 0.98** (0.97 to 1.00) | **1 fewer per 1.000** (from 1 fewer to 0 fewer) | ⨁⨁◯◯ Low^a,e^ |
| **Docosapentaenoic acid (22:5) (per 0.1% of total FAs) in Plasma phospholipids** | | | | | | | | | | |
| 9 | non-randomised studies | serious^a^ | not serious | not serious | not serious | none | 14471/371725 (3.9%) | **RR 0.97** (0.94 to 1.00) | **1 fewer per 1.000** (from 2 fewer to 0 fewer) | ⨁⨁⨁◯ Moderate^a^ |
| **Docosapentaenoic acid (22:5) (per 0.1% of total FAs) in Total plasma** | | | | | | | | | | |
| 6 | non-randomised studies | serious^a^ | serious^d^ | not serious | serious^g^ | none | 1316/10142 (13.0%) | **RR 0.96** (0.91 to 1.01) | **5 fewer per 1.000** (from 12 fewer to 1 more) | ⨁◯◯◯ Very low^a,d,g^ |
| **Docosahexaenoic acid (22:6) (per 1.0% of total FAs) in all biospecimens** | | | | | | | | | | |
| 26 | non-randomised studies | serious^a^ | serious^h^ | not serious | not serious | none | 31113/965032 (3.2%) | **RR 0.93** (0.90 to 0.97) | **2 fewer per 1.000** (from 3 fewer to 1 fewer) | ⨁⨁◯◯ Low^a,h^ |
| **Docosahexaenoic acid (22:6) (per 1.0% of total FAs) in Red blood cells** | | | | | | | | | | |
| 10 | non-randomised studies | serious^a^ | not serious | not serious | not serious | none | 4212/104865 (4.0%) | **RR 1.01** (0.98 to 1.04) | **0 fewer per 1.000** (from 1 fewer to 2 more) | ⨁⨁⨁◯ Moderate^a^ |
| **Docosahexaenoic acid (22:6) (per 1.0% of total FAs) in Plasma phospholipids** | | | | | | | | | | |
| 9 | non-randomised studies | serious^a^ | not serious | not serious | not serious | none | 14471/371725 (3.9%) | **RR 0.92** (0.88 to 0.96) | **3 fewer per 1.000** (from 5 fewer to 2 fewer) | ⨁⨁⨁◯ Moderate^a^ |
| **Docosahexaenoic acid (22:6) (per 1.0% of total FAs) in Total plasma** | | | | | | | | | | |
| 8 | non-randomised studies | serious^a^ | not serious | not serious | serious^f^ | none | 13947/538759 (2.6%) | **RR 0.78** (0.65 to 0.93) | **6 fewer per 1.000** (from 9 fewer to 2 fewer) | ⨁⨁◯◯ Low^a,f^ |
| **Docosahexaenoic acid (22:6) (per 1.0% of total FAs) in Cholesteryl ester** | | | | | | | | | | |
| 3 | non-randomised studies | serious^a^ | not serious | not serious | serious^g^ | none | 503/4035 (12.5%) | **RR 0.92** (0.65 to 1.31) | **10 fewer per 1.000** (from 44 fewer to 39 more) | ⨁⨁◯◯ Low^a,g^ |
| **EPA+DHA (20:5+22:6) (per 1.0% of total FAs) in all biospecimens** | | | | | | | | | | |
| 2 | non-randomised studies | very serious^b^ | serious^d^ | not serious | very serious^i^ | none | 335/4120 (8.1%) | **RR 0.94** (0.92 to 0.96) | **5 fewer per 1.000** (from 7 fewer to 3 fewer) | ⨁◯◯◯ Very low^b,d,i^ |
| **EPA + DPA + DHA (20:5+22:5+22:6) (per 1.0% of total FAs) in all biospecimens** | | | | | | | | | | |
| 21 | non-randomised studies | serious^a^ | not serious | not serious | not serious | none | 17702/396128 (4.5%) | **RR 0.94** (0.92 to 0.96) | **3 fewer per 1.000** (from 4 fewer to 2 fewer) | ⨁⨁⨁◯ Moderate^a^ |
| **EPA + DPA + DHA (20:5+22:5+22:6) (per 1.0% of total FAs) in Red blood cells** | | | | | | | | | | |
| 6 | non-randomised studies | serious^a^ | not serious | not serious | serious^f^ | none | 1156/14450 (8.0%) | **RR 0.95** (0.91 to 0.99) | **4 fewer per 1.000** (from 7 fewer to 1 fewer) | ⨁⨁◯◯ Low^a,f^ |
| **EPA + DPA + DHA (20:5+22:5+22:6) (per 1.0% of total FAs) in Plasma phospholipids** | | | | | | | | | | |
| 8 | non-randomised studies | serious^a^ | not serious | not serious | not serious | none | 14400/370423 (3.9%) | **RR 0.94** (0.91 to 0.96) | **2 fewer per 1.000** (from 3 fewer to 2 fewer) | ⨁⨁⨁◯ Moderate^a^ |
| **EPA + DPA + DHA (20:5+22:5+22:6) (per 1.0% of total FAs) in Total plasma** | | | | | | | | | | |
| 7 | non-randomised studies | serious^a^ | not serious | not serious | not serious | none | 1967/11585 (17.0%) | **RR 0.94** (0.91 to 0.97) | **10 fewer per 1.000** (from 15 fewer to 5 fewer) | ⨁⨁⨁◯ Moderate^a^ |
| **EPA + DPA + DHA (20:5+22:5+22:6) (per 1.0% of total FAs) in Cholesteryl ester** | | | | | | | | | | |
| 3 | non-randomised studies | serious^a^ | not serious | not serious | serious^g^ | none | 503/4035 (12.5%) | **RR 0.95** (0.86 to 1.05) | **6 fewer per 1.000** (from 17 fewer to 6 more) | ⨁⨁◯◯ Low^a,g^ |

CI: confidence interval; DHA: Docosahexaenoic acid, DPA: Docosapentaenoic acid, EPA: Eicosapentaenoic acid, FA: fatty acid, RR: relative risk

Absolute risks were calculated in GRADEpro (<https://www.gradepro.org/>) as the difference between the baseline risk of an outcome (e.g., in persons receiving control intervention or estimated in the observational studies) and the risk of outcome after the intervention is applied, i.e., the risk of an outcome in persons who were exposed or received an intervention. The calculation of the absolute effect was based on the relative magnitude of an effect and baseline risk. Absolute effect calculation option is selected by default and GRADEpro calculates the absolute effect risk once at least one of the baseline risk values is provided and the magnitude of the relative effect (RR, OR, or HR) has been entered.

* The numbers of cases and participants used to calculate absolute effects were obtained from the original cohorts if the included publications applied a case-cohort or nested case-control design.

**Explanations**

a. Downgraded by one level for risk of bias: not more than 50% rated as serious RoB

b. Downgraded by two levels for risk of bias: >50% (based on weights in meta-analysis) of the studies high RoB

c. Downgraded by two levels for inconsistency: estimates pointed to different directions and 95% CIs did not overlap

d. Downgraded by one level for inconsistency: estimates pointed to different directions and 95% CIs partially overlapped

e. Downgraded by one level due publication bias: Small studies on positive association were missing.

f. Downgraded by one level for imprecision: Null value excluded in 95% CI BUT CI included MID (5 cases per 1000), cases >400/ n>800

g. Downgraded by one level for imprecision: Null value included in 95% CI AND CI included MID (5 cases per 1000), cases >400/ n>800

h. Downgraded by one level for inconsistency: estimates pointed to the same direction but 95% CIs partially overlapped

i. Downgraded by two levels for imprecision: Null value excluded in 95% CI AND CI included MID (5 cases per 1000) AND n cases <400

**Supplemental Table 12.** Subgroup analyses by continent of the cohorts included in the meta-analyses on the association between biomarkers of dietary intake of specific fatty acids measured in various biospecimens and risk of type 2 diabetes.

|  | Europe | | | USA | | | Asia | | | P-value between |
| --- | --- | --- | --- | --- | --- | --- | --- | --- | --- | --- |
|  | N cohorts | SRR (95%CI) | I^2^ | N cohorts | SRR (95%CI) | I^2^ | N cohorts | SRR (95%CI) | I^2^ |  |
| Linoleic acid (18:2) in red blood cells | 6 | 0.86 (0.80; 0.94) | 35 | 4 | 0.97 (0.92; 1.02) | 51 | 1 | 0.97 (0.87; 1.06) | - | 0.142 |
| Arachidonic acid (20:4) in red blood cells | 5 | 1.05 (0.94; 1.14) | 40 | 4 | 0.93 (0.87; 0.99) | 57 | 1 | 1.00 (0.94; 1.05) | - | 0.249 |
| α-Linolenic acid (α-18:3) in red blood cells | 5 | 0.87 (0.71; 1.07) | 52 | 4 | 1.12 (1.03; 1.21) | 0 | 1 | 1.16 (0.63; 2.14) | - | 0.141 |
| Eicosapentaenoic acid (20:5) in red blood cells | 5 | 0.96 (0.94; 0.98) | 0 | 4 | 1.00 (0.96; 1.03) | 28 | 1 | 0.96 (0.93; 0.9) | - | 0.227 |
| Docosapentaenoic acid (22:5) in red blood cells | 5 | 1.00 (0.98; 1.02) | 45 | 4 | 0.97 (0.95; 0.99) | 0 | 1 | 0.95 (0.91; 1.00) | - | 0.201 |
| Docosahexaenoic acid (22:6) in red blood cells | 5 | 1.02 (1.00; 1.03) | 0 | 4 | 0.95 (0.89; 1.01) | 0 | 1 | 1.13 (0.98; 1.30) | - | 0.109 |
| CI: confidence interval, SRR: summary relative risk | | | | | | | | | | |

**Supplemental Table 13.** Sensitivity analysis excluding studies with high risk of bias from the meta-analyses on the association between biomarkers of dietary intake of specific fatty acids measured in various biospecimens and risk of type 2 diabetes.

| **Saturated fatty acids** | | | | | | | | | | | |
| --- | --- | --- | --- | --- | --- | --- | --- | --- | --- | --- | --- |
| *Red blood cells* | | | Plasma phospholipids | | | Total plasma | | | Cholesteryl ester | | |
| N | SRR (95%CI) | I^2^ | N cohorts | SRR (95%CI) | I^2^ | N cohorts | SRR (95%CI) | I^2^ | N cohorts | SRR (95%CI) | I^2^ |
| *Total Saturated fatty acids* | | | | | | | | | | | |
| - | - | - | - | - | - | 2 | 1.09 (0.63; 1.89) | 99 | - | - | - |
| *Myristic acid (14:0)* | | | | | | | | | | | |
| 2 | 0.95 (0.72; 1.25) | 84 | - | - | - | - | - | - | - | - | - |
| *Pentadecanoic acid (15:0)* | | | | | | | | | | | |
| 5 | 0.88 (0.78; 1.00) | 10 | 7 | 0.68 (0.56; 0.81) | 58 | 2 | 0.84 (0.52; 1.38) | 85 | - | - | - |
| *Palmitic acid (16:0)* | | | | | | | | | | | |
| 7 | 1.06 (0.98; 1.13) | 85 | 7 | 1.10 (1.03; 1.16) | 55 | 3 | 1.12 (0.95; 1.32) | 93 | 2 |  |  |
| *Margaric acid (17:0)* | | | | | | | | | | | |
| 5 | 0.89 (0.74; 1.07) | 77 | 5 | 0.64 (0.52; 0.78) | 67 | - | - | - | - | - | - |
| *Stearic acid (18:0)* | | | | | | | | | | | |
| 7 | 1.03 (0.96; 1.11) | 77 | 7 | 1.09 (0.98; 1.21) | 77 | 3 | 0.87 (0.77; 0.99) | 0 | - | - | - |
| *Arachidic acid (20:0)* | | | | | | | | | | | |
| 4 | 0.97 (0.76; 1.23) | 69 | 6 | 0.64 (0.39; 1.07) | 85 | 2 | 0.90 (0.73; 1.11) | 63 | - | - | - |
| *Behenic acid (22:0)* | | | | | | | | | | | |
| 4 | 1.02 (1.00; 1.04) | 0 | 6 | 0.88 (0.77; 1.00) | 73 | 3 | 0.96 (0.86; 1.07) | 78 | - | - | - |
| *Lignoceric acid (24:0)* | | | | | | | | | | | |
| 4 | 1.00 (0.99; 1.02) | 0 | 5 | 0.73 (0.25; 0.93) | 82 | 3 | 0.92 (0.82; 1.04) | 52 | - | - | - |
| **Monounsaturated fatty acids** | | | | | | | | | | | |
| *Total monounsaturated fatty acids* | | | | | | | | | | | |
| - | - | - | - | - | - | 2 | 1.24 (1.02; 1.50) | 87 | - | - | - |
| *Palmitoleic acid (16:1n-7)* | | | | | | | | | | | |
| 5 | 1.05 (1.02; 1.08) | 83 | 7 | 1.07 (1.02; 1.12) | 73 | 2 | 1.02 (1.01; 1.04) | 0 | - | - | - |
| *Oleic acid (18:1n-9)* | | | | | | | | | | | |
| 6 | 1.04 (1.01; 1.07) | 15 | 7 | 1.00 (0.96; 1.04) | 12 | 2 | 1.10 (1.02; 1.18) | 43 | - | - | - |
| **Trans-fatty acids** | | | | | | | | | | | |
| *Trans-Elaidic acid (t-18:1n–9)* | | | | | | | | | | | |
| 8 | 0.99 (0.98; 1.01) | 0 | 4 | 0.94 (0.87; 1.01) | 88 | - | - | - | - | - | - |
| *Trans-Hypogeic acid (t-16:1n–9)* | | | | | | | | | | | |
| 2 | 0.81 (0.44; 1.49) | 0 | 4 | 0.72 (0.34; 1.51) | 48 | - | - | - | - | - | - |
| *Trans-Palmitoleic acid (t-16:1n-7)* | | | | | | | | | | | |
| 3 | 0.93 (0.85; 1.02) | 0 | 3 | 0.68 (0.43; 1.01) | 52 | 2 | 1.00 (0.84; 1.19) | 0 | - | - | - |
| *Trans-Linoleic acid (t-18:2)* | | | | | | | | | | | |
| 5 | 0.98 (0.88; 1.08) | 53 | 4 | 0.80 (0.66; 0.98) | 39 | - | - | - | - | - | - |
| **Polyunsaturated fatty acids** | | | | | | | | | | | |
| *Total Polyunsaturated fatty acids* | | | | | | | | | | | |
| - | - | - | - | - | - | - | 0.96 (0.81; 1.14) | 90 | - | - | - |
| *Total n-6 Polyunsaturated fatty acids* | | | | | | | | | | | |
| 2 | 0.97 (0.89; 1.06) | 0 | - | - | - | 2 | 0.97 (0.78; 1.21) | 94 | - | - | - |
| *Linolenic acid (18:2)* | | | | | | | | | | | |
| 9 | 0.92 (0.87; 0.98) | 66 | 7 | 0.93 (0.91; 0.96) | 51 | 8 | 0.96 (0.93; 0.99) | 87 | 4 | 0.96 (0.94; 0.97) | 0 |
| *γ-Linolenic acid (γ-18:3)* | | | | | | | | | | | |
| 2 | 2.58 (1.79; 3.72) | 0 | - | - | - | - | - | - | - | - | - |
| *Arachidonic acid (20:4)* | | | | | | | | | | | |
| 9 | 0.96 (0.92; 1.01) | 52 | 7 | 1.02 (1.00; 1.04) | 0 | 6 | 0.92 (0.85; 0.99) | 57 | 3 | 1.04 (0.98; 1.10) | 0 |
| *Total n-3 Polyunsaturated fatty acids* | | | | | | | | | | | |
| - | - | - | - | - | - | 2 | 0.70 (0.52; 0.94) | 71 | - | - | - |
| *α-Linolenic acid (α-18:3)* | | | | | | | | | | | |
| 7 | 1.10 (1.02; 1.18) | 0 | 7 | 0.95 (0.88; 1.03) | 31 | 7 | 1.01 (0.98; 1.04) | 54 | 3 | 0.98 (0.94; 1.03) | 0 |
| *Eicosapentaenoic acid (20:5)* | | | | | | | | | | | |
| 7 | 0.98 (0.96; 1.00) | 26 | 8 | 1.00 (0.98; 1.01) | 39 | 7 | 0.99 (0.98; 1.00) | 0 | 3 | 0.99 (0.98; 1.01) | 0 |
| *Docosapentaenoic acid (22:5)* | | | | | | | | | | | |
| 7 | 0.98 (0.96; 0.99) | 0 | 8 | 0.97 (0.94; 1.01) | 53 | 6 | 0.96 (0.91; 1.01) | 77 | - | - | - |
| *Docosahexaenoic acid (22:6)* | | | | | | | | | | | |
| 7 | 0.99 (0.93; 1.05) | 26 | 8 | 0.92 (0.88; 0.96) | 0 | 8 | 0.78 (0.65; 0.93) | 87 | 3 | 0.92 (0.65; 1.31) | 0 |
| *Eicosapentaenoic acid (20:5) + Docosapentaenoic acid (22:5) + Docosahexaenoic acid (22:6)* | | | | | | | | | | | |
| 6 | 0.95 (0.91; 0.99) | 0 | 8 | 0.94 (0.91; 0.96) | 0 | 7 | 0.94 (0.91; 0.97) | 0 | 3 | 0.95 (0.86; 1.05) | 0 |
| CI: confidence interval, SRR: summary relative risk | | | | | | | | | | | |


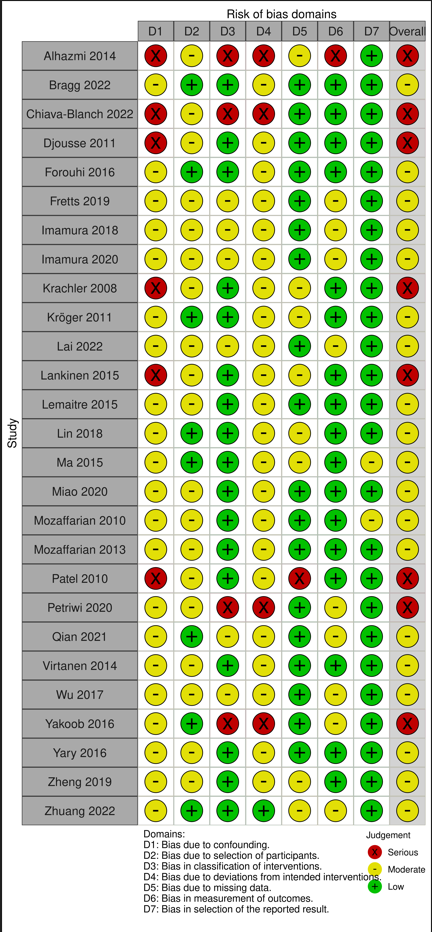


**Supplemental Figure 1.** Results of the evaluation of the risk of bias for each included study using ROBINS-I (Risk Of Bias In Non-randomised Studies - of Interventions) tool.

**Supplemental Figure 2.1-2.34**. Results of linear dose-response meta-analyses of prospective cohort studies on the association between specific fatty acids (% of total fatty acids) and risk of type 2 diabetes in all biospecimens and in subgroups of specific biospecimens of the fatty acids measurement:

1a) Total saturated fatty acids (SFAs) in all biospecimens

1b) Total saturated fatty acids (SFAs) across different biospecimens


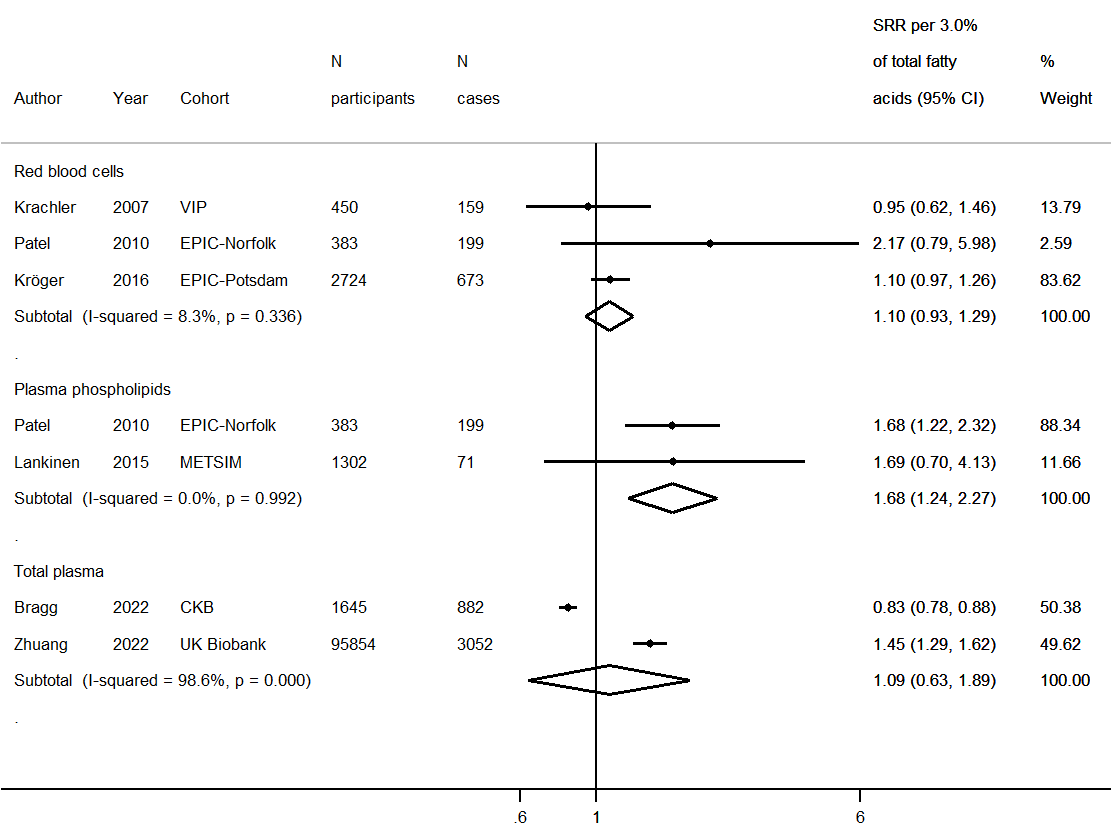


2a) Myristic acid (14:0) in all biospecimens

2b) Myristic acid (14:0) across different biospecimens


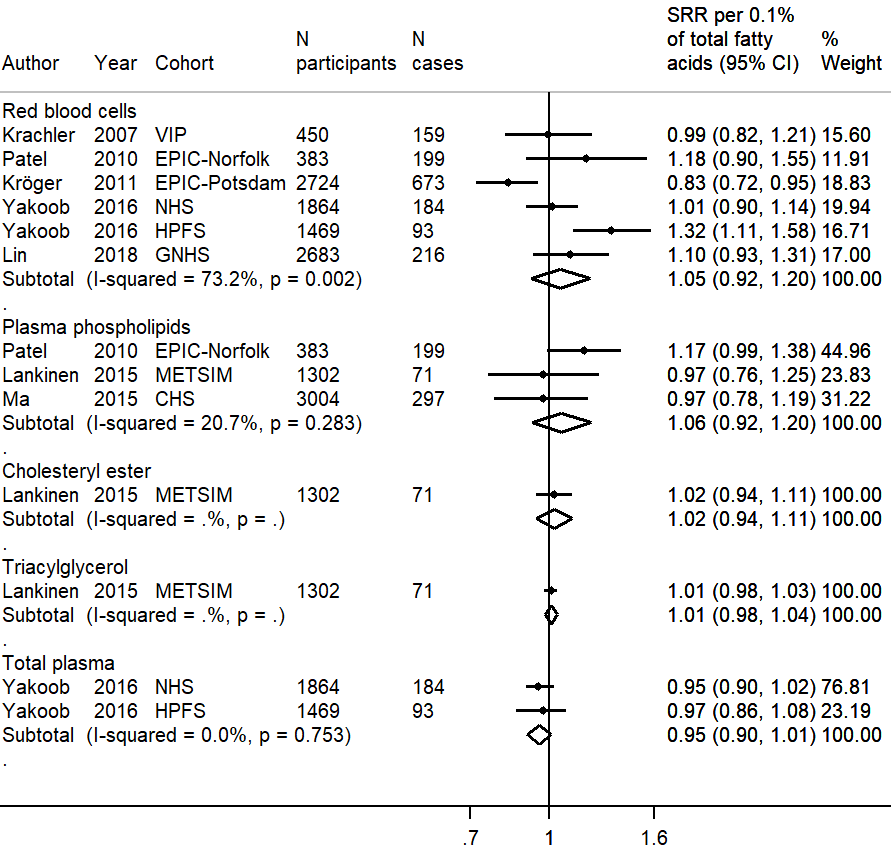


3a) Pentadecanoic acid (15:0) in all biospecimens


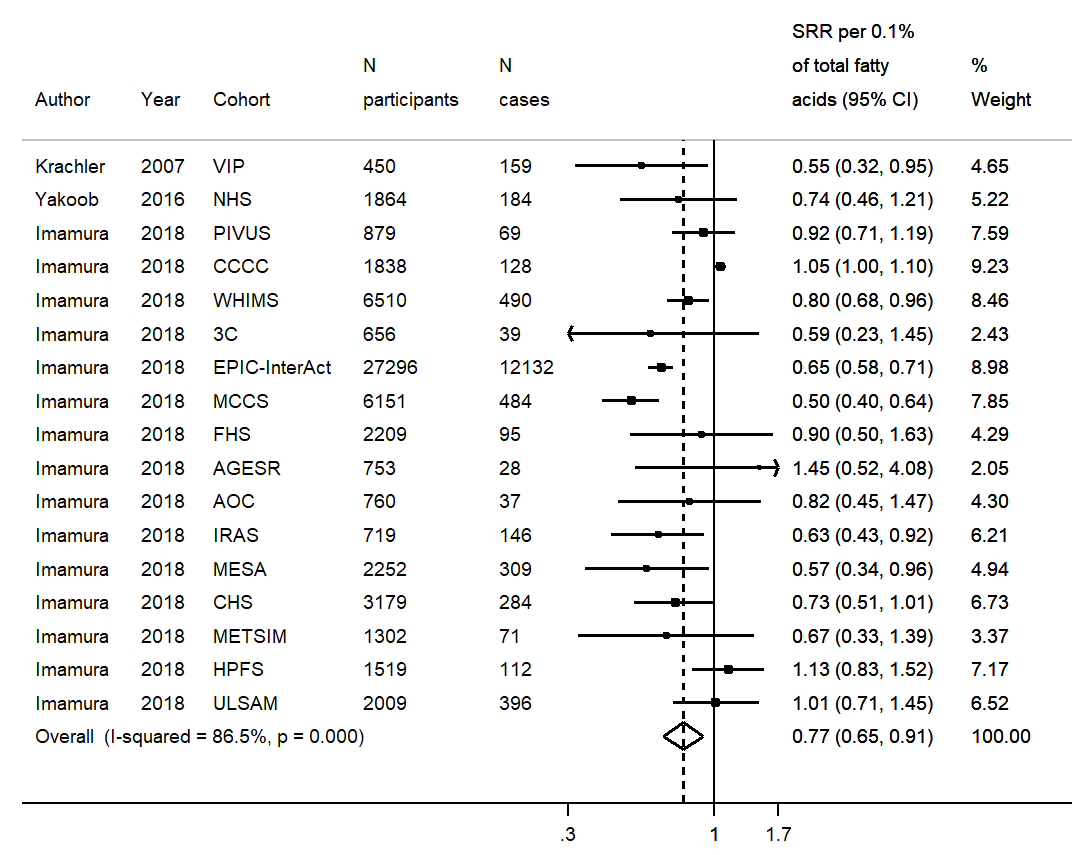


3b) Pentadecanoic acid (15:0) across different biospecimens


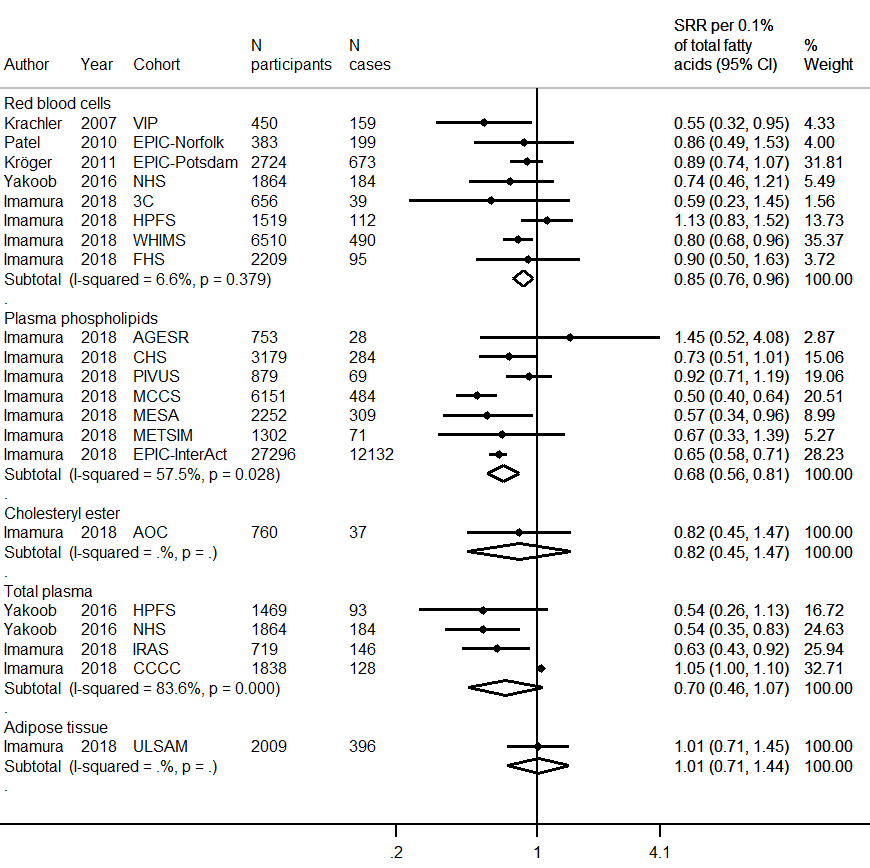


4a) Palmitic acid (16:0) in all biospecimens

4b) Palmitic acid (16:0) across different biospecimens


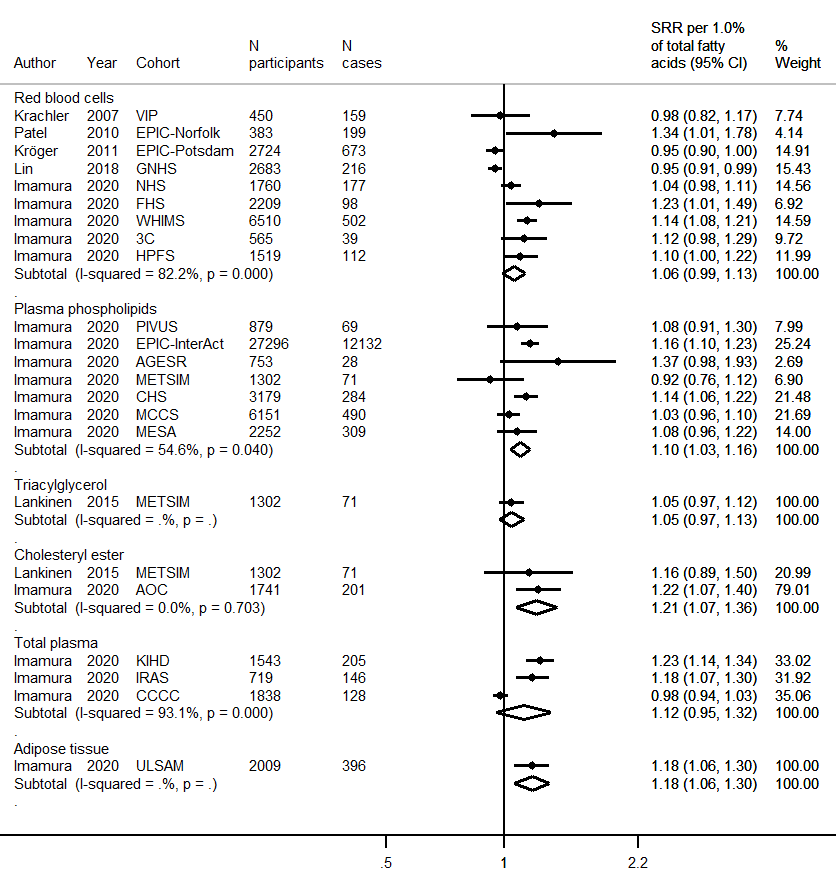


5a) Margaric acid (17:0) in all biospecimens

5b) Margaric acid (17:0) across different biospecimens


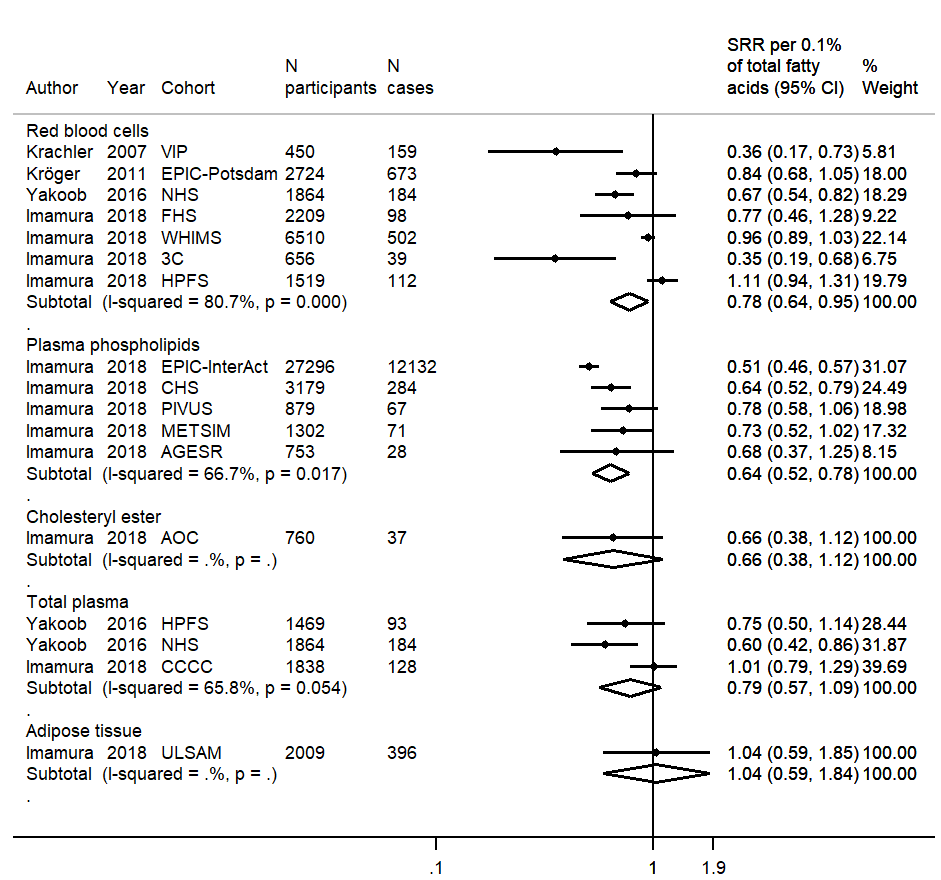


6a) Stearic acid (18:0) in all biospecimens


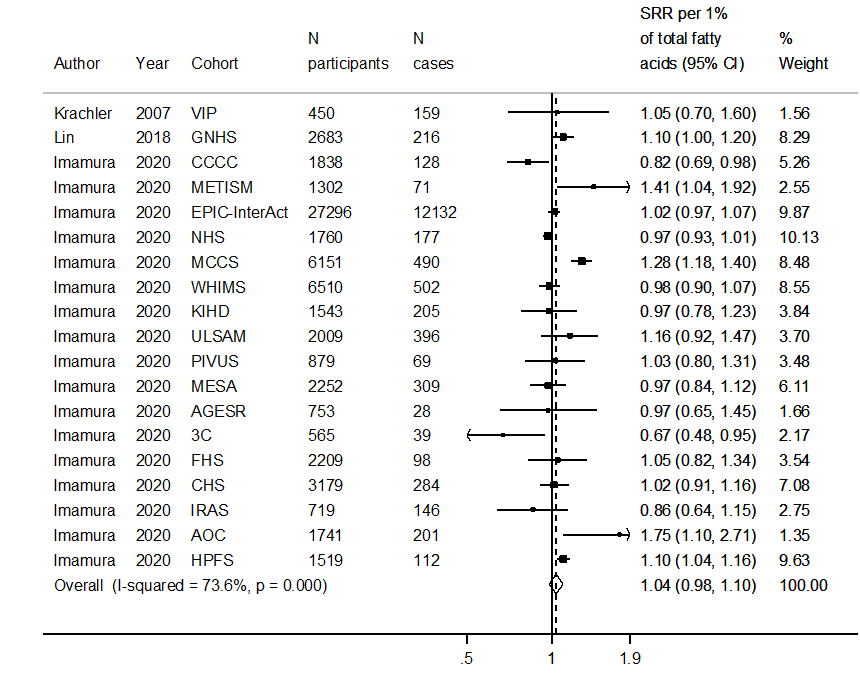


6b) Stearic acid (18:0) across different biospecimens


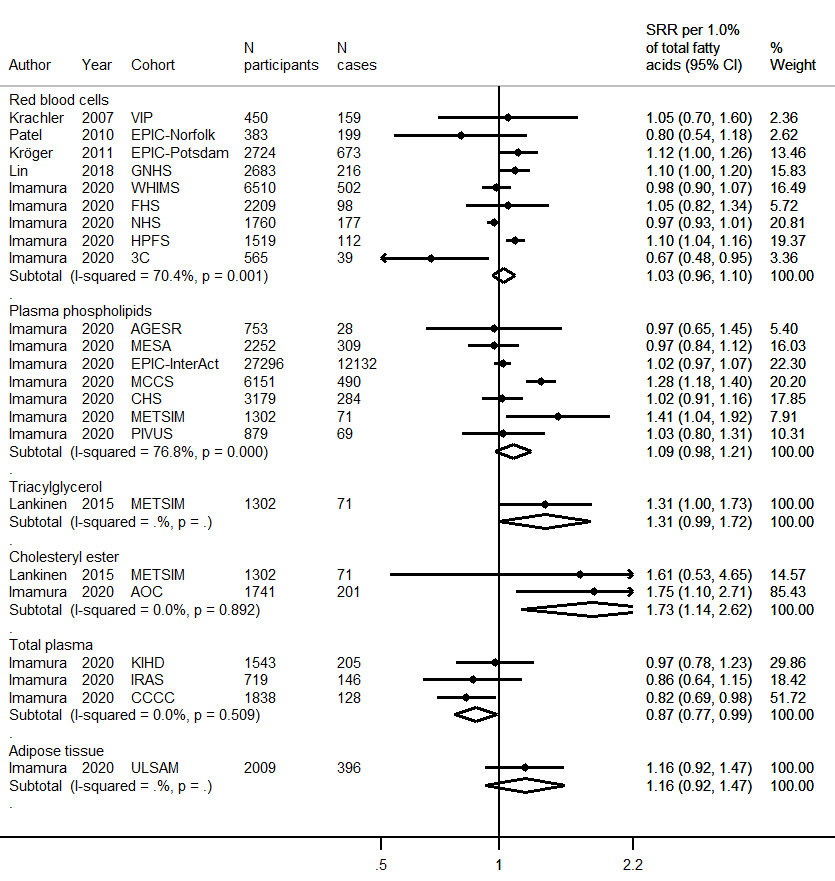


7a) Arachidic acid (20:0) in all biospecimens

7b) Arachidic acid (20:0) across different biospecimens


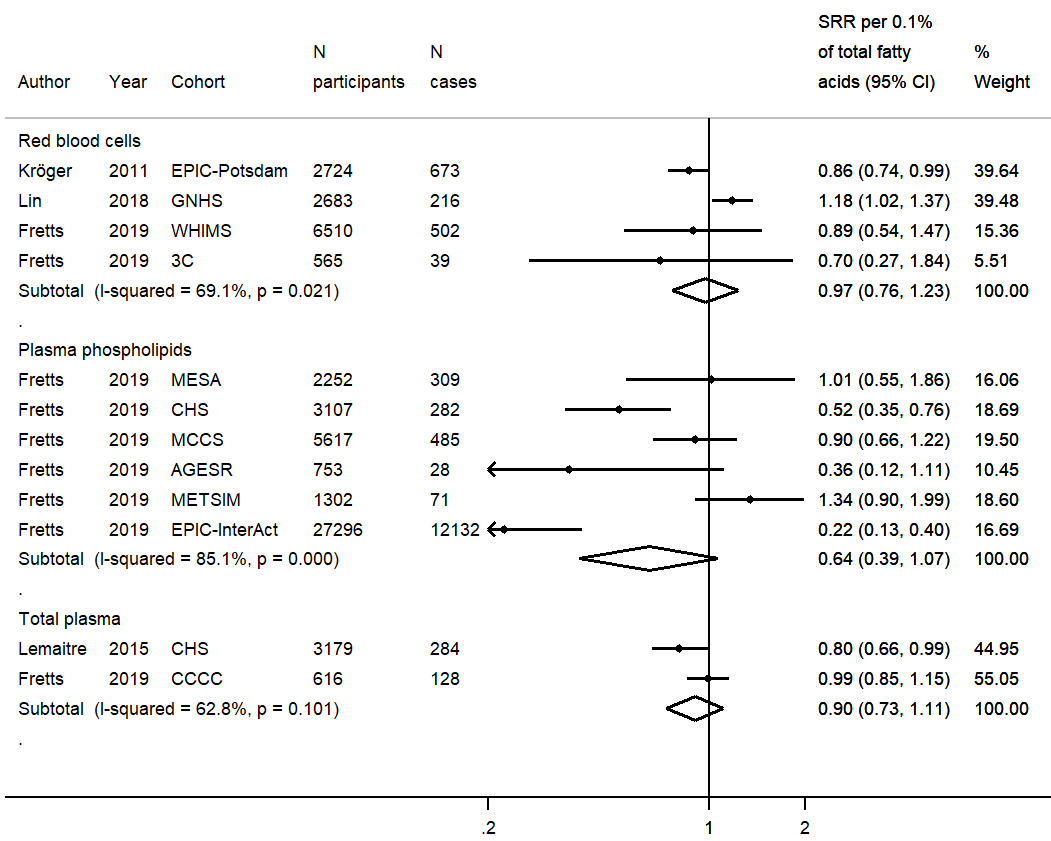


8a) Behenic acid (22:0) in all biospecimens


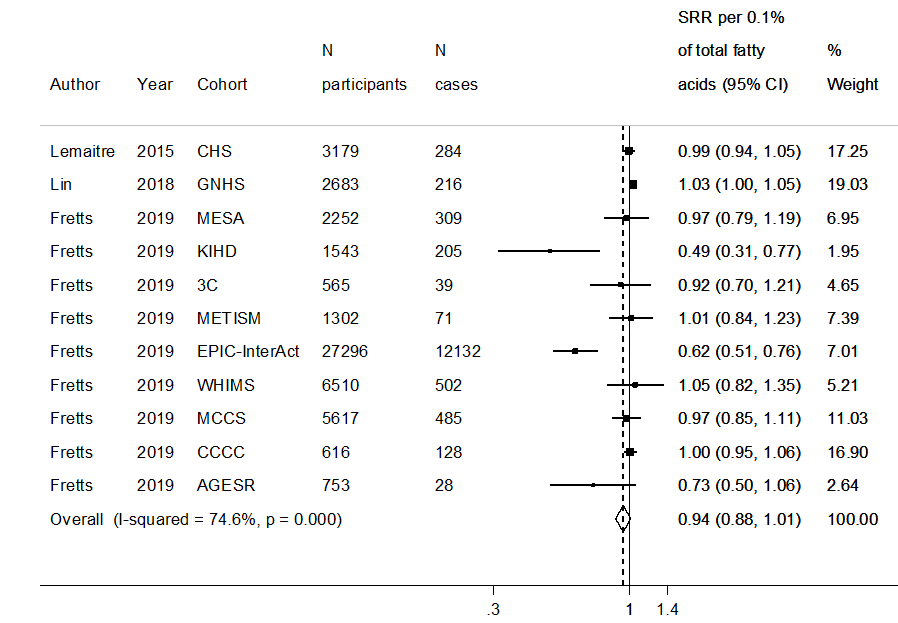


8b) Behenic acid (22:0) across different biospecimens


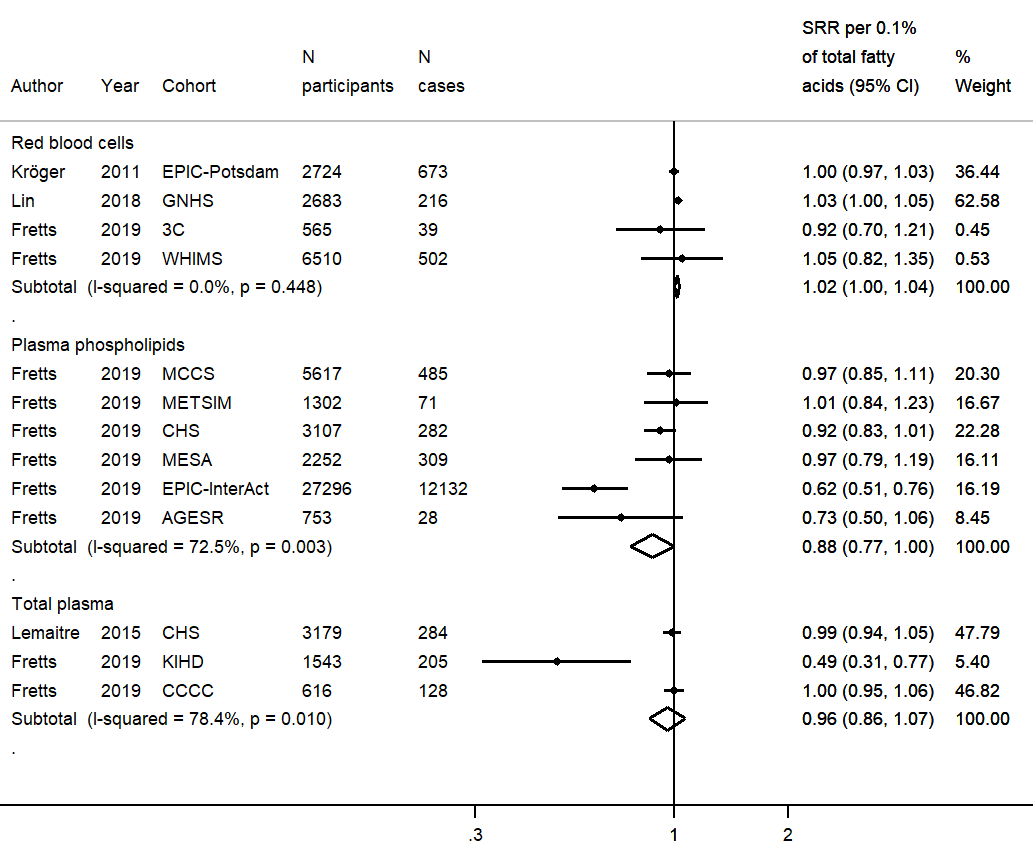


9a) Lignoceric acid (24:0) in all biospecimens

9b) Lignoceric acid (24:0) across different biospecimens


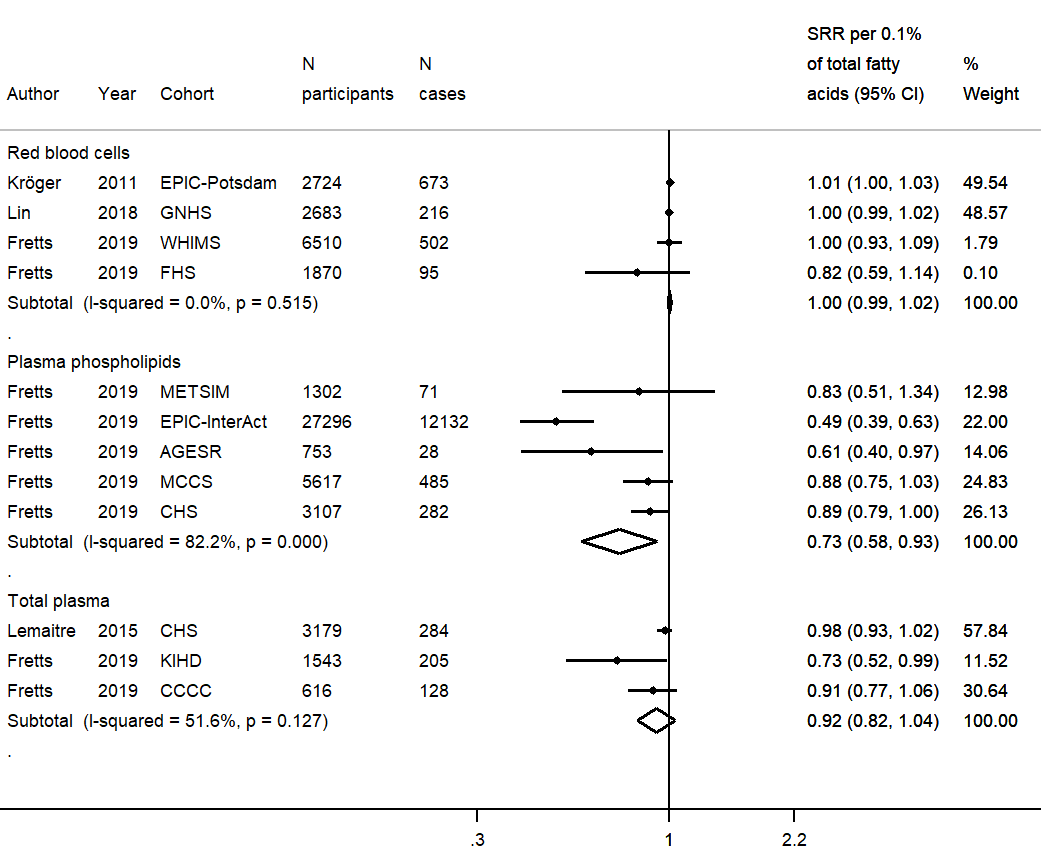


10a) Total monounsaturated fatty acids (MUFAs) in all biospecimens

10b) Total monounsaturated fatty acids (MUFAs) across different biospecimens


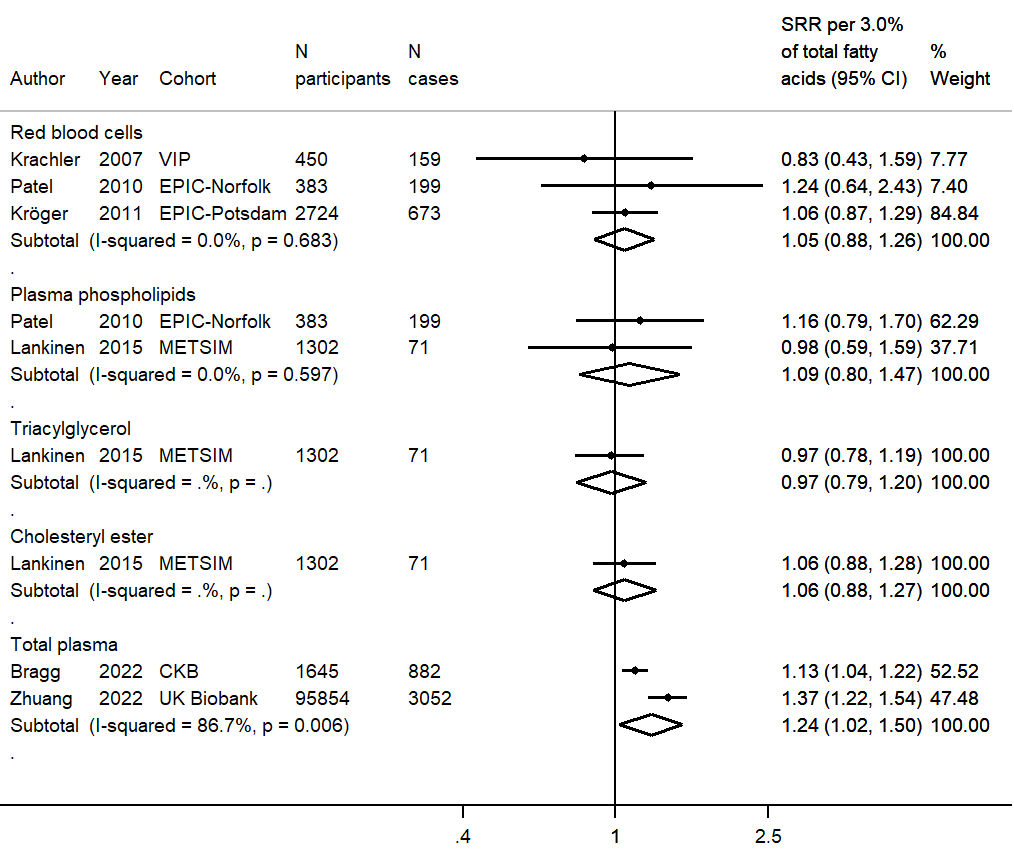


11a) Palmitoleic acid (16:1n-7) in all biospecimens

11b) Palmitoleic acid (16:1n-7) across different biospecimens


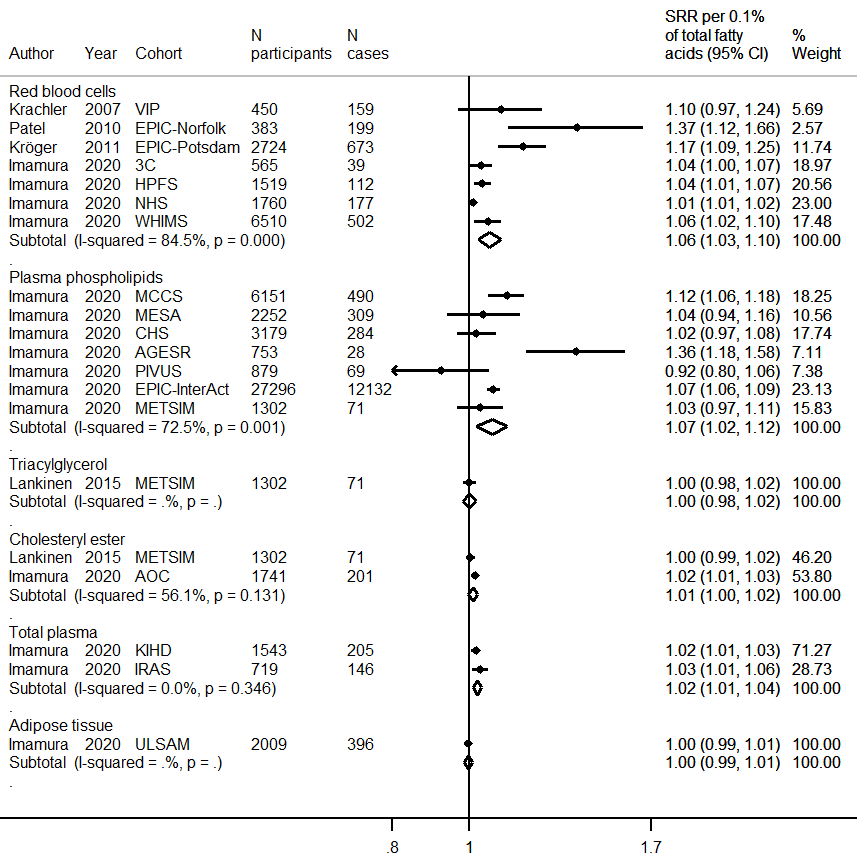


12) Palmitelaidic (16:1n-9) in all biospecimens

13a) Oleic acid (18:1n-9) in all biospecimens

13b) Oleic acid (18:1n-9) across different biospecimens


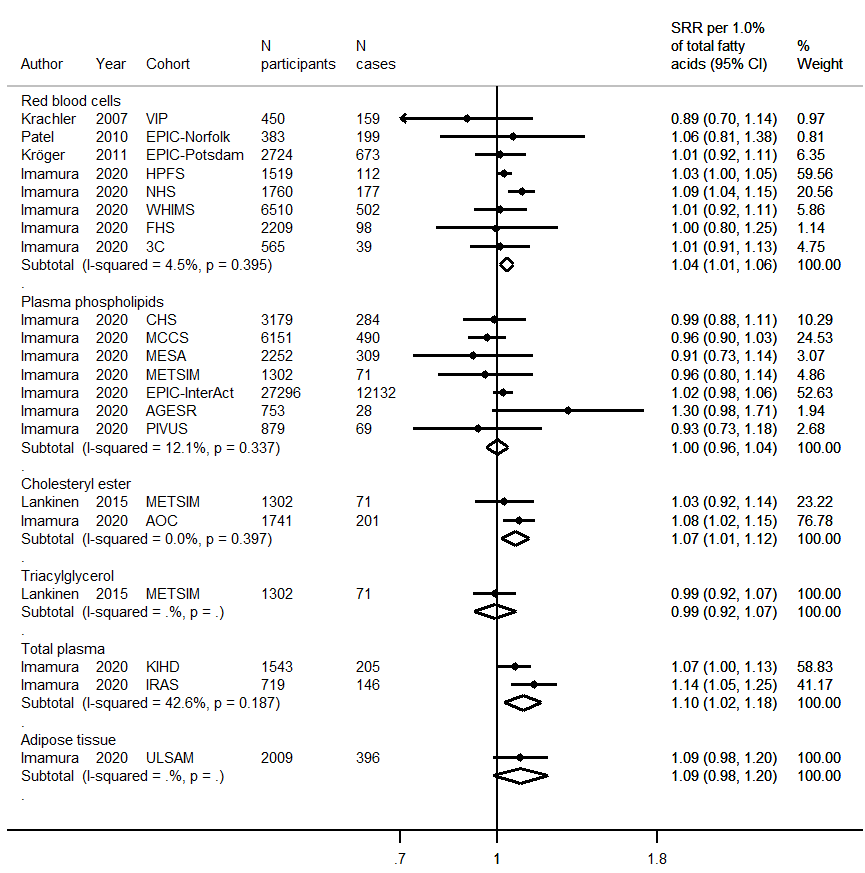


14a) Eicosenoic acid (20:1) in all biospecimens

14b) Eicosenoic acid (20:1) across different biospecimens


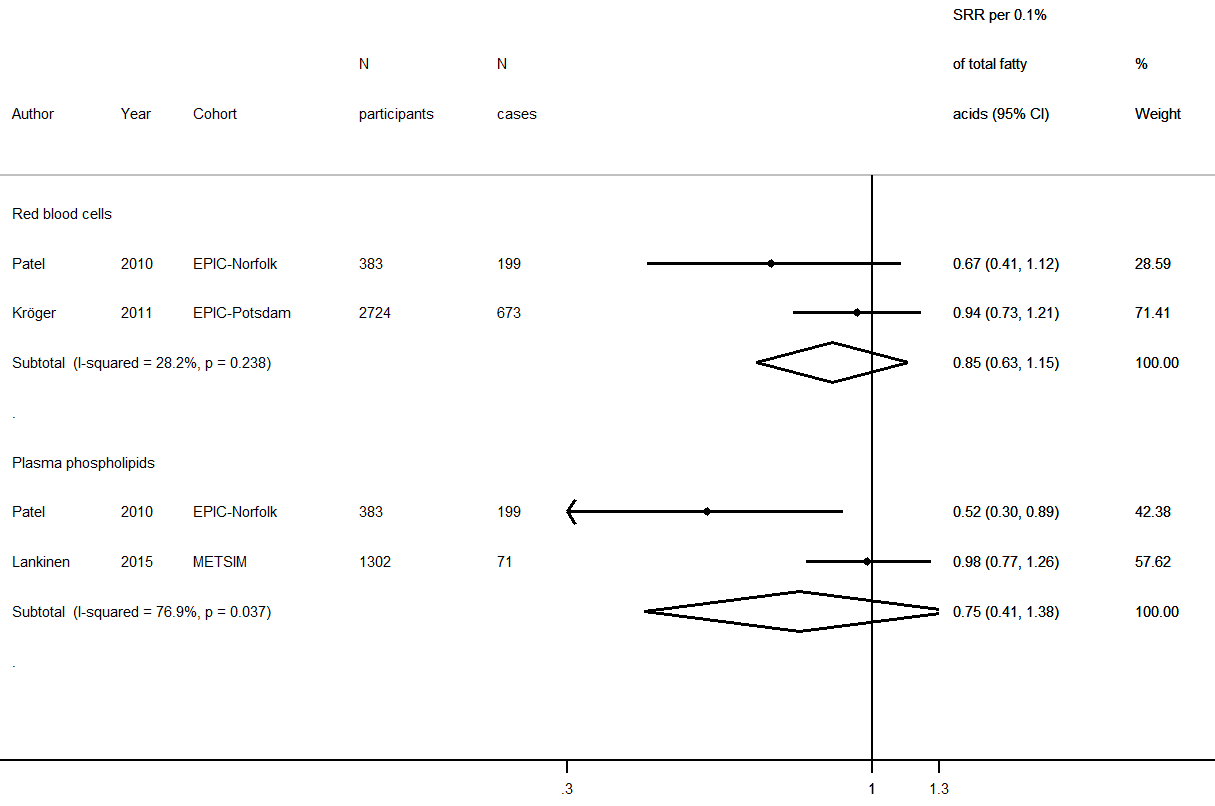


15a) Nervonic acid (24:1) in all biospecimens

15b) Nervonic acid (24:1) across different biospecimens


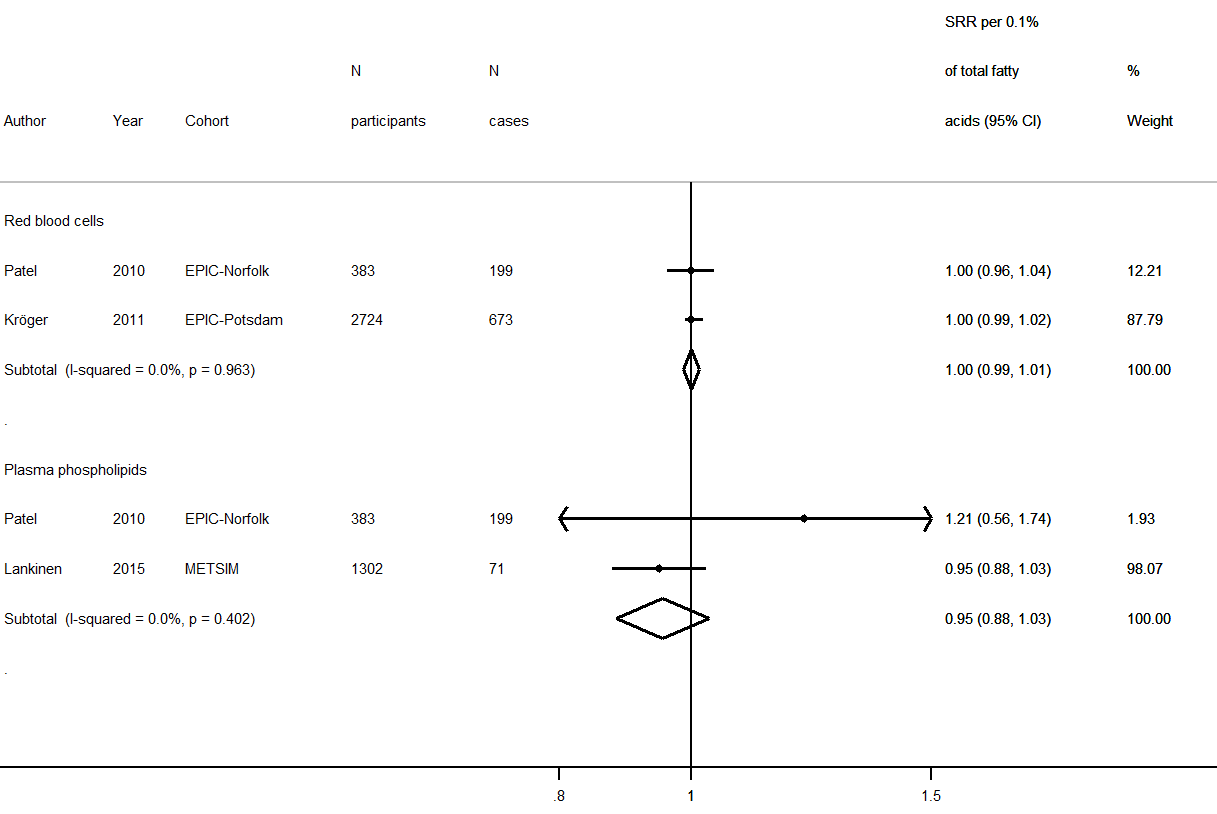


16a) *Trans*-Vaccenic acid (*t-*18:1n-7) in all biospecimens


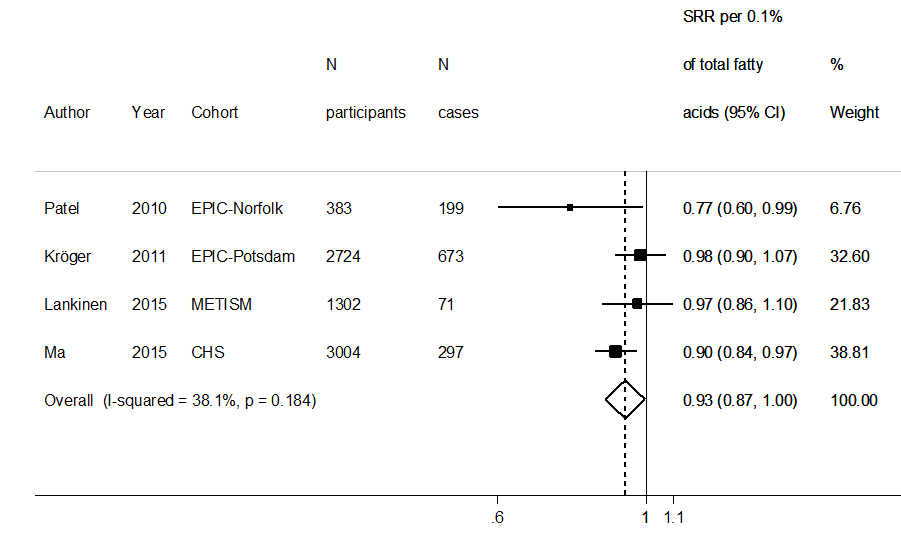


16b) *Trans*-Vaccenic acid (*t-*18:1n-7) across different biospecimens


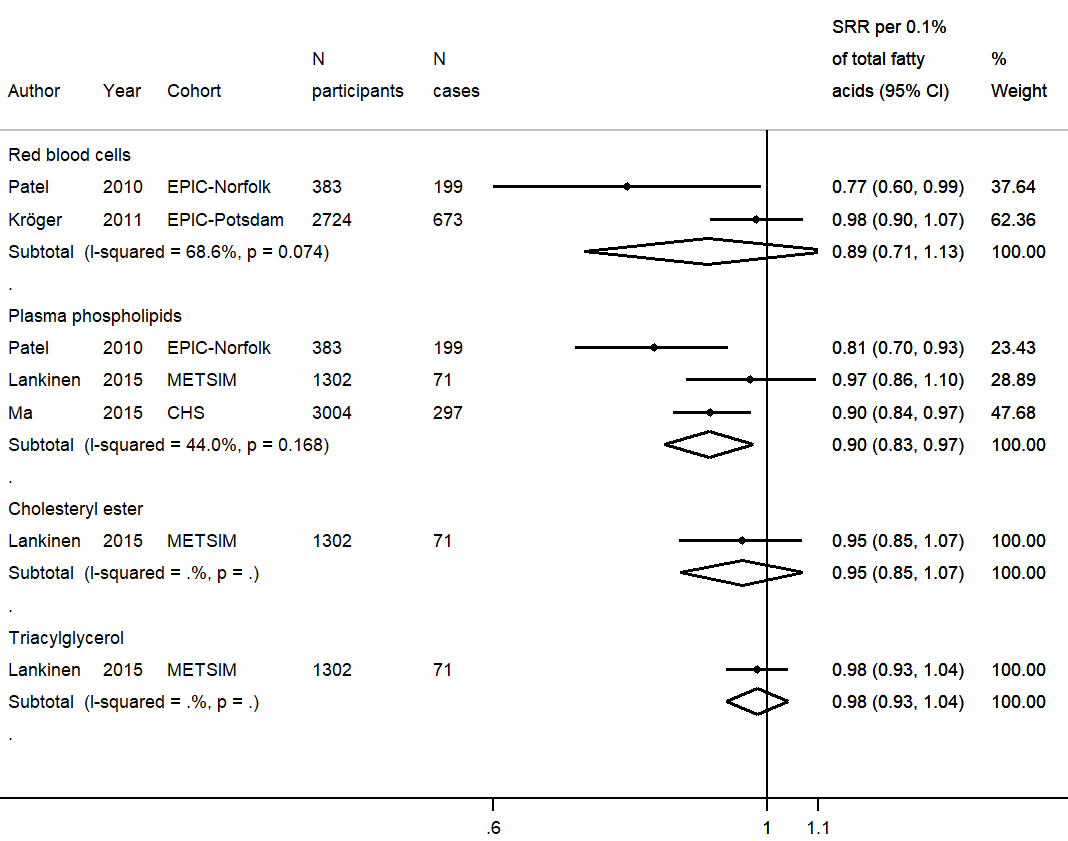


17a) *Trans*-Palmitoleic acid (*t*-16:1n–7) in all biospecimens

17b) *Trans*-Palmitoleic acid (*t*-16:1n–7) across different biospecimens


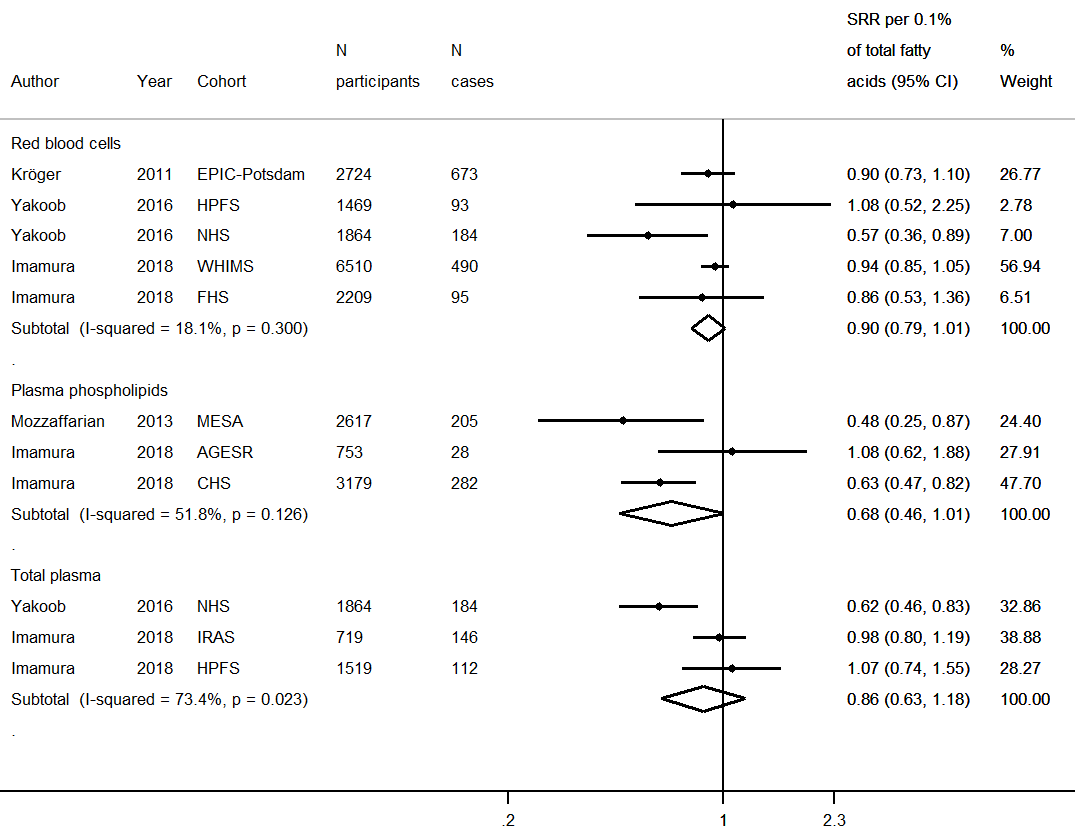


18a) *Trans*-Hypogeic acid (*t*-16:1n-9) in all biospecimens

18b) *Trans*-Hypogeic acid (*t*-16:1n-9) across different biospecimens


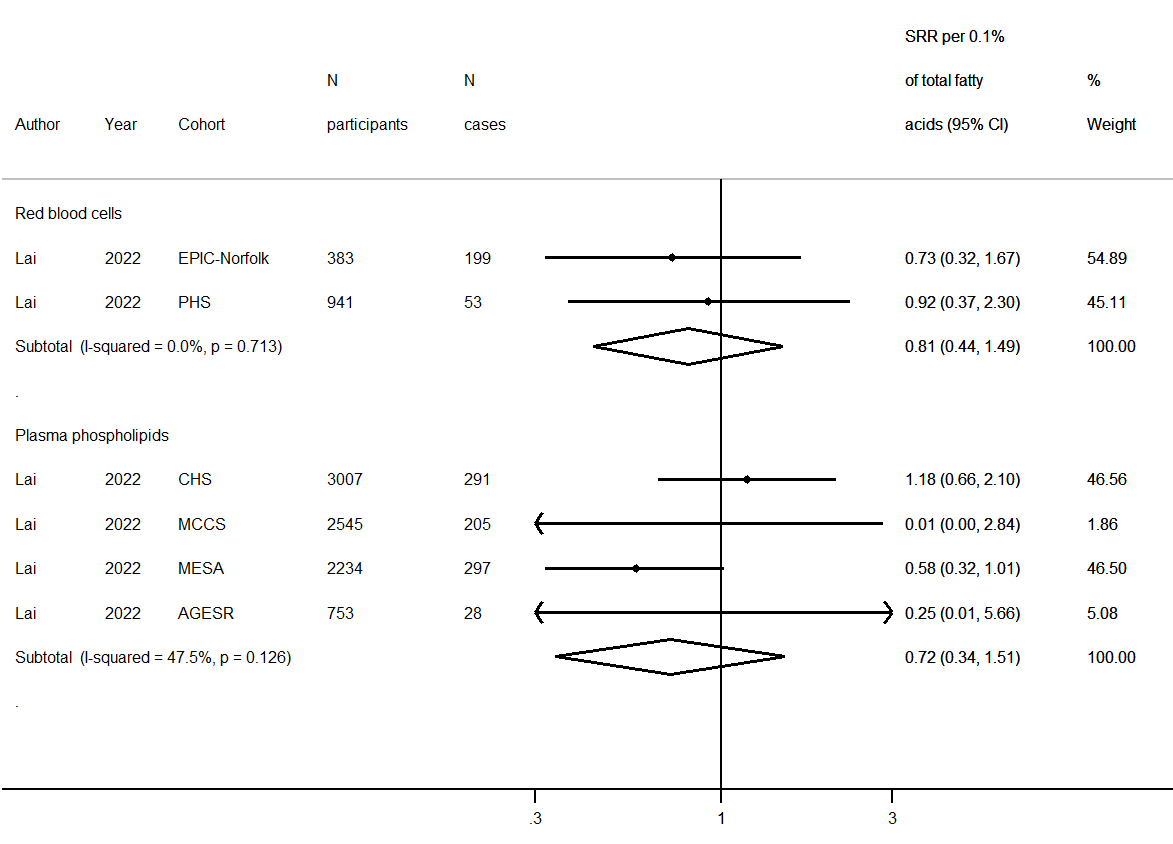


19a) *Trans*-Elaidic acid (*t-*18:1n–9) in all biospecimens


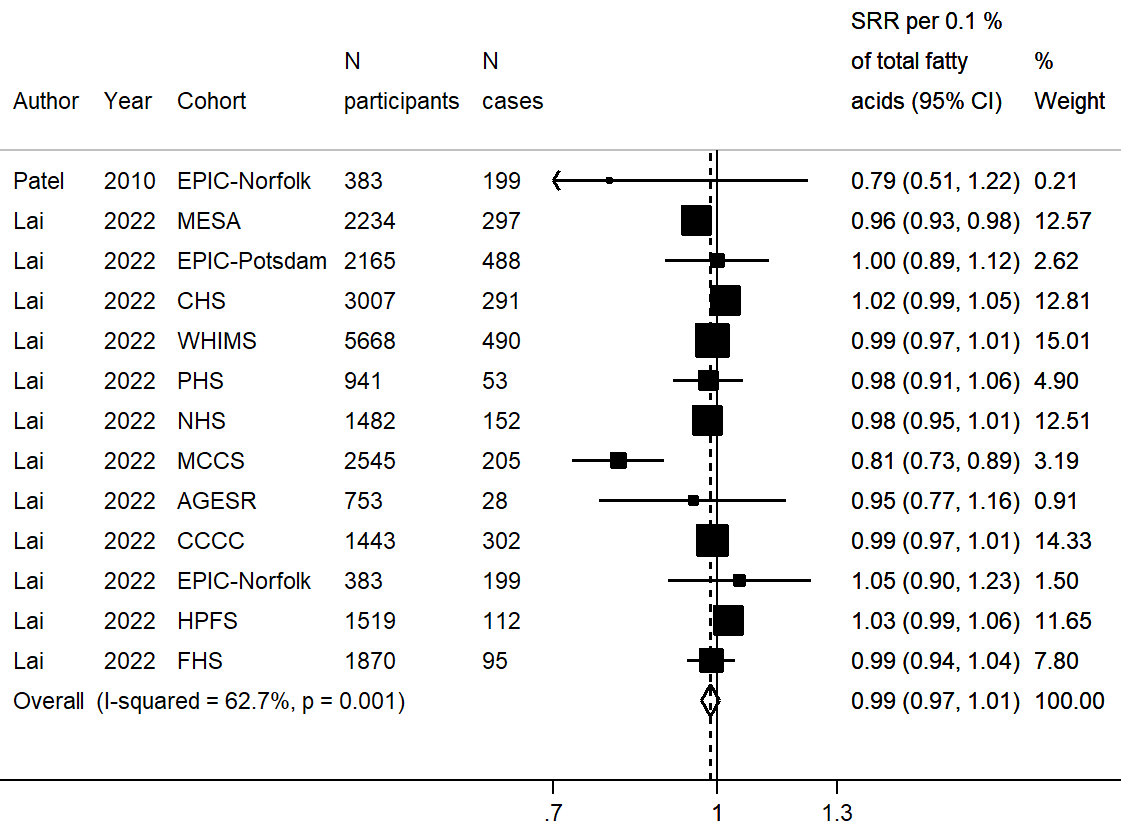


19b) *Trans*-Elaidic acid (*t*-18:1n–9) across different biospecimens


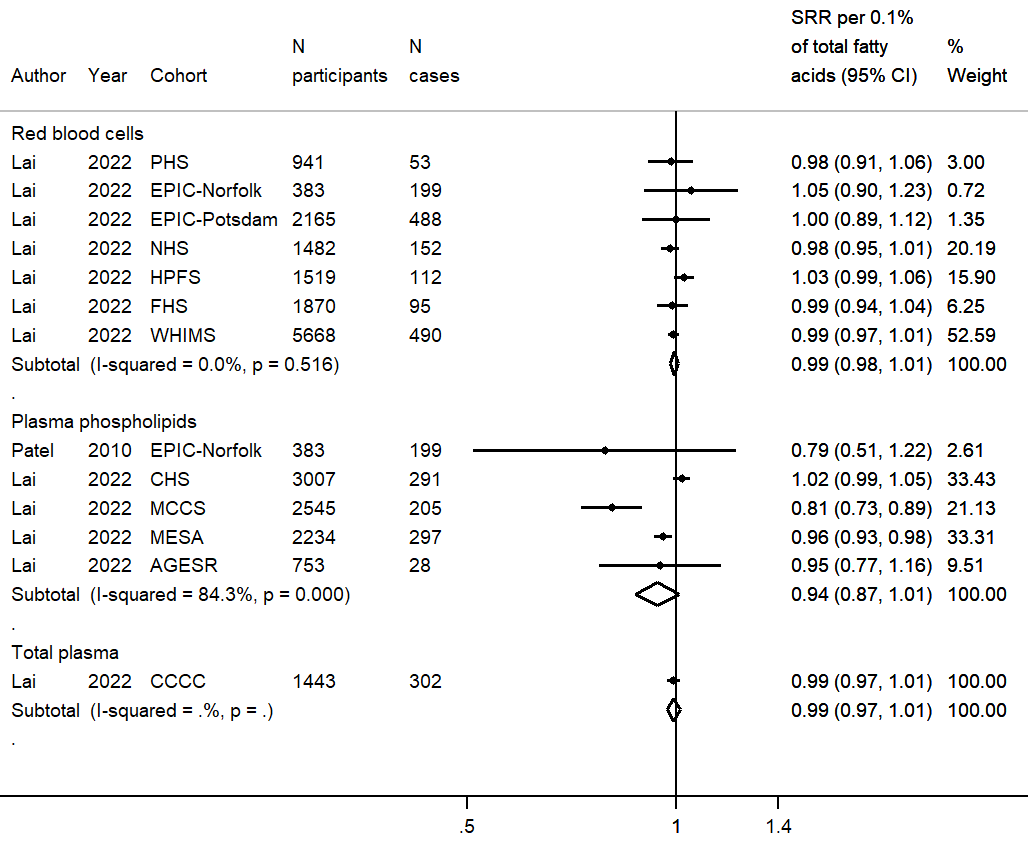


20a) *Trans*-Linoleic acid (*t-*18:2) in all biospecimens

20b) *Trans*-Linoleic acid (*t*-18:2) across different biospecimens


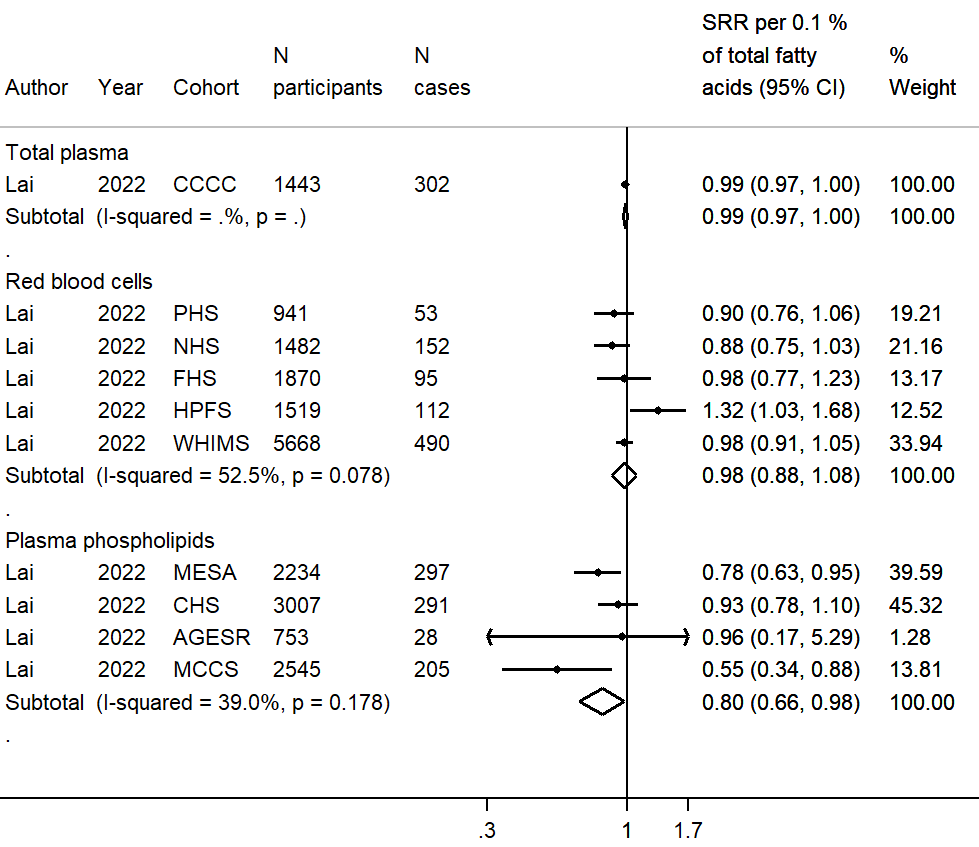


21a) Total polyunsaturated fatty acids (PUFAs) in all biospecimens

21b) Total polyunsaturated fatty acids (PUFAs) across different biospecimens


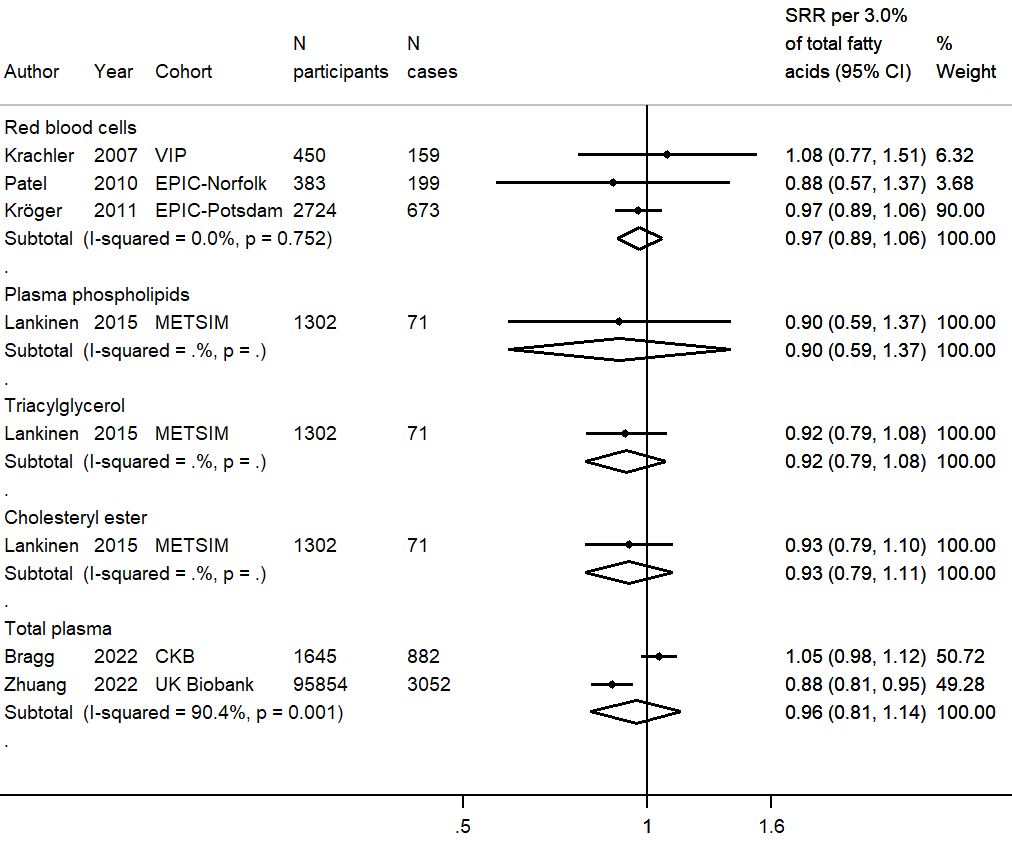


22a) Total n-6 polyunsaturated fatty acids (PUFAs) in all biospecimens

22b) Total n-6 polyunsaturated fatty acids (PUFAs) across different biospecimens


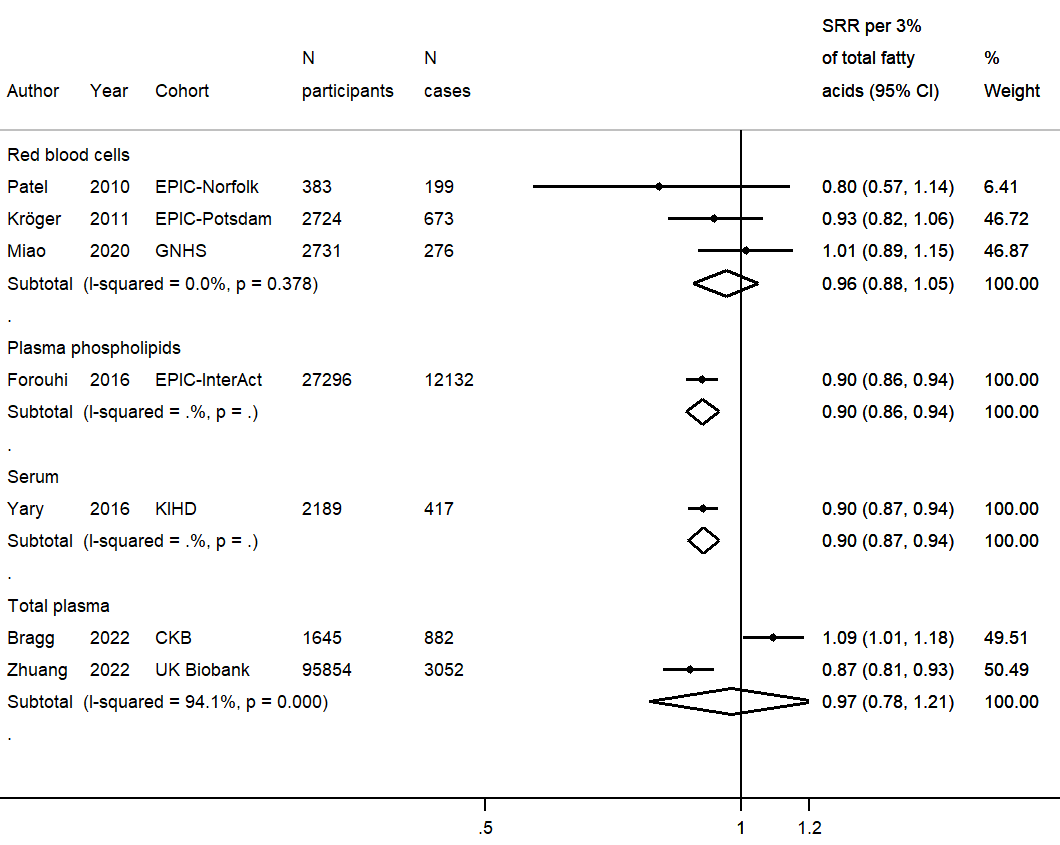


23a) Linoleic acid (18:2) in all biospecimens

23b) Linoleic acid (18:2) across different biospecimens


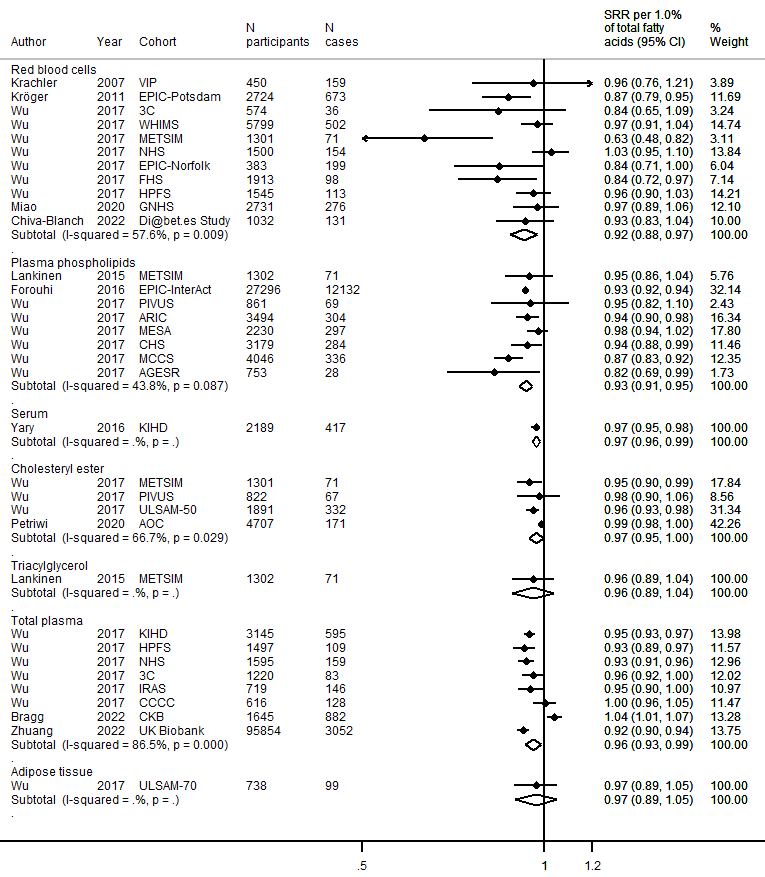


24a) γ-Linolenic acid (γ-18:3) in all biospecimens

24b) γ-Linolenic acid (γ-18:3) across different biospecimens


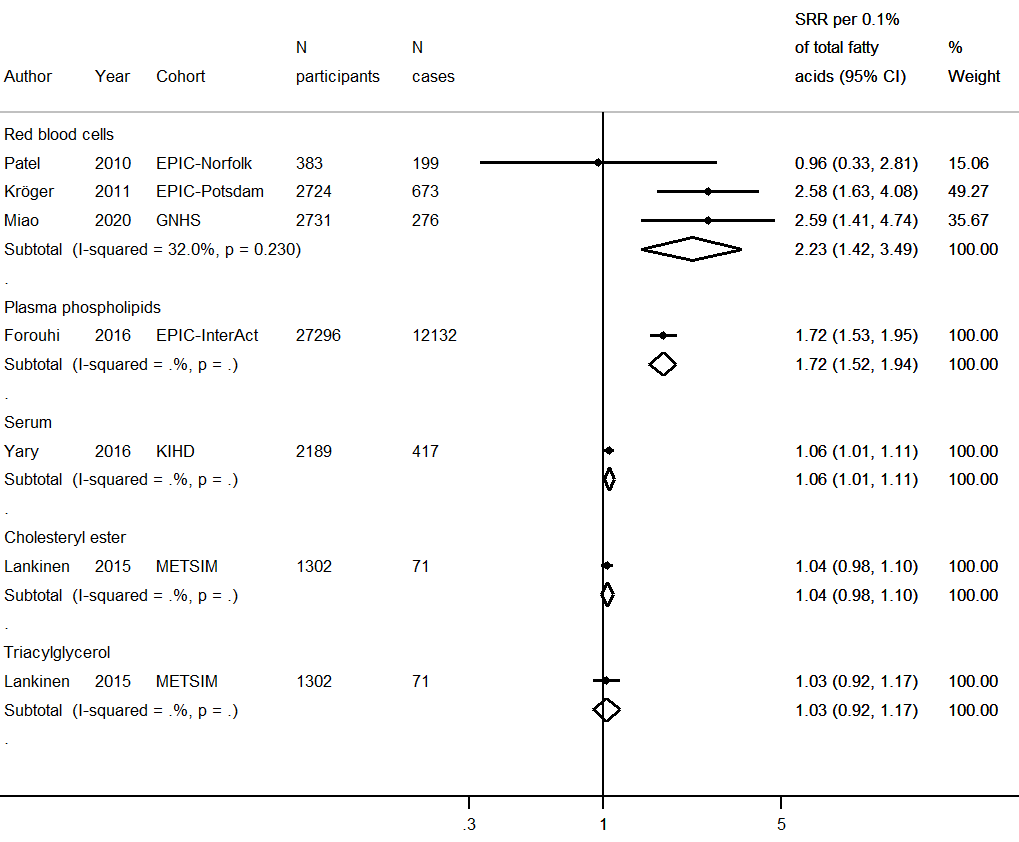


25a) Dihomo-γ-linolenic acid (γ-20:3) in all biospecimens

25b) Dihomo-γ-linolenic acid (γ-20:3) across different biospecimens


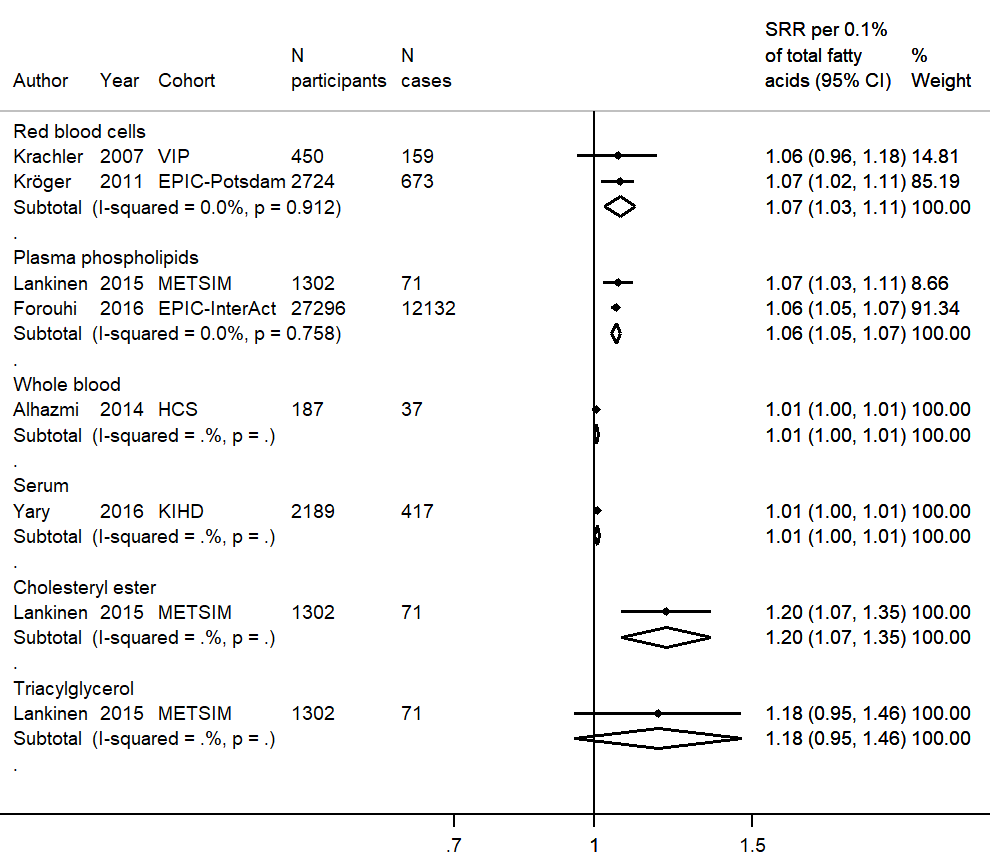


26a) Arachidonic acid (20:4) in all biospecimens

26b) Arachidonic acid (20:4) across different biospecimens


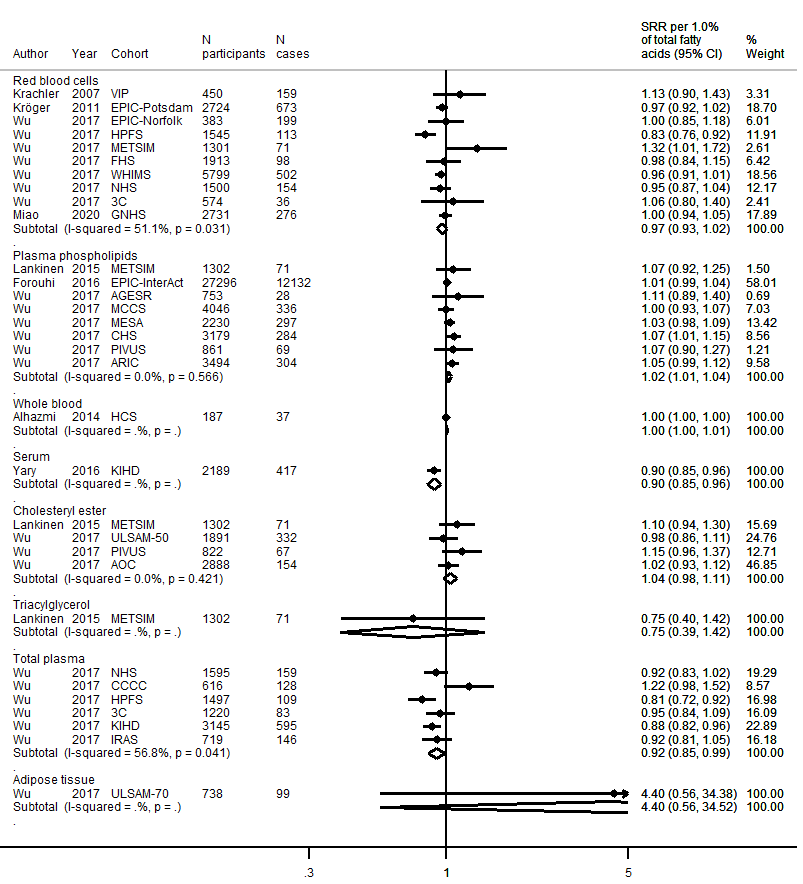


27a) Docosatetraenoic acid (22:4) in all biospecimens

27b) Docosatetraenoic acid (22:4) across different biospecimens


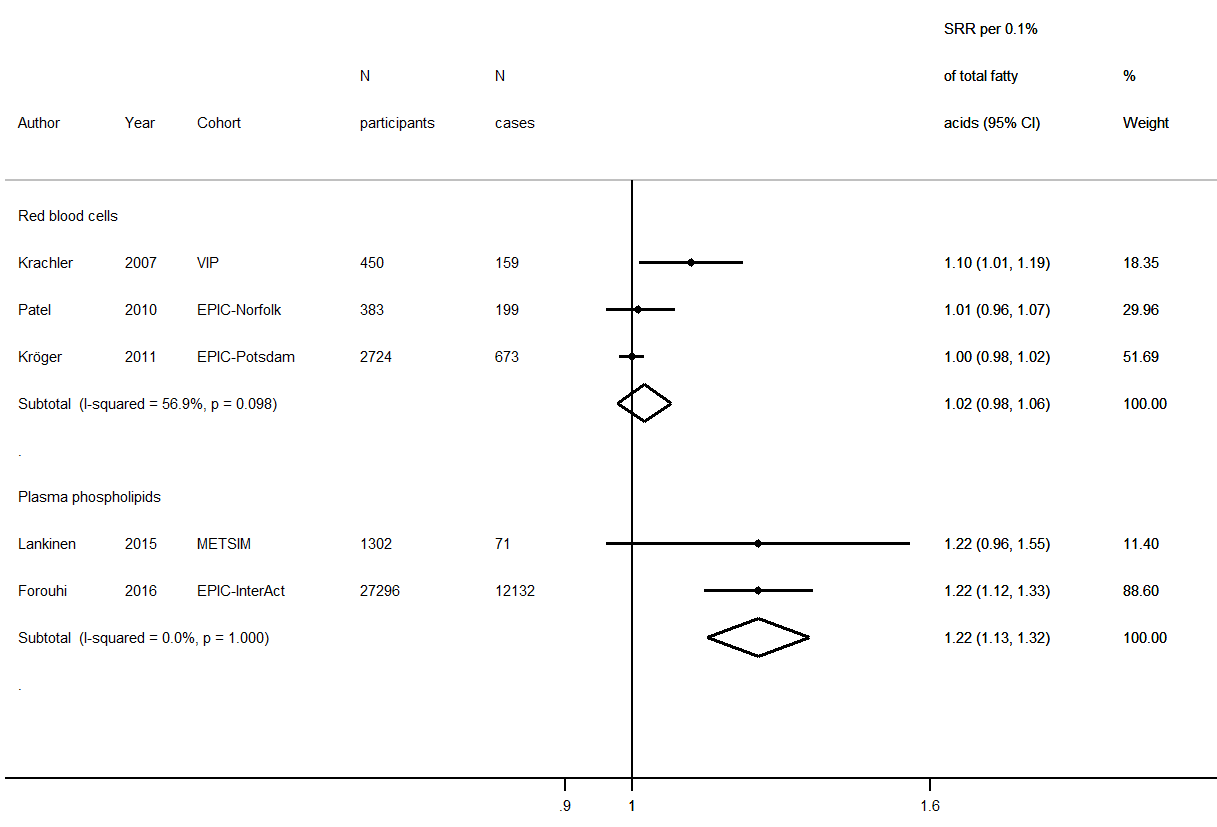


28a) Total n-3 polyunsaturated fatty acids (PUFAs) in all biospecimens

28b) Total n-3 polyunsaturated fatty acids (PUFAs) across different biospecimens


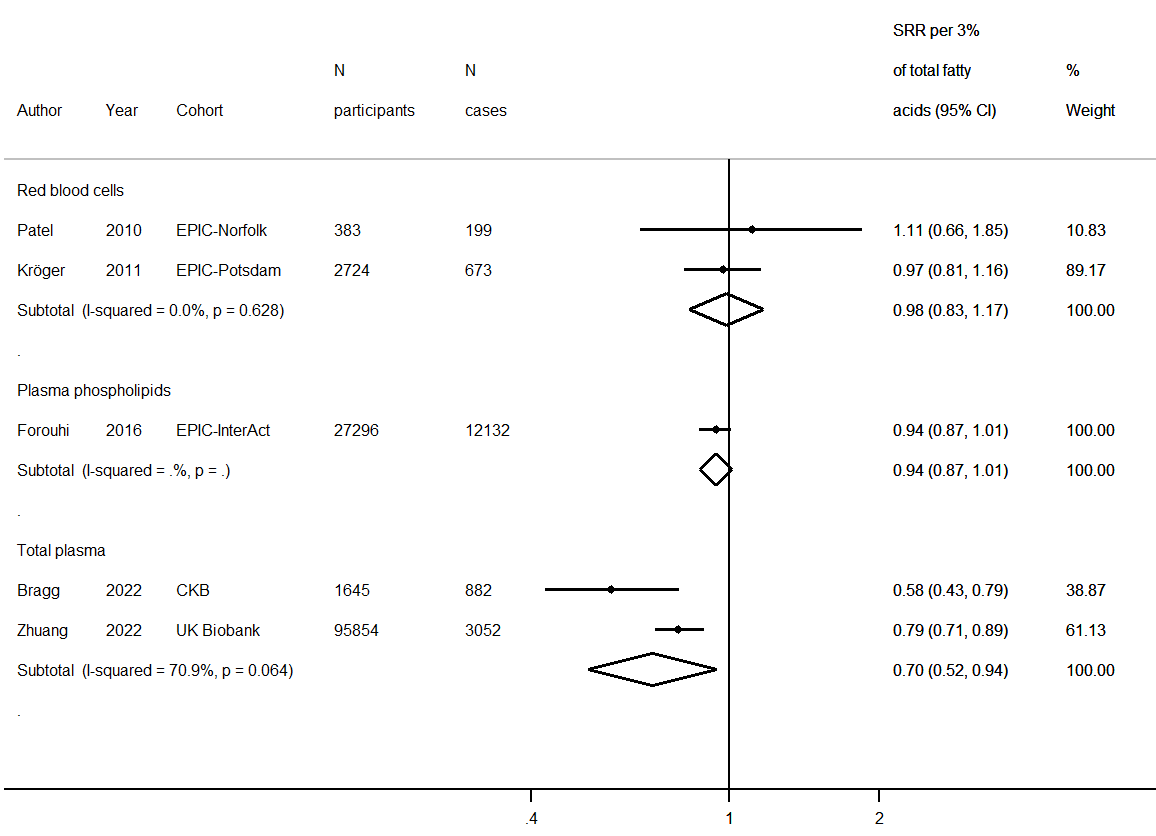


29a) α-Linolenic acid (α-18:3) in all biospecimens

29b) α-Linolenic acid (α-18:3) across different biospecimens


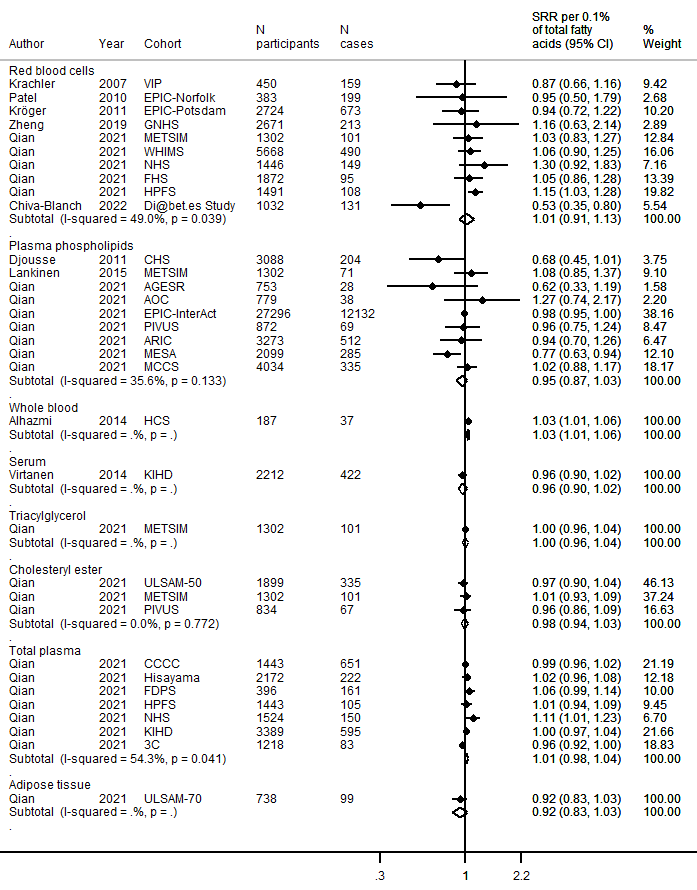


30a) Eicosapentaenoic acid (20:5) in all biospecimens

30b) Eicosapentaenoic acid (20:5) across different biospecimens


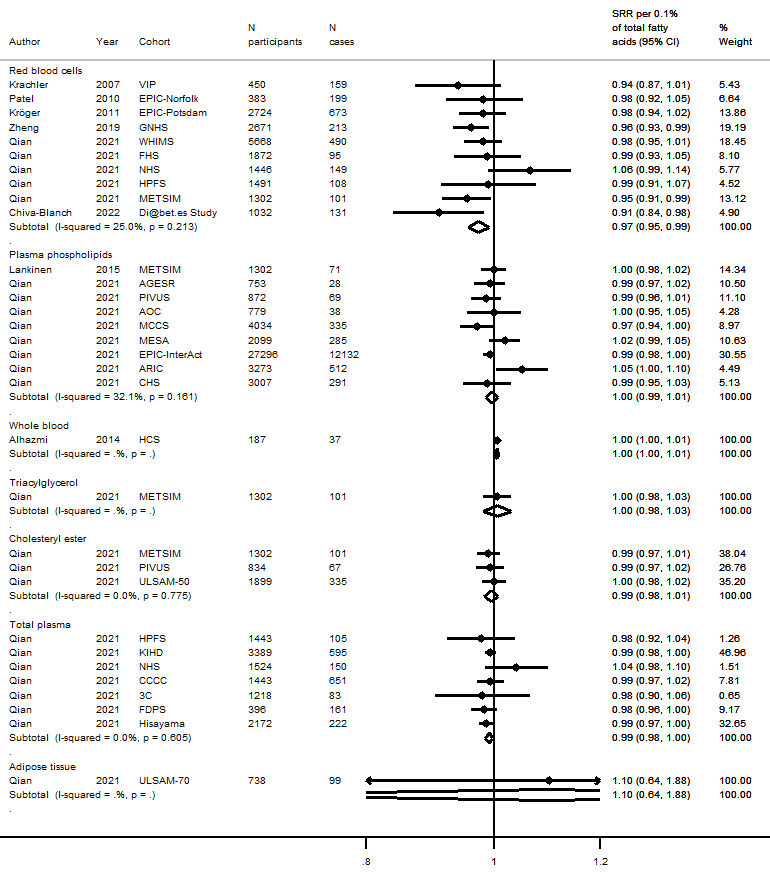


31a) Docosapentaenoic acid (22:5) in all biospecimens

31b) Docosapentaenoic acid (22:5) across different biospecimens


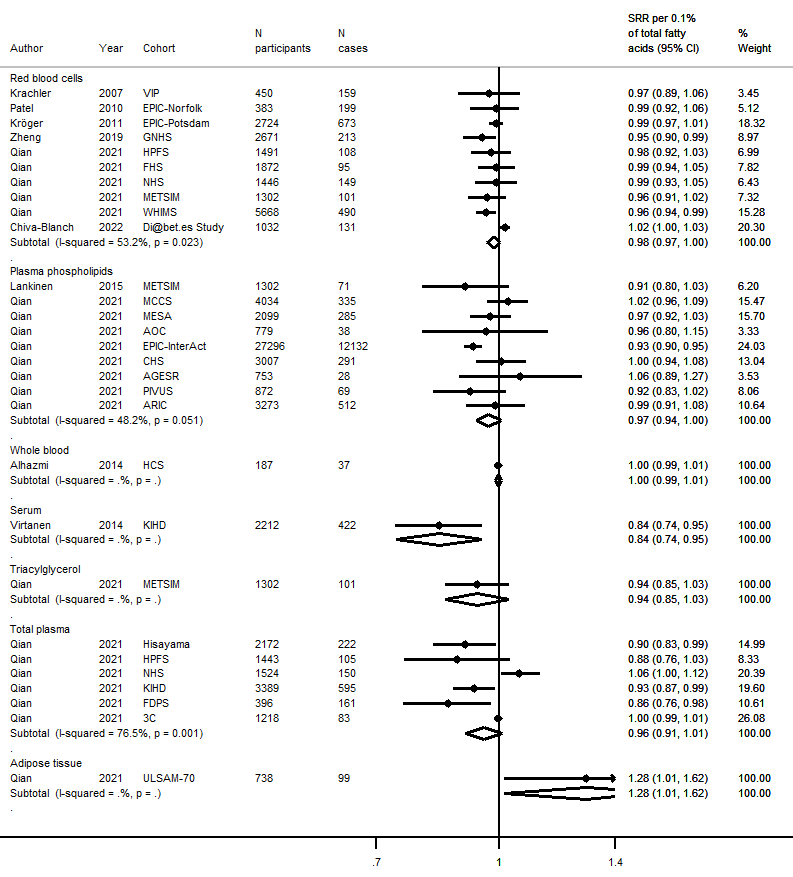


32a) Docosahexaenoic acid (22:6) in all biospecimens

32b) Docosahexaenoic acid (22:6) across different biospecimens


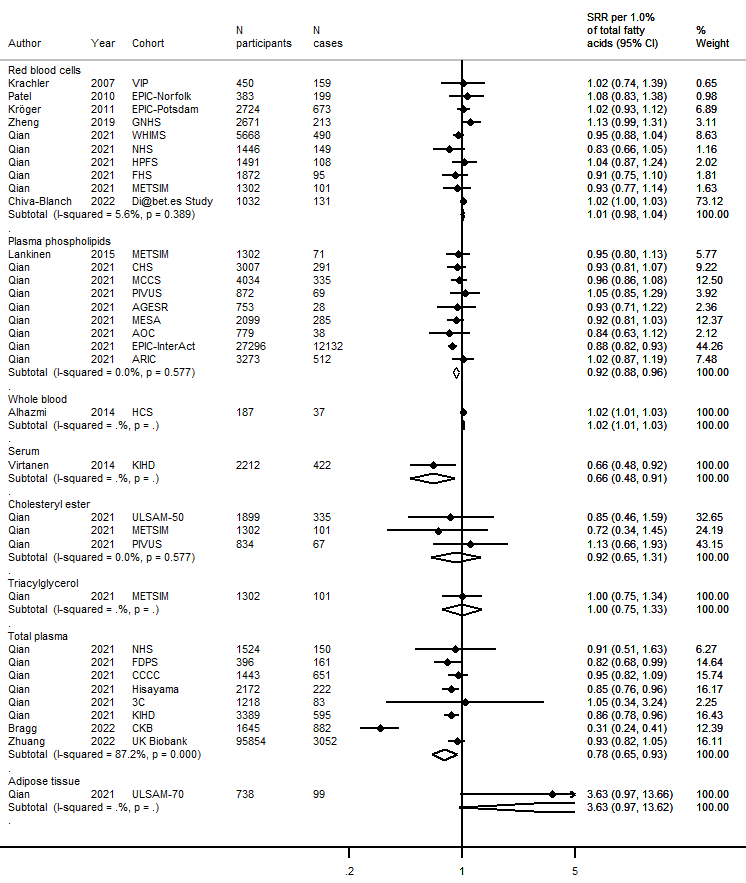


33) Eicosapentaenoic + Docosahexaenoic acid (20:5 + 22:6) in all biospecimens

34a) Eicosapentaenoic + Docosapentaenoic + Docosahexaenoic acid (20:5 + 22:5 + 22:6) in all biospecimens

34b) Eicosapentaenoic + Docosapentaenoic + Docosahexaenoic acid (20:5 + 22:5 + 22:6) across different biospecimens


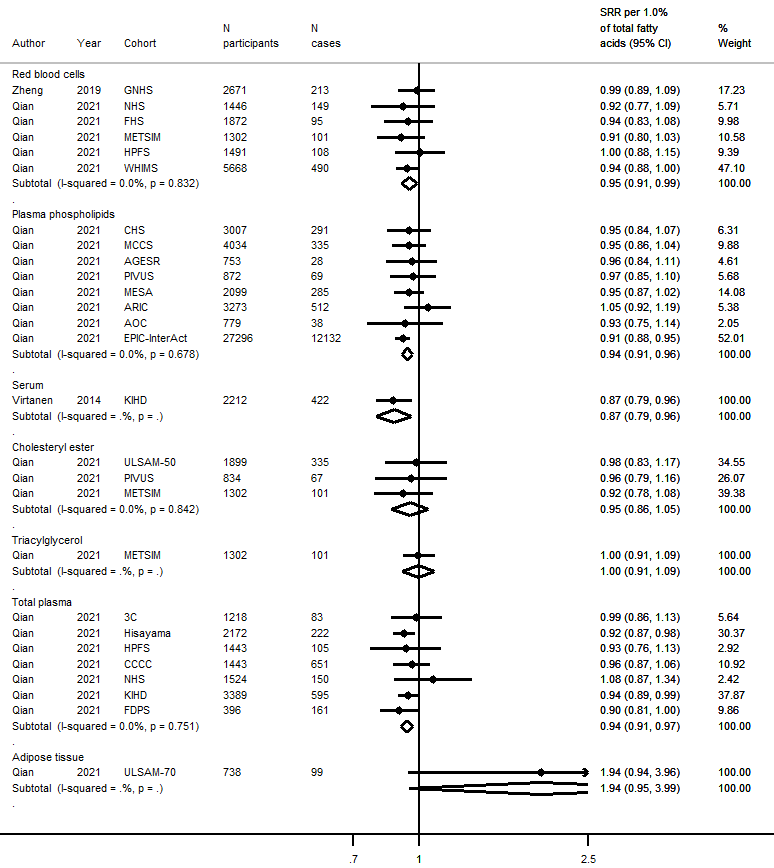


3C Study, Three-City Study; AGESR, Age, Gene/Environment Susceptibility - Reykjavik Study; AOC, Alpha Omega Cohort; ARIC, Atherosclerosis Risk in Communities Study; CCCC, Chin-Shan Community Cardiovascular Cohort; CHS, Cardiovascular Health Study; CI, confidence interval; CKB, China Kadoorie Biobank; Di@betes Study, Spanish nationwide cohort on diabetes; EPIC, European Prospective Investigation into Cancer and Nutrition; FDPS, Finnish Diabetes Prevention Study; FHS, Framingham Heart Study; GNHS, Guangzhou Nutrition and Health Study; HCS, Hunter Community Study; HPFS, Health Professionals Follow-up Study; IRAS, Insulin Resistance Atherosclerosis Study; KIHD, Kuopio Ischaemic Heart Disease Risk Factor Study; MCCS, Melbourne Collaborative Cohort Study; MESA, Multi-Ethnic Study of Atherosclerosis; METSIM, Metabolic Syndrome in Men Study; NHS, Nurses’ Health Study; PHS, Physicians’ Health Study; PIVUS, Prospective Investigation of the Vasculature in Uppsala Seniors; SRR, summary relative risk; ULSAM, Uppsala Longitudinal Study of Adult Men; VIP, Västerbotten Intervention Program survey; WHIMS, Women’s Health Initiative Memory Study

**Supplemental Figure 3.1-3.68**. Stepwise omission of one study at a time from meta-analyses of prospective cohort studies on association between specific fatty acids (% of total fatty acids) and risk of type 2 diabetes:

1) Total saturated fatty acids (SFAs) in red blood cells
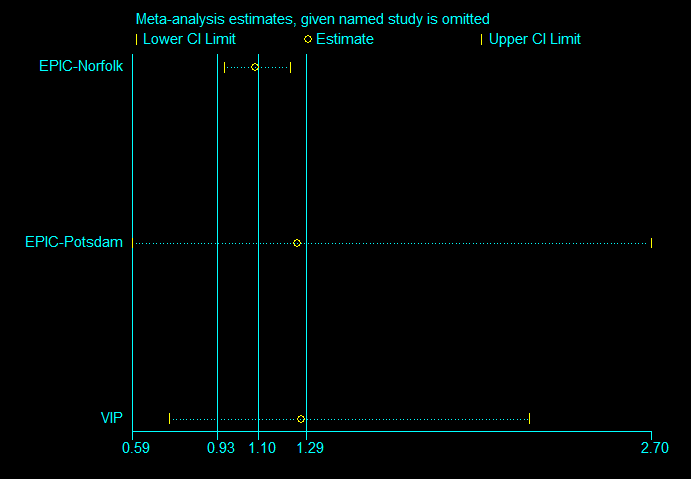


2) Myristic acid (14:0) in red blood cells


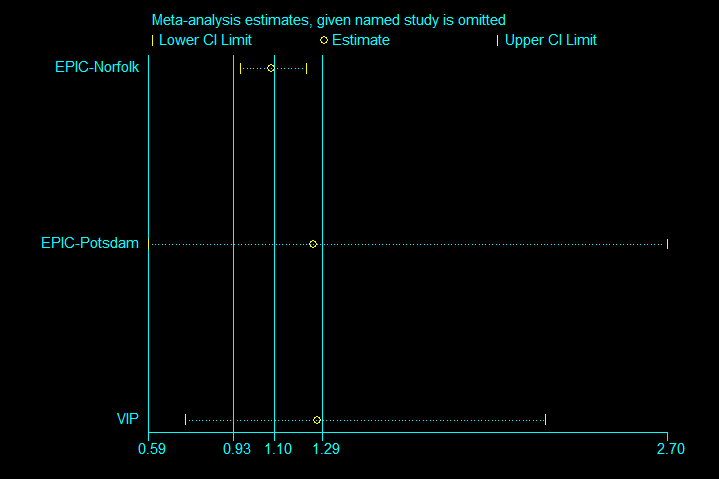


3) Myristic acid (14:0) in red blood cells
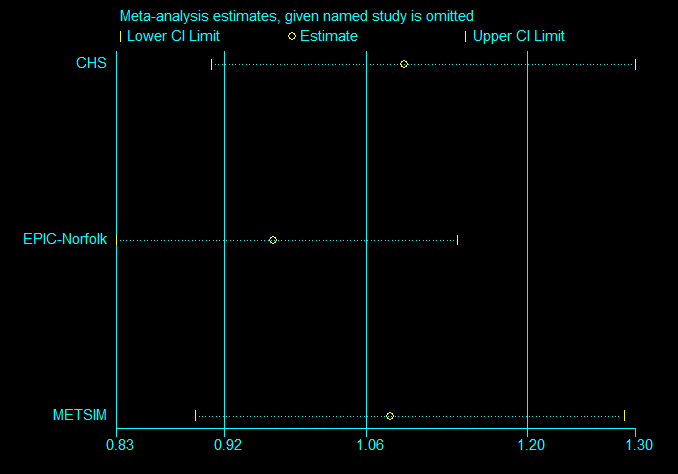


4) Pentadecanoic acid (15:0) in red blood cells


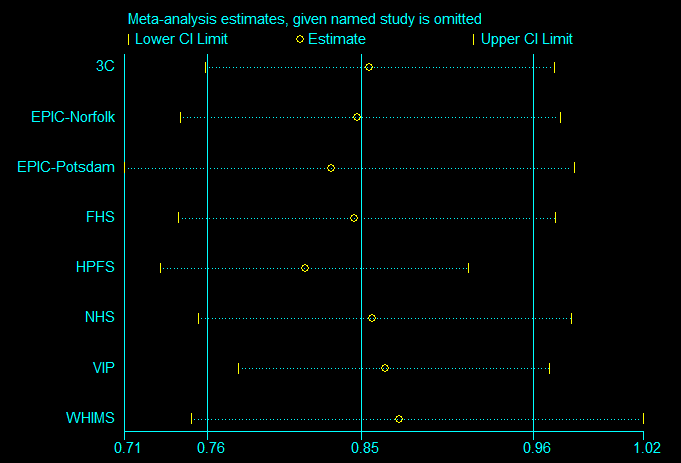


5) Pentadecanoic acid (15:0) in plasma phospholipids
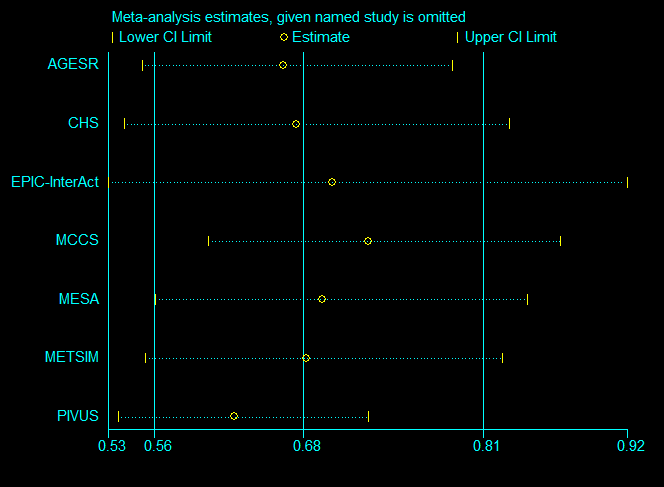


6) Pentadecanoic acid (15:0) in total plasma
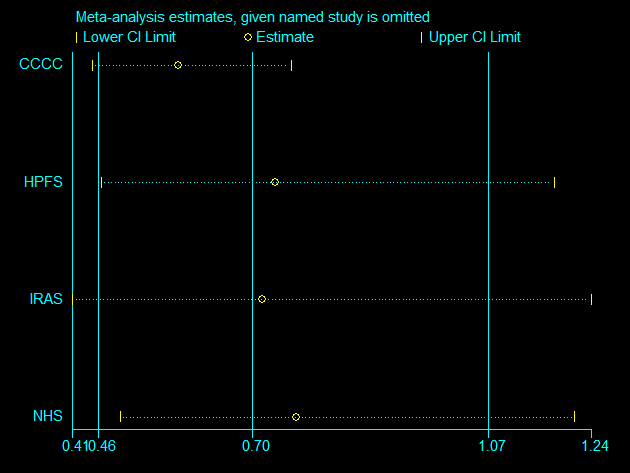


7) Palmitic acid (16:0) in red blood cells
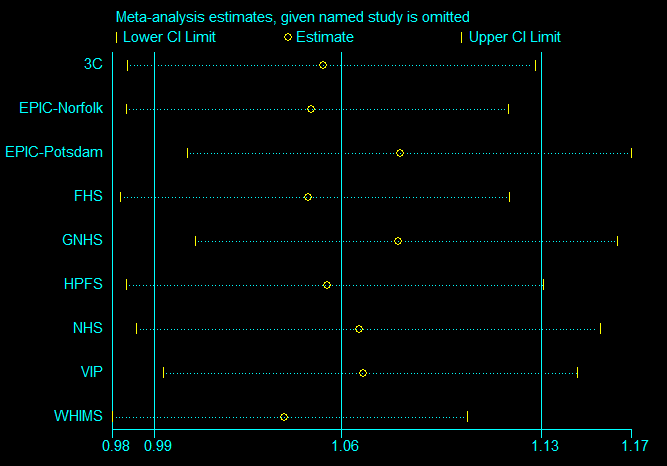


8) Palmitic acid (16:0) in plasma phospholipids
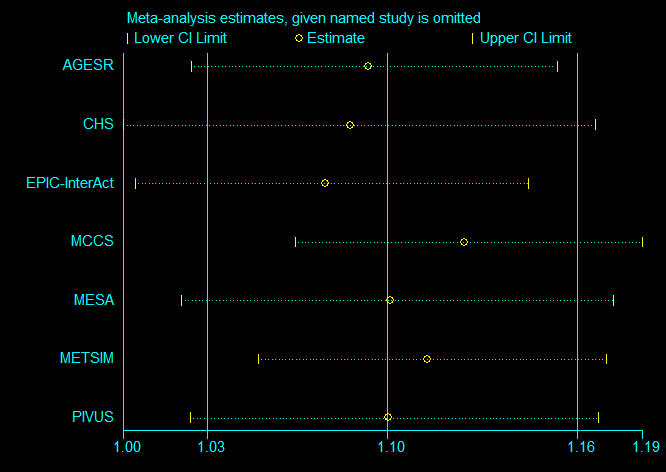


9) Palmitic acid (16:0) in total plasma
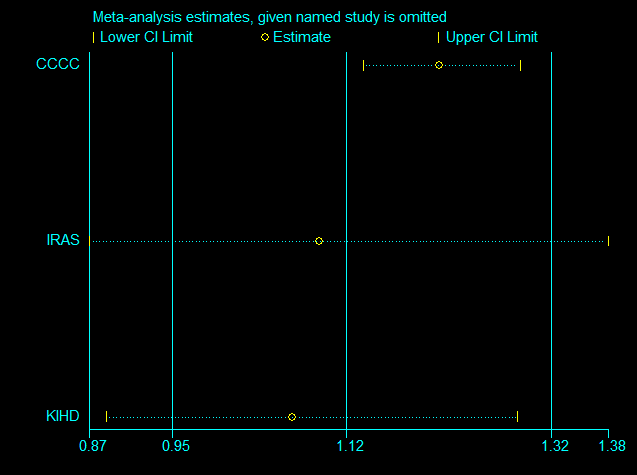


10) Margaric acid (17:0) in red blood cells
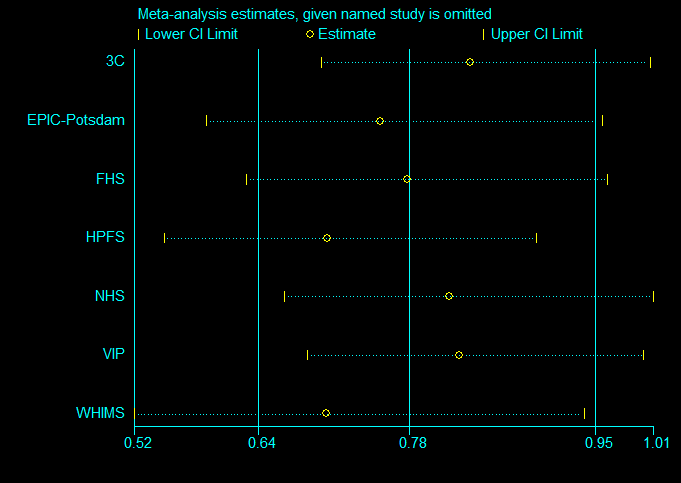


11) Margaric acid (17:0) in plasma phospholipids
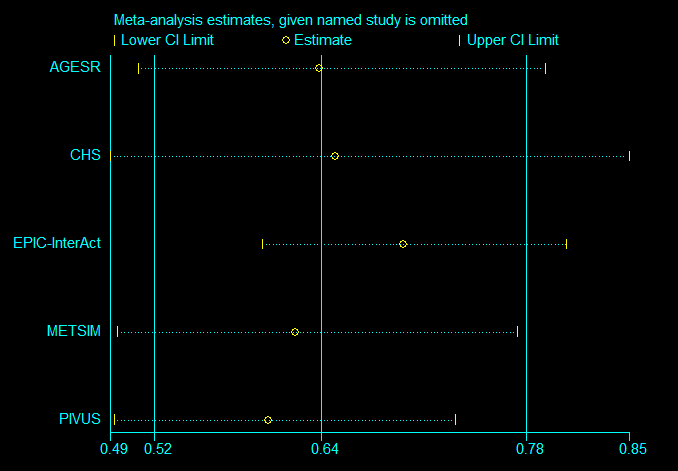


12) Margaric acid (17:0) in total plasma
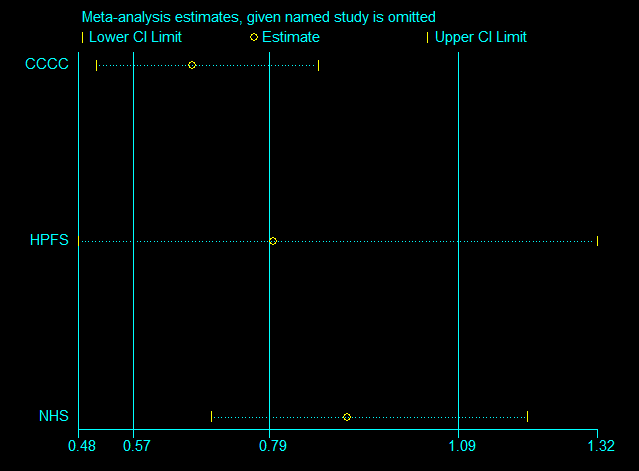


13) Stearic acid (18:0) in red blood cells
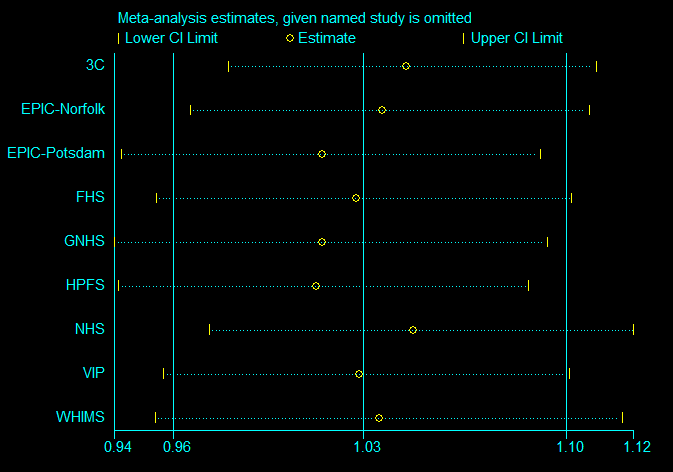


14) Stearic acid (18:0) in plasma phospholipids
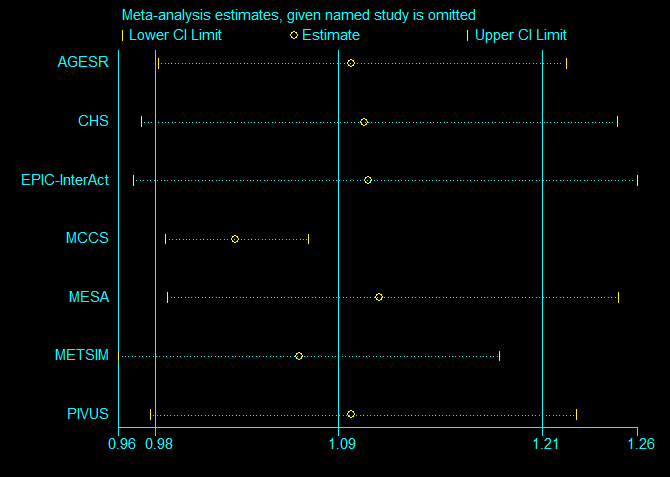


15) Stearic acid (18:0) in total plasma
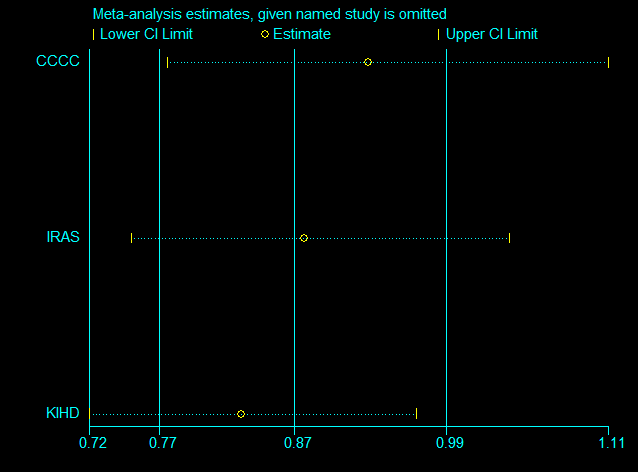


16) Arachidic acid (20:0) in red blood cells
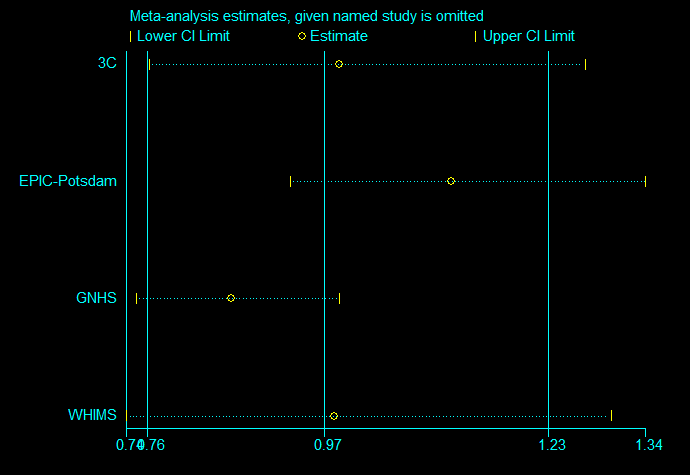


17) Arachidic acid (20:0) in plasma phospholipids
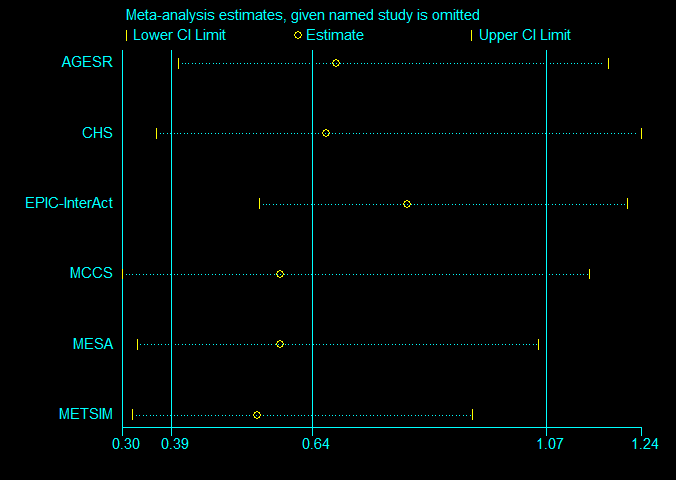


18) Behenic acid (22:0) in red blood cells
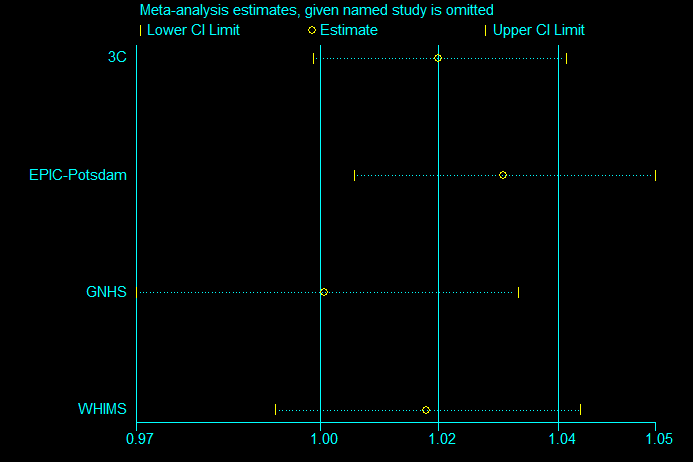


19) Behenic acid (22:0) in plasma phospholipids
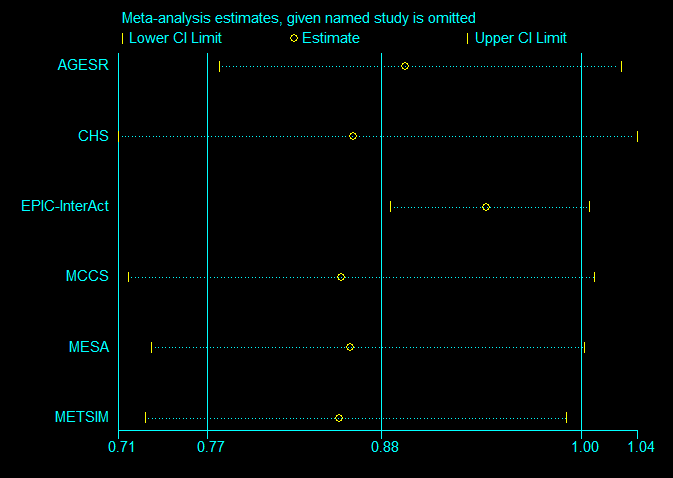


20) Behenic acid (22:0) in total plasma
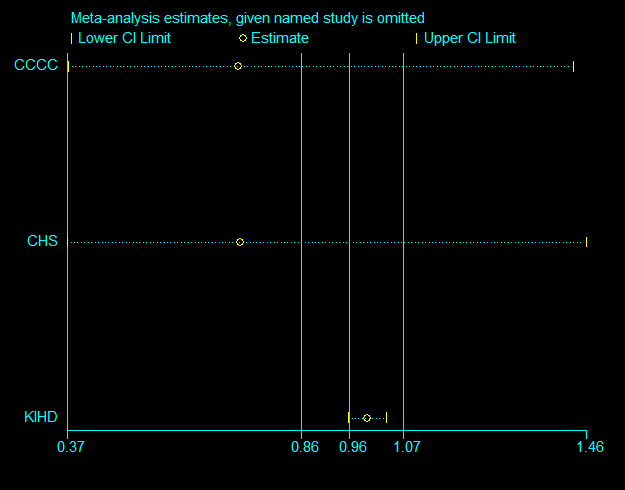


21) Lignoceric acid (24:0) in red blood cells
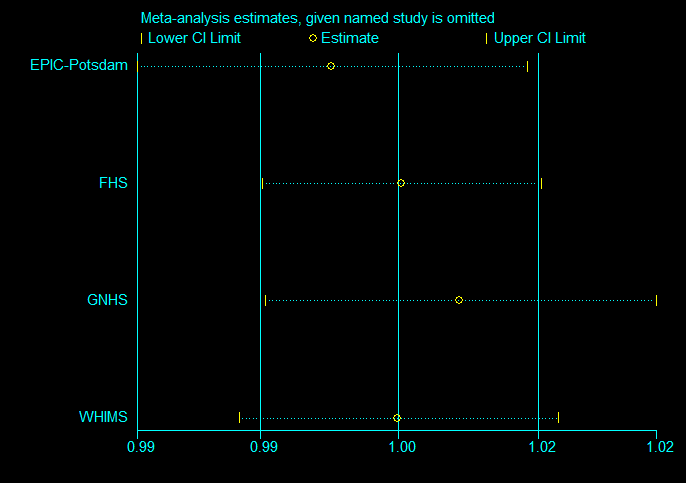


22) Lignoceric acid (24:0) in plasma phospholipids
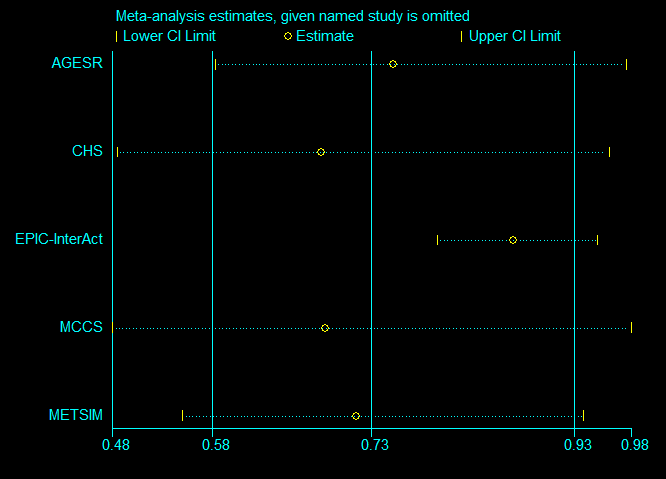


23) Lignoceric acid (24:0) in total plasma
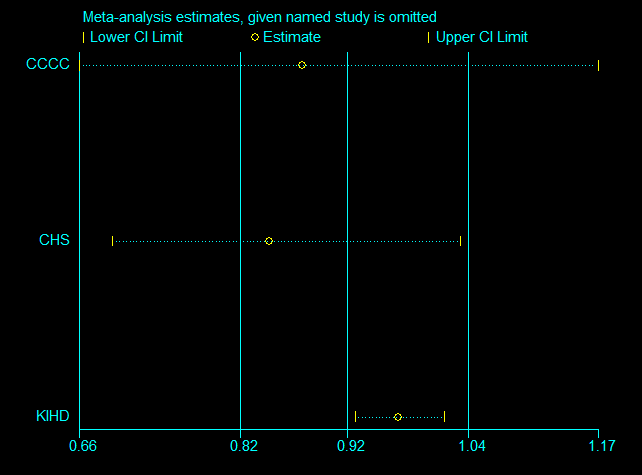


24) Total monounsaturated fatty acids (MUFAs) in red blood cells
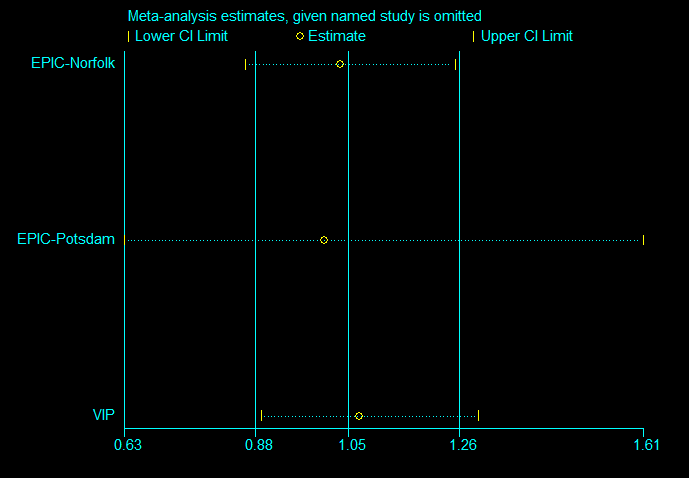


25) Palmitoleic acid (16:1n-7) in red blood cells
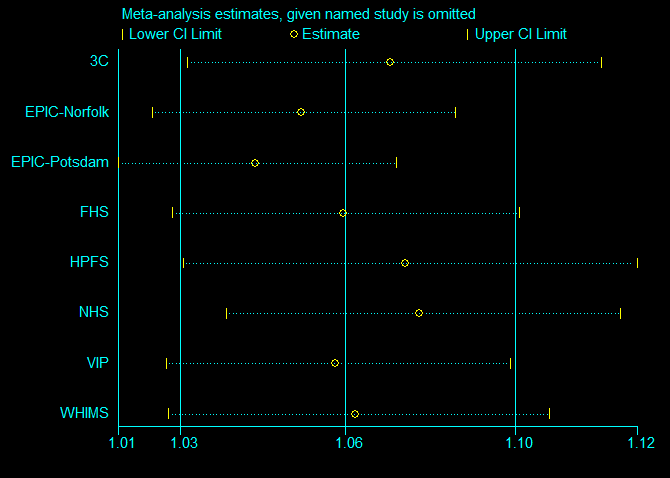


26) Palmitoleic acid (16:1n-7) in plasma phospholipids
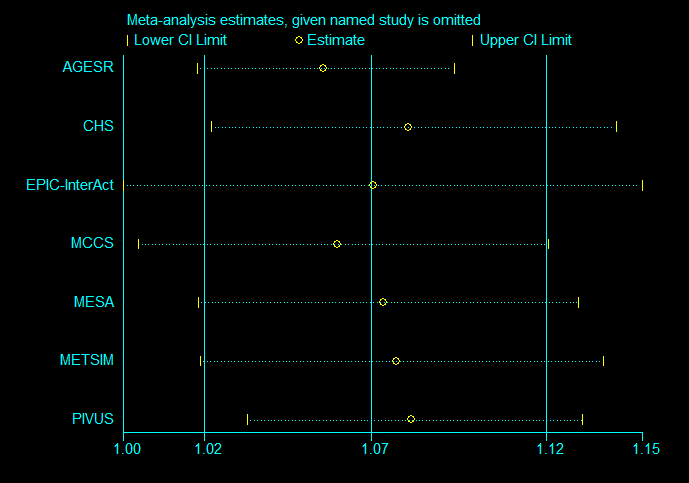


27) Oleic acid (18:1n-9) in red blood cells
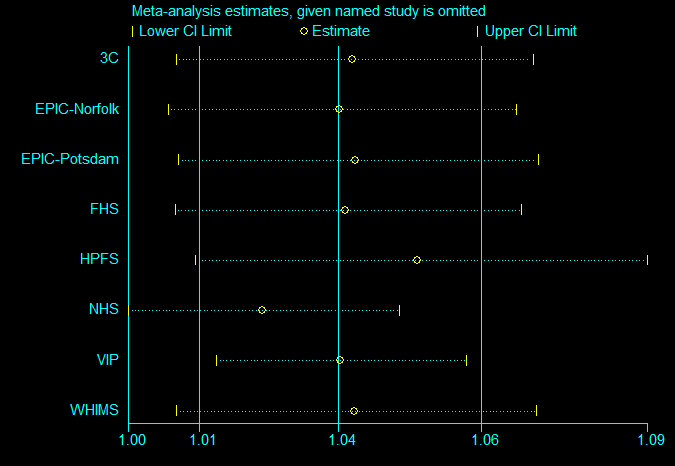


28) Oleic acid (18:1n-9) in plasma phospholipids
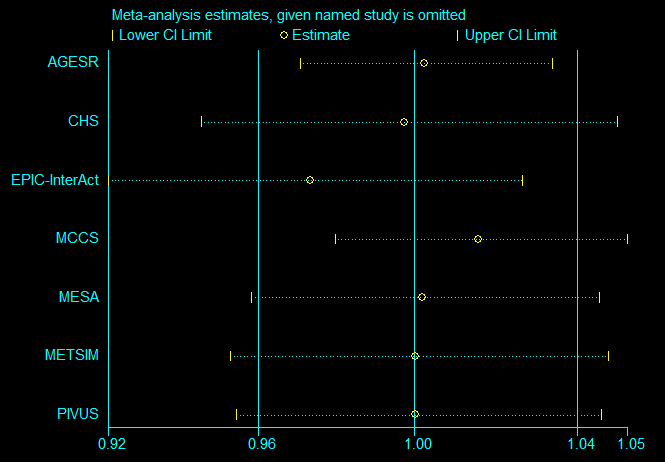


29) *Trans-*Vaccenic acid (*t-*18:1n-7) in plasma phospholipids
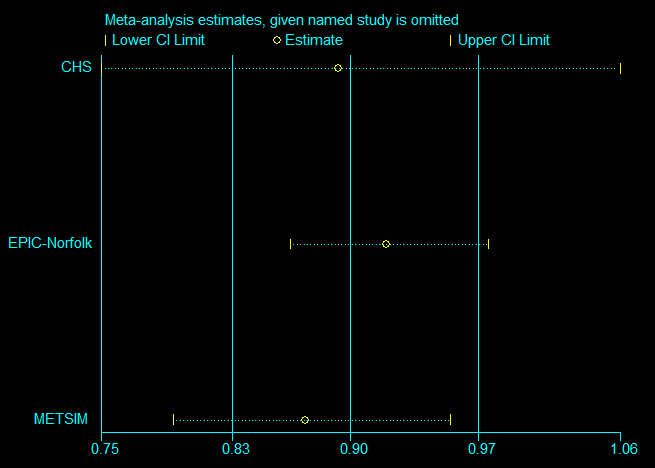


30) *Trans*-Elaidic acid (*t*-18:1n–9) in red blood cells


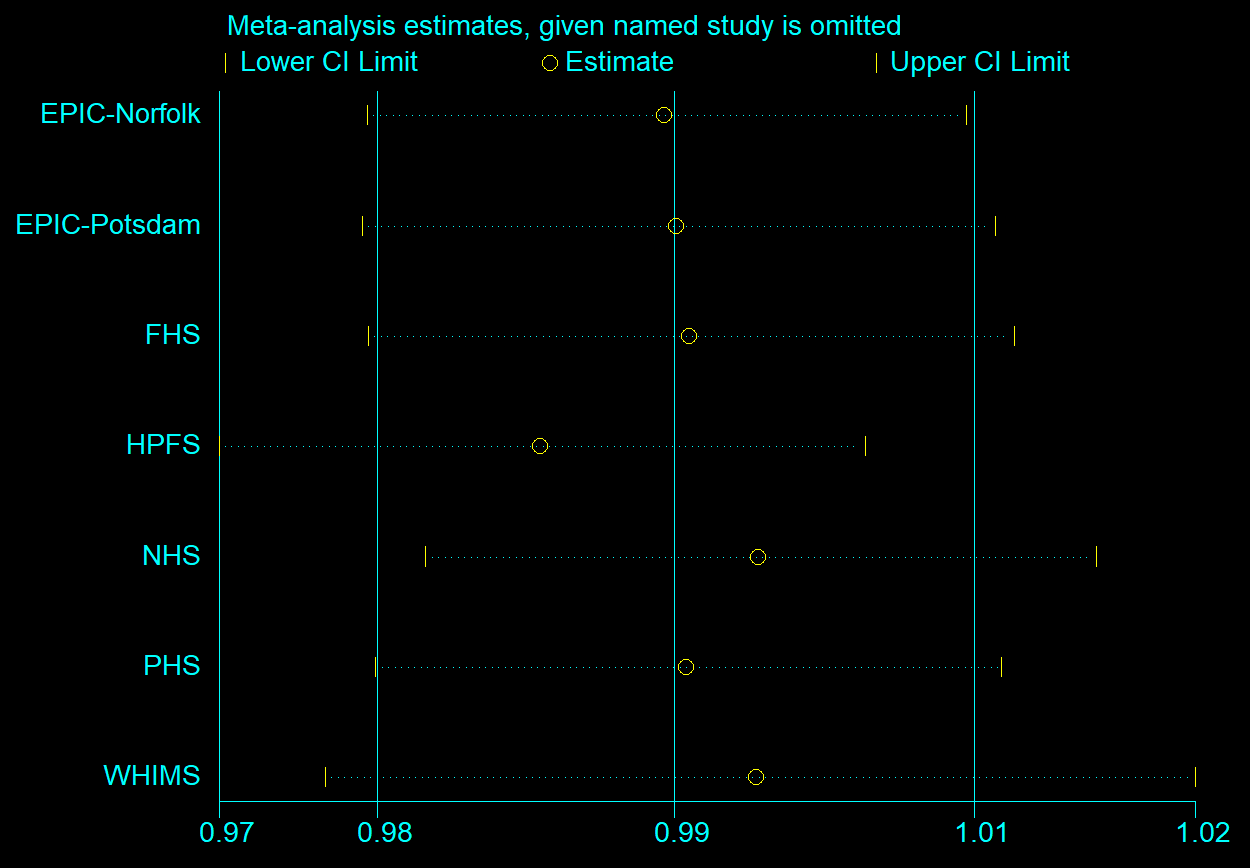


31) *Trans*-Elaidic acid (*t*-18:1n–9) in plasma phospholipids
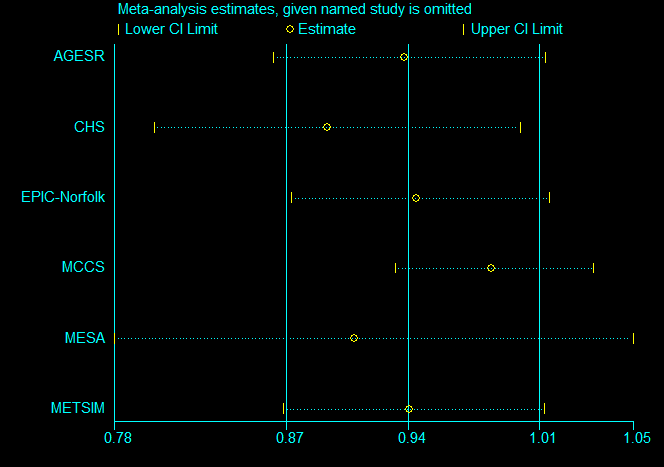


32) *Trans*-Hypogeic acid (*t*-16:1n-9) in plasma phospholipids
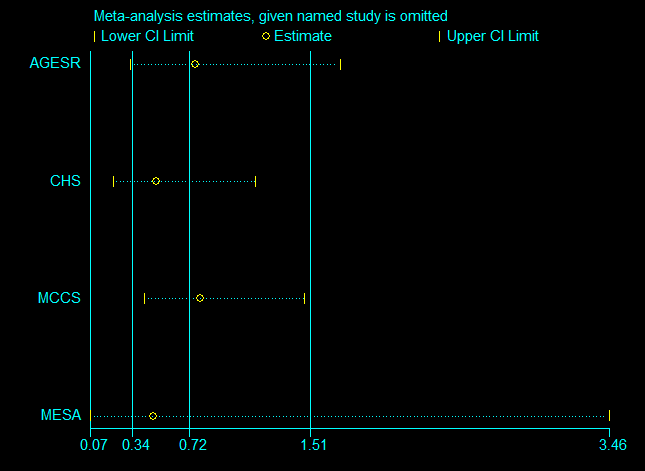


33) *Trans*-Palmitoleic acid (*t*-16:1n–7) in red blood cells
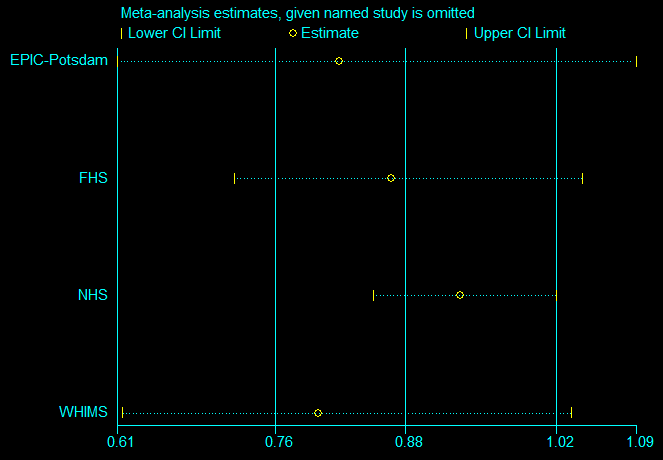


34) *Trans*-Palmitoleic acid (*t*-16:1n–7) in plasma phospholipids
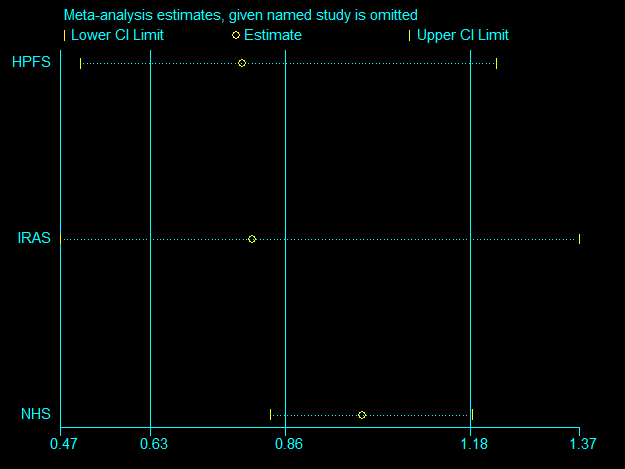


35) *Trans*-Palmitoleic acid (*t*-16:1n–7) in total plasma
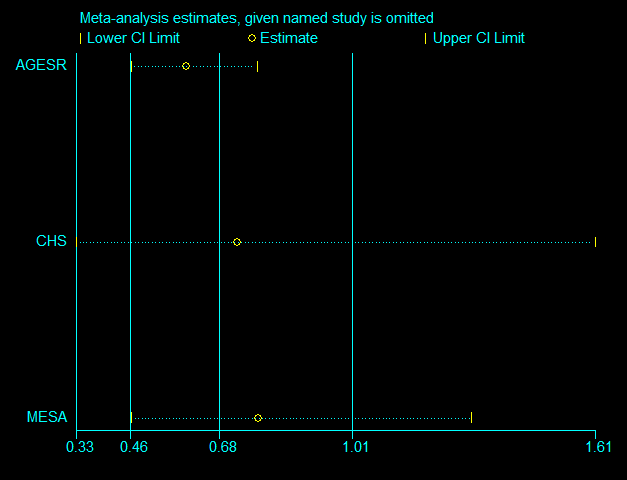


36) *Trans*-Linoleic acid (*t-*18:2) in red blood cells
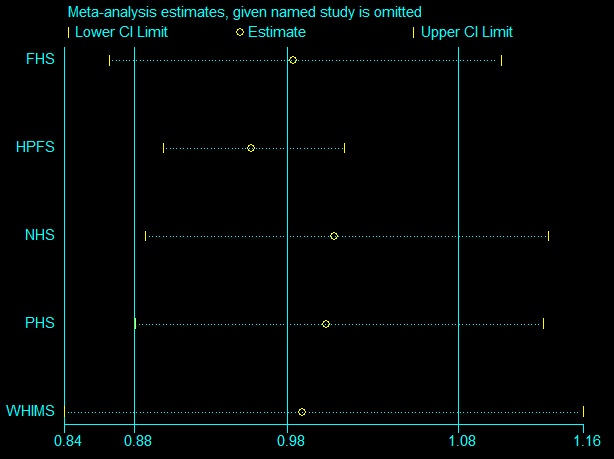


37) *Trans*-Linoleic acid (*t-*18:2) in plasma phospholipids
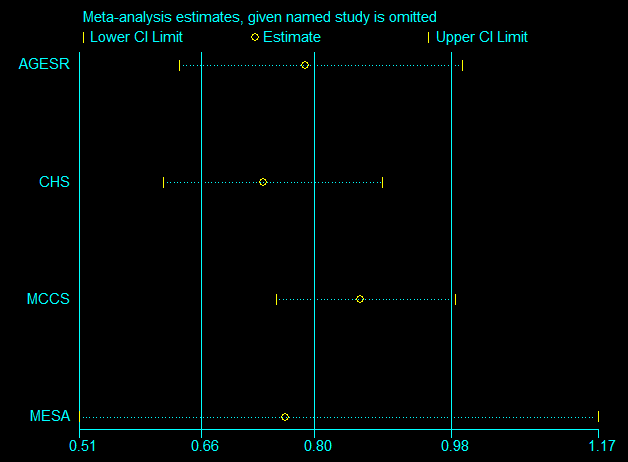


38) Total polyunsaturated fatty acids (PUFAs) in red blood cells
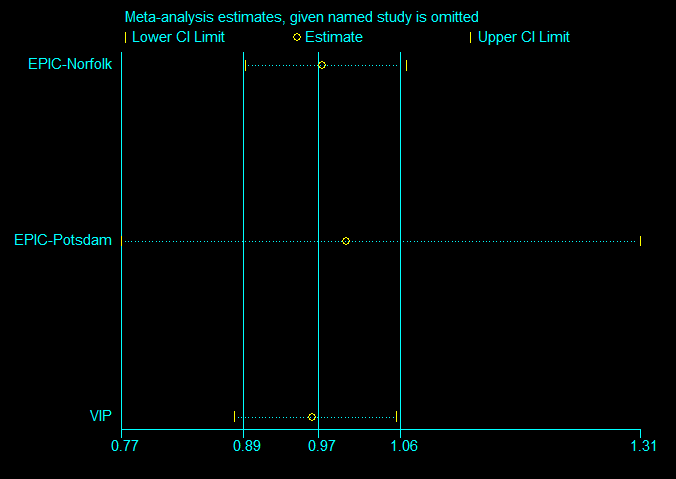


39) Total n-6 polyunsaturated fatty acids (PUFAs) in red blood cells
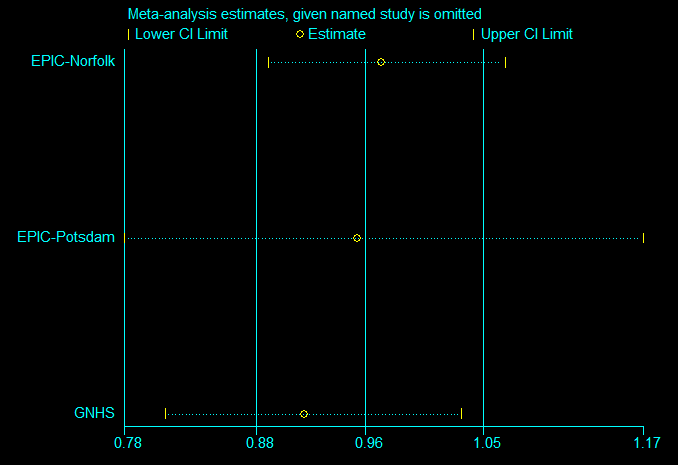


40) Linoleic acid (18:2) in red blood cells
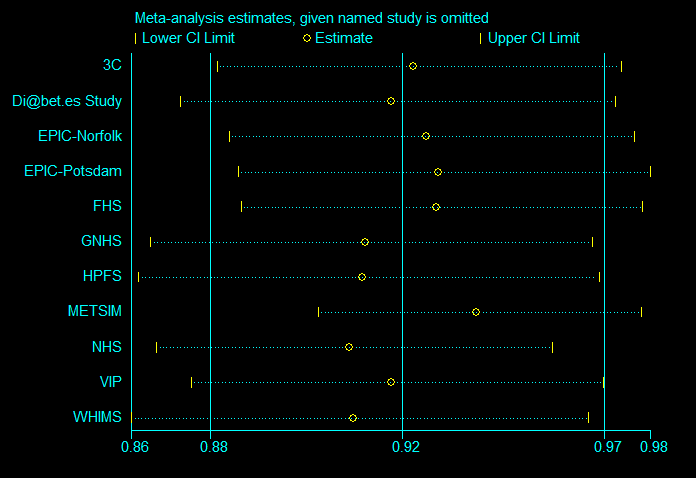


41) Linoleic acid (18:2) in plasma phospholipids
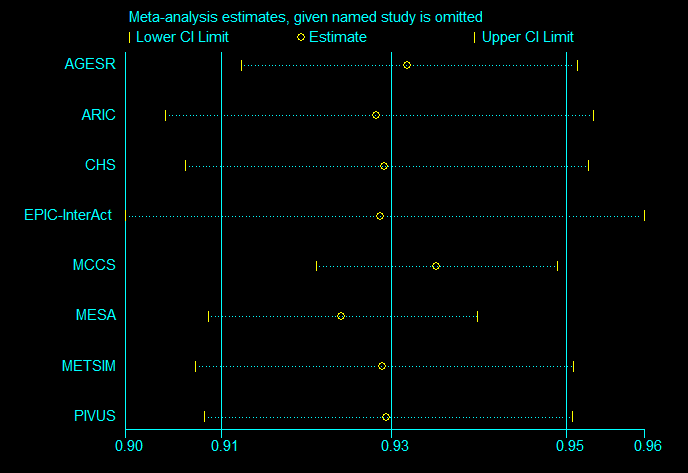


42) Linoleic acid (18:2) in total plasma
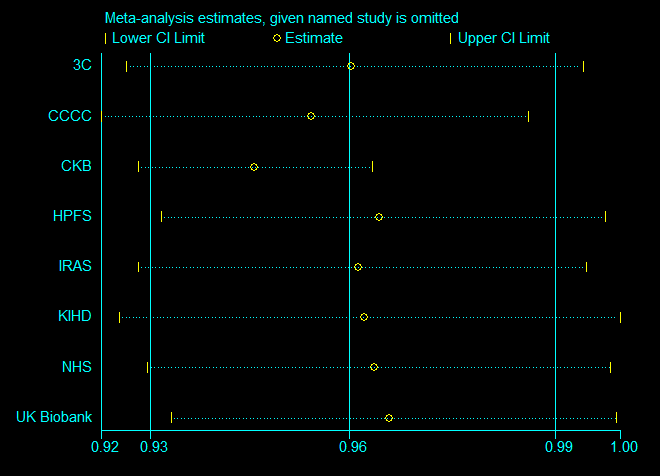


43) Linoleic acid (18:2) in cholesteryl ester
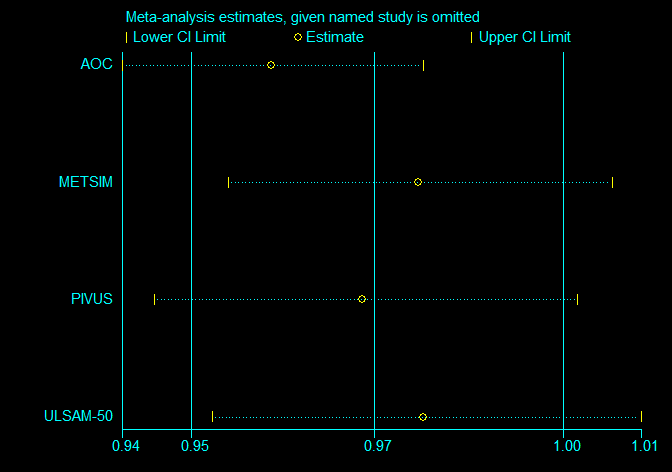


44) γ-Linolenic acid (γ-18:3) in red blood cells
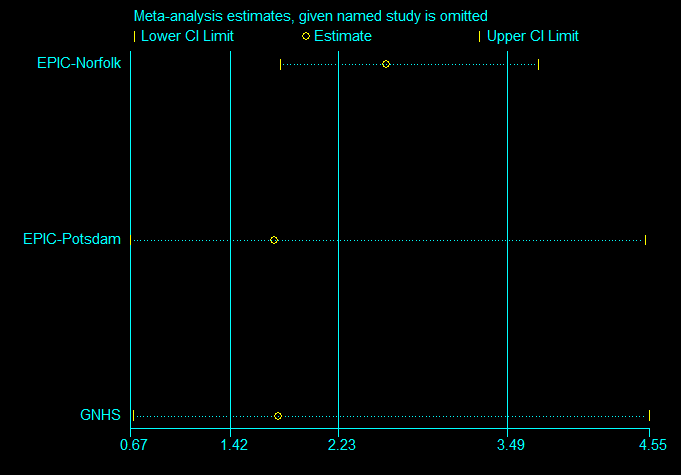


45) Arachidonic acid (20:4) in red blood cells
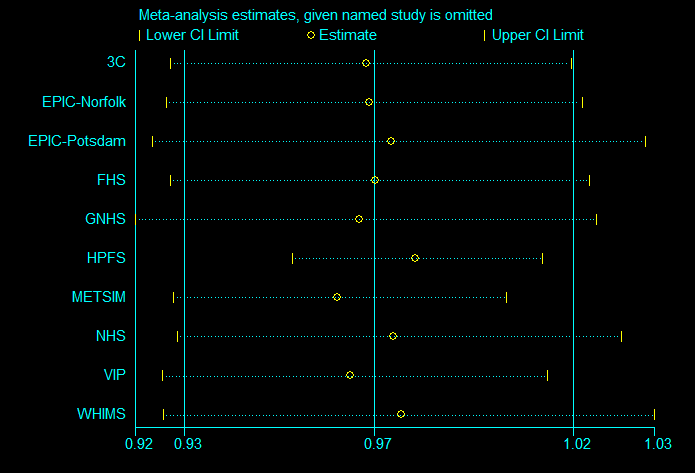


46) Arachidonic acid (20:4) in plasma phospholipids
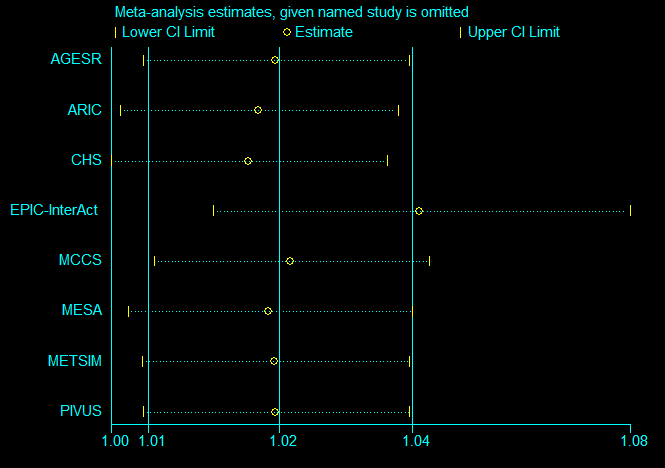


47) Arachidonic acid (20:4) in total plasma
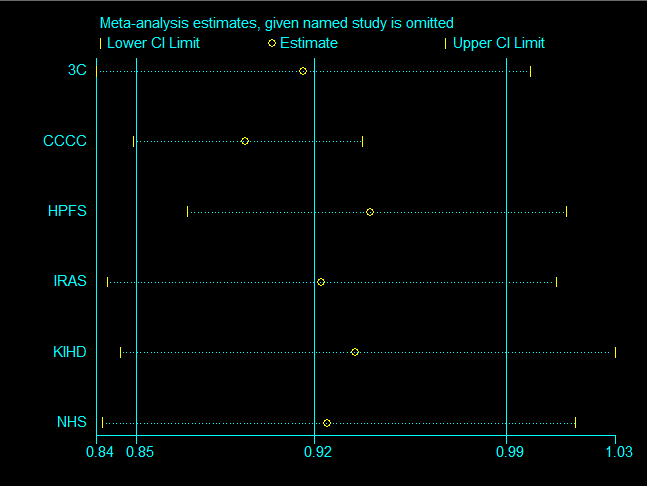


48) Arachidonic acid (20:4) in cholesteryl ester
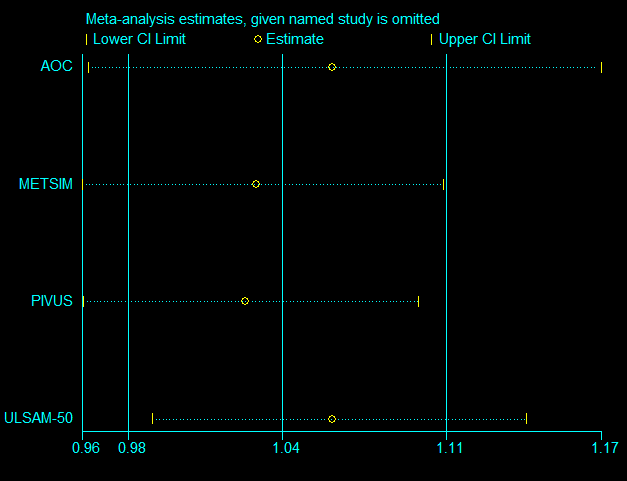


49) Docosatetraenoic acid (22:4) in red blood cells
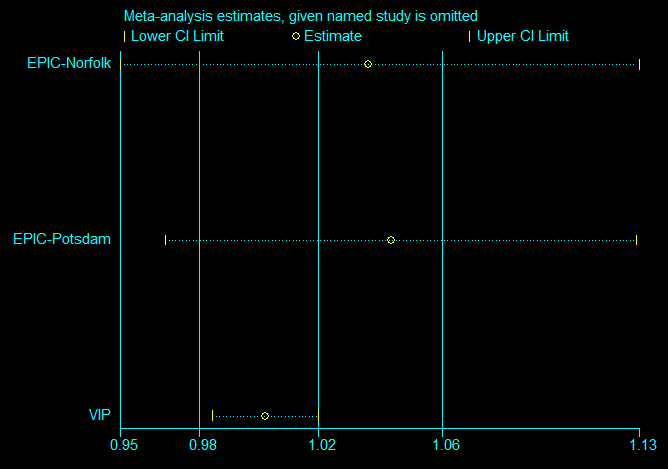


50) α-Linolenic acid (18:3) in red blood cells
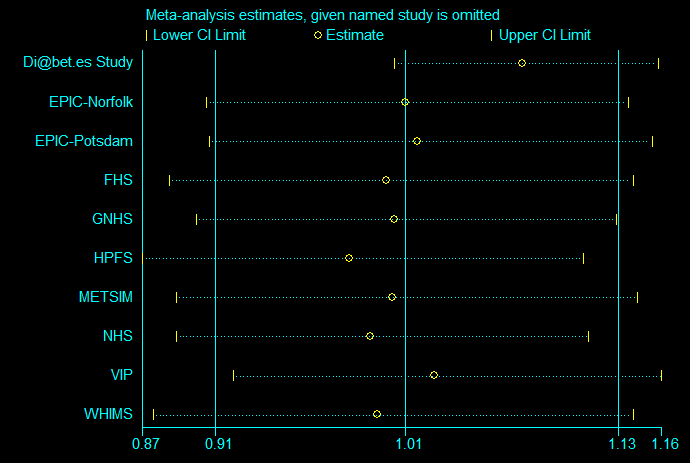


51) α-Llinolenic acid (18:3) in plasma phospholipids
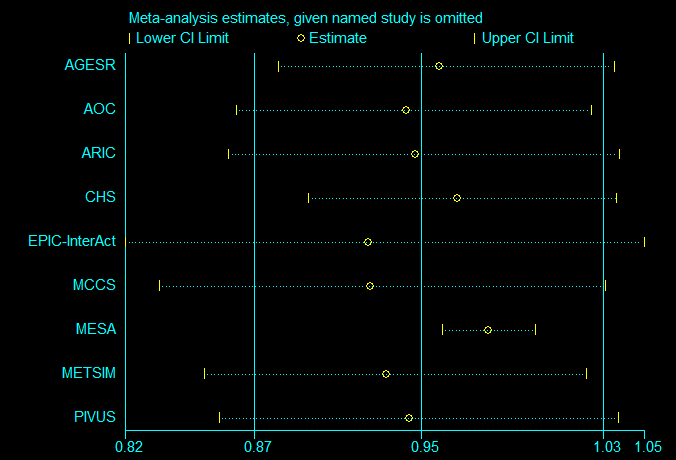


52) α-Linolenic acid (18:3) in total plasma
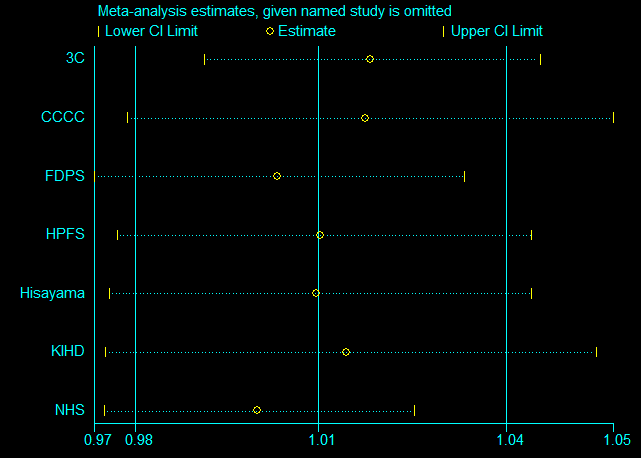


53) α-Linolenic acid (18:3) in cholesteryl ester
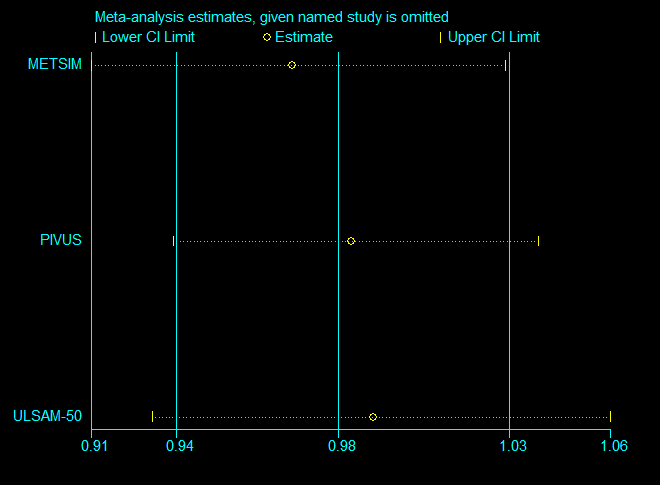


54) Eicosapentaenoic acid (20:5) in red blood cells
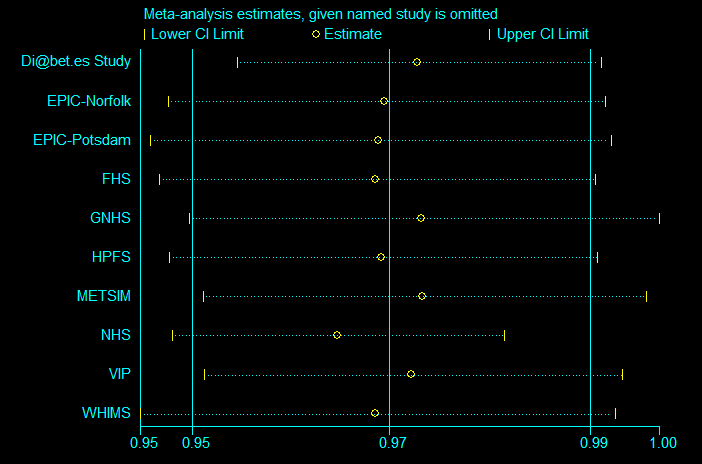


55) Eicosapentaenoic acid (20:5) in plasma phospholipids
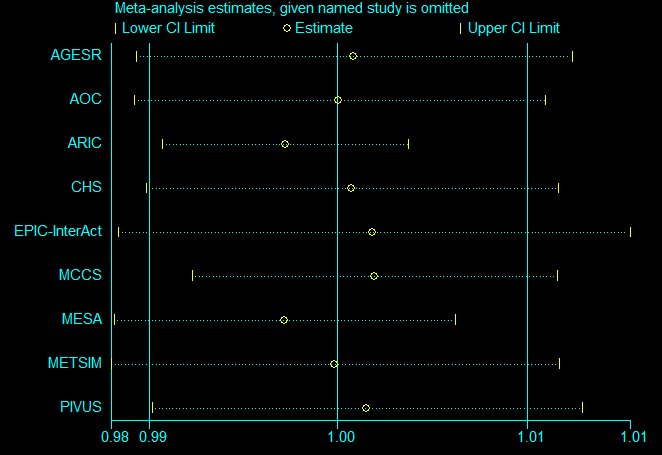


56) Eicosapentaenoic acid (20:5) in total plasma
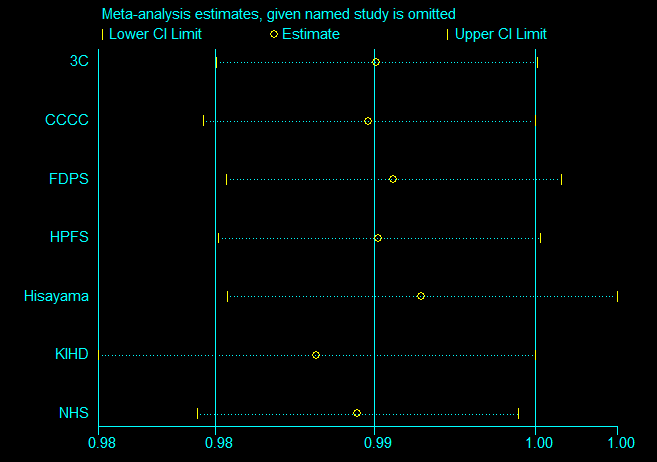


57) Eicosapentaenoic acid (20:5) in cholesteryl ester
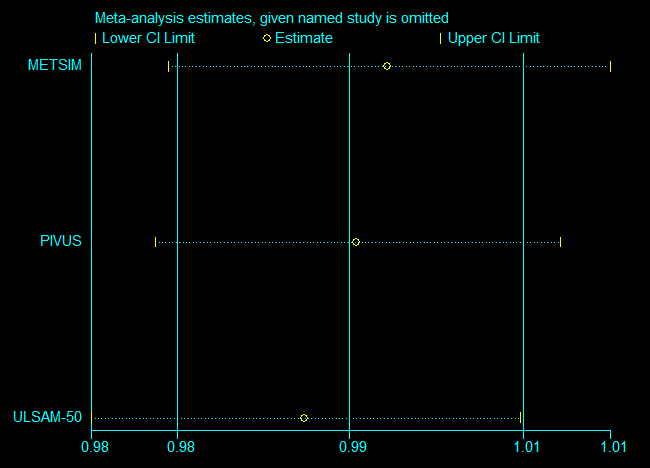


58) Docosapentaenoic acid (22:5) in red blood cells
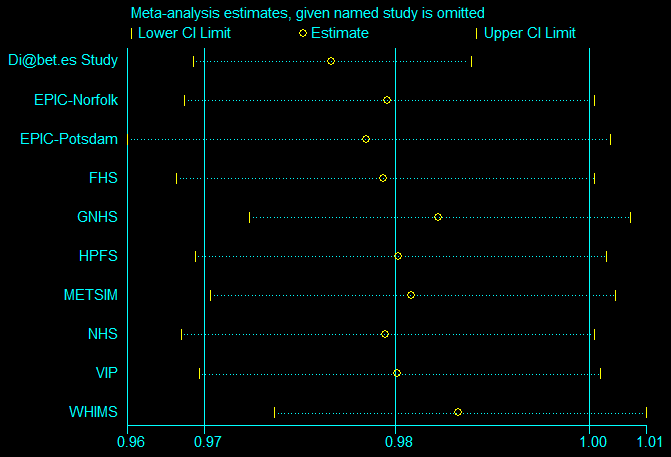


59) Docosapentaenoic acid (22:5) in plasma phospholipids
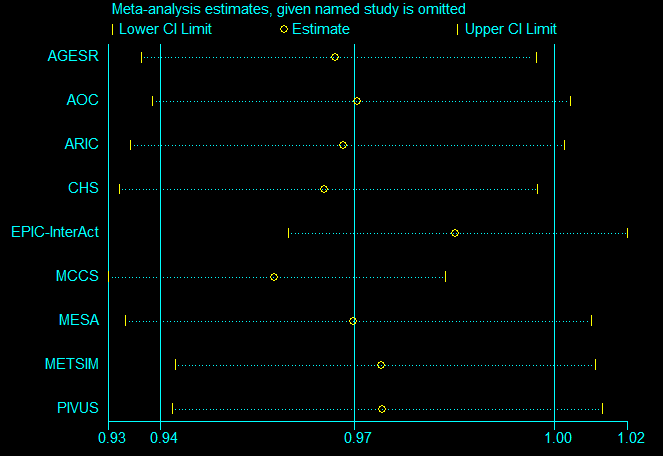


60) Docosapentaenoic acid (22:5) in total plasma
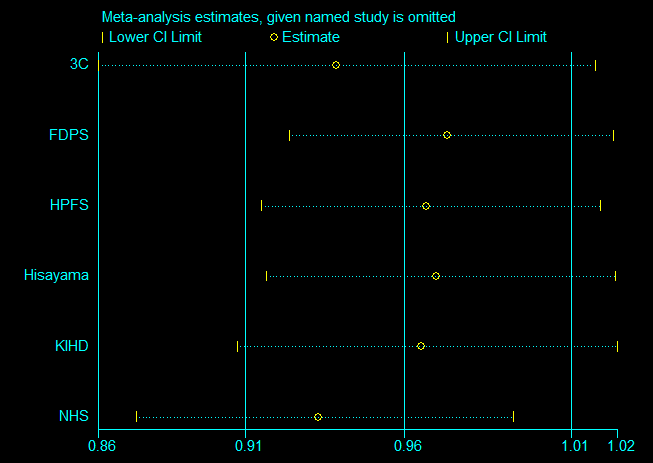


61) Docosahexaenoic acid (22:6) in red blood cells
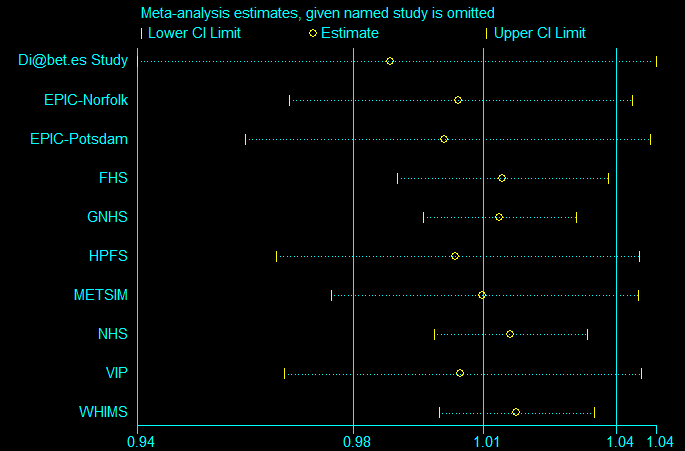


62) Docosahexaenoic acid (22:6) in plasma phospholipids
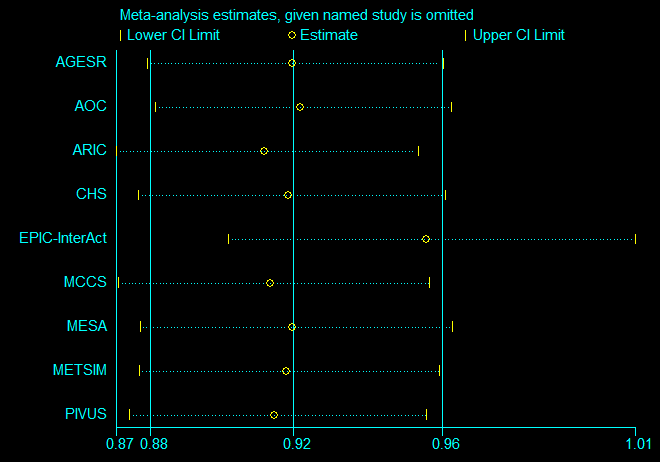


63) Docosahexaenoic acid (22:6) in total plasma

64) Docosahexaenoic acid (22:6) in cholesteryl ester

65) Eicosapentaenoic + Docosapentaenoic + Docosahexaenoic acid (20:5 + 22:5 + 22:6) in red blood cells

66) Eicosapentaenoic + Docosapentaenoic + Docosahexaenoic acid (20:5 + 22:5 + 22:6) in plasma phospholipids

67) Eicosapentaenoic + Docosapentaenoic + Docosahexaenoic acid (20:5 + 22:5 + 22:6) in total plasma

68) Eicosapentaenoic + Docosapentaenoic + Docosahexaenoic acid (20:5 + 22:5 + 22:6) in cholesteryl ester

3C Study, Three-City Study; AGESR, Age, Gene/Environment Susceptibility - Reykjavik Study; AOC, Alpha Omega Cohort; ARIC, Atherosclerosis Risk in Communities Study; CCCC, Chin-Shan Community Cardiovascular Cohort; CHS, Cardiovascular Health Study; CI, confidence interval; CKB, China Kadoorie Biobank; Di@betes Study, Spanish nationwide cohort on diabetes; EPIC, European Prospective Investigation into Cancer and Nutrition; FDPS, Finnish Diabetes Prevention Study; FHS, Framingham Heart Study; GNHS, Guangzhou Nutrition and Health Study; HCS, Hunter Community Study; HPFS, Health Professionals Follow-up Study; IRAS, Insulin Resistance Atherosclerosis Study; KIHD, Kuopio Ischaemic Heart Disease Risk Factor Study; MCCS, Melbourne Collaborative Cohort Study; MESA, Multi-Ethnic Study of Atherosclerosis; METSIM, Metabolic Syndrome in Men Study; NHS, Nurses’ Health Study; PHS, Physicians’ Health Study; PIVUS, Prospective Investigation of the Vasculature in Uppsala Seniors; ULSAM, Uppsala Longitudinal Study of Adult Men; VIP, Västerbotten Intervention Program survey; WHIMS, Women’s Health Initiative Memory Study

**Supplemental Figure 4.1-4.24**. Funnel plots from meta-analyses of prospective cohort studies on association between specific fatty acids (% of total fatty acids) and risk of type 2 diabetes:

1. Pentadecanoic acid (15:0) in all biospecimens

1. Palmitic acid (16:0) in all biospecimens
2. Margaric acid (17:0) in all biospecimens

1. Stearic acid (18:0) in all biospecimens

1. Arachidic acid (20:0) in all biospecimens

1. Behenic acid (22:0) in all biospecimens

1. Lignoceric acid (24:0) in all biospecimens

1. Palmitoleic acid (16:1n-7) in all biospecimens

1. Oleic acid (18:1n-9) in all biospecimens

1. *Trans*-Elaidic acid (*t-*18:1n–9) in all biospecimens

1. *Trans*-Linoleic acid (*t-*18:2)

1. Linoleic acid (18:2) in all biospecimens

1. Linoleic acid (18:2) in red blood cells
2. Arachidonic acid (20:4) in all biospecimens

1. Arachidonic acid (20:4) in red blood cells

1. α-Linolenic acid (α-18:3) in all biospecimens

1. α-Linolenic acid (α-18:3) in red blood cells
2. Eicosapentaenoic acid (20:5) in all biospecimens

1. Eicosapentaenoic acid (20:5) in red blood cells
2. Docosapentaenoic acid (22:5) in all biospecimens

1. Docosapentaenoic acid (22:5) in red blood cells
2. Docosahexaenoic acid (22:6) in all biospecimens

1. Docosahexaenoic acid (22:6) in red blood cells
2. Eicosapentaenoic + Docosapentaenoic + Docosahexaenoic acid (20:5 + 22:5 + 22:6) in all biospecimens

RR, relative risk

**References**

1. Bragg F, Kartsonaki C, Guo Y, Holmes M, Du H, Yu C, et al. The role of NMR-based circulating metabolic biomarkers in development and risk prediction of new onset type 2 diabetes. Sci Rep. 2022;12(1):15071.

2. Forouhi NG, Imamura F, Sharp SJ, Koulman A, Schulze MB, Zheng J, et al. Association of Plasma Phospholipid n-3 and n-6 Polyunsaturated Fatty Acids with Type 2 Diabetes: The EPIC-InterAct Case-Cohort Study. PLoS Med. 2016;13(7):e1002094.

3. Patel PS, Sharp SJ, Jansen E, Luben RN, Khaw KT, Wareham NJ, Forouhi NG. Fatty acids measured in plasma and erythrocyte-membrane phospholipids and derived by food-frequency questionnaire and the risk of new-onset type 2 diabetes: a pilot study in the European Prospective Investigation into Cancer and Nutrition (EPIC)-Norfolk cohort. Am J Clin Nutr. 2010;92(5):1214-22.

4. Takkunen MJ, Schwab US, de Mello VD, Eriksson JG, Lindstrom J, Tuomilehto J, Uusitupa MI. Longitudinal associations of serum fatty acid composition with type 2 diabetes risk and markers of insulin secretion and sensitivity in the Finnish Diabetes Prevention Study. Eur J Nutr. 2016;55(3):967-79.

5. Santaren ID, Watkins SM, Liese AD, Wagenknecht LE, Rewers MJ, Haffner SM, et al. Serum pentadecanoic acid (15:0), a short-term marker of dairy food intake, is inversely associated with incident type 2 diabetes and its underlying disorders. Am J Clin Nutr. 2014;100(6):1532-40.

6. Skeaff CM, Hodson L, McKenzie JE. Dietary-induced changes in fatty acid composition of human plasma, platelet, and erythrocyte lipids follow a similar time course. J Nutr. 2006;136(3):565-9.

7. Katan MB, Deslypere JP, van Birgelen AP, Penders M, Zegwaard M. Kinetics of the incorporation of dietary fatty acids into serum cholesteryl esters, erythrocyte membranes, and adipose tissue: an 18-month controlled study. J Lipid Res. 1997;38(10):2012-22.

8. Furtado JD, Beqari J, Campos H. Comparison of the Utility of Total Plasma Fatty Acids Versus those in Cholesteryl Ester, Phospholipid, and Triglyceride as Biomarkers of Fatty Acid Intake. Nutrients. 2019;11(9).

9. Brenna JT, Plourde M, Stark KD, Jones PJ, Lin YH. Best practices for the design, laboratory analysis, and reporting of trials involving fatty acids. Am J Clin Nutr. 2018;108(2):211-27.

10. Bell JG, Mackinlay EE, Dick JR, Younger I, Lands B, Gilhooly T. Using a fingertip whole blood sample for rapid fatty acid measurement: method validation and correlation with erythrocyte polar lipid compositions in UK subjects. Br J Nutr. 2011;106(9):1408-15.

11. Arab L. Biomarkers of Fat and Fatty Acid Intake. The Journal of Nutrition. 2003;133(3):925S-32S.

12. Feldman AL, Long GH, Johansson I, Weinehall L, Fhärm E, Wennberg P, et al. Change in lifestyle behaviors and diabetes risk: evidence from a population-based cohort study with 10 year follow-up. Int J Behav Nutr Phys Act. 2017;14(1):39.

13. VanWormer JJ, Boucher JL, Sidebottom AC, Sillah A, Knickelbine T. Lifestyle changes and prevention of metabolic syndrome in the Heart of New Ulm Project. Prev Med Rep. 2017;6:242-5.

14. Zheng JS, Imamura F, Sharp SJ, Koulman A, Griffin JL, Mulligan AA, et al. Changes in plasma phospholipid fatty acid profiles over 13 years and correlates of change: European Prospective Investigation into Cancer and Nutrition-Norfolk Study. Am J Clin Nutr. 2019;109(6):1527-34.

15. Schünemann HJ, Cuello C, Akl EA, Mustafa RA, Meerpohl JJ, Thayer K, et al. GRADE guidelines: 18. How ROBINS-I and other tools to assess risk of bias in nonrandomized studies should be used to rate the certainty of a body of evidence. Journal of clinical epidemiology. 2019;111:105-14.

16. Abhari SA, Luben RN, Powell N, Bhaniani A, Wareham NJ, Forouhi NG, Khaw KT. Dietary intake of carbohydrates and risk of type 2 diabetes: European Prospective Investigation into Cancer in Norfolk study. Diabetologia. 2013;56:S159-S60.

17. Adams LA, Waters OR, Knuiman M, Elliott R, Olynyk JK. NAFLD as a risk factor for the development of diabetes and the metabolic syndrome: An eleven year follow-up study. Hepatology. 2007;46(4):744A-A.

18. Hata A, Aki N, Ichihara T, Tamura A, Minagawa T, Kuwamura Y, Funaki M. Serum Fatty Acid Composition as a Biomarker of Diabetes Development: Tokushima Cohort Study. Diabetes. 2022;71.

19. Jo SH, Kim HS, Han SJ, Kim SA. Fenofibrate or omega-3 fatty acid use is associated with lower long term outcomes from propensity matched analysis of 141972 patients with diabetes in Korean national health insurance cohort. European Heart Journal. 2020;41:3336-.

20. Lai HTM, Imamura F, Korat AVA, Murphy R, Tintle NL, Bassett J, et al. TRANS FATTY ACID BIOMARKERS AND INCIDENT TYPE 2 DIABETES: POOLED ANALYSIS OF 10 PROSPECTIVE COHORT STUDIES IN THE FATTY ACIDS AND OUTCOMES RESEARCH CONSORTIUM (FORCE). Journal of Epidemiology and Community Health. 2019;73:A17-A.

21. Marklund M, Laguzzi F, Vikstrom M, Alsharari Z, Sjogren P, Gigante B, et al. Polyunsaturated Fat Intake Estimated by Circulating Biomarkers is Inversely Associated with Cardiovascular Disease and All-Cause Mortality in a Large Population-Based Cohort of Swedish Women and Men. Circulation. 2014;130.

22. Merino J, Guasch-Ferre M, Ellervik C, Dashti H, Smith CE, Kilpelainen TO, et al. Dietary fat quality and genetic risk of type 2 diabetes. Diabetologia. 2018;61:S118-S.

23. Miao ZL. Erythrocyte n-6 Polyunsaturated Fatty Acids, Gut Microbiota and Incident Type 2 Diabetes: A Prospective Cohort Study. Journal of the American Oil Chemists Society. 2021;98:230-.

24. Qian F, Korat AVA, Imamura F, Marklund M, Tintle N, Virtanen JK, et al. Omega-3 Fatty Acid Biomarkers and Incident Type 2 Diabetes: An Individual Participant-level Pooling Project of 20 Prospective Cohort Studies. Circulation. 2019;139.

25. Qian F, Zong G, Li YP, Sun Q. Monounsaturated Fatty Acids From Plant or Animal Sources and Risk of Type 2 Diabetes in Three Large Prospective Cohorts of Men and Women. Circulation. 2019;139.

26. Weir NL, Guan W, Steffen B, Steffen LM, Karger AB, Tsai MY. Omega-6 Eicosadienoic Acid Is Associated with Lower HOMA-IR and Incident T2D in Participants from the MESA Cohort. Diabetes. 2018;67.

27. Weir NL, Johnson L, Guan W, Steffen B, Djousse L, Mukamal KJ, Tsai MY. Cis-Vaccenic Acid Is Associated with Lower HOMA-IR and Incident T2D in Participants from the MESA Cohort. Diabetes. 2018;67.

28. Wu JH, Marklund M, Imamura F, Tintle N, Korat AVA, de Goede J, et al. Omega-6 Fatty Acid Biomarkers and Incident Type 2 Diabetes: A Pooled Analysis of 20 Cohort Studies. Circulation. 2017;135.

29. Zong G, Liu G, Wanders AJ, Alssema M, Zock PL, Willett W, et al. Dietary Linoleic Acid Intake Is Inversely Associated With Type 2 Diabetes Risk In Three Large Prospective Cohort Studies Of U.s. Men And Women. Circulation. 2018;137.

30. Bragg F, Trichia E, Aguilar-Ramirez D, Besevic J, Lewington S, Emberson J. Predictive value of circulating NMR metabolic biomarkers for type 2 diabetes risk in the UK Biobank study. Bmc Medicine. 2022;20(1).

31. Forouhi NG, Koulman A, Sharp SJ, Imamura F, Kroger J, Schulze MB, et al. Differences in the prospective association between individual plasma phospholipid saturated fatty acids and incident type 2 diabetes: the EPIC-InterAct case-cohort study. The lancet Diabetes & endocrinology. 2014;2(10):810-8.

32. Löfvenborg JE, Carlsson S, Andersson T, Hampe CS, Koulman A, Chirlaque Lopez MD, et al. Interaction Between GAD65 Antibodies and Dietary Fish Intake or Plasma Phospholipid n-3 Polyunsaturated Fatty Acids on Incident Adult-Onset Diabetes: The EPIC-InterAct Study. Diabetes Care. 2021;44(2):416-24.

33. Qureshi W, Santaren ID, Hanley AJ, Watkins SM, Lorenzo C, Wagenknecht LE. Risk of diabetes associated with fatty acids in the de novo lipogenesis pathway is independent of insulin sensitivity and response: the Insulin Resistance Atherosclerosis Study (IRAS). BMJ Open Diabetes Res Care. 2019;7(1):e000691.

34. Mozaffarian D, Cao H, King IB, Lemaitre RN, Song X, Siscovick DS, Hotamisligil GS. Circulating palmitoleic acid and risk of metabolic abnormalities and new-onset diabetes. Am J Clin Nutr. 2010;92(6):1350-8.

35. Prada M, Wittenbecher C, Eichelmann F, Wernitz A, Drouin-Chartier JP, Schulze MB. Association of the odd-chain fatty acid content in lipid groups with type 2 diabetes risk: A targeted analysis of lipidomics data in the EPIC-Potsdam cohort. Clinical Nutrition. 2021;40(8):4988-99.

36. Hodge AM, English DR, O'Dea K, Sinclair AJ, Makrides M, Gibson RA, Giles GG. Plasma phospholipid and dietary fatty acids as predictors of type 2 diabetes: interpreting the role of linoleic acid. Am J Clin Nutr. 2007;86(1):189-97.

37. Lu Y, Wang Y, Ong CN, Subramaniam T, Choi HW, Yuan JM, et al. Metabolic signatures and risk of type 2 diabetes in a Chinese population: an untargeted metabolomics study using both LC-MS and GC-MS. Diabetologia. 2016;59(11):2349-59.

38. Lu Y, Wang Y, Zou L, Liang X, Ong CN, Tavintharan S, et al. Serum Lipids in Association With Type 2 Diabetes Risk and Prevalence in a Chinese Population. J Clin Endocrinol Metab. 2018;103(2):671-80.

39. Seah JYH, Hong Y, Cichonska A, Sabanayagam C, Nusinovici S, Wong TY, et al. Circulating Metabolic Biomarkers Are Consistently Associated With Type 2 Diabetes Risk in Asian and European Populations. Journal of Clinical Endocrinology & Metabolism. 2022;107(7):E2751-E61.

40. Wang Q, Imamura F, Ma W, Wang M, Lemaitre RN, King IB, et al. Circulating and Dietary Trans Fatty Acids and Incident Type 2 Diabetes in Older Adults: The Cardiovascular Health Study. Diabetes Care. 2015;38(6):1099-107.

41. Ahola-Olli AV, Mustelin L, Kalimeri M, Kettunen J, Jokelainen J, Auvinen J, et al. Circulating metabolites and the risk of type 2 diabetes: a prospective study of 11,896 young adults from four Finnish cohorts. Diabetologia. 2019;62(12):2298-309.

42. Bragg F, Kartsonaki C, Guo Y, Holmes M, Du H, Yu C, et al. Circulating Metabolites and the Development of Type 2 Diabetes in Chinese Adults. Diabetes Care. 2022;45(2):477-80.

43. Chen S, Zong G, Wu Q, Yun H, Niu Z, Zheng H, et al. Associations of plasma glycerophospholipid profile with modifiable lifestyles and incident diabetes in middle-aged and older Chinese. Diabetologia. 2022;65(2):315-28.

44. Eichelmann F, Sellem L, Wittenbecher C, Jager S, Kuxhaus O, Prada M, et al. Deep Lipidomics in Human Plasma: Cardiometabolic Disease Risk and Effect of Dietary Fat Modulation. Circulation. 2022;146(1):21-35.

45. Fretts AM, Jensen PN, Hoofnagle AN, McKnight B, Howard BV, Umans J, et al. Plasma ceramides containing saturated fatty acids are associated with risk of type 2 diabetes. Journal of Lipid Research. 2021;62.

46. Harris WS, Luo J, Pottala JV, Margolis KL, Espeland MA, Robinson JG. Red Blood Cell Fatty Acids and Incident Diabetes Mellitus in the Women's Health Initiative Memory Study. PloS one. 2016;11(2):e0147894.

47. Mahendran Y, Agren J, Uusitupa M, Cederberg H, Vangipurapu J, Stancakova A, et al. Association of erythrocyte membrane fatty acids with changes in glycemia and risk of type 2 diabetes. Am J Clin Nutr. 2014;99(1):79-85.

48. Mahendran Y, Cederberg H, Vangipurapu J, Kangas AJ, Soininen P, Kuusisto J, et al. Glycerol and fatty acids in serum predict the development of hyperglycemia and type 2 diabetes in Finnish men. Diabetes Care. 2013;36(11):3732-8.

49. Pan XF, Chen ZZ, Wang TJ, Shu X, Cai H, Cai QY, et al. Plasma metabolomic signatures of obesity and risk of type 2 diabetes. Obesity.

50. Prada M, Wittenbecher C, Eichelmann F, Wernitz A, Kuxhaus O, Kroger J, et al. Plasma Industrial and Ruminant Trans Fatty Acids and Incident Type 2 Diabetes in the EPIC-Potsdam Cohort. Diabetes Care. 2022;45(4):845-53.

51. Qiu GK, Wang H, Yan Q, Ma HX, Niu RD, Lei YS, et al. A Lipid Signature with Perturbed Triacylglycerol Co-Regulation, Identified from Targeted Lipidomics, Predicts Risk for Type 2 Diabetes and Mediates the Risk from Adiposity in Two Prospective Cohorts of Chinese Adults. Clinical Chemistry. 2022;68(8):1094-107.

52. Savolainen O, Lind MV, Bergstrom G, Fagerberg B, Sandberg AS, Ross A. Biomarkers of food intake and nutrient status are associated with glucose tolerance status and development of type 2 diabetes in older Swedish women. Am J Clin Nutr. 2017;106(5):1302-10.

53. Villasanta-Gonzalez A, Alcala-Diaz JF, Vals-Delgado C, Arenas AP, Cardelo MP, Romero-Cabrera JL, et al. A plasma fatty acid profile associated to type 2 diabetes development: from the CORDIOPREV study. European Journal of Nutrition. 2022;61(2):843-57.

54. Summaries for patients. Dietary trans-palmitoleic acid and diabetes. Annals of internal medicine. 2010;153(12):I-56.

55. Charles MA, Eschwege E, Thibult N, Claude JR, Warnet JM, Rosselin GE, et al. The role of non-esterified fatty acids in the deterioration of glucose tolerance in Caucasian subjects: results of the Paris Prospective Study. Diabetologia. 1997;40(9):1101-6.

56. Khan SR, Mohan H, Liu Y, Batchuluun B, Gohil H, Al Rijjal D, et al. The discovery of novel predictive biomarkers and early-stage pathophysiology for the transition from gestational diabetes to type 2 diabetes. Diabetologia. 2019;62(4):687-703.

57. Wang L, Folsom AR, Zheng ZJ, Pankow JS, Eckfeldt JH. Plasma fatty acid composition and incidence of diabetes in middle-aged adults: the Atherosclerosis Risk in Communities (ARIC) Study. Am J Clin Nutr. 2003;78(1):91-8.

58. Al-Sulaiti H, Diboun I, Agha MV, Mohamed FFS, Atkin S, Domling AS, et al. Metabolic signature of obesity-associated insulin resistance and type 2 diabetes. Journal of Translational Medicine. 2019;17(1).

59. Arabi YM, Tamimi W, Jones G, Jawdat D, Tamim H, Al-Dorzi HM, et al. Free Fatty Acids' Level and Nutrition in Critically Ill Patients and Association with Outcomes: A Prospective Sub-Study of PermiT Trial. Nutrients. 2019;11(2).

60. Ardisson Korat AV, Li Y, Sacks F, Rosner B, Willett WC, Hu FB, Sun Q. Dairy fat intake and risk of type 2 diabetes in 3 cohorts of US men and women. Am J Clin Nutr. 2019;110(5):1192-200.

61. Barbaresko J, Rienks J, Oluwagbemigun K, Jacobs G, Lieb W, Laudes M, Nöthlings U. Dietary patterns associated with inflammatory biomarkers in a Northern German population. Eur J Nutr. 2020;59(4):1433-41.

62. Barupal DK, Baillie R, Fan S, Saykin AJ, Meikle PJ, Arnold M, et al. Sets of coregulated serum lipids are associated with Alzheimer's disease pathophysiology. Alzheimers Dement (Amst). 2019;11:619-27.

63. Cai XT, Ji LW, Liu SS, Wang MR, Heizhati M, Li NF. Derivation and Validation of a Prediction Model for Predicting the 5-Year Incidence of Type 2 Diabetes in Non-Obese Adults: A Population-Based Cohort Study. Diabetes Metabolic Syndrome and Obesity-Targets and Therapy. 2021;14:2087-101.

64. Chen PC, Chang YD, Lee MC, Hsu BG. High Serum Fibroblast Growth Factor 23 Level Is Associated With Metabolic Syndrome in Kidney Transplantation Patients. Transplant Proc. 2020.

65. Colomba J, Rabasa-Lhoret R, Bonhoure A, Bergeron C, Boudreau V, Tremblay F, et al. Dyslipidemia is not associated with the development of glucose intolerance or diabetes in cystic fibrosis. J Cyst Fibros. 2020.

66. Cordier S, Anassour-Laouan-Sidi E, Lemire M, Costet N, Lucas M, Ayotte P. Association between exposure to persistent organic pollutants and mercury, and glucose metabolism in two Canadian Indigenous populations. Environ Res. 2020;184:109345.

67. Cresci S, Zhang R, Yang Q, Duncan MS, Xanthakis V, Jiang X, et al. Genetic Architecture of Circulating Very-Long-Chain (C24:0 and C22:0) Ceramide Concentrations. J Lipid Atheroscler. 2020;9(1):172-83.

68. De Silva ST, Niriella MA, Ediriweera DS, Kottahachchi D, Kasturiratne A, de Silva AP, et al. Incidence and risk factors for metabolic syndrome among urban, adult Sri Lankans: a prospective, 7-year community cohort, follow-up study. Diabetol Metab Syndr. 2019;11:66.

69. Eriksen R, Perez IG, Posma JM, Haid M, Sharma S, Prehn C, et al. Dietary metabolite profiling brings new insight into the relationship between nutrition and metabolic risk: An IMI DIRECT study. EBioMedicine. 2020;58:102932.

70. Forslund M, Landin-Wilhelmsen K, Trimpou P, Schmidt J, Brännström M, Dahlgren E. Type 2 diabetes mellitus in women with polycystic ovary syndrome during a 24-year period: importance of obesity and abdominal fat distribution. Hum Reprod Open. 2020;2020(1):hoz042.

71. Gadowski AM, Nanayakkara N, Heritier S, Magliano DJ, Shaw JE, Curtis AJ, et al. Association between Dietary Intake and Lipid-Lowering Therapy: Prospective Analysis of Data from Australian Diabetes, Obesity, and Lifestyle Study (AusDiab) Using a Quantile Regression Approach. Nutrients. 2019;11(8).

72. Guillocheau E, Penhoat C, Drouin G, Godet A, Catheline D, Legrand P, Rioux V. Current intakes of trans-palmitoleic (trans-C16:1 n-7) and trans-vaccenic (trans-C18:1 n-7) acids in France are exclusively ensured by ruminant milk and ruminant meat: A market basket investigation. Food Chem X. 2020;5:100081.

73. Hong GB, Gao PC, Chen YY, Xia Y, Ke XS, Shao X, et al. High-Sensitivity C-Reactive Protein Leads to Increased Incident Metabolic Syndrome in Women but Not in Men: A Five-Year Follow-Up Study in a Chinese Population. Diabetes Metabolic Syndrome and Obesity-Targets and Therapy. 2020;13:581-90.

74. Iqbal K, Schwingshackl L, Floegel A, Schwedhelm C, Stelmach-Mardas M, Wittenbecher C, et al. Gaussian graphical models identified food intake networks and risk of type 2 diabetes, CVD, and cancer in the EPIC-Potsdam study. European Journal of Nutrition. 2019;58(4):1673-86.

75. Jin JL, Cao YX, Liu HH, Zhang HW, Guo YL, Wu NQ, et al. Impact of free fatty acids on prognosis in coronary artery disease patients under different glucose metabolism status. Cardiovascular diabetology. 2019;18(1):134.

76. Julibert A, Bibiloni MD, Bouzas C, Martinez-Gonzalez MA, Salas-Salvado J, Corella D, et al. Total and Subtypes of Dietary Fat Intake and Its Association with Components of the Metabolic Syndrome in a Mediterranean Population at High Cardiovascular Risk. Nutrients. 2019;11(7).

77. Kemppainen SM, Fernandes Silva L, Lankinen MA, Schwab U, Laakso M. Metabolite Signature of Physical Activity and the Risk of Type 2 Diabetes in 7271 Men. Metabolites. 2022;12(1).

78. Kraus WE, Bhapkar M, Huffman KM, Pieper CF, Krupa Das S, Redman LM, et al. 2 years of calorie restriction and cardiometabolic risk (CALERIE): exploratory outcomes of a multicentre, phase 2, randomised controlled trial. The lancet Diabetes & endocrinology. 2019;7(9):673-83.

79. Lee KW, Woo HD, Cho MJ, Park JK, Kim SS. Identification of Dietary Patterns Associated with Incidence of Hyperglycemia in Middle-Aged and Older Korean Adults. Nutrients. 2019;11(8).

80. Li Z, Yu SC, Han XY, Liu JJ, Yao HY. Changes to cardiovascular risk factors over 7 years: a prospective cohort study of in situ urbanised residents in the Chaoyang District of Beijing. Bmj Open. 2020;10(3).

81. Lim WLF, Huynh K, Chatterjee P, Martins I, Jayawardana KS, Giles C, et al. Relationships Between Plasma Lipids Species, Gender, Risk Factors, and Alzheimer's Disease. J Alzheimers Dis. 2020;76(1):303-15.

82. Mahajan UV, Varma VR, Huang CW, An Y, Tanaka T, Ferrucci L, et al. Blood Metabolite Signatures of Metabolic Syndrome in Two Cross-Cultural Older Adult Cohorts. International journal of molecular sciences. 2020;21(4).

83. Malik VS, Guasch-Ferre M, Hu FB, Townsend MK, Zeleznik OA, Eliassen AH, et al. Identification of Plasma Lipid Metabolites Associated with Nut Consumption in US Men and Women. Journal of Nutrition. 2019;149(7):1215-21.

84. Mansourian M, Yazdani A, Faghihimani E, Aminorraya A, Amini M, Jafari-Koshki T. Factors associated with progression to pre-diabetes: a recurrent events analysis. Eat Weight Disord. 2020;25(1):135-41.

85. Marklund M, Wu JHY, Imamura F, Del Gobbo LC, Fretts A, de Goede J, et al. Biomarkers of Dietary Omega-6 Fatty Acids and Incident Cardiovascular Disease and Mortality: An Individual-Level Pooled Analysis of 30 Cohort Studies. Circulation. 2019;139(21):2422-36.

86. Marlatt KL, Redman LM, Beyl RA, Smith SR, Champagne CM, Yi F, Lovejoy JC. Racial differences in body composition and cardiometabolic risk during the menopause transition: a prospective, observational cohort study. Am J Obstet Gynecol. 2020;222(4):365.e1-.e18.

87. Matsha TE, Ismail S, Speelman A, Hon GM, Davids S, Erasmus RT, Kengne AP. Visceral and subcutaneous adipose tissue association with metabolic syndrome and its components in a South African population. Clin Nutr ESPEN. 2019;32:76-81.

88. Mazidi M, Mikhailidis DP, Sattar N, Toth PP, Judd S, Blaha MJ, et al. Association of types of dietary fats and all-cause and cause-specific mortality: A prospective cohort study and meta-analysis of prospective studies with 1,164,029 participants. Clin Nutr. 2020.

89. Meddens SFW, de Vlaming R, Bowers P, Burik CAP, Linner RK, Lee C, et al. Genomic analysis of diet composition finds novel loci and associations with health and lifestyle. Molecular Psychiatry. 2021;26(6):2056-69.

90. Mellon SH, Bersani FS, Lindqvist D, Hammamieh R, Donohue D, Dean K, et al. Metabolomic analysis of male combat veterans with post traumatic stress disorder. PloS one. 2019;14(3).

91. Mey JT, Hari A, Axelrod CL, Fealy CE, Erickson ML, Kirwan JP, et al. Lipids and ketones dominate metabolism at the expense of glucose control in pulmonary arterial hypertension: a hyperglycaemic clamp and metabolomics study. Eur Respir J. 2020;55(4).

92. Murphy AM, Smith CE, Murphy LM, Follis JL, Tanaka T, Richardson K, et al. Potential Interplay between Dietary Saturated Fats and Genetic Variants of the NLRP3 Inflammasome to Modulate Insulin Resistance and Diabetes Risk: Insights from a Meta-Analysis of 19 005 Individuals. Mol Nutr Food Res. 2019;63(22):e1900226.

93. Nasser FA, Algenabi AA, Hadi NR, Hussein MK, Fatima G, Al-Aubaidy HA. The association of the common fat mass and obesity associated gene polymorphisms with type 2 diabetes in obese Iraqi population. Diabetes Metab Syndr. 2019;13(4):2451-5.

94. Ni LP, Du LY, Huang YQ, Zhou JY. Egg consumption and risk of type 2 diabetes mellitus in middle and elderly Chinese population An observational study. Medicine. 2020;99(16).

95. Palomino-Schätzlein M, Mayneris-Perxachs J, Caballano-Infantes E, Rodríguez MA, Palomo-Buitrago ME, Xiao X, et al. Combining metabolic profiling of plasma and faeces as a fingerprint of insulin resistance in obesity. Clin Nutr. 2020;39(7):2292-300.

96. Pertiwi K, Küpers LK, Geleijnse JM, Zock PL, Wanders AJ, Kruger HS, et al. Associations of linoleic acid with markers of glucose metabolism and liver function in South African adults. Lipids in health and disease. 2020;19(1):138.

97. Román GC, Jackson RE, Gadhia R, Román AN, Reis J. Mediterranean diet: The role of long-chain ω-3 fatty acids in fish; polyphenols in fruits, vegetables, cereals, coffee, tea, cacao and wine; probiotics and vitamins in prevention of stroke, age-related cognitive decline, and Alzheimer disease. Rev Neurol (Paris). 2019;175(10):724-41.

98. Sanna S, van Zuydam NR, Mahajan A, Kurilshikov A, Vila AV, Vosa U, et al. Causal relationships among the gut microbiome, short-chain fatty acids and metabolic diseases. Nature Genetics. 2019;51(4):600-+.

99. Simila ME, Kontto JP, Virtamo J, Hatonen KA, Valsta LM, Sundvall J, Mannisto S. Insulin-like growth factor I, binding proteins-1 and-3, risk of type 2 diabetes and macronutrient intakes in men. British Journal of Nutrition. 2019;121(8):938-44.

100. Singh RG, Nguyen NN, Cervantes A, Cho J, Petrov MS. Serum lipid profile as a biomarker of intra-pancreatic fat deposition: A nested cross-sectional study. Nutrition, metabolism, and cardiovascular diseases : NMCD. 2019;29(9):956-64.

101. Spiller S, Blueher M, Hoffmann R. Plasma levels of free fatty acids correlate with type 2 diabetes mellitus. Diabetes Obesity & Metabolism. 2018;20(11):2661-9.

102. Stevens VL, Carter BD, McCullough ML, Campbell PT, Wang Y. Metabolomic Profiles Associated with BMI, Waist Circumference, and Diabetes and Inflammation Biomarkers in Women. Obesity (Silver Spring). 2020;28(1):187-96.

103. Sun DK, Tiedt S, Yu B, Jian XQ, Gottesman RF, Mosley TH, et al. A prospective study of serum metabolites and risk of ischemic stroke. Neurology. 2019;92(16):E1890-E8.

104. Tricò D, Mengozzi A, Nesti L, Hatunic M, Gabriel Sanchez R, Konrad T, et al. Circulating palmitoleic acid is an independent determinant of insulin sensitivity, beta cell function and glucose tolerance in non-diabetic individuals: a longitudinal analysis. Diabetologia. 2020;63(1):206-18.

105. Wedekind R, Kiss A, Keski-Rahkonen P, Viallon V, Rothwell JA, Cross AJ, et al. A metabolomic study of red and processed meat intake and acylcarnitine concentrations in human urine and blood. Am J Clin Nutr. 2020;112(2):381-8.

106. Xie G, Wang X, Wei R, Wang J, Zhao A, Chen T, et al. Serum metabolite profiles are associated with the presence of advanced liver fibrosis in Chinese patients with chronic hepatitis B viral infection. BMC Med. 2020;18(1):144.

107. Abiemo EE, Alonso A, Nettleton JA, Steffen LM, Bertoni AG, Jain A, Lutsey PL. Relationships of the Mediterranean dietary pattern with insulin resistance and diabetes incidence in the Multi-Ethnic Study of Atherosclerosis (MESA). Br J Nutr. 2013;109(8):1490-7.

108. Abulmeaty MMA, Aljuraiban GS, Alaidarous TA, Alkahtani NM. Body Composition and the Components of Metabolic Syndrome in Type 2 Diabetes: The Roles of Disease Duration and Glycemic Control. Diabetes Metabolic Syndrome and Obesity-Targets and Therapy. 2020;13:1051-9.

109. Ahmadi-Abhari S, Luben RN, Powell N, Bhaniani A, Chowdhury R, Wareham NJ, et al. Dietary intake of carbohydrates and risk of type 2 diabetes: the European Prospective Investigation into Cancer-Norfolk study. British Journal of Nutrition. 2014;111(2):342-52.

110. Akasaka H, Ohnishi H, Narita Y, Kameda M, Miki T, Takahashi H, et al. The Serum Level of KL-6 Is Associated with the Risk of Insulin Resistance and New-onset Diabetes Mellitus: The Tanno-Sobetsu Study. Internal medicine (Tokyo, Japan). 2017;56(22):3009-18.

111. Aleksandrova K, Drogan D, Weikert C, Schulze MB, Fritsche A, Boeing H, Pischon T. Fatty Acid-Binding Protein 4 and Risk of Type 2 Diabetes, Myocardial Infarction, and Stroke: A Prospective Cohort Study. J Clin Endocrinol Metab. 2019;104(12):5991-6002.

112. Almoosawi S, Prynne CJ, Hardy R, Stephen AM. Diurnal eating rhythms: association with long-term development of diabetes in the 1946 British birth cohort. Nutrition, metabolism, and cardiovascular diseases : NMCD. 2013;23(10):1025-30.

113. Anjana RM, Sudha V, Nair DH, Lakshmipriya N, Deepa M, Pradeepa R, et al. Diabetes in Asian Indians-How much is preventable? Ten-year follow-up of the Chennai Urban Rural Epidemiology Study (CURES-142). Diabetes Res Clin Pract. 2015;109(2):253-61.

114. Ayubi E, Khalili D, Delpisheh A, Hadaegh F, Azizi F. Factor analysis of metabolic syndrome components and predicting type 2 diabetes: Results of 10-year follow-up in a Middle Eastern population. Journal of Diabetes. 2015;7(6):830-8.

115. Baghdasarian S, Lin HP, Pickering RT, Mott MM, Singer MR, Bradlee ML, Moore LL. Dietary Cholesterol Intake Is Not Associated with Risk of Type 2 Diabetes in the Framingham Offspring Study. Nutrients. 2018;10(6).

116. Bao W, Li S, Chavarro JE, Tobias DK, Zhu Y, Hu FB. Adherence to Low-Carbohydrate Dietary Pattern and Long-Term Risk of Type 2 Diabetes among Women with a History of Gestational Diabetes: A Prospective Cohort Study. Diabetes. 2015;64:A423-A.

117. Bhavadharini B, Dehghan M, Mente A, Rangarajan S, Sheridan P, Mohan V, et al. Association of dairy consumption with metabolic syndrome, hypertension and diabetes in 147 812 individuals from 21 countries. BMJ Open Diabetes Res Care. 2020;8(1).

118. Bhavadharini B, Dehghan M, Mente A, Rangarajan S, Sheridan P, Mohan V, et al. Association of dairy consumption with metabolic syndrome, hypertension and diabetes in 147 812 individuals from 21 countries. Bmj Open Diabetes Research & Care. 2020;8(1).

119. Bidel S, Silventoinen K, Hu G, Lee DH, Kaprio J, Tuomilehto J. Coffee consumption, serum gamma-glutamyltransferase and risk of type II diabetes. European journal of clinical nutrition. 2008;62(2):178-85.

120. Bozorgmanesh M, Hadaegh F, Ghaffari S, Harati H, Azizi F. A simple risk score effectively predicted type 2 diabetes in Iranian adult population: population-based cohort study. European Journal of Public Health. 2011;21(5):554-9.

121. Brahimaj A, Rivadeneira F, Muka T, Sijbrands EJG, Franco OH, Dehghan A, Kavousi M. Novel metabolic indices and incident type 2 diabetes among women and men: the Rotterdam Study. Diabetologia. 2019;62(9):1581-90.

122. Brayner B, Kaur G, Keske MA, Perez-Cornago A, Piernas C, Livingstone KM. Dietary Patterns Characterized by Fat Type in Association with Obesity and Type 2 Diabetes: A Longitudinal Study of UK Biobank Participants. Journal of Nutrition. 2021;151(11):3570-8.

123. Brostow DP, Odegaard AO, Koh WP, Duval S, Gross MD, Yuan JM, Pereira MA. Omega-3 fatty acids and incident type 2 diabetes: the Singapore Chinese Health Study. Am J Clin Nutr. 2011;94(2):520-6.

124. Brouwer-Brolsma EM, van Woudenbergh GJ, Elferink SJWHO, Singh-Povel CM, Hofman A, Dehghan A, et al. Intake of different types of dairy and its prospective association with risk of type 2 diabetes: The Rotterdam Study. Nutrition Metabolism and Cardiovascular Diseases. 2016;26(11):987-95.

125. Brouwers M, de Graaf J, Simons N, Meex S, Ten Doeschate S, van Heertum S, et al. Incidence of type 2 diabetes in familial combined hyperlipidemia. BMJ Open Diabetes Res Care. 2020;8(1).

126. Brunner EJ, Mosdol A, Witte DR, Martikainen P, Stafford M, Shipley MJ, Marmot MG. Dietary patterns and 15-y risks of major coronary events, diabetes, and mortality. Am J Clin Nutr. 2008;87(5):1414-21.

127. Cahill LE, Pan A, Chiuve SE, Sun Q, Willett WC, Hu FB, Rimm EB. Fried-food consumption and risk of type 2 diabetes and coronary artery disease: a prospective study in 2 cohorts of US women and men. Am J Clin Nutr. 2014;100(2):667-75.

128. Cai X, Zhu Q, Cao Y, Liu S, Wang M, Wu T, et al. A Prediction Model Based on Noninvasive Indicators to Predict the 8-Year Incidence of Type 2 Diabetes in Patients with Nonalcoholic Fatty Liver Disease: A Population-Based Retrospective Cohort Study. Biomed Res Int. 2021;2021:5527460.

129. Camargo A, Jimenez-Lucena R, Alcala-Diaz JF, Rangel-Zuniga OA, Garcia-Carpintero S, Lopez-Moreno J, et al. Postprandial endotoxemia may influence the development of type 2 diabetes mellitus: From the CORDIOPREV study. Clinical Nutrition. 2019;38(2):529-38.

130. Cejudo MGJ, Cruijsen E, Heuser C, Soedamah-Muthu SS, Voortman T, Geleijnse JM. Dairy Consumption and 3-Year Risk of Type 2 Diabetes after Myocardial Infarction: A Prospective Analysis in the Alpha Omega Cohort. Nutrients. 2021;13(9).

131. Chen M, Sun Q, Giovannucci E, Mozaffarian D, Manson JE, Willett WC, Hu FB. Dairy consumption and risk of type 2 diabetes: 3 cohorts of US adults and an updated meta-analysis. BMC Med. 2014;12:215.

132. Choi HK, Willett WC, Stampfer MJ, Rimm E, Hu FB. Dairy consumption and risk of type 2 diabetes mellitus in men: a prospective study. Archives of internal medicine. 2005;165(9):997-1003.

133. Chow LS, Li S, Eberly LE, Seaquist ER, Eckfeldt JH, Hoogeveen RC, et al. Estimated plasma stearoyl co-A desaturase-1 activity and risk of incident diabetes: the Atherosclerosis Risk in Communities (ARIC) study. Metabolism: clinical and experimental. 2013;62(1):100-8.

134. Currenti W, Godos J, Alanazi AM, Grosso G, Cincione RI, La Vignera S, et al. Dietary Fats and Cardio-Metabolic Outcomes in a Cohort of Italian Adults. Nutrients. 2022;14(20).

135. Dagogo-Jack S, Edeoga C, Ebenibo S, Nyenwe E, Wan J. Lack of racial disparity in incident prediabetes and glycemic progression among black and white offspring of parents with type 2 diabetes: the pathobiology of prediabetes in a biracial cohort (POP-ABC) study. J Clin Endocrinol Metab. 2014;99(6):E1078-87.

136. de Koning L, Chiuve SE, Fung TT, Willett WC, Rimm EB, Hu FB. Diet-quality scores and the risk of type 2 diabetes in men. Diabetes Care. 2011;34(5):1150-6.

137. de Koning L, Fung TT, Liao X, Chiuve SE, Rimm EB, Willett WC, et al. Low-carbohydrate diet scores and risk of type 2 diabetes in men. Am J Clin Nutr. 2011;93(4):844-50.

138. de Simone G, Wang W, Best LG, Yeh F, Izzo R, Mancusi C, et al. Target organ damage and incident type 2 diabetes mellitus: the Strong Heart Study. Cardiovascular diabetology. 2017;16(1):64.

139. de Souza RJ, Dehghan M, Mente A, Bangdiwala SI, Ahmed SH, Alhabib KF, et al. Association of nut intake with risk factors, cardiovascular disease, and mortality in 16 countries from 5 continents: analysis from the Prospective Urban and Rural Epidemiology (PURE) study. American Journal of Clinical Nutrition. 2020;112(1):208-19.

140. Diaz-Lopez A, Bullo M, Martinez-Gonzalez MA, Corella D, Estruch R, Fito M, et al. Dairy product consumption and risk of type 2 diabetes in an elderly Spanish Mediterranean population at high cardiovascular risk. Eur J Nutr. 2016;55(1):349-60.

141. Djousse L, Gaziano JM. Plasma levels of FABP4, but not FABP3, are associated with increased risk of diabetes. Lipids. 2012;47(8):757-62.

142. Djousse L, Gaziano JM, Buring JE, Lee IM. Egg consumption and risk of type 2 diabetes in men and women. Diabetes Care. 2009;32(2):295-300.

143. Djousse L, Gaziano JM, Buring JE, Lee IM. Dietary omega-3 fatty acids and fish consumption and risk of type 2 diabetes. Am J Clin Nutr. 2011;93(1):143-50.

144. Djousse L, Kamineni A, Nelson TL, Carnethon M, Mozaffarian D, Siscovick D, Mukamal KJ. Egg consumption and risk of type 2 diabetes in older adults. Am J Clin Nutr. 2010;92(2):422-7.

145. Djousse L, Khawaja O, Bartz TM, Biggs ML, Ix JH, Zieman SJ, et al. Plasma fatty acid-binding protein 4, nonesterified fatty acids, and incident diabetes in older adults. Diabetes Care. 2012;35(8):1701-7.

146. Dow C, Mangin M, Balkau B, Affret A, Boutron-Ruault MC, Clavel-Chapelon F, et al. Fatty acid consumption and incident type 2 diabetes: an 18-year follow-up in the female E3N (Etude Epidemiologique aupres des femmes de la Mutuelle Generale de l'Education Nationale) prospective cohort study. Br J Nutr. 2016:1-9.

147. Drehmer M, Pereira MA, Schmidt MI, Del Carmen BMM, Alvim S, Lotufo PA, Duncan BB. Associations of dairy intake with glycemia and insulinemia, independent of obesity, in Brazilian adults: the Brazilian Longitudinal Study of Adult Health (ELSA-Brasil). Am J Clin Nutr. 2015;101(4):775-82.

148. Drouin-Chartier JP, Hernandez-Alonso P, Guasch-Ferre M, Ruiz-Canela M, Li J, Wittenbecher C, et al. Dairy consumption, plasma metabolites, and risk of type 2 diabetes. American Journal of Clinical Nutrition. 2021;114(1):163-74.

149. Du HD, Guo Y, Bennett DA, Bragg F, Bian Z, Chadni M, et al. Red meat, poultry and fish consumption and risk of diabetes: a 9 year prospective cohort study of the China Kadoorie Biobank. Diabetologia. 2020;63(4):767-79.

150. Ericson U, Brunkwall L, Alves Dias J, Drake I, Hellstrand S, Gullberg B, et al. Food patterns in relation to weight change and incidence of type 2 diabetes, coronary events and stroke in the Malmö Diet and Cancer cohort. Eur J Nutr. 2019;58(5):1801-14.

151. Ericson U, Hellstrand S, Brunkwall L, Schulz CA, Sonestedt E, Wallstrom P, et al. Food sources of fat may clarify the inconsistent role of dietary fat intake for incidence of type 2 diabetes. Am J Clin Nutr. 2015;101(5):1065-80.

152. Feskens EJ, Virtanen SM, Rasanen L, Tuomilehto J, Stengard J, Pekkanen J, et al. Dietary factors determining diabetes and impaired glucose tolerance. A 20-year follow-up of the Finnish and Dutch cohorts of the Seven Countries Study. Diabetes Care. 1995;18(8):1104-12.

153. Gaeini Z, Bahadoran Z, Mirmiran P, Djazayery A. The Association between Dietary Fat Pattern and the Risk of Type 2 Diabetes. Preventive nutrition and food science. 2019;24(1):1-7.

154. Greenberg JA, Jiang X, Tinker LF, Snetselaar LG, Saquib N, Shadyab AH. Eggs, dietary cholesterol, choline, betaine, and diabetes risk in the Women's Health Initiative: a prospective analysis. Am J Clin Nutr. 2021;114(1):368-77.

155. Guasch-Ferre M, Becerra-Tomas N, Ruiz-Canela M, Corella D, Schroder H, Estruch R, et al. Total and subtypes of dietary fat intake and risk of type 2 diabetes mellitus in the Prevencion con Dieta Mediterranea (PREDIMED) study. Am J Clin Nutr. 2017;105(3):723-35.

156. Guasch-Ferre M, Hernandez-Alonso P, Drouin-Chartier JP, Ruiz-Canela M, Razquin C, Toledo E, et al. Walnut Consumption, Plasma Metabolomics, and Risk of Type 2 Diabetes and Cardiovascular Disease. Journal of Nutrition. 2021;151(2):303-11.

157. Guasch-Ferre M, Hruby A, Salas-Salvado J, Martinez-Gonzalez MA, Sun Q, Willett WC, Hu FB. Olive oil consumption and risk of type 2 diabetes in US women. Am J Clin Nutr. 2015;102(2):479-86.

158. Ha K, Joung H, Song Y. Inadequate fat or carbohydrate intake was associated with an increased incidence of type 2 diabetes mellitus in Korean adults: A 12-year community-based prospective cohort study. Diabetes Research and Clinical Practice. 2019;148:254-61.

159. Halton TL, Liu S, Manson JE, Hu FB. Low-carbohydrate-diet score and risk of type 2 diabetes in women. Am J Clin Nutr. 2008;87(2):339-46.

160. Halton TL, Willett WC, Liu SM, Manson JE, Stampfer MJ, Hu FB. Potato and french fry consumption and risk of type 2 diabetes in women. American Journal of Clinical Nutrition. 2006;83(2):284-90.

161. Harding AH, Day NE, Khaw KT, Bingham S, Luben R, Welsh A, Wareham NJ. Dietary fat and the risk of clinical type 2 diabetes: the European prospective investigation of Cancer-Norfolk study. Am J Epidemiol. 2004;159(1):73-82.

162. Hodge AM, English DR, O'Dea K, Giles GG. Dietary patterns and diabetes incidence in the Melbourne Collaborative Cohort Study. Am J Epidemiol. 2007;165(6):603-10.

163. Hruby A, Ma J, Rogers G, Meigs JB, Jacques PF. Associations of Dairy Intake with Incident Prediabetes or Diabetes in Middle-Aged Adults Vary by Both Dairy Type and Glycemic Status. J Nutr. 2017;147(9):1764-75.

164. Hu FB, Manson JE, Stampfer MJ, Colditz G, Liu S, Solomon CG, Willett WC. Diet, lifestyle, and the risk of type 2 diabetes mellitus in women. The New England journal of medicine. 2001;345(11):790-7.

165. Hu H, Wang J, Han X, Li YR, Miao XP, Yuan J, et al. Prediction of 5-year risk of diabetes mellitus in relatively low risk middle-aged and elderly adults. Acta Diabetologica. 2020;57(1):63-70.

166. Ibsen DB, Laursen ASD, Lauritzen L, Tjonneland A, Overvad K, Jakobsen MU. Substitutions between dairy product subgroups and risk of type 2 diabetes: the Danish Diet, Cancer and Health cohort. Br J Nutr. 2017;118(11):989-97.

167. Ibsen DB, Overvad K, Laursen ASD, Halkjaer J, Tjonneland A, Kilpelainen TO, et al. Changes in intake of dairy product subgroups and risk of type 2 diabetes: modelling specified food substitutions in the Danish Diet, Cancer and Health cohort. European Journal of Nutrition. 2021;60(6):3449-59.

168. Ibsen DB, Warberg CK, Würtz AML, Overvad K, Dahm CC. Substitution of red meat with poultry or fish and risk of type 2 diabetes: a Danish cohort study. Eur J Nutr. 2019;58(7):2705-12.

169. Il'yasova D, Wang F, D'Agostino RB, Jr., Hanley A, Wagenknecht LE. Prospective association between fasting NEFA and type 2 diabetes: impact of post-load glucose. Diabetologia. 2010;53(5):866-74.

170. Imamura F, Lichtenstein AH, Dallal GE, Meigs JB, Jacques PF. Generalizability of dietary patterns associated with incidence of type 2 diabetes mellitus. American Journal of Clinical Nutrition. 2009;90(4):1075-83.

171. Imamura F, Sharp SJ, Koulman A, Schulze MB, Kroeger J, Griffin JL, et al. A combination of plasma phospholipid fatty acids and its association with incidence of type 2 diabetes: The EPIC-InterAct case-cohort study. Plos Medicine. 2017;14(10).

172. Jiang R, Manson JE, Stampfer MJ, Liu S, Willett WC, Hu FB. Nut and peanut butter consumption and risk of type 2 diabetes in women. Jama. 2002;288(20):2554-60.

173. Johansson I, Esberg A, Nilsson LM, Jansson JH, Wennberg P, Winkvist A. Dairy Product Intake and Cardiometabolic Diseases in Northern Sweden: A 33-Year Prospective Cohort Study. Nutrients. 2019;11(2).

174. Kaushik M, Mozaffarian D, Spiegelman D, Manson JE, Willett WC, Hu FB. Long-chain omega-3 fatty acids, fish intake, and the risk of type 2 diabetes mellitus. Am J Clin Nutr. 2009;90(3):613-20.

175. Khalili-Moghadam S, Mirmiran P, Bahadoran Z, Azizi F. The Mediterranean diet and risk of type 2 diabetes in Iranian population. European journal of clinical nutrition. 2019;73(1):72-8.

176. Kiyohara Y, Shinohara A, Kato I, Shirota T, Kubo M, Tanizaki Y, et al. Dietary factors and development of impaired glucose tolerance and diabetes in a general Japanese population: the hisayama study. Journal of epidemiology. 2003;13(5):251-8.

177. Koloverou E, Panagiotakos DB, Pitsavos C, Chrysohoou C, Georgousopoulou EN, Pitaraki E, et al. 10-year incidence of diabetes and associated risk factors in Greece: the ATTICA study (2002-2012). The review of diabetic studies : RDS. 2014;11(2):181-9.

178. Konishi K, Wada K, Yamakawa M, Goto Y, Mizuta F, Koda S, et al. Dietary Soy Intake Is Inversely Associated with Risk of Type 2 Diabetes in Japanese Women but Not in Men. J Nutr. 2019;149(7):1208-14.

179. Kouvari M, Panagiotakos DB, Chrysohoou C, Georgousopoulou EN, Yannakoulia M, Tousoulis D, et al. Dairy products, surrogate markers, and cardiovascular disease; a sex-specific analysis from the ATTICA prospective study. Nutrition Metabolism and Cardiovascular Diseases. 2020;30(12):2194-206.

180. Kroeger J, Jacobs S, Jansen EHJM, Fritsche A, Boeing H, Schulze MB. Erythrocyte membrane fatty acid fluidity and risk of type 2 diabetes in the EPIC-Potsdam study. Diabetologia. 2015;58(2):282-9.

181. Kummer K, Jensen PN, Kratz M, Lemaitre RN, Howard BV, Cole SA, Fretts AM. Full-Fat Dairy Food Intake is Associated with a Lower Risk of Incident Diabetes Among American Indians with Low Total Dairy Food Intake. J Nutr. 2019;149(7):1238-44.

182. Kurotani K, Nanri A, Goto A, Mizoue T, Noda M, Oba S, et al. Red meat consumption is associated with the risk of type 2 diabetes in men but not in women: a Japan Public Health Center-based Prospective Study. British Journal of Nutrition. 2013;110(10):1910-8.

183. Kurotani K, Nanri A, Goto A, Mizoue T, Noda M, Oba S, et al. Cholesterol and egg intakes and the risk of type 2 diabetes: the Japan Public Health Center-based Prospective Study. Br J Nutr. 2014;112(10):1636-43.

184. Laaksonen DE, Lakka TA, Lakka HM, Nyyssonen K, Rissanen T, Niskanen LK, Salonen JT. Serum fatty acid composition predicts development of impaired fasting glycaemia and diabetes in middle-aged men. Diabetic medicine : a journal of the British Diabetic Association. 2002;19(6):456-64.

185. Lacoppidan SA, Kyro C, Loft S, Helnaes A, Christensen J, Hansen CP, et al. Adherence to a Healthy Nordic Food Index Is Associated with a Lower Risk of Type-2 Diabetes-The Danish Diet, Cancer and Health Cohort Study. Nutrients. 2015;7(10):8633-44.

186. Lajous M, Bijon A, Fagherazzi G, Balkau B, Boutron-Ruault MC, Clavel-Chapelon F. Egg and cholesterol intake and incident type 2 diabetes among French women. Br J Nutr. 2015;114(10):1667-73.

187. Lajous M, Tondeur L, Fagherazzi G, de Lauzon-Guillain B, Boutron-Ruaualt MC, Clavel-Chapelon F. Processed and unprocessed red meat consumption and incident type 2 diabetes among French women. Diabetes Care. 2012;35(1):128-30.

188. Lamri A, Bonnefond A, Meyre D, Balkau B, Roussel R, Marre M, et al. Interaction between GPR120 p.R270H loss-of-function variant and dietary fat intake on incident type 2 diabetes risk in the D.E.S.I.R. study. Nutrition, metabolism, and cardiovascular diseases : NMCD. 2016;26(10):931-6.

189. Lamri A, Khalil CA, Jaziri R, Velho G, Lantieri O, Vol S, et al. Dietary fat intake and polymorphisms at the PPARG locus modulate BMI and type 2 diabetes risk in the D.E.S.I.R. prospective study. International Journal of Obesity. 2012;36(2):218-24.

190. Li Q, Zhao M, Wang Y, Zhong F, Liu J, Gao L, Zhao J. Associations Between Serum Free Fatty Acid Levels and Incident Diabetes in a 3-Year Cohort Study. Diabetes Metab Syndr Obes. 2021;14:2743-51.

191. Lindstrom J, Peltonen M, Eriksson JG, Louheranta A, Fogelholm M, Uusitupa M, Tuomilehto J. High-fibre, low-fat diet predicts long-term weight loss and decreased type 2 diabetes risk: the Finnish Diabetes Prevention Study. Diabetologia. 2006;49(5):912-20.

192. Liu S, Choi HK, Ford E, Song Y, Klevak A, Buring JE, Manson JE. A prospective study of dairy intake and the risk of type 2 diabetes in women. Diabetes Care. 2006;29(7):1579-84.

193. Liu SX, van der Schouw YT, Soedamah-Muthu SS, Spijkerman AMW, Sluijs I. Intake of dietary saturated fatty acids and risk of type 2 diabetes in the European Prospective Investigation into Cancer and Nutrition-Netherlands cohort: associations by types, sources of fatty acids and substitution by macronutrients. European Journal of Nutrition. 2019;58(3):1125-36.

194. Lofvenborg JE, Ahlqvist E, Alfredsson L, Andersson T, Groop L, Tuomi T, et al. Consumption of red meat, genetic susceptibility, and risk of LADA and type 2 diabetes. European Journal of Nutrition.

195. Lu J, Lam SM, Wan Q, Shi L, Huo Y, Chen L, et al. High-Coverage Targeted Lipidomics Reveals Novel Serum Lipid Predictors and Lipid Pathway Dysregulation Antecedent to Type 2 Diabetes Onset in Normoglycemic Chinese Adults. Diabetes Care. 2019;42(11):2117-26.

196. Ma W, Wu JH, Wang Q, Lemaitre RN, Mukamal KJ, Djousse L, et al. Prospective association of fatty acids in the de novo lipogenesis pathway with risk of type 2 diabetes: the Cardiovascular Health Study. Am J Clin Nutr. 2015;101(1):153-63.

197. Mandalazi E, Drake I, Wirfalt E, Orho-Melander M, Sonestedt E. A High Diet Quality Based on Dietary Recommendations Is Not Associated with Lower Incidence of Type 2 Diabetes in the Malmo Diet and Cancer Cohort. International journal of molecular sciences. 2016;17(6).

198. Margolis KL, Wei F, de Boer IH, Howard BV, Liu S, Manson JE, et al. A diet high in low-fat dairy products lowers diabetes risk in postmenopausal women. J Nutr. 2011;141(11):1969-74.

199. Mari-Sanchis A, Gea A, Basterra-Gortari FJ, Martinez-Gonzalez MA, Beunza JJ, Bes-Rastrollo M. Meat Consumption and Risk of Developing Type 2 Diabetes in the SUN Project: A Highly Educated Middle-Class Population. PloS one. 2016;11(7):e0157990.

200. Martinez-Gonzalez MA, de la Fuente-Arrillaga C, Nunez-Cordoba JM, Basterra-Gortari FJ, Beunza JJ, Vazquez Z, et al. Adherence to Mediterranean diet and risk of developing diabetes: prospective cohort study. Bmj-British Medical Journal. 2008;336(7657):1348-51.

201. Meyer KA, Kushi LH, Jacobs DR, Jr., Folsom AR. Dietary fat and incidence of type 2 diabetes in older Iowa women. Diabetes Care. 2001;24(9):1528-35.

202. Miyamori D, Tanaka M, Furuhashi M, Ohnishi H, Koyama M, Osanami A, et al. Prediction of new onset of diabetes mellitus during a 10-year period by using a combination of levels of alanine aminotransferase and gamma-glutamyl transferase. Endocrine Journal. 2021;68(12):1391-402.

203. Montonen J, Jarvinen R, Heliovaara M, Reunanen A, Aromaa A, Knekt P. Food consumption and the incidence of type II diabetes mellitus. European journal of clinical nutrition. 2005;59(3):441-8.

204. Mtintsilana A, Micklesfield LK, Chorell E, Olsson T, Goedecke JH. Fat redistribution and accumulation of visceral adipose tissue predicts type 2 diabetes risk in middle-aged black South African women: a 13-year longitudinal study. Nutrition & Diabetes. 2019;9.

205. Murthy VL, Nayor M, Carnethon M, Reis JP, Lloyd-Jones D, Allen NB, et al. Circulating metabolite profile in young adulthood identifies long-term diabetes susceptibility: the Coronary Artery Risk Development in Young Adults (CARDIA) study. Diabetologia. 2022;65(4):657-74.

206. Nanri A, Mizoue T, Noda M, Takahashi Y, Matsushita Y, Poudel-Tandukar K, et al. Fish intake and type 2 diabetes in Japanese men and women: the Japan Public Health Center-based Prospective Study. Am J Clin Nutr. 2011;94(3):884-91.

207. Niu Z, Wu Q, Sun L, Qi Q, Zheng H, Li H, et al. Circulating Glycerolipids, Fatty Liver Index, and Incidence of Type 2 Diabetes: A Prospective Study Among Chinese. J Clin Endocrinol Metab. 2021;106(7):2010-20.

208. O'Connor LM, Lentjes MA, Luben RN, Khaw KT, Wareham NJ, Forouhi NG. Dietary dairy product intake and incident type 2 diabetes: a prospective study using dietary data from a 7-day food diary. Diabetologia. 2014;57(5):909-17.

209. Palli D, InterAct C. Association between dietary meat consumption and incident type 2 diabetes: the EPIC-InterAct study. Diabetologia. 2013;56(1):47-59.

210. Pan A, Sun Q, Bernstein AM, Schulze MB, Manson JE, Willett WC, Hu FB. Red meat consumption and risk of type 2 diabetes: 3 cohorts of US adults and an updated meta-analysis. Am J Clin Nutr. 2011;94(4):1088-96.

211. Pan A, Sun Q, Manson JE, Willett WC, Hu FB. Walnut consumption is associated with lower risk of type 2 diabetes in women. J Nutr. 2013;143(4):512-8.

212. Pankow JS, Duncan BB, Schmidt MI, Ballantyne CM, Couper D, Hoogeveen RC, Golden SH. Fasting plasma free fatty acids and risk of type 2 diabetes - The atherosclerosis risk in communities study. Diabetes Care. 2004;27(1):77-82.

213. Pastorino S, Richards M, Pierce M, Ambrosini GL. A high-fat, high-glycaemic index, low-fibre dietary pattern is prospectively associated with type 2 diabetes in a British birth cohort. Br J Nutr. 2016;115(9):1632-42.

214. Patel PS, Forouhi NG, Kuijsten A, Schulze MB, van Woudenbergh GJ, Ardanaz E, et al. The prospective association between total and type of fish intake and type 2 diabetes in 8 European countries: EPIC-InterAct Study. Am J Clin Nutr. 2012;95(6):1445-53.

215. Pishgar F, Shabani M, Quinaglia ACST, Bluemke DA, Budoff M, Barr RG, et al. Adipose tissue biomarkers and type 2 diabetes incidence in normoglycemic participants in the MESArthritis Ancillary Study: A cohort study. PLoS Med. 2021;18(7):e1003700.

216. Ramezan M, Asghari G, Mirmiran P, Tahmasebinejad Z, Azizi F. Mediterranean dietary patterns and risk of type 2 diabetes in the Islamic Republic of Iran. East Mediterr Health J. 2019;25(12):896-904.

217. Rylander C, Sandanger TM, Engeset D, Lund E. Consumption of lean fish reduces the risk of type 2 diabetes mellitus: a prospective population based cohort study of Norwegian women. PloS one. 2014;9(2):e89845.

218. Sakurai M, Nakamura K, Miura K, Takamura T, Yoshita K, Sasaki S, et al. Family history of diabetes, lifestyle factors, and the 7-year incident risk of type 2 diabetes mellitus in middle-aged Japanese men and women. Journal of Diabetes Investigation. 2013;4(3):261-8.

219. Salmeron J, Hu FB, Manson JE, Stampfer MJ, Colditz GA, Rimm EB, Willett WC. Dietary fat intake and risk of type 2 diabetes in women. Am J Clin Nutr. 2001;73(6):1019-26.

220. Satija A, Bhupathiraju SN, Rimm EB, Spiegelman D, Chiuve SE, Borgi L, et al. Plant-Based Dietary Patterns and Incidence of Type 2 Diabetes in US Men and Women: Results from Three Prospective Cohort Studies. PLoS Med. 2016;13(6):e1002039.

221. Schulze MB, Manson JE, Willett WC, Hu FB. Processed meat intake and incidence of Type 2 diabetes in younger and middle-aged women. Diabetologia. 2003;46(11):1465-73.

222. Schulze MB, Schulz M, Heidemann C, Schienkiewitz A, Hoffmann K, Boeing H. Carbohydrate intake and incidence of type 2 diabetes in the European Prospective Investigation into Cancer and Nutrition (EPIC)-Potsdam Study. Br J Nutr. 2008;99(5):1107-16.

223. Shan R, Duan W, Liu L, Qi J, Gao J, Zhang Y, et al. Low-Carbohydrate, High-Protein, High-Fat Diets Rich in Livestock, Poultry and Their Products Predict Impending Risk of Type 2 Diabetes in Chinese Individuals that Exceed Their Calculated Caloric Requirement. Nutrients. 2018;10(1).

224. Shi L, Brunius C, Bergdahl IA, Johansson I, Rolandsson O, Donat Vargas C, et al. Joint Analysis of Metabolite Markers of Fish Intake and Persistent Organic Pollutants in Relation to Type 2 Diabetes Risk in Swedish Adults. J Nutr. 2019;149(8):1413-23.

225. Shitole SG, Biggs ML, Ix JH, Fretts AM, Tracy RP, Siscovick DS, et al. Fasting and Postload Nonesterified Fatty Acids and Glucose Dysregulation in Older Adults. Am J Epidemiol. 2022;191(7):1235-47.

226. Shuai Y, Liu MW, He QQ, Larsson SC. Egg, cholesterol and protein intake and incident type 2 diabetes mellitus: Results of repeated measurements from a prospective cohort study. Clinical Nutrition. 2021;40(6):4180-6.

227. Simila ME, Kontto JP, Valsta LM, Mannisto S, Albanes D, Virtamo J. Carbohydrate substitution for fat or protein and risk of type 2 diabetes in male smokers. European journal of clinical nutrition. 2012;66(6):716-21.

228. Sluijs I, Forouhi NG, Beulens JWJ, van der Schouw YT, Agnoli C, Arriola L, et al. The amount and type of dairy product intake and incident type 2 diabetes: results from the EPIC-InterAct Study. American Journal of Clinical Nutrition. 2012;96(2):382-90.

229. Slurink IAL, Voortman T, Ochoa-Rosales C, Ahmadizar F, Kavousi M, Kupper N, et al. Dairy Product Consumption in Relation to Incident Prediabetes and Longitudinal Insulin Resistance in the Rotterdam Study. Nutrients. 2022;14(3).

230. Soedamah-Muthu SS, Masset G, Verberne L, Geleijnse JM, Brunner EJ. Consumption of dairy products and associations with incident diabetes, CHD and mortality in the Whitehall II study. Br J Nutr. 2013;109(4):718-26.

231. Song Y, Buring JE, Manson JE, Liu SM. A prospective study of red meat consumption and type 2 diabetes in middle-aged and elderly women. Diabetes Care. 2004;27(9):2108-15.

232. Steffen BT, Steffen LM, Zhou X, Ouyang P, Weir NL, Tsai MY. n-3 Fatty acids attenuate the risk of diabetes associated with elevated serum nonesterified fatty acids: the multi-ethnic study of atherosclerosis. Diabetes Care. 2015;38(4):575-80.

233. Struijk EA, Heraclides A, Witte DR, Soedamah-Muthu SS, Geleijnse JM, Toft U, Lau CJ. Dairy product intake in relation to glucose regulation indices and risk of type 2 diabetes. Nutrition, metabolism, and cardiovascular diseases : NMCD. 2013;23(9):822-8.

234. Stuber JM, Vissers LET, Verschuren WMM, Boer JMA, van der Schouw YT, Sluijs I. Substitution among milk and yogurt products and the risk of incident type 2 diabetes in the EPIC-NL cohort. J Hum Nutr Diet. 2020.

235. Tso AW, Xu A, Sham PC, Wat NM, Wang Y, Fong CH, et al. Serum adipocyte fatty acid binding protein as a new biomarker predicting the development of type 2 diabetes: a 10-year prospective study in a Chinese cohort. Diabetes Care. 2007;30(10):2667-72.

236. van Dam RM, Stampfer M, Willett WC, Hu FB, Rimm EB. Dietary fat and meat intake in relation to risk of type 2 diabetes in men. Diabetes Care. 2002;25(3):417-24.

237. van Woudenbergh GJ, Kuijsten A, Tigcheler B, Sijbrands EJG, van Rooij FJA, Hofman A, et al. Meat Consumption and Its Association With C-Reactive Protein and Incident Type 2 Diabetes The Rotterdam Study. Diabetes Care. 2012;35(7):1499-505.

238. van Woudenbergh GJ, van Ballegooijen AJ, Kuijsten A, Sijbrands EJ, van Rooij FJ, Geleijnse JM, et al. Eating fish and risk of type 2 diabetes: A population-based, prospective follow-up study. Diabetes Care. 2009;32(11):2021-6.

239. Vang A, Singh PN, Lee JW, Haddad EH, Brinegar CH. Meats, processed meats, obesity, weight gain and occurrence of diabetes among adults: Findings from Adventist Health Studies. Annals of Nutrition and Metabolism. 2008;52(2):96-104.

240. Villegas R, Xiang YB, Elasy T, Li HL, Yang G, Cai H, et al. Fish, shellfish, and long-chain n-3 fatty acid consumption and risk of incident type 2 diabetes in middle-aged Chinese men and women. Am J Clin Nutr. 2011;94(2):543-51.

241. Virtanen JK, Mursu J, Tuomainen T-P, Virtanen HEK, Voutilainen S. Egg consumption and risk of incident type 2 diabetes in men: the Kuopio lschaemic Heart Disease Risk Factor Study. American Journal of Clinical Nutrition. 2015;101(5):1088-96.

242. Wallin A, Di Giuseppe D, Orsini N, Akesson A, Forouhi NG, Wolk A. Fish consumption and frying of fish in relation to type 2 diabetes incidence: a prospective cohort study of Swedish men. Eur J Nutr. 2017;56(2):843-52.

243. Wang Q, Xie T, Zhang T, Deng YJ, Zhang YY, Wu QF, et al. The Role of Changes in Cumulative Lipid Parameter Burden in the Pathogenesis of Type 2 Diabetes Mellitus: A Cohort Study of People Aged 35-65 Years in Rural China. Diabetes Metabolic Syndrome and Obesity-Targets and Therapy. 2022;15:1831-43.

244. Wang Y, Meng X, Deng X, Okekunle AP, Wang P, Zhang Q, et al. Postprandial Saturated Fatty Acids Increase the Risk of Type 2 Diabetes: A Cohort Study in a Chinese Population. J Clin Endocrinol Metab. 2018;103(4):1438-46.

245. Wang Y, Zhu J, Aroner S, Overvad K, Cai T, Yang M, et al. Plasma CD36 and Incident Diabetes: A Case-Cohort Study in Danish Men and Women. Diabetes Metab J. 2020;44(1):134-42.

246. Wang YL, Koh WP, Jensen MK, Yuan JM, Pan A. Plasma Fetuin-A Levels and Risk of Type 2 Diabetes Mellitus in A Chinese Population: A Nested Case-Control Study. Diabetes & Metabolism Journal. 2019;43(4):474-86.

247. Yashpal S, Liese AD, Boucher BA, Wagenknecht LE, Haffner SM, Johnston LW, et al. Metabolomic profiling of the Dietary Approaches to Stop Hypertension diet provides novel insights for the nutritional epidemiology of type 2 diabetes mellitus. Br J Nutr. 2021:1-11.

248. Yoshimoto M, Sakuma Y, Ogino J, Iwai R, Watanabe S, Inoue T, et al. Sex differences in predictive factors for onset of type 2 diabetes in Japanese individuals: A 15-year follow-up study. J Diabetes Investig. 2022.

249. Zafra-Tanaka JH, Miranda JJ, Gilman RH, Checkley W, Smeeth L, Bernabe-Ortiz A. Obesity markers for the prediction of incident type 2 diabetes mellitus in resource-poor settings: The CRONICAS Cohort Study. Diabetes Research and Clinical Practice. 2020;170.

250. Zong G, Liu G, Willett WC, Wanders AJ, Alssema M, Zock PL, et al. Associations Between Linoleic Acid Intake and Incident Type 2 Diabetes Among U.S. Men and Women. Diabetes Care. 2019;42(8):1406-13.

251. Aglago EK, Huybrechts I, Murphy N, Casagrande C, Nicolas G, Pischon T, et al. Consumption of Fish and Long-chain n-3 Polyunsaturated Fatty Acids Is Associated With Reduced Risk of Colorectal Cancer in a Large European Cohort. Clin Gastroenterol Hepatol. 2020;18(3):654-66.e6.

252. Akbaraly T, Wurtz P, Singh-Manoux A, Shipley MJ, Haapakoski R, Lehto M, et al. Association of circulating metabolites with healthy diet and risk of cardiovascular disease: analysis of two cohort studies. Sci Rep. 2018;8(1):8620.

253. Akesson A, Donat-Vargas C, Berglund M, Glynn A, Wolk A, Kippler M. Dietary exposure to polychlorinated biphenyls and risk of heart failure - A population-based prospective cohort study. Environment International. 2019;126:1-6.

254. Albala C, Villarroel A, Santos JL, Angel B, Lera L, Liberman C, et al. FABP2 Ala54Thr polymorphism and diabetes in Chilean elders. Diabetes Res Clin Pract. 2007;77(2):245-50.

255. Amarapurkar DN, Patel ND, Kamani PM. Evaluating risk factors for development of non-alcoholic steatohepatitis in type-II diabetes mellitus. Hepatitis Monthly. 2008;8(3):197-200.

256. Baulderstone L, Yaxley A, Luszcz M, Miller M. Diet Liberalisation in Older Australians Decreases Frailty without Increasing the Risk of Developing Chronic Disease. The Journal of frailty & aging. 2012;1(4):174-82.

257. Bennacer AF, Haffaf E, Kacimi G, Oudjit B, Koceir E-A. Association of polyunsaturated/saturated fatty acids to metabolic syndrome cardiovascular risk factors and lipoprotein (a) in hypertensive type 2 diabetic patients. Annales De Biologie Clinique. 2017;75(3):293-304.

258. Bigornia SJ, Lichtenstein AH, Harris WS, Tucker KL. Associations of erythrocyte fatty acid patterns with insulin resistance. Am J Clin Nutr. 2016;103(3):902-9.

259. Block RC, Liu L, Herrington DM, Huang S, Tsai MY, O'Connell TD, Shearer GC. Predicting Risk for Incident Heart Failure With Omega-3 Fatty Acids: From MESA. JACC Heart Fail. 2019;7(8):651-61.

260. Borgeraas H, Hertel JK, Seifert R, Berge RK, Bohov P, Ueland PM, et al. Serum trans fatty acids, asymmetric dimethylarginine and risk of acute myocardial infarction and mortality in patients with suspected coronary heart disease: a prospective cohort study. Lipids in health and disease. 2016;15:38.

261. Borges MC, Haycock PC, Zheng J, Hemani G, Holmes MV, Smith GD, et al. Role of circulating polyunsaturated fatty acids on cardiovascular diseases risk: analysis using Mendelian randomization and fatty acid genetic association data from over 114,000 UK Biobank participants. Bmc Medicine. 2022;20(1).

262. Byrne CD, Maison P, Halsall D, Martensz N, Hales CN, Wareham NJ. Cross-sectional but not longitudinal associations between non-esterified fatty acid levels and glucose intolerance and other features of the metabolic syndrome. Diabetic medicine : a journal of the British Diabetic Association. 1999;16(12):1007-15.

263. Cabout M, Alssema M, Nijpels G, Stehouwer CDA, Zock PL, Brouwer IA, et al. Circulating linoleic acid and alpha-linolenic acid and glucose metabolism: the Hoorn Study. Eur J Nutr. 2017;56(6):2171-80.

264. Chang AR, Lazo M, Appel LJ, Gutierrez OM, Grams ME. High dietary phosphorus intake is associated with all-cause mortality: results from NHANES III. Am J Clin Nutr. 2014;99(2):320-7.

265. Chen HY, Cairns BJ, Small AM, Burr HA, Ambikkumar A, Martinsson A, et al. Association of FADS1/2 Locus Variants and Polyunsaturated Fatty Acids With Aortic Stenosis. JAMA Cardiol. 2020;5(6):1-9.

266. de Mello VD, Selander T, Lindstrom J, Tuomilehto J, Uusitupa M, Kaarniranta K. Serum Levels of Plasmalogens and Fatty Acid Metabolites Associate with Retinal Microangiopathy in Participants from the Finnish Diabetes Prevention Study. Nutrients. 2021;13(12).

267. Dearborn JL, Qiao Y, Guallar E, Steffen LM, Gottesman RF, Zhang Y, Wasserman BA. Polyunsaturated fats, carbohydrates and carotid disease: The Atherosclerosis Risk in Communities (ARIC) Carotid MRI study. Atherosclerosis. 2016;251:361-6.

268. Dehghan M, Mente A, Zhang X, Swaminathan S, Li W, Mohan V, et al. Associations of fats and carbohydrate intake with cardiovascular disease and mortality in 18 countries from five continents (PURE): a prospective cohort study. Lancet (London, England). 2017;390(10107):2050-62.

269. den Biggelaar L, Eussen S, Sep SJS, Mari A, Ferrannini E, van Greevenbroek MM, et al. Prospective associations of dietary carbohydrate, fat, and protein intake with -cell function in the CODAM study. European Journal of Nutrition. 2019;58(2):597-608.

270. Djousse L, Benkeser D, Arnold A, Kizer JR, Zieman SJ, Lemaitre RN, et al. Plasma free fatty acids and risk of heart failure: the Cardiovascular Health Study. Circulation Heart failure. 2013;6(5):964-9.

271. Hellstrand S, Ericson U, Gullberg B, Hedblad B, Orho-Melander M, Sonestedt E. Genetic variation in FADS1 has little effect on the association between dietary PUFA intake and cardiovascular disease. J Nutr. 2014;144(9):1356-63.

272. Higashioka M, Hirakawa Y, Kawamura R, Honda T, Hata J, Yoshida D, et al. Ratios of serum eicosapentaenoic acid to arachidonic acid and docosahexaenoic acid to arachidonic acid were inversely associated with serum resistin levels: The Hisayama Study. J Diabetes Investig. 2020;11(2):482-9.

273. Houston DK, Ding J, Lee JS, Garcia M, Kanaya AM, Tylavsky FA, et al. Dietary fat and cholesterol and risk of cardiovascular disease in older adults: the Health ABC Study. Nutrition, metabolism, and cardiovascular diseases : NMCD. 2011;21(6):430-7.

274. Kaikkonen JE, Jula A, Viikari JSA, Juonala M, Hutri-Kahonen N, Kahonen M, et al. Associations of Serum Fatty Acid Proportions with Obesity, Insulin Resistance, Blood Pressure, and Fatty Liver: The Cardiovascular Risk in Young Finns Study. Journal of Nutrition. 2021;151(4):970-8.

275. Kim H, Anderson CA, Hu EA, Zheng Z, Appel LJ, He J, et al. Plasma Metabolomic Signatures of Healthy Dietary Patterns in the Chronic Renal Insufficiency Cohort (CRIC) Study. J Nutr. 2021;151(10):2894-907.

276. Kim YS, Xun P, Iribarren C, Van Horn L, Steffen L, Daviglus ML, et al. Intake of fish and long-chain omega-3 polyunsaturated fatty acids and incidence of metabolic syndrome among American young adults: a 25-year follow-up study. Eur J Nutr. 2016;55(4):1707-16.

277. Krishnan S, Steffen LM, Paton CM, Cooper JA. Impact of dietary fat composition on prediabetes: a 12-year follow-up study. Public health nutrition. 2017;20(9):1617-26.

278. Marklund M, Wu JHY, Imamura F, Del Gobbo LC, Fretts A, de Goede J, et al. Biomarkers of Dietary Omega-6 Fatty Acids and Incident Cardiovascular Disease and Mortality. Circulation. 2019;139(21):2422-36.

279. Parnell LD, Noel SE, Bhupathiraju SN, Smith CE, Haslam DE, Zhang X, et al. Metabolite patterns link diet, obesity, and type 2 diabetes in a Hispanic population. Metabolomics. 2021;17(10):88.

280. Pertiwi K, Küpers LK, Wanders AJ, de Goede J, Zock PL, Geleijnse JM. Associations of dairy and fiber intake with circulating odd-chain fatty acids in post-myocardial infarction patients. Nutr Metab (Lond). 2019;16:78.

281. Pranger IG, Muskiet FAJ, Kema IP, Singh-Povel C, Bakker SJL. Potential Biomarkers for Fat from Dairy and Fish and Their Association with Cardiovascular Risk Factors: Cross-sectional Data from the LifeLines Biobank and Cohort Study. Nutrients. 2019;11(5).

282. Saber H, Yakoob MY, Shi P, Longstreth W, Rimm EB, Lemaitre RN, et al. Circulating Phospholipid n-3 Polyunsaturated Fatty Acids and Incident Atherothrombotic and Cardioembolic Ischemic Stroke in 3 Large US Cohorts. Circulation. 2016;133.

283. Senftleber NK, Albrechtsen A, Lauritzen L, Diaz LJ, Ronn PF, Jorgensen ME. Omega-3 fatty acids and risk of first cardiovascular event in Greenlandic Inuit: A prospective cohort study. International Journal of Circumpolar Health. 2021;80.

284. Sheng GT, Kuang MB, Yang RJ, Zhong YJ, Zhang SH, Zou Y. Evaluation of the value of conventional and unconventional lipid parameters for predicting the risk of diabetes in a non-diabetic population. Journal of Translational Medicine. 2022;20(1).

285. Younossi ZM, Felix S, Jeffers T, Younossi E, Lam B, Nader F, et al. Serum Biomarkers are Associated With Atherosclerotic Cardiovascular Disease Among Patients With Nonalcoholic Fatty Liver Disease Undergoing Elective Angiography. Clin Gastroenterol Hepatol. 2022;20(5):e1149-e56.

286. More good news about the Mediterranean diet. Harvard women's health watch. 2014;21(8):8.

287. Abbott KA, Burrows TL, Thota RN, Alex A, Acharya S, Attia J, et al. Association between plasma phospholipid omega-3 polyunsaturated fatty acids and type 2 diabetes is sex dependent: The Hunter Community Study. Clin Nutr. 2020;39(4):1059-66.

288. Abbott KA, Veysey M, Lucock M, Niblett S, King K, Burrows T, Garg ML. Sex-dependent association between erythrocyte n-3 PUFA and type 2 diabetes in older overweight people. Br J Nutr. 2016;115(8):1379-86.

289. Adeva-Andany MM, Gonzalez-Lucan M, Fernandez-Fernandez C, Carneiro-Freire N, Seco-Filgueira M, Pedre-Pineiro AM. Effect of diet composition on insulin sensitivity in humans. Clinical Nutrition Espen. 2019;33:29-38.

290. Adeva-Andany MM, Ranal-Muino E, Vila-Altesor M, Fernandez-Fernandez C, Funcasta-Calderon R, Castro-Quintela E. Dietary habits contribute to define the risk of type 2 diabetes in humans. Clinical Nutrition Espen. 2019;34:8-17.

291. Azizi F, Hadaegh F, Hosseinpanah F, Mirmiran P, Amouzegar A, Abdi H, et al. Metabolic health in the Middle East and north Africa. The lancet Diabetes & endocrinology. 2019;7(11):866-79.

292. Bos MM, Noordam R, Bennett K, Beekman M, Mook-Kanamori DO, van Dijk KW, et al. Metabolomics analyses in non-diabetic middle-aged individuals reveal metabolites impacting early glucose disturbances and insulin sensitivity. Metabolomics. 2020;16(3).

293. Bradley CA. Diabetes: Omega-6 PUFAs and T2DM. Nature reviews Endocrinology. 2017;13(12):689.

294. Brouwer-Brolsma EM, Sluik D, Singh-Povel CM, Feskens EJM. Dairy product consumption is associated with pre-diabetes and newly diagnosed type 2 diabetes in the Lifelines Cohort Study. Br J Nutr. 2018;119(4):442-55.

295. Burgos SA, LaForce S, Zhao X. The role of dairy fat on cardiometabolic health: what is the current state of knowledge? Canadian Journal of Animal Science. 2019;99(3):429-41.

296. Carlsson M, Wessman Y, Almgren P, Groop L. High levels of nonesterified fatty acids are associated with increased familial risk of cardiovascular disease. Arteriosclerosis Thrombosis and Vascular Biology. 2000;20(6):1588-94.

297. Cespedes E, Baylin A, Campos H. Adipose tissue n-3 fatty acids and metabolic syndrome. European journal of clinical nutrition. 2015;69(1):114-20.

298. Chen G, Li Y, Zeng F, Deng G, Liang J, Wang J, et al. Biomarkers of fatty acids and risk of type 2 diabetes: a systematic review and meta-analysis of prospective cohort studies. Crit Rev Food Sci Nutr. 2020:1-14.

299. Chu NF, Stampfer MJ, Spiegelman D, Rifai N, Hotamisligil GS, Rimm EB. Dietary and lifestyle factors in relation to plasma leptin concentrations among normal weight and overweight men. International journal of obesity and related metabolic disorders : journal of the International Association for the Study of Obesity. 2001;25(1):106-14.

300. Del Gobbo LC, Imamura F, Aslibekyan S, Marklund M, Virtanen JK, Wennberg M, et al. omega-3 Polyunsaturated Fatty Acid Biomarkers and Coronary Heart Disease: Pooling Project of 19 Cohort Studies. JAMA internal medicine. 2016;176(8):1155-66.

301. Drehmer M, Odegaard AO, Schmidt MI, Duncan BB, Cardoso LdO, Alvim Matos SM, et al. Brazilian dietary patterns and the dietary approaches to stop hypertension (DASH) diet-relationship with metabolic syndrome and newly diagnosed diabetes in the ELSA-Brasil study. Diabetology & Metabolic Syndrome. 2017;9.

302. Haslam DE, Liang L, Wang DD, Kelly RS, Wittenbecher C, Pérez CM, et al. Associations of network-derived metabolite clusters with prevalent type 2 diabetes among adults of Puerto Rican descent. BMJ Open Diabetes Res Care. 2021;9(1).

303. Huang MC, Chang CI, Chang WT, Liao YL, Chung HF, Hsu CC, et al. Blood biomarkers of various dietary patterns correlated with metabolic indicators in Taiwanese type 2 diabetes. Food Nutr Res. 2019;63.

304. Huang X, Yan D, Xu M, Li F, Ren M, Zhang J, Wu M. Interactive association of lipopolysaccharide and free fatty acid with the prevalence of type 2 diabetes: A community-based cross-sectional study. J Diabetes Investig. 2019;10(6):1438-46.

305. Jiang H, Wang L, Wang D, Yan N, Li C, Wu M, et al. Omega-3 polyunsaturated fatty acid biomarkers and risk of type 2 diabetes, cardiovascular disease, cancer, and mortality. Clin Nutr. 2022;41(8):1798-807.

306. Kaur D, Tallman DA, Khosla P. The health effects of saturated fats - the role of whole foods and dietary patterns. Diabetes & Metabolic Syndrome-Clinical Research & Reviews. 2020;14(2):151-3.

307. Lee YB, Kim DH, Kim SM, Kim NH, Choi KM, Baik SH, et al. Risk of type 2 diabetes according to the cumulative exposure to metabolic syndrome or obesity: A nationwide population-based study. Journal of Diabetes Investigation.

308. Li N, Qiu Y, Wu Y, Zhang M, Lai Z, Wang Q, et al. Association of serum total fatty acids with type 2 diabetes. Clin Chim Acta. 2020;500:59-68.

309. Luo C, Liu H, Wang X, Xia L, Huang H, Peng X, et al. The associations between individual plasma SFAs, serine palmitoyl-transferase long-chain base subunit 3 gene rs680379 polymorphism, and type 2 diabetes among Chinese adults. Am J Clin Nutr. 2021;114(2):704-12.

310. Schulze MB, Minihane AM, Saleh RNM, Riserus U. Intake and metabolism of omega-3 and omega-6 polyunsaturated fatty acids: nutritional implications for cardiometabolic diseases. Lancet Diabetes & Endocrinology. 2020;8(11):915-30.

311. Stoeckli R, Keller U. Nutritional fats and the risk of type 2 diabetes and cancer. Physiology & behavior. 2004;83(4):611-5.

312. Thanopoulou AC, Karamanos BG, Angelico FV, Assaad-Khalil SH, Barbato AF, Del Ben MP, et al. Dietary fat intake as risk factor for the development of diabetes: multinational, multicenter study of the Mediterranean Group for the Study of Diabetes (MGSD). Diabetes Care. 2003;26(2):302-7.

313. Wei W, Zi T, Yang R, Xu J, Chen Y, Jiang X, et al. A Newly Developed Indicator of Overeating Saturated Fat Based on Serum Fatty Acids and Amino Acids and Its Association With Incidence of Type 2 Diabetes: Evidence From Two Randomized Controlled Feeding Trials and a Prospective Study. Front Nutr. 2022;9:897375.

314. Xuan Q, Hu C, Zhang Y, Wang Q, Zhao X, Liu X, et al. Serum lipidomics profiles reveal potential lipid markers for prediabetes and type 2 diabetes in patients from multiple communities. Front Endocrinol (Lausanne). 2022;13:966823.

315. Akter S, Kurotani K, Sato M, Hayashi T, Kuwahara K, Matsushita Y, et al. High Serum Phospholipid Dihomo-gamma-Linoleic Acid Concentration and Low Delta5-Desaturase Activity Are Associated with Increased Risk of Type 2 Diabetes among Japanese Adults in the Hitachi Health Study. J Nutr. 2017;147(8):1558-66.

316. Ardisson Korat AV, Malik VS, Furtado JD, Sacks F, Rosner B, Rexrode KM, et al. Circulating Very-Long-Chain SFA Concentrations Are Inversely Associated with Incident Type 2 Diabetes in US Men and Women. J Nutr. 2020;150(2):340-9.

317. Zong G, Sun Q, Yu D, Zhu J, Sun L, Ye X, et al. Dairy consumption, type 2 diabetes, and changes in cardiometabolic traits: a prospective cohort study of middle-aged and older Chinese in Beijing and Shanghai. Diabetes Care. 2014;37(1):56-63.

318. Alhazmi A, Stojanovski E, Garg ML, McEvoy M. Fasting whole blood fatty acid profile and risk of type 2 diabetes in adults: a nested case control study. PloS one. 2014;9(5):e97001.

319. Chiva-Blanch G, Giro O, Cofan M, Calle-Pascual AL, Delgado E, Gomis R, et al. Low Percentage of Vegetable Fat in Red Blood Cells Is Associated with Worse Glucose Metabolism and Incidence of Type 2 Diabetes. Nutrients. 2022;14(7).

320. Djousse L, Biggs ML, Lemaitre RN, King IB, Song X, Ix JH, et al. Plasma omega-3 fatty acids and incident diabetes in older adults. Am J Clin Nutr. 2011;94(2):527-33.

321. Fretts AM, Imamura F, Marklund M, Micha R, Wu JHY, Murphy RA, et al. Associations of circulating very-long-chain saturated fatty acids and incident type 2 diabetes: a pooled analysis of prospective cohort studies. American Journal of Clinical Nutrition. 2019;109(4):1216-23.

322. Imamura F, Fretts A, Marklund M, Ardisson Korat AV, Yang WS, Lankinen M, et al. Fatty acid biomarkers of dairy fat consumption and incidence of type 2 diabetes: A pooled analysis of prospective cohort studies. PLoS Med. 2018;15(10):e1002670.

323. Imamura F, Fretts AM, Marklund M, Ardisson Korat AV, Yang WS, Lankinen M, et al. Fatty acids in the de novo lipogenesis pathway and incidence of type 2 diabetes: A pooled analysis of prospective cohort studies. PLoS Med. 2020;17(6):e1003102.

324. Krachler B, Norberg M, Eriksson JW, Hallmans G, Johansson I, Vessby B, et al. Fatty acid profile of the erythrocyte membrane preceding development of Type 2 diabetes mellitus. Nutrition, metabolism, and cardiovascular diseases : NMCD. 2008;18(7):503-10.

325. Kroeger J, Zietemann V, Enzenbach C, Weikert C, Jansen EHJM, Doering F, et al. Erythrocyte membrane phospholipid fatty acids, desaturase activity, and dietary fatty acids in relation to risk of type 2 diabetes in the European Prospective Investigation into Cancer and Nutrition (EPIC)-Potsdam Study. American Journal of Clinical Nutrition. 2011;93(1):127-42.

326. Lai HTM, Imamura F, Korat AVA, Murphy RA, Tintle N, Bassett JK, et al. Trans Fatty Acid Biomarkers and Incident Type 2 Diabetes: Pooled Analysis of 12 Prospective Cohort Studies in the Fatty Acids and Outcomes Research Consortium (FORCE). Diabetes Care. 2022;45(4):854-63.

327. Lankinen MA, Stancakova A, Uusitupa M, Agren J, Pihlajamaki J, Kuusisto J, et al. Plasma fatty acids as predictors of glycaemia and type 2 diabetes. Diabetologia. 2015;58(11):2533-44.

328. Lemaitre RN, Fretts AM, Sitlani CM, Biggs ML, Mukamal K, King IB, et al. Plasma phospholipid very-long-chain saturated fatty acids and incident diabetes in older adults: the Cardiovascular Health Study. Am J Clin Nutr. 2015;101(5):1047-54.

329. Lin JS, Dong HL, Chen GD, Chen ZY, Dong XW, Zheng JS, Chen YM. Erythrocyte Saturated Fatty Acids and Incident Type 2 Diabetes in Chinese Men and Women: A Prospective Cohort Study. Nutrients. 2018;10(10).

330. Miao Z, Lin JS, Mao Y, Chen GD, Zeng FF, Dong HL, et al. Erythrocyte n-6 Polyunsaturated Fatty Acids, Gut Microbiota, and Incident Type 2 Diabetes: A Prospective Cohort Study. Diabetes Care. 2020.

331. Mozaffarian D, Cao H, King IB, Lemaitre RN, Song X, Siscovick DS, Hotamisligil GS. Trans-palmitoleic acid, metabolic risk factors, and new-onset diabetes in U.S. adults: a cohort study. Annals of internal medicine. 2010;153(12):790-9.

332. Mozaffarian D, Otto MCdO, Lemaitre RN, Fretts AM, Hotamisligil G, Tsai MY, et al. trans-Palmitoleic acid, other dairy fat biomarkers, and incident diabetes: the Multi-Ethnic Study of Atherosclerosis (MESA). American Journal of Clinical Nutrition. 2013;97(4):854-61.

333. Qian F, Ardisson Korat AV, Imamura F, Marklund M, Tintle N, Virtanen JK, et al. n-3 Fatty Acid Biomarkers and Incident Type 2 Diabetes: An Individual Participant-Level Pooling Project of 20 Prospective Cohort Studies. Diabetes Care. 2021;44(5):1133-42.

334. Virtanen JK, Mursu J, Voutilainen S, Uusitupa M, Tuomainen T-P. Serum Omega-3 Polyunsaturated Fatty Acids and Risk of Incident Type 2 Diabetes in Men: The Kuopio Ischemic Heart Disease Risk Factor Study. Diabetes Care. 2014;37(1):189-96.

335. Wu JHY, Marklund M, Imamura F, Tintle N, Ardisson Korat AV, de Goede J, et al. Omega-6 fatty acid biomarkers and incident type 2 diabetes: pooled analysis of individual-level data for 39 740 adults from 20 prospective cohort studies. The lancet Diabetes & endocrinology. 2017;5(12):965-74.

336. Yakoob MY, Shi P, Willett WC, Rexrode KM, Campos H, Orav EJ, et al. Circulating Biomarkers of Dairy Fat and Risk of Incident Diabetes Mellitus Among Men and Women in the United States in Two Large Prospective Cohorts. Circulation. 2016;133(17):1645-54.

337. Yary T, Voutilainen S, Tuomainen TP, Ruusunen A, Nurmi T, Virtanen JK. Serum n-6 polyunsaturated fatty acids, Delta5- and Delta6-desaturase activities, and risk of incident type 2 diabetes in men: the Kuopio Ischaemic Heart Disease Risk Factor Study. Am J Clin Nutr. 2016;103(5):1337-43.

338. Zheng JS, Lin JS, Dong HL, Zeng FF, Li D, Song Y, Chen YM. Association of erythrocyte n-3 polyunsaturated fatty acids with incident type 2 diabetes in a Chinese population. Clin Nutr. 2019;38(5):2195-201.

339. Zhuang P, Liu X, Li Y, Li H, Zhang L, Wan X, et al. Circulating Fatty Acids and Genetic Predisposition to Type 2 Diabetes: Gene-Nutrient Interaction Analysis. Diabetes Care. 2022;45(3):564-75.

340. Pertiwi K, Wanders AJ, Harbers MC, Kupers LK, Soedamah-Muthu SS, de Goede J, et al. Plasma and Dietary Linoleic Acid and 3-Year Risk of Type 2 Diabetes After Myocardial Infarction: A Prospective Analysis in the Alpha Omega Cohort. Diabetes Care. 2020;43(2):358-65.

341. Song Z, Gao M, Lv J, Yu C, Guo Y, Bian Z, et al. Metabolically healthy obesity, transition to unhealthy phenotypes, and type 2 diabetes in 0.5 million Chinese adults: the China Kadoorie Biobank. Eur J Endocrinol. 2022;186(2):233-44.

342. Hodge AM, Karim MN, Hébert JR, Shivappa N, de Courten B. Association between Diet Quality Indices and Incidence of Type 2 Diabetes in the Melbourne Collaborative Cohort Study. Nutrients [Internet]. 2021; 13(11).

343. Feldman AL, Long GH, Johansson I, Weinehall L, Fhärm E, Wennberg P, et al. Change in lifestyle behaviors and diabetes risk: evidence from a population-based cohort study with 10 year follow-up. International Journal of Behavioral Nutrition and Physical Activity. 2017;14(1):39.
